# Supplementary material for: Inferring multimodal latent topics from electronic health records
Source: Nat Commun. 2020 May 21;11:2536. doi: 10.1038/s41467-020-16378-3 (PMC7242436; doi:10.1038/s41467-020-16378-3)
Supplement: Supplementary file 1 — Reporting Summary [file 41467_2020_16378_MOESM1_ESM.pdf]

nature research

Corresponding author(s): *Double-blind peer review submissions: write DBPR and your manuscript number here instead of author names.*

Last updated by author(s): *YYYY-MM-DD*

Reporting Summary

Nature Research wishes to improve the reproducibility of the work that we publish. This form provides structure for consistency and transparency in reporting. For further information on Nature Research policies, see Authors & Referees and the Editorial Policy Checklist.

Statistics

For all statistical analyses, confirm that the following items are present in the figure legend, table legend, main text, or Methods section.

n/a	Confirmed	
☐	☒	The exact sample size (*n*) for each experimental group/condition, given as a discrete number and unit of measurement
☐	☒	A statement on whether measurements were taken from distinct samples or whether the same sample was measured repeatedly
☐	☒	The statistical test(s) used AND whether they are one- or two-sided<br>*Only common tests should be described solely by name; describe more complex techniques in the Methods section.*
☐	☒	A description of all covariates tested
☐	☒	A description of any assumptions or corrections, such as tests of normality and adjustment for multiple comparisons
☐	☒	A full description of the statistical parameters including central tendency (e.g. means) or other basic estimates (e.g. regression coefficient) AND variation (e.g. standard deviation) or associated estimates of uncertainty (e.g. confidence intervals)
☐	☒	For null hypothesis testing, the test statistic (e.g. *F*, *t*, *r*) with confidence intervals, effect sizes, degrees of freedom and *P* value noted<br>*Give P values as exact values whenever suitable.*
☐	☒	For Bayesian analysis, information on the choice of priors and Markov chain Monte Carlo settings
☐	☒	For hierarchical and complex designs, identification of the appropriate level for tests and full reporting of outcomes
☐	☒	Estimates of effect sizes (e.g. Cohen's *d*, Pearson's *r*), indicating how they were calculated

Our web collection on statistics for biologists contains articles on many of the points above.

Software and code

Policy information about availability of computer code

Data collection	no software was used
Data analysis	The MixEHR software is implemented in C++ and R programming environment was used to analyze the data and generate the plots

For manuscripts utilizing custom algorithms or software that are central to the research but not yet described in published literature, software must be made available to editors/reviewers. We strongly encourage code deposition in a community repository (e.g. GitHub). See the Nature Research guidelines for submitting code & software for further information.

Data

Policy information about availability of data

All manuscripts must include a data availability statement. This statement should provide the following information, where applicable:

- Accession codes, unique identifiers, or web links for publicly available datasets
- A list of figures that have associated raw data
- A description of any restrictions on data availability

The MIMIC-III data analyzed in this manuscript are publicly available through PhysioNet (http://mimic.physionet.org). Mayo Clinic and Quebec CHD data are not publicly accessible due to restricted user agreement.

Field-specific reporting

Please select the one below that is the best fit for your research. If you are not sure, read the appropriate sections before making your selection.

☒ Life sciences ☐ Behavioural & social sciences ☐ Ecological, evolutionary & environmental sciences

For a reference copy of the document with all sections, see nature.com/documents/nr-reporting-summary-flat.pdf

Life sciences study design

All studies must disclose on these points even when the disclosure is negative.

Sample size	All of the samples were used for the analysis no filtering was performed.
Data exclusions	No data were excluded from the analysis.
Replication	All findings were produced by computer program and fully reproducible.
Randomization	This is not relevant to our study. The MIMIC-III data we used were prospective study and the patient cohort were not pre-screened for specific research.
Blinding	The investigators were blinded to the group allocation during the data collection and analysis.

Reporting for specific materials, systems and methods

We require information from authors about some types of materials, experimental systems and methods used in many studies. Here, indicate whether each material, system or method listed is relevant to your study. If you are not sure if a list item applies to your research, read the appropriate section before selecting a response.

Materials & experimental systems

n/a	Involved in the study	
☒	☐	Antibodies
☒	☐	Eukaryotic cell lines
☒	☐	Palaeontology
☒	☐	Animals and other organisms
☐	☒	Human research participants
☒	☐	Clinical data

Methods

n/a	Involved in the study	
☒	☐	ChIP-seq
☒	☐	Flow cytometry
☒	☐	MRI-based neuroimaging

Human research participants

Policy information about studies involving human research participants

Population characteristics	For the Mayo Clinic data, the 187 patients (94 bipolar disorder cases and 93 controls) were selected from the Mayo Clinic Bipolar Disorder Biobank and the Mayo Clinic Biobank, respectively. There are 112 females (55 cases and 56 controls) and 76 males (38 cases and 38 controls). The ages are also matched. All of the subjects are Caucasian. Quebec Congenital Heart Disease (CHD) database contains 84,498 patients with CHD as the prospective cohort with 28 years of follow up from 1983 to 2010.
Recruitment	The participants with bipolar disorder were identified at the Mayo Clinic. The controls were selected by Joanna M. Biernacka from the Mayo Clinic Biobank by excluding any sign of bipolar disorder and matching the sex, age, ethnicity with the case subjects. The Quebec CHD database is an administrative database not sourced from patient recruitment.
Ethics oversight	This Mayo Clinic study was reviewed and approved by Mayo Clinic Institutional Review Board and by the access committees from the Mayo Clinic Bipolar Disorder Biobank and the Mayo Clinic Biobank. The participants had consented for research, including research using their EHR data (Frye et al., 2015; Olsen et al., 2013). The analysis on Quebec CHD database was reviewed and approved by the Research Ethics Board of McGill University Health Centre.

Note that full information on the approval of the study protocol must also be provided in the manuscript.
