# Supplementary material for: Inferring multimodal latent topics from electronic health records
Source: Nat Commun. 2020 May 21;11:2536. doi: 10.1038/s41467-020-16378-3 (PMC7242436; doi:10.1038/s41467-020-16378-3)
Supplement: Supplementary file 2 — Supplementary Information [file 41467_2020_16378_MOESM2_ESM.pdf]

Supplementary Information: Inferring multimodal latent topics of electronic health records

Yue Li[1,*], Pratheeksha Nair[1], Xing Han Lu[1], Zhi Wen[1], Yuening Wang[1], Amir Ardalan Kalantari Dehaghi[1], Yan Miao[1], Weiqi Liu[1], Tamas Ordog[2], Joanna M. Biernacka[3,4], Euijung Ryu[3], Janet E. Olson[3], Mark A. Frye[4], Aihua Liu[5], Liming Guo[5], Ariane Marelli[5], Yuri Ahuja[6], Jose Davila-Velderrain[6] and Manolis Kellis[6,7,*]

[1]School of Computer Science and McGill Centre for Bioinformatics, McGill University, Montreal, Quebec, H3A0E9, Canada
[2]Department of Physiology and Biomedical Engineering and Division of Gastroenterology and Hepatology, Department of Medicine, and Center for Individualized Medicine, Mayo Clinic, Rochester, Minnesota 55902, USA
[3]Department of Health Sciences Research, Mayo Clinic, Rochester, Minnesota 55902, USA
[4]Department of Psychiatry and Psychology, Mayo Clinic, Rochester, Minnesota 55902, USA
[5]McGill Adult Unit for Congenital Heart Disease Excellence (MAUDE Unit), Montreal, QC H4A 3J1, Canada
[6]Computer Science and Artificial Intelligence Lab, Massachusetts Institute of Technology, 32 Vassar St, Cambridge, Massachusetts 02139, USA
[7]The Broad Institute of Harvard and MIT, 415 Main Street, Cambridge, Massachusetts 02142, USA
[*]Correspondence to yueli@cs.mcgill.ca and manoli@mit.edu

Supplementary Methods

MixEHR generative model

MixEHR assumes the following generative process. The notations used below are summarized in **Supplementary Table** 3. Hyperparameters are sampled as follows:

$$\alpha_k \sim Gam(a_\alpha, b_\alpha) \tag{1}$$
$$\beta_{wt} \sim Gam(a_\beta, b_\beta) \tag{2}$$
$$\zeta_{lv} \sim Gam(a_\zeta, b_\zeta) \tag{3}$$
$$a_l \sim Gam(a_\psi b_\psi) \tag{4}$$
$$b_l \sim Gam(a_\psi b_\psi) \tag{5}$$

Global variables are sampled as follows:

$$\phi_k^{(t)} \sim Dir(\beta_t) : \frac{\Gamma(\sum_w \beta_{wt})}{\prod_w \Gamma(\beta_{wt})} \prod_w \phi_{wk}^{\beta_{wt}-1} \tag{6}$$
$$\eta_{lk} \sim Dir(\zeta_l) : \frac{\Gamma(\sum_v \zeta_{lv})}{\prod_v \Gamma(\zeta_{lv})} \prod_v \eta_{lkv}^{\zeta_{lv}-1} \tag{7}$$
$$\psi_{lk} \sim Bet(a_l, b_l) : \frac{\Gamma(a_l + b_l)}{\Gamma(a_l)\Gamma(b_l)} \psi_{lk}^{a_l-1}(1-\psi_{lk})^{b_l-1} \tag{8}$$

Local variables for binary EHR features of each patient are sampled as follows:

$$\theta_j \sim Dir(\alpha) : \frac{\Gamma(\sum_k \alpha_k)}{\prod_k \Gamma(\alpha_k)} \prod_k \theta_{jk}^{\alpha_k-1} \tag{9}$$
$$z_{ij}^{(t)} \sim Mul(\theta_j) : \prod_k \theta_{jk}^{[z_{ij}^{(t)}=k]} \tag{10}$$
$$x_{ij}^{(t)} \sim Mul(\phi_k^{(t)}) : \prod_w \phi_{kw}^{[x_{ij}^{(t)}=w]} \tag{11}$$

Local variables for each lab test and each patient are sampled as follows:

$$h_{lj} \sim Mul(\theta_j) : \prod_k \theta_{jk}^{[h_{lj}=k]} \tag{12}$$
$$y_{lj} \sim Mul(\eta_{lh_{lj}}) : \prod_v \eta_{lh_{lj}v}^{y_{ljv}} \tag{13}$$
$$r_{lj} \sim Bin(\psi_{lh_{lj}}) : \psi_{lh_{lj}}^{r_{lj}}(1-\psi_{lh_{lj}})^{1-r_{lj}} \tag{14}$$

where $Gam(.)$, $Dir(.)$, $Bet(.)$, $Mul(.)$, and $Bin(.)$ denote Gamma, Dirichlet, Beta, Multinomial, and Binomial distributions, respectively.

Joint collapsed variational Bayesian inference

To learn the model, we use Joint collapsed variational Bayesian inference. Treating the latent variables as missing data, we can express the complete joint likelihood based on our model as follows:

$$p(x, y, r, z, h, \theta, \phi, \psi, \eta|\alpha, \beta, \zeta, a, b) = \\ p(\theta|\alpha)p(z, h|\theta)p(x|z, \phi)p(\phi|\beta)p(r|h, \psi)p(y|h, \eta)p(\eta|\zeta)p(\psi|a, b) \tag{15}$$

Due to the conjugacy of Dirichlet prior variables ϕ, η, θ to the multinomial likelihood variables $x, y, \{z, h\}$, respectively, and the conjugacy of Beta prior variable ψ to binomial lab observation indicator variable r, we can perform a collapsed Bayesian inference by first analytically integrating out the Dirichlet and Beta variables and then infer directly the distribution of the latent variables $\{z, h\}$.

$$p(x, y, r, z, h|\alpha, \beta, \zeta, a, b) = \tag{16}$$
$$\int p(z, h|\theta)p(\theta|\alpha)d\theta \tag{17}$$
$$\int p(x|z, \phi)p(\phi|\beta)d\phi \tag{18}$$
$$\int p(y|h, \eta)p(\eta|\zeta)d\eta \tag{19}$$
$$\int p(r|h, \psi)p(\psi|a, b)d\psi \tag{20}$$

We can separately solve the above four integrals as follows.

$$\begin{aligned}
p(z, h|\alpha) &= \int p(\theta|\alpha)p(z, h|\theta)d\theta \\
&= \prod_j \int \underbrace{\frac{\Gamma(\sum_k \alpha_k)}{\prod_k \Gamma(\alpha_k)} \prod_k \theta_{jk}^{\alpha_k - 1}}_{p(\theta|\alpha)} \underbrace{\prod_{t=1}^{T} \prod_{i=1}^{M_j} \theta_{jk}^{[z_{ij}^{(t)}=k]} \prod_{l=1}^{L} \theta_{jk}^{[h_{lj}=k]}}_{p(z,h|\theta)} d\theta_j \\
&= \prod_j \frac{\Gamma(\sum_k \alpha_k)}{\prod_k \Gamma(\alpha_k)} \int \prod_k \theta_{jk}^{\alpha_k + n_{.jk}^{(.)} + m_{.jk}^{(.)} - 1} d\theta_j \\
&= \prod_j \frac{\Gamma(\sum_k \alpha_k)}{\prod_k \Gamma(\alpha_k)} \frac{\prod_k \Gamma(\alpha_k + n_{.jk}^{(.)} + m_{.jk}^{(.)})}{\Gamma(\sum_k \alpha_k + n_{.jk}^{(.)} + m_{.jk}^{(.)})} \underbrace{\int \frac{\Gamma(\sum_k \alpha_k + n_{.jk}^{(.)} + m_{.jk}^{(.)})}{\prod_k \Gamma(\alpha_k + n_{.jk}^{(.)} + m_{.jk}^{(.)})} \prod_k \theta_{ik}^{\alpha_k + n_{.jk}^{(.)} + m_{.jk}^{(.)} - 1} d\theta_j}_{\text{integrating over the entire density of } \theta \text{ equal to 1}} \\
&= \prod_j \frac{\Gamma(\sum_k \alpha_k)}{\prod_k \Gamma(\alpha_k)} \frac{\prod_k \Gamma(\alpha_k + n_{.jk}^{(.)} + m_{.jk}^{(.)})}{\Gamma(\sum_k \alpha_k + n_{.jk}^{(.)} + m_{.jk}^{(.)})}
\end{aligned}$$

Similar principle applies to the other three integrals:

$$
\begin{aligned}
p(x|z) &= \int p(\phi|\beta)p(x|z,\phi)d\phi \\
&= \prod_{k=1}^{K}\prod_{t=1}^{T}\int \frac{\Gamma(\sum_w \beta_{wt})}{\prod_w \Gamma(\beta_{wt})}\prod_w [\phi_{wk}^{(t)}]^{\beta_{wt}-1}\prod_{j=1}^{D}\prod_{i=1}^{M_j}[\phi_{wk}^{(t)}]^{[x_{ij}^{(t)}=w][z_{ij}^{(t)}=k]}d\phi_k^{(t)} \\
&= \prod_{k=1}^{K}\prod_{t=1}^{T}\int \frac{\Gamma(\sum_w \beta_{wt})}{\prod_w \Gamma(\beta_{wt})}\int \prod_w [\phi_{wk}^{(t)}]^{\beta_{wt}+n_{w.k}^{(t)}-1}d\phi_k^{(t)} \\
&= \prod_{k=1}^{K}\prod_{t=1}^{T}\int \frac{\Gamma(\sum_w \beta_{wt})}{\prod_w \Gamma(\beta_{wt})}\frac{\prod_w \Gamma(\beta_{wt}+n_{w.k}^{(t)})}{\Gamma(\sum_w \beta_{wt}+n_{w.k}^{(t)})}
\end{aligned}
$$

$$
\begin{aligned}
p(y|h,\zeta) &= \int p(\eta|\zeta)p(y|h,\eta)d\eta \\
&= \prod_{k=1}^{K}\prod_{l=1}^{L}\int \frac{\Gamma(\sum_v \zeta_v)}{\prod_v(\Gamma(\zeta_v)}\prod_v [\eta_{lkv}]^{\zeta_v-1}\prod_j\prod_l [\eta_{lkv}]^{[y_{lj}=v][h_{lj}=k]} \\
&= \prod_{k=1}^{K}\prod_{l=1}^{L}\frac{\Gamma(\sum_v \zeta_v)}{\prod_v(\Gamma(\zeta_v)}\int \prod_v [\eta_{lkv}]^{\zeta_{lv}+m_{lkv}-1}d\eta_{lk} \\
&\prod_{k=1}^{K}\prod_{l=1}^{L}\frac{\Gamma(\sum_v \zeta_v)}{\prod_v(\Gamma(\zeta_v)}\frac{\prod_v \Gamma(\zeta_{lv}+m_{lkv})}{\Gamma(\sum_v \zeta_{lv}+m_{lkv})}
\end{aligned}
$$

$$
\begin{aligned}
p(r|h,a,b) &= \int p(\psi|a,b)p(r|h,\psi)d\psi \\
&= \prod_{l=1}^{L}\prod_{k=1}^{K}\int \frac{\Gamma(a_l+b_l)}{\Gamma(a_l)\Gamma(b_l)}\psi_{lk}^{a_l-1}(1-\psi_{lk})^{b_l-1}\prod_j \psi_{lk}^{[h_{lj}=k][r_{lj}=1]}(1-\psi_{lk})^{[h_{lj}=k][r_{lj}=0]}d\psi_{lk} \\
&= \prod_l\prod_k \frac{\Gamma(a_l+b_l)}{\Gamma(a_l)\Gamma(b_l)}\int \psi_{lk}^{a_l+p_{lk}-1}(1-\psi_{lk})^{b_l+q_{lk}-1}d\psi_{lk} \\
&= \prod_l\prod_k \frac{\Gamma(a_l+b_l)}{\Gamma(a_l)\Gamma(b_l)}\frac{\Gamma(a_l+p_{lk})\Gamma(b_l+q_{lk})}{\Gamma(a_l+p_{lk}+b_l+q_{lk})}
\end{aligned}
$$

Together, we obtain the marginal likelihood as shown below:

$$p(x, y, r, z, h|\alpha, \beta, \zeta, a, b) = \tag{21}$$

$$\prod_j \frac{\Gamma(\sum_k \alpha_k)}{\prod_k \Gamma(\alpha_k)} \frac{\prod_k \Gamma(\alpha_k + n_{.jk}^{(.)} + m_{.jk})}{\Gamma(\sum_k \alpha_k + n_{.jk}^{(.)} + m_{.jk})} \tag{22}$$

$$\prod_k \prod_t \frac{\Gamma(\sum_w \beta_{wt})}{\prod_w \Gamma(\beta_{wt})} \frac{\prod_w \Gamma(\beta_t + n_{w.k}^{(t)})}{\Gamma(\sum_w \beta_{wt} + n_{w.k}^{(t)})} \tag{23}$$

$$\prod_k \prod_l \frac{\Gamma(\sum_v \zeta_{lv})}{\prod_v \Gamma(\zeta_{lv})} \frac{\prod_v \Gamma(\zeta_{lv} + m_{l.kv})}{\Gamma(\sum_v \zeta_{lv} + m_{l.kv})} \tag{24}$$

$$\prod_k \prod_l \frac{\Gamma(a_l + b_l)}{\Gamma(a_l)\Gamma(b_l)} \frac{\Gamma(a_l + p_{lk})\Gamma(b_l + q_{lk})}{\Gamma(a_l + p_{lk} + b_l + q_{lk})} \tag{25}$$

where

$$n_{.jk}^{(.)} = \sum_{t=1}^{T} \sum_{i=1}^{M_j^{(t)}} [z_{ij}^{(t)} = k] \tag{26}$$

$$n_{w.k}^{(t)} = \sum_{j=1}^{D} \sum_{i=1}^{M_{j'}^{(t)}} [x_{ij}^{(t)} = w, z_{ij}^{(t)} = k] \tag{27}$$

$$m_{.jk.} = \sum_{l=1}^{L} \sum_{v=1}^{V_l} y_{ljv}[h_{lj} = k] \tag{28}$$

$$m_{l.kv} = \sum_{j=1}^{D} y_{ljv}[h_{lj} = k] \tag{29}$$

$$p_{lk} = \sum_j [r_{lj} = 1] \sum_v y_{ljv}[h_{lj} = k] \tag{30}$$

$$q_{lk} = \sum_j [r_{lj} = 0] \sum_v [h_{lj} = k, y_{lj} = v] \tag{31}$$

Note that we use y_{ljv} to denote frequency that the lab test l of outcome v for patient j and $[y_{lj} = v]$ as binary indicator of a single test.

Conditional distribution of latent variables

We derive the conditional distribution of latent variables $z_{ij}^{(t)} = k$ as follows:

$$p(z_{ij}^{(t)} = k | z_{-(ij)}, x, r, y, h, \psi, \eta) \tag{32}$$

$$= \frac{p(x, r, y, z_{ij}^{(t)} = k, z_{-(ij)}, h, \psi, \eta)}{\sum_{k'=1}^{K} p(x, r, y, z_{ij} = k', z_{-(ij)}, h, \psi, \eta)} \tag{33}$$

$$\propto p(z_{ij}^{(t)} = k, z_{-(ij)}, h | \alpha) p(x | z_{ij}^{(t)} = k, z_{-(ij)}, \beta) \tag{34}$$

$$\propto \frac{\prod_k \Gamma(\alpha_k + n_{.jk}^{(.)} + m_{.jk})}{\Gamma(\sum_k \alpha_k + n_{.jk}^{(.)} + m_{.jk})} \prod_k \frac{\Gamma(\beta_{tx_{ij}} + n_{w.k}^{(t)})}{\Gamma(\sum_w \beta_{wt} + n_{w.k}^{(t)})} \tag{35}$$

$$\propto (\alpha_k + [n_{.jk}^{(.)}]^{-(i,j)} + m_{.jk}^{(.)}) \frac{\beta_{wt} + [n_{x_{ij}^{(}t).k}]^{-(i,j)}}{\sum_w \beta_{wt} + [n_{w.k}]^{-(i,j)}} \tag{36}$$

where the derivations from (35) to (36) are as follows:

$$\frac{\prod_k \Gamma(\alpha_k + n_{.jk}^{(.)} + m_{.jk})}{\Gamma(\sum_k \alpha_k + n_{.jk}^{(.)} + m_{.jk})} \tag{37}$$

$$= \frac{\prod_{k' \neq k} \Gamma(\alpha_{k'} + [n_{.jk'}^{(.)}]^{-(i,j)} + m_{.jk'}^{(.)}) \Gamma(\alpha_k + [n_{.jk}^{(.)}]^{-(i,j)} + m_{.jk}^{(.)} + 1)}{\Gamma(\sum_k \alpha_k + n_{.jk}^{(.)} + m_{.jk}^{(.)}} \tag{38}$$

$$= \frac{\prod_{k=1}^{K} \Gamma(\alpha_k + [n_{.jk}^{(.)}]^{-(i,j)} + m_{.jk}^{(.)})(\alpha_k + [n_{.jk}^{(.)}]^{-(i,j)} + m_{.jk}^{(.)})}{\Gamma(\sum_k \alpha_k + n_{.jk}^{(.)} + m_{.jk}^{(.)})} \tag{39}$$

$$\propto \alpha_k + [n_{.jk}^{(.)}]^{-(i,j)} + m_{.jk}^{(.)} \tag{40}$$

$$\tag{41}$$

$$\prod_k \frac{\Gamma(\beta_{tx_{ij}} + n_{w.k}^{(t)})}{\Gamma(\sum_w \beta_{wt} + n_{w.k}^{(t)})} \tag{42}$$

$$= \left(\prod_{k' \neq k} \frac{\Gamma(\beta_{tx_{ij}} + [n_{x_{ij}.k'}^{(t)}]^{-(i,j)})}{\Gamma(\sum_w \beta_{wt} + [n_{w.k'}^{(t)}]^{-(i,j)})} \right) \frac{\Gamma(\beta_{tx_{ij}} + [n_{x_{ij}.k}]^{-(i,j)} + 1)}{\Gamma(1 + \sum_w \beta_{wt} + [n_{w.k}]^{-(i,j)})} \tag{43}$$

$$= \left(\prod_{k=1} \frac{\Gamma(\beta_{tx_{ij}} + [n_{x_{ij}.k}^{(t)}]^{-(i,j)})}{\Gamma(\sum_w \beta_{wt} + [n_{w.k}^{(t)}]^{-(i,j)})} \right) \frac{\beta_{tx_{ij}} + [n_{x_{ij}.k}]^{-(i,j)}}{\sum_w \beta_{wt} + [n_{w.k}]^{-(i,j)}} \tag{44}$$

$$\propto \frac{\beta_{wt} + [n_{x_{ij}^{(}t).k}]^{-(i,j)}}{\sum_w \beta_{wt} + [n_{w.k}]^{-(i,j)}} \tag{45}$$

Note in the above derivation that we have used the property of $\Gamma(t+1) = t\Gamma(t)$ to cancel out terms not involving $z_{ij}^{(t)}$.

We derive the joint conditional distribution of $h_{lj} = k, y_{lj} = v$ as follows:

$$
\begin{aligned}
&p(h_{lj} = k, y_{lj} = v | x, r, y, z, h_{-(lj)}, \psi, \eta) && (46) \\
&= \frac{p(h_{lj} = k, y_{lj} = v, x, r, y, z, h_{-(lj)}, \psi, \eta)}{\sum_{k'=1}^{K} p(x, r, y, z, h_{lj} = k', h_{-(lj)}, \psi, \eta)} && (47) \\
&\propto p(z, h|\alpha) p(y|h, \zeta) p(r|y, h, a, b) && (48) \\
&\propto \frac{\prod_k \Gamma(\alpha_k + n_{.jk}^{(.)} + m_{.jk})}{\Gamma(\sum_k \alpha_k + n_{.jk}^{(.)} + m_{.jk})} \prod_k \frac{\prod_v \Gamma(\zeta_{lv} + m_{l.kv})}{\Gamma(\sum_v \zeta_{lv} + m_{l.kv})} \frac{\Gamma(a_l + p_{lk}) \Gamma(b_l + q_{lk})}{\Gamma(a_l + p_{lk} + b_l + q_{lk})} && (49) \\
&\propto \begin{cases} \left(\alpha_k + n_{jk} + m_{jk}^{-(l,j)}\right) \left(\frac{\zeta_{lv} + m_{l.kv}^{-(l,j)}}{\sum_v \zeta_{lv} + m_{l.kv}^{-(l,j)}}\right) \left(\frac{a_l + p_{lk}^{-(l,j)}}{a_l + p_{lk}^{-(l,j)} + b_l + q_{lk}}\right) & \text{if } r_{lj} = 1 \\ \left(\alpha_k + n_{jk} + m_{jk}^{-(l,j)}\right) \left(\frac{\zeta_{lv} + m_{lkv}^{-(l,j,v)}}{\sum_v \zeta_{lv} + m_{lkv}^{-(l,j,v)}}\right) \left(\frac{b_l + q_{lk}^{-(l,j)}}{a_l + p_{lk} + b_l + q_{lk}^{-(l,j)}}\right) & \text{if } r_{lj} = 0 \end{cases} && (50)
\end{aligned}
$$

where

$$
\begin{aligned}
\frac{\prod_k \Gamma(\alpha_k + n_{.jk}^{(.)} + m_{.jk})}{\Gamma(\sum_k \alpha_k + n_{.jk}^{(.)} + m_{.jk})} &= \frac{\prod_{k' \neq k} \Gamma(\alpha_{k'} + n_{.jk'}^{(.)} + m_{.jk'}^{-(l,j)}) \Gamma(\alpha_k + n_{jk} + m_{jk}^{-(l,j)} + 1)}{\Gamma(\sum_{k=1}^{K} \alpha_k + n_{jk} + m_{jk})} && (51) \\
&= \frac{\prod_{k=1} \Gamma(\alpha_k + n_{.jk}^{(.)} + m_{.jk}^{-(l,j)}) (\alpha_k + n_{jk} + m_{jk}^{-(l,j)})}{\Gamma(\sum_{k=1}^{K} \alpha_k + n_{jk} + m_{jk})} && (52) \\
&\propto \alpha_k + n_{jk} + m_{jk}^{-(l,j)} && (53) \\
& && (54)
\end{aligned}
$$

$$
\begin{aligned}
\prod_k \frac{\prod_v \Gamma(\zeta_{lv} + m_{l.kv})}{\Gamma(\sum_v \zeta_{lv} + m_{l.kv})} &= \prod_{k' \neq k} \frac{\prod_v \Gamma(\zeta_{lv} + m_{l.k'v}^{-(l,j)})}{\Gamma(\sum_v \zeta_{lv} + m_{l.k'v}^{-(l,j)})} \frac{\Gamma(\zeta_{lv} + m_{l.k'v}^{-(l,j)} + 1)}{\Gamma(\sum_v \zeta_{lv} + m_{l.k'v}^{-(l,j)} + 1)} && (55) \\
&= \prod_{k=1} \frac{\prod_v \Gamma(\zeta_{lv} + m_{l.kv}^{-(l,j)})}{\Gamma(\sum_v \zeta_{lv} + m_{l.kv}^{-(l,j)})} \frac{(\zeta_{lv} + m_{l.kv}^{-(l,j)})}{(\sum_v \zeta_{lv} + m_{l.kv}^{-(l,j)})} && (56) \\
&\propto \frac{(\zeta_{lv} + m_{l.kv}^{-(l,j)})}{(\sum_v \zeta_{lv} + m_{l.kv}^{-(l,j)})} && (57)
\end{aligned}
$$

$$\prod_k \frac{\Gamma(a_l + p_{lk})\Gamma(b_l + q_{lk})}{\Gamma(a_l + p_{lk} + b_l + q_{lk})} \tag{58}$$

$$= \prod_{k' \neq k} \frac{\Gamma(a_l + p_{lk'}^{-(l,j)})\Gamma(b_l + q_{lk'}^{-(l,j)})}{\Gamma(a_l + p_{lk'}^{-(l,j)} + b_l + q_{lk'}^{-(l,j)})} \frac{\Gamma(a_l + p_{lk'}^{-(l,j)} + [r_{lj} = 1])\Gamma(b_l + q_{lk'}^{-(l,j)} + [r_{lj} = 0])}{\Gamma(a_l + p_{lk'}^{-(l,j)} + b_l + q_{lk'}^{-(l,j)} + 1)} \tag{59}$$

$$= \begin{cases} \prod_{k=1} \frac{\Gamma(a_l+p_{lk}^{-(l,j)})\Gamma(b_l+q_{lk}^{-(l,j)})}{\Gamma(a_l+p_{lk}^{-(l,j)}+b_l+q_{lk}^{-(l,j)})} \frac{(a_l+p_{lk}^{-(l,j)})}{(a_l+p_{lk}^{-(l,j)}+b_l+q_{lk})} & \text{if } r_{lj} = 1 \\ \prod_{k=1} \frac{\Gamma(a_l+p_{lk'})\Gamma(b_l+q_{lk'}^{-(l,j)})}{\Gamma(a_l+p_{lk'}+b_l+q_{lk'}^{-(l,j)})} \frac{b_l+q_{lk}^{-(l,j)}}{(a_l+p_{lk}+b_l+q_{lk}^{-(l,j)})} & \text{if } r_{lj} = 0 \end{cases} \tag{60}$$

$$\propto \begin{cases} \frac{a_l+p_{lk}^{-(l,j)}}{a_l+p_{lk}^{-(l,j)}+b_l+q_{lk}} & \text{if } r_{lj} = 1 \\ \frac{b_l+q_{lk}^{-(l,j)}}{a_l+p_{lk}+b_l+q_{lk}^{-(l,j)}} & \text{if } r_{lj} = 0 \end{cases} \tag{61}$$

Joint collapsed variational Bayesian inference

The marginal likelihood can be approximated by evidence lower bound as follows:

$$\log p(x, y, r|\alpha, \beta, \zeta) = \log \sum_{z,h} \frac{p(x, y, z, h|\alpha, \beta, \zeta)}{q(z, h)} q(z, h) \tag{62}$$

$$\geq \sum_z q(z, h) \log p(x, y, z, h|\alpha, \beta, \zeta, a, b) - \sum_{z,h} q(z, h) \log q(z, h) \tag{63}$$

$$= \mathbb{E}_{q(z,h)}[\log p(x, y, z, h|\alpha, \beta, \zeta, a, b)] - \mathbb{E}_{q(z,h)}[\log q(z, h)] \tag{64}$$

$$\equiv \mathcal{L}_{ELBO} \tag{65}$$

Maximizing $\mathcal{L}_{ELBO}$ is equivalent to minimizing Kullback-Leibler (KL) divergence:

$$\mathcal{KL}[q(z, h)||p(z, h|x, y, r)] = \mathbb{E}_{q(z,h)}[\log q(z, h)] - \mathbb{E}_{q(z,h)}[\log p(z, h|x, y, r)] \tag{66}$$

$$= \mathbb{E}_{q(z,h)}[\log q(z, h)] - \mathbb{E}_{q(z,h)}[\log p(z, h, x, y)] + \log p(x, y, r) \tag{67}$$

by noting that the marginal likelihood $\log p(x, y, r)$ is constant and that $\mathcal{KL}[q(z, h)||p(z, h|x, y, r)] + \mathcal{L}_{ELBO} = \log p(x, y, r)$.

Under mean-field factorization, the proposed distribution of latent variables z and h are defined as:

$$\log q(z|\gamma) = \sum_{t,i,j,k} [z_{ij}^{(t)} = k] \log \gamma_{ijk}^{(t)} \tag{68}$$

$$\log q(h|\lambda) = \sum_{l,j,k} [h_{lj} = k] \log \lambda_{ljk} \tag{69}$$

Maximizing (64) with respect to the variational parameter $\gamma_{ijk}^{(t)}$ (or λ_{ljk}) is equivalent to calculating the expectation of $\gamma_{ijk}^{(t)}$ (or λ_{ljk}) with respect to all latent variables except for variable (i, j) (or

(l, j)) [1,2]:

$$\log \gamma_{ijk}^{(t)} = \mathbb{E}_{q(z^{-(i,j)})}[\log p(x, z)] \tag{70}$$

$$\log \lambda_{ljk} = \mathbb{E}_{q(h^{-(l,j)})}[\log p(y, r, h)] \tag{71}$$

or equivalently expressed by normalizing the distribution of $\gamma_{ijk}^{(t)}$ and λ_{ljk}:

$$\gamma_{ijk}^{(t)} = \frac{\exp(\mathbb{E}_{q(z^{-(i,j)})}[\log p(x, z)])}{\sum_k \exp(\mathbb{E}_{q(z^{-(i,j)})}[\log p(x, z)])} \tag{72}$$

$$\lambda_{ljk} = \frac{\exp(\mathbb{E}_{q(h^{-(l,j)})}[\log p(y, r, h)])}{\sum_k \exp(\mathbb{E}_{q(h^{-(l,j)})}[\log p(y, r, h)])} \tag{73}$$

Following the above conditional distribution in Equation (36), the expectation of the latent variable can be expressed in the same form:

$$\gamma_{ijk}^{(t)} \propto \left(\alpha_k + \tilde{n}_{.jk}^{-(i,j)} + \tilde{m}_{.jk}\right) \left(\frac{\beta_{tx_{ij}^{(t)}} + [\tilde{n}^{(t)}_{x_{ij}^{(t)}.k}]^{-(i,j)}}{\sum_w \beta_{wt} + [\tilde{n}^{(t)}_{w.k}]^{-(i,j)}}\right) \tag{74}$$

To infer $h_{lj} = k$, we will need to separately consider whether the lab test l is observed or missing. When the lab test l is observed for patient j at value v ($y_{lj} = v$), we have:

$$\lambda_{ljkv} \propto \left(\alpha_k + \tilde{n}_{.jk} + \tilde{m}_{.jk}^{-(l,j)}\right) \left(\frac{\zeta_{lv} + \tilde{m}_{lkv}^{-(l,j,v)}}{\sum_{v'} \zeta_{lv'} + \tilde{m}_{lkv'}^{-(l,j,v)}}\right) \left(\frac{a_l + \tilde{p}_{lk}^{-(l,j)}}{a_l + \tilde{p}_{lk}^{-(l,j)} + b_l + \tilde{q}_{lk}}\right) \tag{75}$$

When the lab test is missing for patient j, we will need to infer the joint distribution of $h_{lj} = k, y_{lj} = v$:

$$\pi_{ljkv} \propto \left(\alpha_k + \tilde{n}_{.jk} + \tilde{m}_{.jk}^{-(l,j)}\right) \left(\frac{\zeta_{lv} + \tilde{m}_{lkv}^{-(l,j,v)}}{\sum_{v'} \zeta_{lv'} + \tilde{m}_{lkv'}^{-(l,j,v)}}\right) \left(\frac{b_l + \tilde{q}_{lk}^{-(l,j)}}{a_l + \tilde{p}_{lk} + b_l + \tilde{q}_{lk}^{-(l,j)}}\right) \tag{76}$$

where we used the zero-order information from the second-order Taylor expansion to approxi-

mate the latent variable summations [2, 3]:

$$\tilde{n}_{x_{ij}^{(t)}.k}^{-(i,j)} = \sum_{j' \neq j}^{D} \sum_{i=1}^{M_{j'}^{(t)}} [x_{ij'}^{(t)} = x_{ij}^{(t)}] \gamma_{ij'k}^{(t)} \tag{77}$$

$$\tilde{n}_{.jk}^{-(i,j)} = \sum_{t=1}^{T} \sum_{i' \neq i}^{M_j^{(t)}} \gamma_{i'jk} \tag{78}$$

$$\tilde{m}_{.jk.}^{-(l,j)} = \sum_{l' \neq l}^{L} \sum_{v=1}^{V_{l'}} [r_{l'j} = 1] y_{l'jv} \lambda_{l'jkv} + [r_{l'j} = 0] \pi_{l'jkv} \tag{79}$$

$$\tilde{m}_{l.kv}^{-(l,j,v)} = \sum_{j=1}^{D} \sum_{l=1}^{L} \sum_{v' \neq v}^{V_l} [r_{lj} = 1] y_{ljv'} \lambda_{ljkv'} + [r_{lj} = 0] \pi_{ljkv'} \tag{80}$$

$$\tilde{p}_{lk}^{-(l,j)} = \sum_{j \neq j'} [r_{lj'} = 1] \sum_{v} y_{lj'v} \lambda_{lj'k} \tag{81}$$

$$\tilde{q}_{lk}^{-(l,j)} = \sum_{j \neq j'} [r_{lj'} = 0] \sum_{v} \pi_{ljkv} \tag{82}$$

Furthermore, we update the hyper-parameters by maximizing the marginal likelihood under the variational expectations via empirical Bayes fixed-point update [3, 4]):

$$\alpha_k^* \leftarrow \frac{a_\alpha - 1 + \alpha_k \sum_j \Psi(\alpha_k + \tilde{n}_{jk} + \tilde{m}_{jk}) - \Psi(\alpha_k)}{b_\alpha + \sum_j \Psi(\tilde{n}_{jk} + \sum_k \alpha_k) - \Psi(\sum_k \alpha_k)} \tag{83}$$

$$\beta_{wt}^* \leftarrow \frac{a_\beta - 1 + \beta_{wt} \left(\sum_k \Psi(\beta_{wt} + n_{w.k}^{(t)}) - K W_t \Psi(\beta_{wt}) \right)}{b_\beta + \sum_k \Psi(W_t \beta_{wt} + \sum_w n_{w.k}^{(t)}) - K \Psi(W_t \beta_{wt})} \tag{84}$$

$$\zeta_{lv}^* \leftarrow \frac{a_\zeta - 1 + \zeta_{lv} \sum_k \Psi(\zeta_{lv} + m_{l.kv}) - K \Psi(\zeta_{lv})}{b_\zeta + \sum_k \Psi(\sum_v \zeta_{lv} + m_{l.kv}) - K \Psi(\sum_v \zeta_{kv})} \tag{85}$$

$$a_l^* \leftarrow \frac{a_\psi - 1 + a_l \left(\sum_k \Psi(a_l + p_{lk}) - K \Psi(a_l) \right)}{b_\psi + \sum_k \Psi(a_l + p_{lk} + b_l + q_{lk}) - K \Psi(a_l + b_l)} \tag{86}$$

$$b_l^* \leftarrow \frac{a_\psi - 1 + b_l \left(\sum_k \Psi(a_l + p_{lk}) - K \Psi(a_l) \right)}{b_\psi + \sum_k \Psi(a_l + p_{lk} + b_l + q_{lk}) - K \Psi(a_l + b_l)} \tag{87}$$

where the Gamma parameters are set to fixed values mainly for numerical stability: $a_\alpha = 1; b_\alpha = 0$, $a_\beta = 1, b_\beta = 100$, $a_\zeta = a_\psi = 1.001, b_\zeta = b_\psi = 0.001$.

In summary, the joint collapsed variational Bayesian zero-order (JCVB0) algorithm follows a variational Bayesian expectation maximization scheme as outlined in Algorithm 1.

for iteration n = 1 to N **do**

Update patient-feature variational parameters γ_{ijk} by Equation (74) and λ_{ljk} by Equation (75) when lab test l is tested on patient j or by Equation (76) for missing lab test

Update patient-topic variables:

$$\tilde{n}_{.jk} = \sum_{t=1}^{T} \sum_{i=1}^{M_j^{(t)}} \gamma_{ijk}^{(t)} \tag{88}$$

$$\tilde{m}_{.jk} = \sum_{l=1}^{L} \sum_{v=1}^{V_l} [r_{lj} = 1] y_{ljv} \lambda_{ljkv} + [r_{lj} = 0] \pi_{ljkv} \tag{89}$$

Update the global disease-topic variables:

$$\tilde{n}_{w.k}^{(t)} = \sum_{j=1}^{D} \sum_{i=1}^{M_j^{(t)}} [x_{ij}^{(t)} = w] \gamma_{ijk}^{(t)} \tag{90}$$

$$\tilde{m}_{l.kv} = \sum_{j=1}^{D} \sum_{l=1}^{L} [r_{lj} = 1] y_{ljv} \lambda_{ljkv} + [r_{lj} = 0] \pi_{ljkv} \tag{91}$$

$$\tilde{p}_{lk} = \sum_{j=1}^{D} [r_{lj} = 1] \sum_{v} y_{ljv} \lambda_{ljk} \tag{92}$$

$$\tilde{q}_{lk} = \sum_{j=1}^{D} [r_{lj} = 0] \sum_{v} \pi_{ljkv} \tag{93}$$

Update hyperparameters by Equation (83) to (87).

end for

Algorithm 1: Joint collapsed variational Bayesian zero-order (JCVB0) inference algorithm to simultaneously model the distribution of binary EHR variables under T distinct data types and lab test and test results using K latent topics.

Stochastic collapsed variational Bayesian

An issue with the above proposed JCVB0 is that, we need to keep track of $O(DMK + DLVK)$ $\lambda_{ljk}^{(t)}$'s in the variational Bayesian expectation step (VB E-step), where D number of patients, M is the number of non-lab tests (e.g., clinical note vocabulary, ICD-9 codes, etc), L is the total number of lab tests, V number of discrete test results, K the number of meta-phenotype disease topics. This is necessary for lab tests because we need to not only infer the latent variables of the observed tests but also the joint expectation of the missing lab tests for every patient in order to account for the non-missing at random (NMAR) biases in the data. This quickly becomes prohibitively large to fit into memory even with a small number of disease topics K for modest number of patients (e.g., ~40000 in our dataset). Although unlike lab tests, for binary phenotypes, we only need to keep track of MK $\gamma_{wjk}^{(t)}$'s, where M is the total number of observed patient-phenotype pairs, it also becomes intractable for large number of patients in the order of

millions.

To remedy this problem, we propose a stochastic joint collapsed variational Bayesian inference algorithm (SJCVB0). The main objective of our SJCVB0 is to avoid keeping track of all of the $\gamma_{ijk}^{(t)}$ and λ_{ljkv}'s while striking the balance between the accuracy and memory-efficiency.

As a brief review, the stochastic variational inference (SVI) algorithm operates by estimating the global update for λ_n at iteration n using a randomly sampled mini-batch of D' data points, where $D' \in [1, D]$ [5]. Denoting such intermediate estimate by $\hat{\lambda}_n$, then the global update is set by a weighted average of the current estimate and the intermediate estimate

$$\lambda_{n+1} = (1 - \rho_n)\lambda_n + \rho_n \hat{\lambda}_n \tag{94}$$

where $\rho_n = (n + \tau)^{-\kappa}$ is the "learning rate" that decreases as iteration increases [5].

The original SVI [5] operates on an uncollapsed variational lower bound of the LDA model. The SVI algorithm was then outperformed by the stochastic collapsed variational Bayesian zero-order algorithm (SCVB0) [6], which uses a hybrid mini-batch update strategy that scales the local and global updates using mini-batch towards total number of data points. Here our stochastic inference algorithm also operates on the collapsed evidence lower bound (i.e., integrating out the Dirichlet and Beta variables). However, our SJCVB0 differs from SCVB0 method in that it follows more closely the SVI framework, which has several good properties including guaranteed local convergence due to natural gradients and better predictive likelihood based on our initial assessment. Specifically, we describe the following distinctions between our proposed SJCVB0 and the existing SCVB0 [6].

As one of the main contributions of our method, we consider not only the observed lab tests but also the missing lab tests (by inferring the joint expectations of lab test and latent topic), which is important for EHR data due to the non-missing at random mechanisms. Our model generalizes to jointly modeling the multimodal distribution of multiple data matrices. We do not update local patient-topic parameters on a "per-token" basis and then scaled towards total tokens, which was the SCVB0 algorithm. The reason is because the EHR data are much noisier than corpus document. Rather, we update the local and global parameters on a "per-patient" basis, which with proper learning rate function has the convergence property that is proven by the original SVI framework [5]. Similarly, we do not scale up the global updates towards total patient size (i.e., by a scaler D/D' for $D > D'$ total patients and D' is size of the patient batch) as in SCVB0 as that assumes a somewhat homogeneous data population. Although the scaling approach works well for mining corpus text, it does not work in the case of EHR data because the patient healthcare records are highly heterogeneous and scaling the updates based only on a small subset of patient will result in unstable and poor convergence (as measured by the predictive log likelihood of the held-out patients). We do not approximate the inference of $\gamma_{ijk}^{(t)}$ as in SCVB0 but rather use the variational expectations as in our JCVB0 algorithm (i.e., discounting patient j and phenotype i when updating $\gamma_{ijk}^{(t)}$), which guarantees minimizing the KL-divergence.

We outline our stochastic variational inference algorithm in Algorithm 2:

for iteration n = 1 to N **do**

Sample a minibatch of D' patients

Update patient-feature variational parameters $\gamma_{ijk}^{(t)}$ and λ_{ljkv} the same way as in Algorithm 1

Update patient-topic sufficient statistics $\tilde{n}_{.j'k}$ for each patient j' in the mini-batch the same way as in Algorithm 1

Obtain intermediate updates $\hat{n}_{w.k}^{(t)}$ and $\hat{m}_{l.kv}$ of feature-topic variables as in Algorithm 1 but using only the patient mini-batch:

$$\hat{n}_{w.k}^{(t)} = \sum_{j'=1}^{D'} \sum_{i=1}^{M_{j'}^{(t)}} [x_{ij'}^{(t)} = w]\gamma_{ij'k}^{(t)} \tag{95}$$

$$\hat{m}_{l.kv} = \sum_{j'=1}^{D'} \sum_{l=1}^{L} [r_{lj'} = 1] y_{lj'v} \lambda_{lj'kv} + [r_{lj'} = 0]\pi_{lj'kv} \tag{96}$$

Update global feature-topic variables using the estimates:

$$\hat{n}_{w.k}^{(t)} = (1-\rho)\tilde{n}_{w.k}^{(t)} + \rho\hat{n}_{w.k}^{(t)} \tag{97}$$

$$\hat{m}_{l.kv} = (1-\rho)\tilde{m}_{l.kv}^{(t)} + \rho\hat{m}_{l.kv}^{(t)} \tag{98}$$

Obtain intermediate updates for hyperparameters as in Algorithm 1 but using the patient mini-batch

$$\alpha_k^* = (1-\rho)\alpha_k + \rho\hat{\alpha}_k \tag{99}$$

$$\beta_t^* = (1-\rho)\beta_t + \rho\hat{\beta}_t \tag{100}$$

$$\zeta_{lv}^* = (1-\rho)\zeta_{lv} + \rho\hat{\zeta}_{lv} \tag{101}$$

$$a_l^* = (1-\rho)a_l + \rho\hat{a}_l \tag{102}$$

$$b_l^* = (1-\rho)b_l + \rho\hat{b}_l \tag{103}$$

end for

Algorithm 2: Stochastic joint collapsed variational Bayesian zero-order (SJCVB0) inference algorithm using patient mini-batch without keeping track of all of the latent variables.

Time complexity and practical runtime

In theory, the computational complexity of our model is the same as the standard LDA model: O(DKJ). Here D is the number of documents (i.e., patients/admissions), K is the number of topics, and J is the size of the vocabulary (i.e., the total number of distinct EHR codes across all data types). In practice, because the actual EHR data are extremely sparse, only a very small fraction of the EHR codes is observed in any given patient's admission. Also, our model employs collapsed variational inference such that the topic inference operates on the counts of the distinct EHR code for each patient. Therefore, we can formulate the time complexity based on topics and the total number of distinct pairs of patient and EHR-code observed in the training data: O(MK).

However, this does not apply for laboratory tests. The reason is because of the non-missing-at-randomness (NMAR) in the data. To account for NMAR, we not only model the observed lab results but also impute the missing lab results for every patient during the training. Therefore, the time complexity for D patients, K topics, and L lab tests each with V discrete values is O(DKLV).

Together, we can conclude the time complexity for the model is O(MK + DKLV), where M is the number of unique admission-EHR pairs for the non-lab test data, K is the number of topics, D is the number of patients, L is the number of lab tests, and V is the number of discrete lab test values per lab test.

In practice, our C++ implementation uses efficient numerical libraries such as Armadillo and Boost (source code: `https://github.com/li-lab-mcgill/mixehr`). We also leverage OpenMP to harness multi-core CPUs. This allows us to perform the inference step over multiple patients/admissions simultaneously (one CPU core per patient). Compared to a single-core CPU machine, we observed that the speed improvement on a multi-core CPU machine is almost linear to the number of cores. For example, if training EHR data takes 20 hours on a single core machine, training the same data set will take 2 hours on a 10-core machine.

When training a 75-topic model on the MIMIC-III data with 12-16 million observations over 50,000 admissions on a 2.2 GHz 20-core Xeon intel CPU server with 128 GB of RAM, MixEHR converges within 3 hours with 1000 iterations. We achieved similar runtime training on a 50-topic MixEHR model on the Quebec CHD data with 80,000 patients over 13 million out-patient visits for about 4 hours upon convergence. Additionally, our stochastic variational inference version of the MixEHR does not depend on the sample size N but rather updates the model on small mini-batch of patients, further improving the efficiency and scalability on large EHR dataset.

MixEHR+RNN on predicting diagnostic code from the 28-year Quebec CHD dataset

Supplementary Figure 21 depicts the architecture of the proposed MixEHR+RNN and the baseline RNN framework. Details are described below.

Similarly to Doctor AI [7], for all patient p and time step t, the objective of the training process is to minimize the loss function $L(y^p_{d,t}, \widehat{y}^p_{d,t})$.

$$L(y^p_t, \widehat{y}^p_t) = \sum_{d=1}^{D} \sum_{t=1}^{N_p-1} (y^p_{d,t} \log(\widehat{y}^p_{d,t}) + (1 - y^p_{d,t}) \log(1 - \widehat{y}^p_{d,t})) \tag{104}$$

Here, $y^p_{d,t} \in \{0, 1\}$, for a total of D diagnoses groups, and $\widehat{y}^p_{d,t}$ is the output of an activation function applied on the logit $a^{(J)}_{d,t}$ of the last fully-connected layer J for disease code d at visit block t. In the Doctor AI paper [7], the activation is the softmax function:

$$f(a^{(J)}_{d,t}) = \frac{\exp(a^{(J)}_{d,t})}{\sum_d \exp(a^{(J)}_{d,t})} \tag{105}$$

In contrast, we use the sigmoid function:

$$\sigma(a^{(J)}_{d,t}) = \frac{1}{1 + \exp(-a^{(J)}_{d,t})} \tag{106}$$

Although the softmax function (105) ensures that the sum across all D dimensions adds up to 1, sigmoid function $\sigma(.)$ ensures that every disease code of $y^p_{d,t}$ will be a value between 0 and 1, with no restriction on the summation. Therefore, the model will not be penalized if it predicts correctly the positive label with probability equal to one for a patient with more than more EHR code observed.

The Gated Recurrent Unit (GRU), presented in [8, 9], are expressed as follow:

$$\mathbf{z}^l_t = \sigma(\mathbf{W}^l_z \mathbf{h}^{l-1}_t + \mathbf{U}^l_z \mathbf{h}^l_{t-1} + b^l_z) \tag{107}$$
$$\mathbf{r}^l_t = \sigma(\mathbf{W}^l_r \mathbf{h}^{l-1}_t + \mathbf{U}^l_r \mathbf{h}^l_{t-1} + b^l_r) \tag{108}$$
$$\tilde{\mathbf{h}}^l_t = \tanh(\mathbf{W}^l_h \mathbf{h}^{l-1}_t + \mathbf{r}^l_t \circ \mathbf{U}^l_h \mathbf{h}^l_{t-1} + b^l_h) \tag{109}$$
$$\mathbf{h}^l_t = \mathbf{z}^l_t \circ \mathbf{h}^l_{t-1} + (1 - \mathbf{z}^l_t) \circ \tilde{\mathbf{h}}^l_t \tag{110}$$

where l indicates the l-th layer of the stacked GRUs (with a total of L layers), $\mathbf{h}^{(0)}_t = \mathbf{x}_t$ is the embedded input to the GRU, and $\circ$ indicates element-wise product. Also, $\mathbf{W}^l_z, \mathbf{U}^l_z, \mathbf{W}^l_r, \mathbf{U}^l_r, b^l_z, b_r$ are all learned using backpropagation, and are different for every layer of the GRU. For simplicity, we denote the last hidden layer $\mathbf{h}^L_t$ as $\mathbf{g}_t$.

We denote $\mathbf{z}_t^j$ as the output of the j-th dense layer, and

$$\begin{aligned}\mathbf{e}_t &= ReLU(\mathbf{W}_e\mathbf{x}_t + b_e)\\ \mathbf{g}_t &= GRU(\mathbf{e}_t)\\ \mathbf{m}_t &= MXR(\mathbf{x}_t)\\ \mathbf{z}_t^{(0)} &= [\mathbf{g}_t, \mathbf{m}_t]\end{aligned}$$

where $ReLU(x) = max\{0, x\}$ is the rectifier activation function [10] and $MXR(\mathbf{x}_t)$ is the topic mixture embedding of input $\mathbf{x}_t$ at visit block t. Furthermore, the same weights are applied in parallel to each of the time step t in patient p's history.

Then, another sets of dense layers $\mathbf{W}_d^j, b_d^j$ for $j = 1, \dots, J$ (J=2) were applied to the concatenation of the GRU layer's output and MixEHR's output:

$$\mathbf{z}_t^j = R(\mathbf{W}_d^j\mathbf{z}_t^{j-1} + b_d^j)$$

Finally, the last hidden layer J is passed to a sigmoid output:

$$\hat{\mathbf{y}}_t = \sigma(\mathbf{W}_o\mathbf{z}_t^J + b_o)$$

where $\hat{\mathbf{y}}_t \in \mathbb{R}^C$, for C classes (which corresponds to the number of ICD9 3-digit labels). In our model, $J = 2,\ L = 2$.

We decided to use the initialization scheme described in [10] to initialize all of the network weights due to its ability to speed up convergence. After every dense layer, dropout with probability $p = 0.5$ was applied in order to prevent overfitting. While Doctor AI used the Adadelta optimizer [11], we decided to use Adam [12] method, since the added momentum leads to faster convergence. We set the parameters to be $\alpha = 0.001,\ \beta_1 = 0.9,\ \beta_2 = 0.999,\ \epsilon = 10^{-8}$. All models were trained with a batch size of 64 samples for 50 epochs. Doctor AI originally proposes to have hidden units of dimensions 2000; in our paper, we propose to reduce that number to 128 (**Supplementary Figure** 21), which speeds up the computation time significantly, makes the model easier to fine-tune and less prone to overfitting.

Supplementary Notes 1

Lab imputation using the simulated MIMIC-III data

To evaluate the NMAR aspect of MixEHR, ideally we would need to know the underlying results of the truly missing lab tests for each patient, which is obviously not available. Therefore, we simulated EHR data from the specified generative model and evaluated MixEHR's NMAR treatment on this set. Specifically, we ran a 75-topic MixEHR on the full MIMIC-III data until convergence. We then used the patients' mixtures and trained model parameters to generate lab data as well as the other five EHR data types (**Supplementary Table** 1). Specifically, we sampled multinomial latent topic assignments for each EHR variable and each patient from the given patient topic mixtures, which were shared across all EHR variables. The missing rate of each lab test of each patient were simulated from a binomial distribution with the parameters specified by the MixEHR.

Because NMAR is only relevant to the lab data, we focused on evaluating the predictive log likelihood on the missing lab test (i.e., $\sum_{l,j}[r_{lj} = 1] \log \sum_k \hat{\theta}_{jk}(\hat{\psi}_{lk} + \hat{\eta}_{lky_{lj}})$. Specifically, we used the observed data to infer the mixture of the held-out patients and then used the inferred patient mixtures and the model parameters to impute the missing lab tests. Because the predictive log likelihood is the product of the normalized mixtures and normalized imputed lab results, it is independent of the model complexity and therefore an appropriate evaluation metric. As comparisons, we evaluated four different variations of the model: (1) MixEHR_nmar modeling NMAR using lab view only (Lab Only); (2) MixEHR_nmar using all EHR (All Data); (3) & (4) MixEHR_mar assuming the lab results are missing at random (MAR) using lab (Lab Only) and all EHR (All Data), respectively. For the MAR model we did not estimate the distribution of the observation biases, using only the observed lab results to update model parameters as opposed to using both observed and imputed missing results.

Five-fold cross-validation on the simulated data showed that while the training likelihoods increased in all models, only the NMAR model achieved increasing averaged predictive log likelihood on the missing lab tests for the held-out patients (**Supplementary Figure** 25a). Integrating both lab tests and auxiliary EHR data (i.e., clinical notes, ICD-9, prescriptions, etc) provided further improvements. Therefore, our model is robust to NMAR lab results.

We further compared our NMAR inference with MAR models, using the learned lab-result topics vs. ground truth lab-result topics used in the simulation, taking the highest correlation per topic (as topics are not identifiable). We find that MAR models confer much lower correlation (median 0.03) than our NMAR model (median 0.4) as they are biased by the observed data (**Supplementary Figure** 25b). Because of this bias, the predictive likelihood of MAR models on imputing the missing lab results is decreasing on held-out patients (**Supplementary Figure** 25a).

We also compared MixEHR lab result imputation with a popular imputation method, MICE (version 3.2.0) [13, 14], using default R settings for MICE [15] and 75 topics for MixEHR . MICE *separately* and *repeatedly* regresses on each lab variable using all of the other (imputed) variables and thus does not scale to the large-scale EHR data with over 53,000 clinical variables for over 38,000 patients, and thus we used only the 564 lab variables. Even in this subset, Mix-

EHR conferred much higher accuracy (median 85%) than MICE (median 50%) (**Supplementary Figure** 25c). This improvement may stem from our *joint* modeling approach that works well on sparse, high-dimensional, heterogeneous, and NMAR data. In contrast, MICE assumes dense, low-dimensional, homogeneous, and MAR data.

Supplementary Discussion

MixEHR builds on the concepts of collaborative filtering [16–20] and latent topic modeling [21–26]. In particular, our method is related to the widely popular text-mining method Latent Dirichlet Allocation (LDA) [21]. However, LDA is not well suited to EHR phenotyping because (1) LDA assumes that clinical "terms" are completely observed in the patient "document", whereas as mentioned above EHR data such as lab tests are often missing; (2) LDA is not suitable to jointly model heterogeneous EHR data categories with distinct distributions; and (3) even variations of LDA that deal with missing data do not cope with NMAR mechanisms [27]. As a result, the model is biased by the distribution of observed data, which may differ from the distribution of the missing data. Some existing approaches model the observation biases as a function of the results [19,28]. This is challenging because often the function dictating the missing mechanisms are unknown and likely to be both lab-specific and patient-specific.

Our method is also related to several EHR phenotyping studies focusing on matrix factorization and model interpretability [27,29–35]. Joshi et al. (2016) proposed a single-view non-probabilistic modeling of clinical notes, requiring predefined knowledge to learn interpretable disease topics [30]. Halpern et al. (2016) jointly model multiple data matrices but require expert-curated tagging data [29]. Gunasekar et al. (2016) proposed a sparsity induced collective non-negative matrix factorization (SiCNMF) approach on multi-source EHR data [32]. SiCNMF models multi-source data matrices by minimizing the Bregman divergence between the reconstructed and input matrices with L1-norm constraints on the basis matrices and the Frobenius norm constraint on the patient-level loading matrix. Pivovarov et al. (2015) proposed a multi-view LDA method with Gibbs sampling called UPhenome, which only works on patient records with a fixed set of four data types (i.e. notes, ICD-9, lab, and prescriptions, all observed jointly) [31]. Several recent reviews are helpful in gaining perspectives on the current machine learning advancements and challenges in healthcare [36–38].

Zhao et al. (2019) used a tensor non-negative factorization approach to model longitudinal EHR data as a tensor with patients, PheCodes (aggregates of ICD-9 code), and time dimensions [34]. In contrast, our MixEHR models not only ICD-9 code but also laboratory tests, medication, clinical notes, procedural code, and DRG code. While we use RNN to model the longitudinal EHR data using the corresponding time-dependent topic mixture due to its efficiency, it is also possible to model the time dimension in a tensor multi-modal topic model in future works.

Wang et al. (2020) described an interesting topic model called Poisson Dirichlet Model (PDM) [27]. In contrast to the multinomial likelihood in LDA and in our MixEHR, PDM uses Poisson likelihood to model the diagnosis counts of each patient. To account for age and sex confounders, the authors introduced a weighting factor as the fitted response value from a Generalized Additive Model on sex and age. The authors showed that PDM improves topic semantics over LDA. However, because of the non-conjugacy of Dirichlet to Poisson, the PDM model inference needs to be carried out by a much slower Metropolis-Hasting MCMC sampling. This limits its application to a much smaller number of patients (below 1000 patients), a much smaller number of diagnosis codes (around 1000 codes), also a smaller number of topics (10-30 topics). In contrast, our

variational collapsed Bayesian variation can easily scale to 50,000 patients and 50,000 EHR codes with 100 topics and converges within 3 hours on a multi-core CPU server. It would be interesting to compare the topic semantics inferred by MixEHR using a large cohort with those from PDM using the allowable much smaller samples, despite the lack of explicit weighting factor of age and sex in our model. Nonetheless, the idea of age and sex correction in PDM is an important point to address in our future work.

Our work is also related to the research in collaborative filtering (CF). In CF, we assign a low-dimensional feature vector to each user (or patient) and a low-dimensional feature vector to rating (e.g., movie rating or lab test results) so that the expected rating from each user is modelled by the scalar-product of the two feature vectors. Conditioned factored restricted Boltzmann Machine (CF-RBM) [16] is one of the state-of-the-art approaches in CF. In contrast to traditional approaches such as multiple imputation by chained equation (MICE) [15] that are not scalable to large-scale CF application, CF-RBM is scalable to large data set. This method trains a set of small RBMs only on the observed features (one RBM per user/ patient) and shares the input-hidden weights among users. CF-RBM achieved the state-of-art performance in collaborative filtering applications such as imputing Netflix movie ratings, outperforming the second best carefully fine-tuned singular vector decomposition (SVD) method. One drawback in CF-RBM is that it assumes that data are missing at random, which is an unrealistic assumption because of the aforementioned diagnosis-driven lab tests that often leads to non-missing-at-random data.

Recently developed deep learning methods primarily focus on prediction performance of target clinical outcomes [7, 39–45]. These methods mainly use recurrent neural networks (RNN) to model the sequential events for each patient. In EHR, these events are in-hospital admissions or outpatient visits, where the diagnostic codes, medical prescriptions, and interventions take place. One of the recently developed state-of-the-art deep learning frameworks called Graph-based Attention Model (GRAM) [46] uses self-attention mechanism to represent each ICD-9 code by its "ancestor" ICD-9 codes along the path from the observed code in a patient to the root of the tree. The tree is constructed based on the Clinical Classification Software (CCS) multi-level diagnosis hierarchy or the taxonomical tree-structure of the ICD-9 coding system. GRAM then uses the graph embedding to predict future diagnostic code at the next admission.

Apart from these methods, unsupervised deep learning were also applied to mine EHR data. In particular, Deep Patient [43] uses a stacked stochastic denoising autoencoder to model the EHR code by its latent embedding and demonstrates good performance on mining 700,000 patients' EHR data. Inspired by Deep patient [43], Wang et al used autoencoders to encode EHR codes along with their missing indicators. They then trained a logistic regression model on the features derived from the autoencoder to predict readmission [35].

Deep learning models often require large number of training examples and expensive computing resources such as graphical processing unit (GPU) servers. Also, for RNNs to work well, we also need to have a long history of patient medical information. In particular, although GRAM is able to operate on an existing knowledge graph to remedy the sparsity problem, it is ideal to apply GRAM to a large dataset. For example, Choi et al (2016, 2017) trained GRAM and Doctor AI models on over 250,000 patients [7, 46]. Unfortunately, access to such large data is often the

bottleneck due to regulatory policies that protects patient privacy. Model interpretability is also an ongoing challenge in deep learning [47], which is often paramount in clinical decision making.

Relative to these previous works, our work has several key advances: (1) we infer the joint distribution of data observation biases and the data themselves (i.e., lab tests) using latent topic distributions, thereby accounting for the distinct distributions of observed and missing data; (2) we model heterogeneity in EHR data types, allowing each data type to have its own distinct distribution, inferred separately from high-dimensional sparse data matrices; (3) we provide a efficient joint stochastic collapsed variational Bayesian (SCVB) inference algorithm for fast convergence to good local solutions, in contrast to the slower Gibbs sampling previously used [31]; (4) we infer the joint expectations of latent topics and lab results not only for observed, but also for missing lab tests, which enables us to account for the NMAR observation bias in EHR data, by extending CVB (which was originally developed for learning LDA model only [24–26]) . We further make the CVB scalable by enabling multi-core inference leveraging OpenMP library in order to mine massive-scale EHR datasets of millions of patient records, which to the best of our knowledge is not possible with existing EHR methods. Moreover, we introduce a way to impute missing EHR codes by k-nearest neighbour algorithm that finds similar patients based on the inferred patient disease mixtures, which (to the best of our knowledge) has been not demonstrated before. Lastly, we present a framework that combines MixEHR inferred topic mixture with RNN to model longitudinal EHR data, which is also more efficient than learning the dense layer embedding of an RNN from scratch.

Supplementary Figures

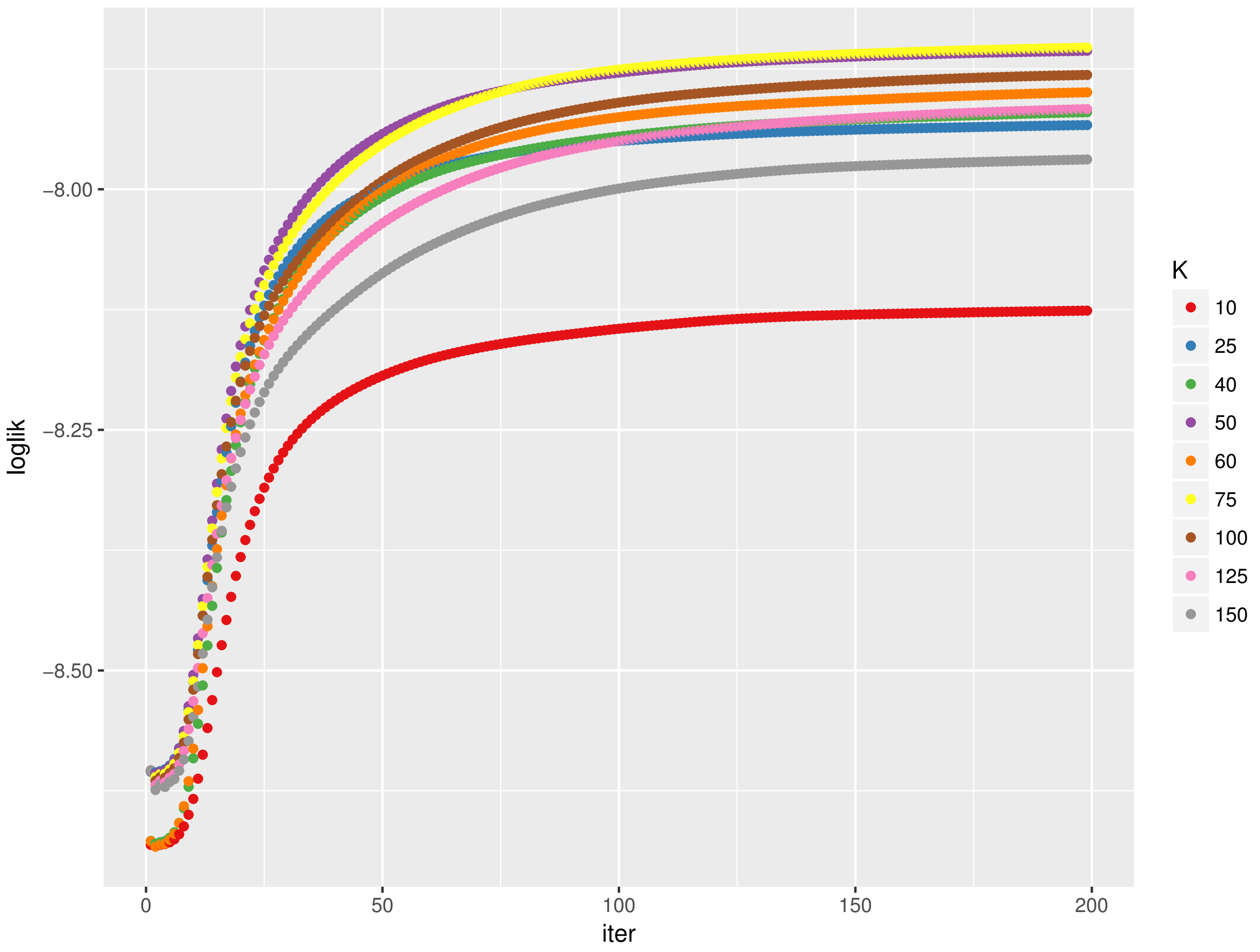

Figure 1: Averaged predictive likelihood. The predictive likelihood was evaluated on the held-out MIMIC-III patients by inferring the mixture memberships based on randomly chosen half of their EHR and evaluate the imputation over the other half. This is repeated a 5-fold cross-validation settings by training on 4 folds and evaluated on the held-out fold of patients and averaged over the 5 folds. The averaged likelihood was then plotted as a function of model iterations. Different colors indicate different number of topics (K).

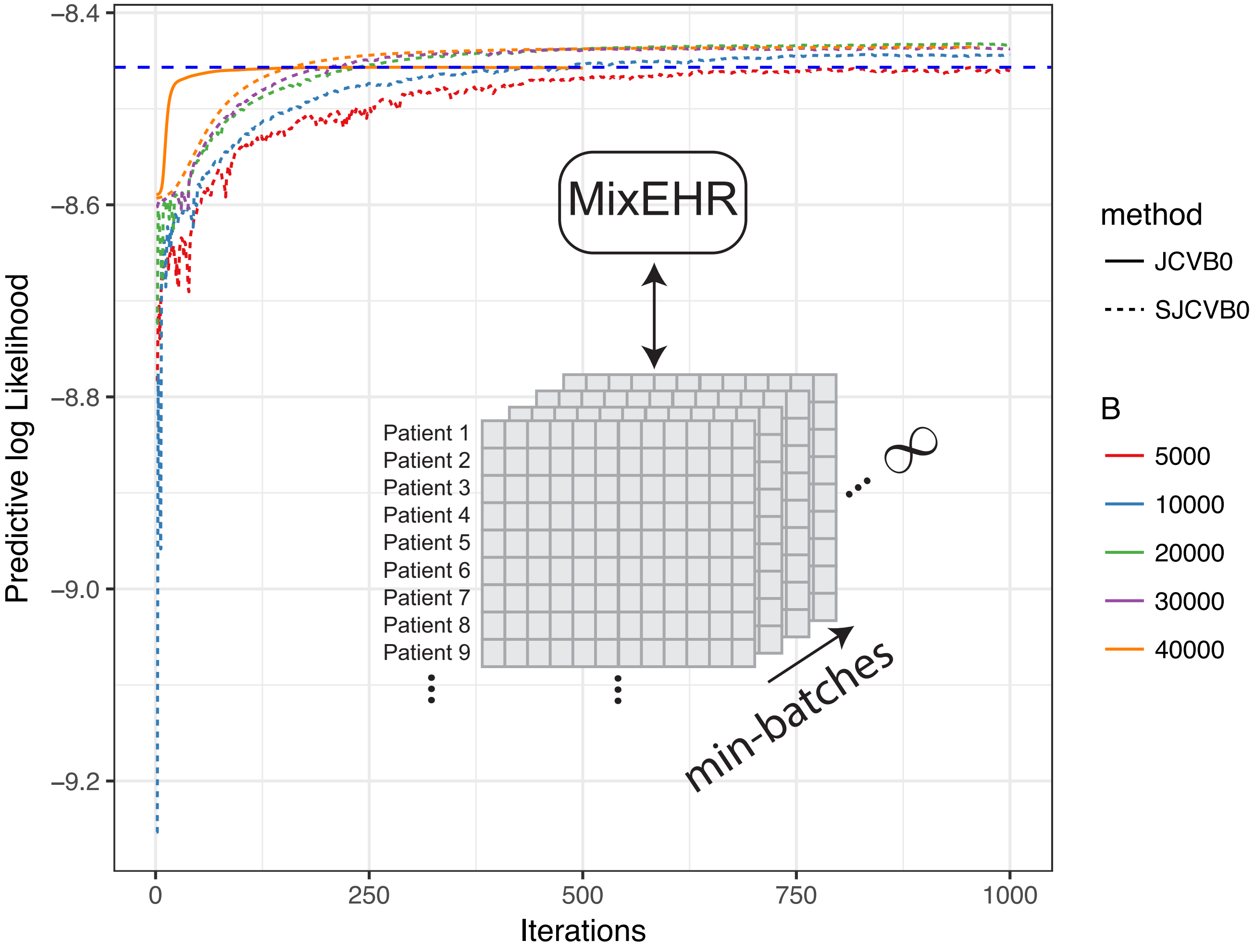

Figure 2: Stochastic achieve comparable performance as the full-batch learning with fraction of time and constant memory. The plot were generated using the MIMIC-III dataset.

Topic 1

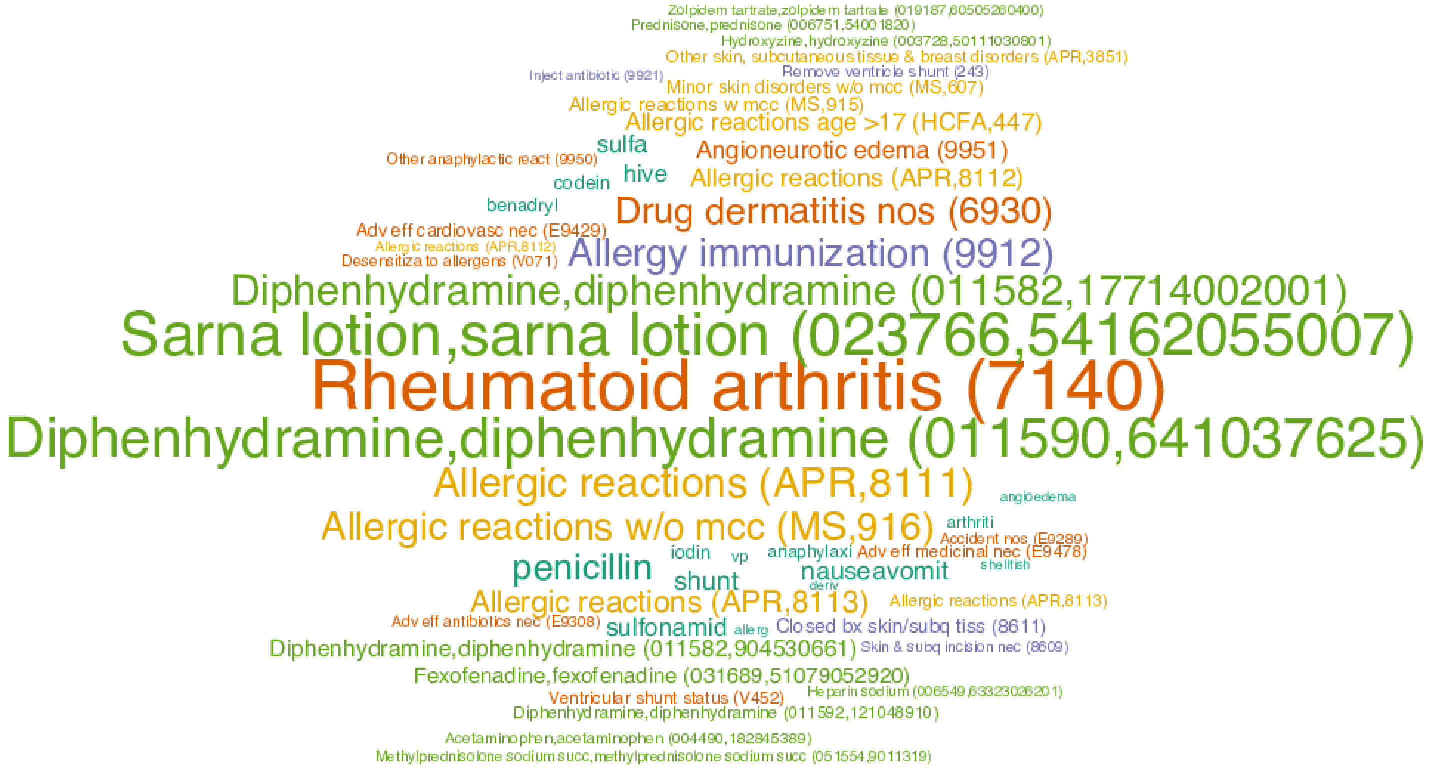

Zolpidem tartrate,zolpidem tartrate (019187,60505260400)
Prednisone,prednisone (006751,54001820)
Hydroxyzine,hydroxyzine (003728,50111030801)
Other skin, subcutaneous tissue & breast disorders (APR,3851)
Inject antibiotic (9921)
Remove ventricle shunt (243)
Minor skin disorders w/o mcc (MS,607)
Allergic reactions w mcc (MS,915)
Allergic reactions age >17 (HCFA,447)
sulfa
Other anaphylactic react (9950)
Angioneurotic edema (9951)
codein
hive
Allergic reactions (APR,8112)
benadryl
Drug dermatitis nos (6930)
Adv eff cardiovasc nec (E9429)
Allergic reactions (APR,8112)
Desensitiza to allergens (V071)
Allergy immunization (9912)
Diphenhydramine,diphenhydramine (011582,17714002001)
Sarna lotion,sarna lotion (023766,54162055007)
Rheumatoid arthritis (7140)
Diphenhydramine,diphenhydramine (011590,641037625)
Allergic reactions (APR,8111)
angioedema
Allergic reactions w/o mcc (MS,916)
arthriti
Accident nos (E9289)
penicillin
iodin
vp
anaphylaxi
Adv eff medicinal nec (E9478)
shellfish
shunt
nauseavomit
deriv
Allergic reactions (APR,8113)
Allergic reactions (APR,8113)
Adv eff antibiotics nec (E9308)
sulfonamid
allerg
Closed bx skin/subq tiss (8611)
Diphenhydramine,diphenhydramine (011582,904530661)
Skin & subq incision nec (8609)
Fexofenadine,fexofenadine (031689,51079052920)
Ventricular shunt status (V452)
Heparin sodium (006549,63323026201)
Diphenhydramine,diphenhydramine (011592,121048910)
Acetaminophen,acetaminophen (004490,182845389)
Methylprednisolone sodium succ,methylprednisolone sodium succ (051554,9011319)

Topic 2

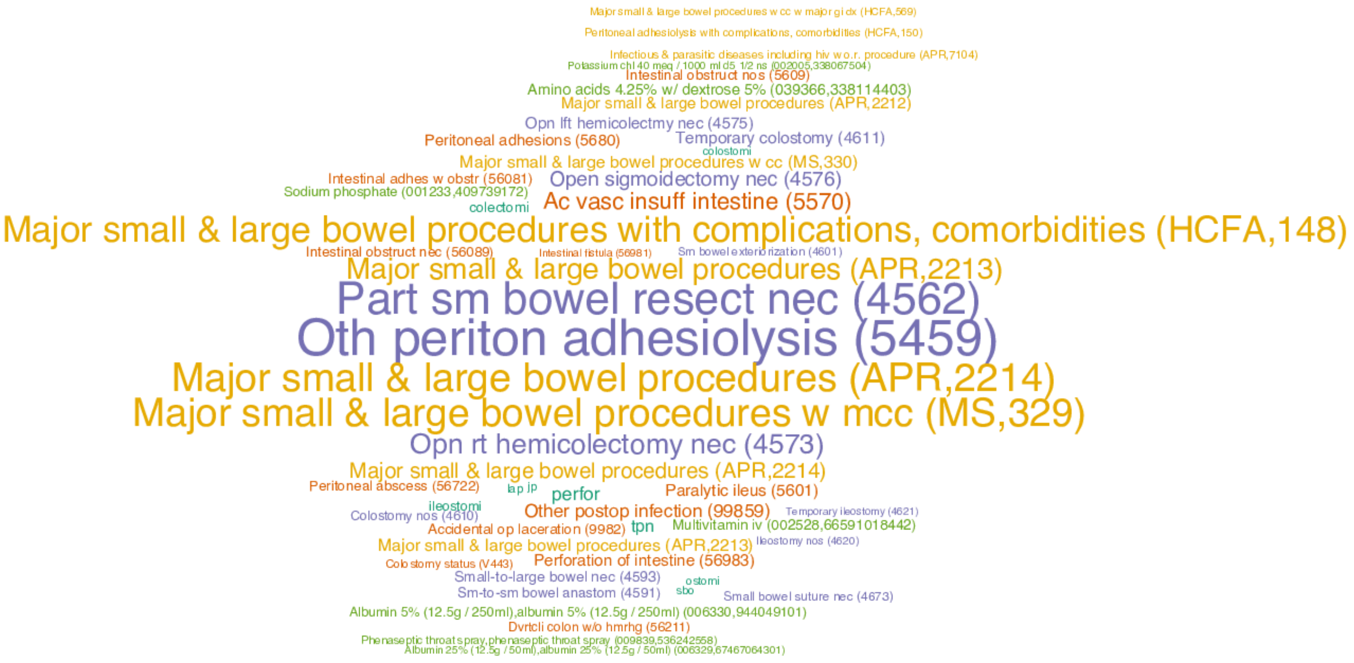
Major small & large bowel procedures w cc w major gi dx (HCFA,569)
Peritoneal adhesiolysis with complications, comorbidities (HCFA,150)
Infectious & parasitic diseases including hiv w o.r. procedure (APR,7104)
Potassium chl 40 meq / 1000 ml d5 1/2 ns (002005,338067504)
Intestinal obstruct nos (5609)
Amino acids 4.25% w/ dextrose 5% (039366,338114403)
Major small & large bowel procedures (APR,2212)
Opn lft hemicolectmy nec (4575)
Peritoneal adhesions (5680)
Temporary colostomy (4611)
colostomi
Major small & large bowel procedures w cc (MS,330)
Intestinal adhes w obstr (56081)
Open sigmoidectomy nec (4576)
Sodium phosphate (001233,409739172)
Ac vasc insuff intestine (5570)
colectomi
Major small & large bowel procedures with complications, comorbidities (HCFA,148)
Intestinal obstruct nec (56089)
Intestinal fistula (56981)
Sm bowel exteriorization (4601)
Major small & large bowel procedures (APR,2213)
Part sm bowel resect nec (4562)
Oth periton adhesiolysis (5459)
Major small & large bowel procedures (APR,2214)
Major small & large bowel procedures w mcc (MS,329)
Opn rt hemicolectomy nec (4573)
Major small & large bowel procedures (APR,2214)
Peritoneal abscess (56722)
lap jp
perfor
Paralytic ileus (5601)
ileostomi
Colostomy nos (4610)
Other postop infection (99859)
Temporary ileostomy (4621)
Accidental op laceration (9982)
tpn
Multivitamin iv (002528,66591018442)
Major small & large bowel procedures (APR,2213)
Ileostomy nos (4620)
Colostomy status (V443)
Perforation of intestine (56983)
Small-to-large bowel nec (4593)
ostomi
sbo
Sm-to-sm bowel anastom (4591)
Small bowel suture nec (4673)
Albumin 5% (12.5g / 250ml),albumin 5% (12.5g / 250ml) (006330,944049101)
Dvrtcli colon w/o hmrhg (56211)
Phenaseptic throat spray,phenaseptic throat spray (009839,536242558)
Albumin 25% (12.5g / 50ml),albumin 25% (12.5g / 50ml) (006329,67467064301)

Topic 3

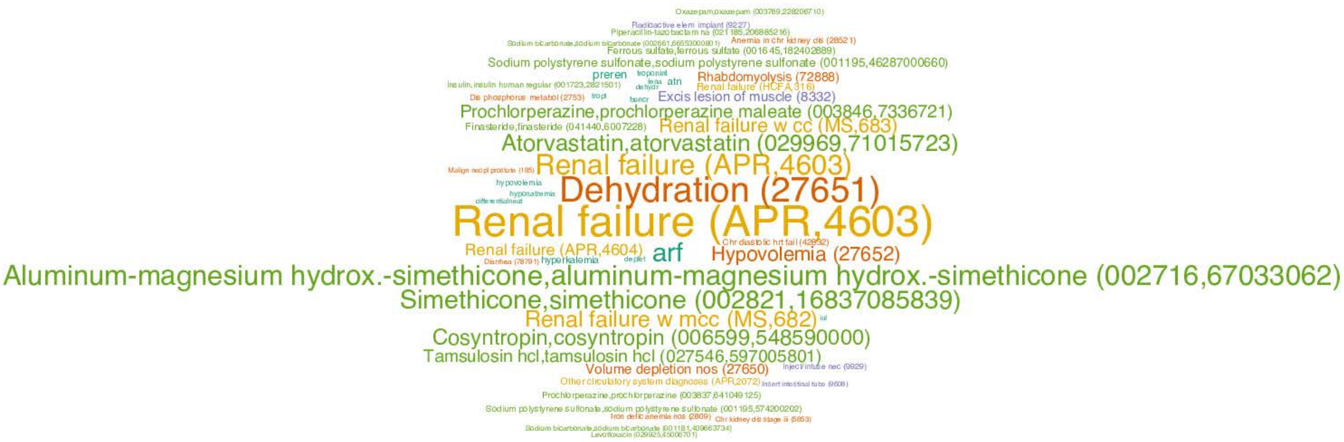
Oxazepam,oxazepam (003769,228206710)
Radioactive elem implant (9227)
Piperacillin-tazobactam na (021185,206885216)
Sodium bicarbonate,sodium bicarbonate (002661,6655300801)
Anemia in chr kidney dis (28521)
Ferrous sulfate,ferrous sulfate (001645,182402889)
Sodium polystyrene sulfonate,sodium polystyrene sulfonate (001195,46287000660)
preren
troponint
atn
Rhabdomyolysis (72888)
Insulin,insulin human regular (001723,2821501)
Renal failure (HCFA,316)
Dis phosphorus metabol (2753)
Excis lesion of muscle (8332)
Prochlorperazine,prochlorperazine maleate (003846,7336721)
Finasteride,finasteride (041440,6007228)
Renal failure w cc (MS,683)
Atorvastatin,atorvastatin (029969,71015723)
Malign neopl prostate (185)
Renal failure (APR,4603)
hypovolemia
hyponatremia
Dehydration (27651)
Renal failure (APR,4603)
Chr diastolic hrt fail (42832)
Renal failure (APR,4604)
arf
Hypovolemia (27652)
Diarrhea (78791)
hyperkalemia
Aluminum-magnesium hydrox.-simethicone,aluminum-magnesium hydrox.-simethicone (002716,67033062)
Simethicone,simethicone (002821,16837085839)
Renal failure w mcc (MS,682)
Cosyntropin,cosyntropin (006599,548590000)
Tamsulosin hcl,tamsulosin hcl (027546,597005801)
Volume depletion nos (27650)
Inject/infuse nec (9929)
Other circulatory system diagnoses (APR,2072)
Insert intestinal tube (9608)
Prochlorperazine,prochlorperazine (003837,641049125)
Sodium polystyrene sulfonate,sodium polystyrene sulfonate (001195,574200202)
Iron defic anemia nos (2809)
Chr kidney dis stage iii (5853)
Sodium bicarbonate,sodium bicarbonate (001181,409663734)
Levofloxacin (029925,45006701)

Topic 4

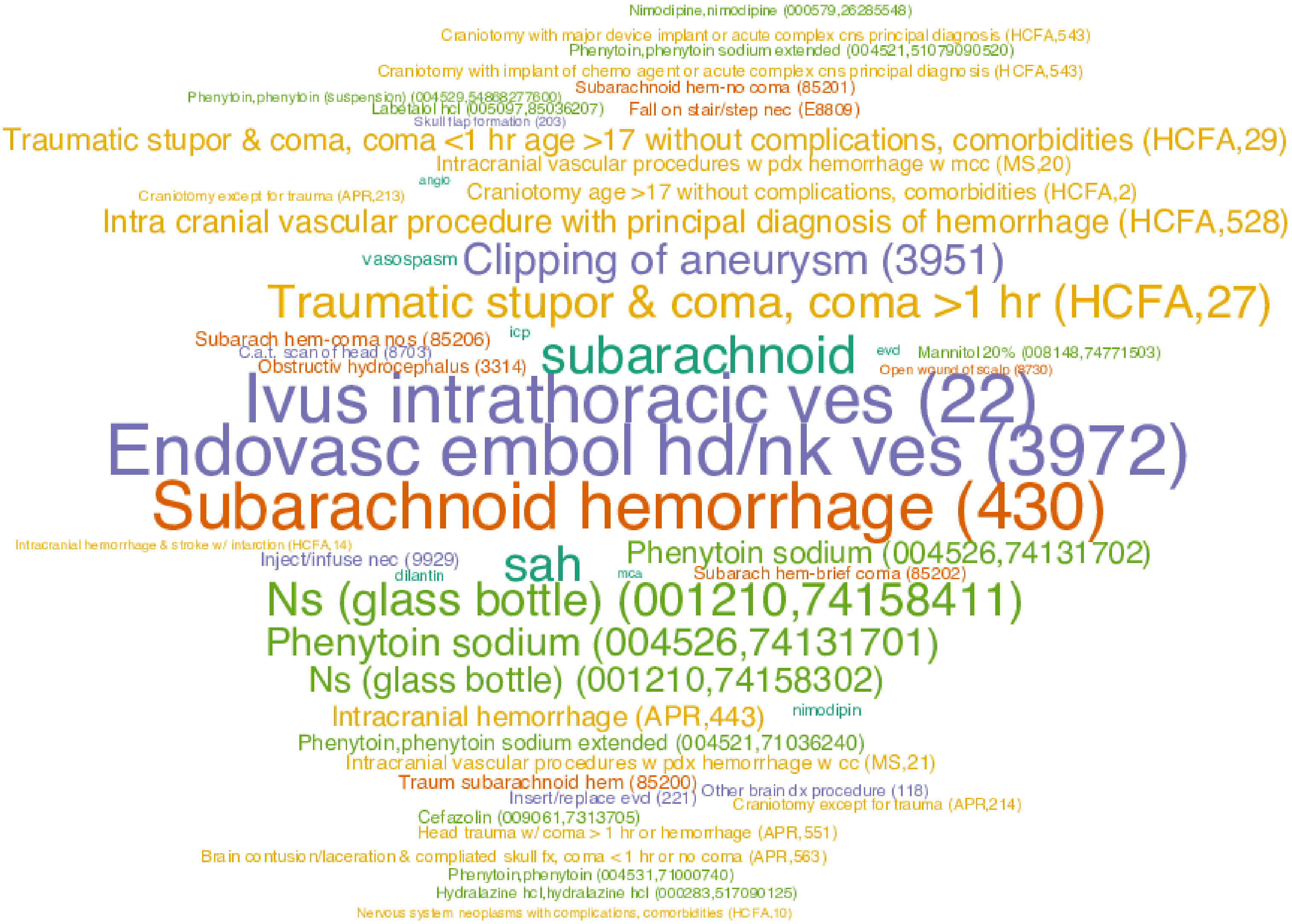

Nimodipine,nimodipine (000579,26285548)
Craniotomy with major device implant or acute complex cns principal diagnosis (HCFA,543)
Phenytoin,phenytoin sodium extended (004521,51079090520)
Craniotomy with implant of chemo agent or acute complex cns principal diagnosis (HCFA,543)
Subarachnoid hem-no coma (85201)
Phenytoin,phenytoin (suspension) (004529,54868277600)
Labetalol hcl (005097,85036207)
Fall on stair/step nec (E8809)
Skull flap formation (203)
Traumatic stupor & coma, coma <1 hr age >17 without complications, comorbidities (HCFA,29)
Intracranial vascular procedures w pdx hemorrhage w mcc (MS,20)
angio
Craniotomy except for trauma (APR,213)
Craniotomy age >17 without complications, comorbidities (HCFA,2)
Intra cranial vascular procedure with principal diagnosis of hemorrhage (HCFA,528)
vasospasm
Clipping of aneurysm (3951)
Traumatic stupor & coma, coma >1 hr (HCFA,27)
Subarach hem-coma nos (85206)
icp
C.a.t. scan of head (8703)
Obstructiv hydrocephalus (3314)
subarachnoid
evd
Mannitol 20% (008148,74771503)
Open wound of scalp (8730)
Ivus intrathoracic ves (22)
Endovasc embol hd/nk ves (3972)
Subarachnoid hemorrhage (430)
Intracranial hemorrhage & stroke w/ infarction (HCFA,14)
Inject/infuse nec (9929)
sah
Phenytoin sodium (004526,74131702)
dilantin
mca
Subarach hem-brief coma (85202)
Ns (glass bottle) (001210,74158411)
Phenytoin sodium (004526,74131701)
Ns (glass bottle) (001210,74158302)
Intracranial hemorrhage (APR,443)
nimodipin
Phenytoin,phenytoin sodium extended (004521,71036240)
Intracranial vascular procedures w pdx hemorrhage w cc (MS,21)
Traum subarachnoid hem (85200)
Other brain dx procedure (118)
Insert/replace evd (221)
Craniotomy except for trauma (APR,214)
Cefazolin (009061,7313705)
Head trauma w/ coma > 1 hr or hemorrhage (APR,551)
Brain contusion/laceration & compliated skull fx, coma < 1 hr or no coma (APR,563)
Phenytoin,phenytoin (004531,71000740)
Hydralazine hcl,hydralazine hcl (000283,517090125)
Nervous system neoplasms with complications, comorbidities (HCFA,10)

Topic 5

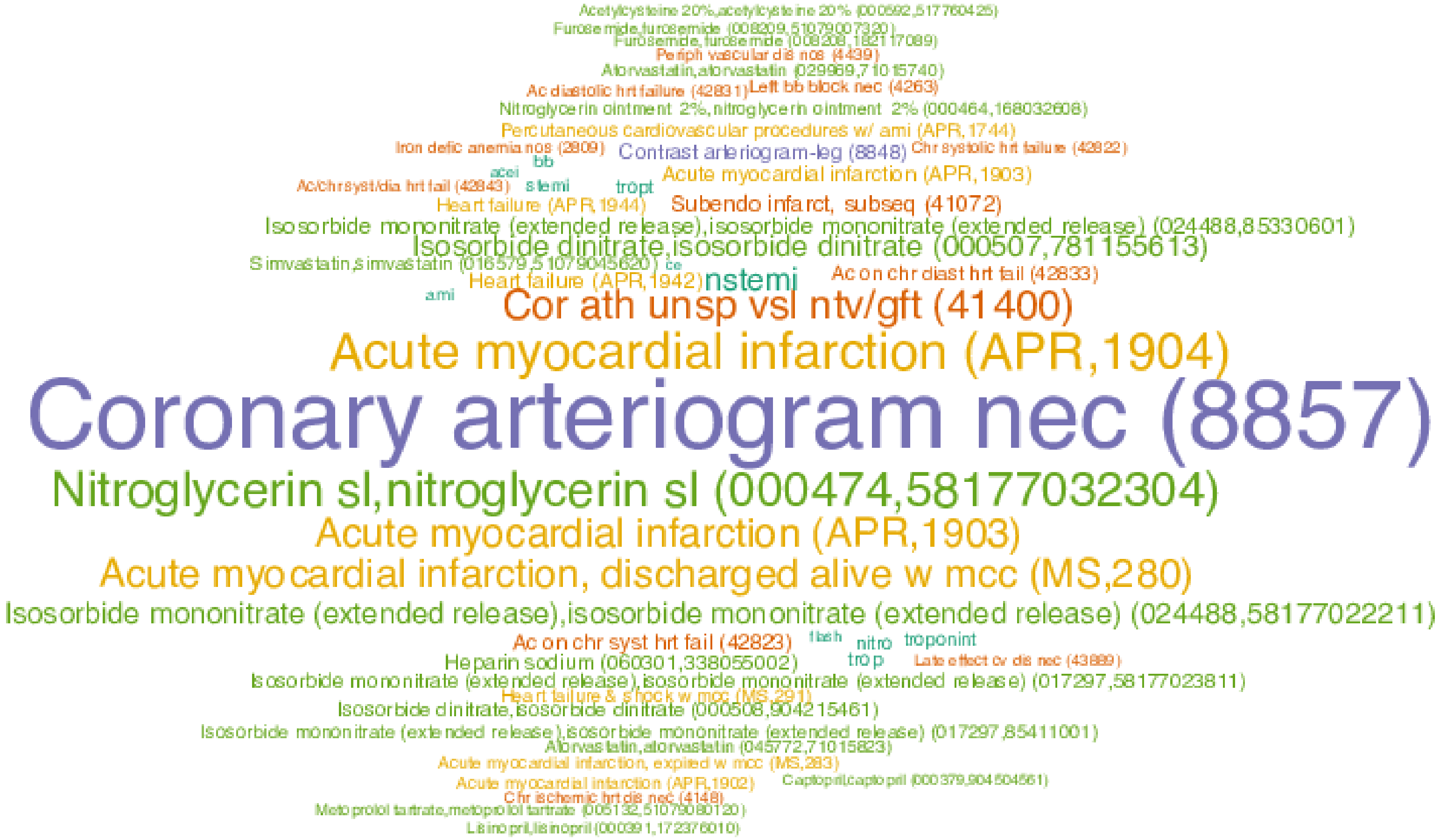
Acetylcysteine 20%,acetylcysteine 20% (000592,517760425)
Furosemide,furosemide (008209,51079007320)
Furosemide,furosemide (008208,182117089)
Periph vascular dis nos (4439)
Atorvastatin,atorvastatin (029969,71015740)
Ac diastolic hrt failure (42831)
Left bb block nec (4263)
Nitroglycerin ointment 2%,nitroglycerin ointment 2% (000464,168032608)
Percutaneous cardiovascular procedures w/ ami (APR,1744)
Iron defic anemia nos (2809)
Contrast arteriogram-leg (8848)
Chr systolic hrt failure (42822)
bb
acei
Acute myocardial infarction (APR,1903)
Ac/chr syst/dia hrt fail (42843)
stemi
tropt
Heart failure (APR,1944)
Subendo infarct, subseq (41072)
Isosorbide mononitrate (extended release),isosorbide mononitrate (extended release) (024488,85330601)
Isosorbide dinitrate,isosorbide dinitrate (000507,781155613)
Simvastatin,simvastatin (016579,51079045620)
ce
Heart failure (APR,1942)
nstemi
Ac on chr diast hrt fail (42833)
ami
Cor ath unsp vsl ntv/gft (41400)
Acute myocardial infarction (APR,1904)
Coronary arteriogram nec (8857)
Nitroglycerin sl,nitroglycerin sl (000474,58177032304)
Acute myocardial infarction (APR,1903)
Acute myocardial infarction, discharged alive w mcc (MS,280)
Isosorbide mononitrate (extended release),isosorbide mononitrate (extended release) (024488,58177022211)
Ac on chr syst hrt fail (42823)
flash
nitro
troponint
Heparin sodium (060301,338055002)
trop
Late effect cv dis nec (43889)
Isosorbide mononitrate (extended release),isosorbide mononitrate (extended release) (017297,58177023811)
Heart failure & shock w mcc (MS,291)
Isosorbide dinitrate,isosorbide dinitrate (000508,904215461)
Isosorbide mononitrate (extended release),isosorbide mononitrate (extended release) (017297,85411001)
Atorvastatin,atorvastatin (045772,71015823)
Acute myocardial infarction, expired w mcc (MS,283)
Acute myocardial infarction (APR,1902)
Captopril,captopril (000379,904504561)
Chr ischemic hrt dis nec (4148)
Metoprolol tartrate,metoprolol tartrate (005132,51079080120)
Lisinopril,lisinopril (000391,172376010)

Topic 6

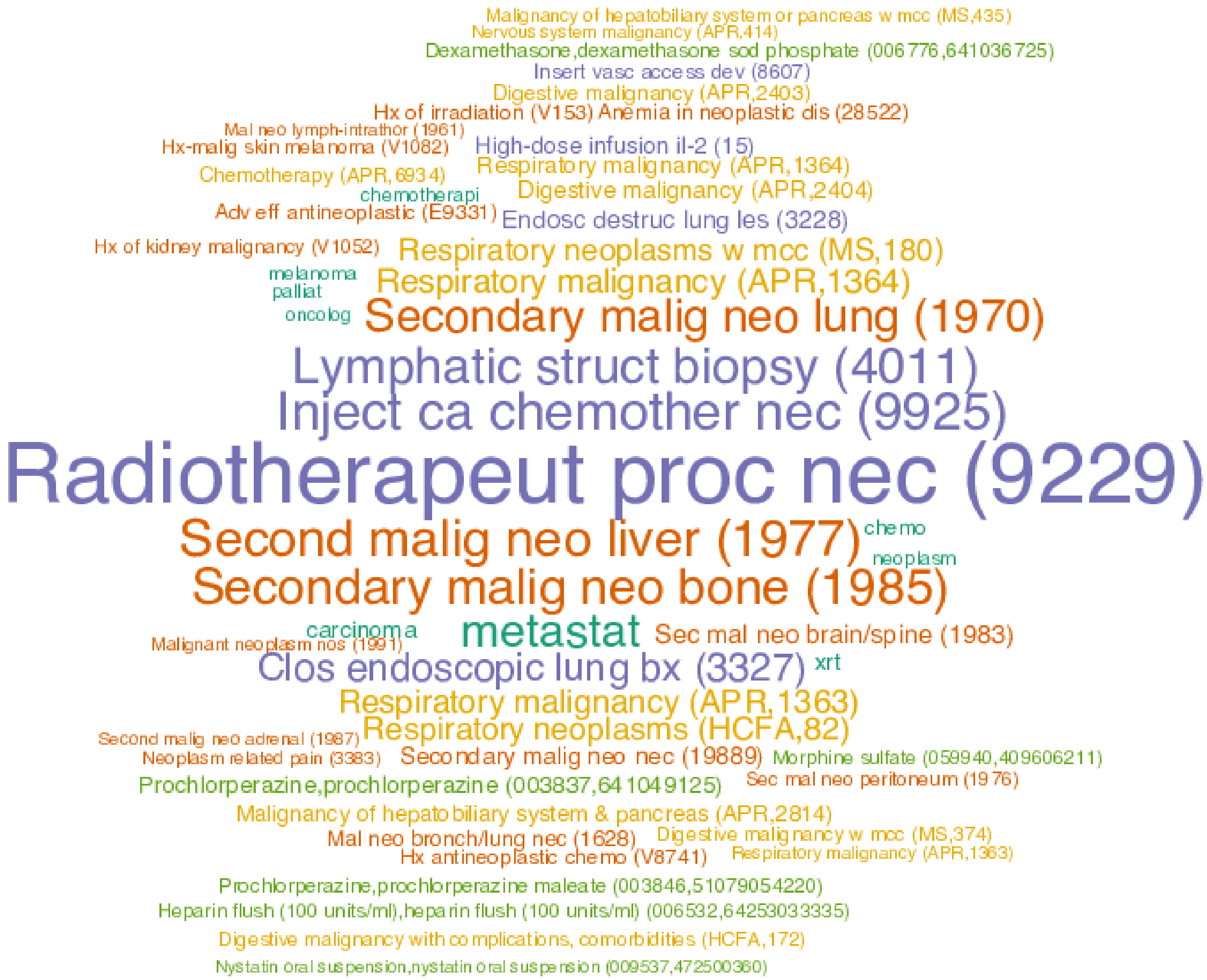

Malignancy of hepatobiliary system or pancreas w mcc (MS,435)
Nervous system malignancy (APR,414)
Dexamethasone,dexamethasone sod phosphate (006776,641036725)
Insert vasc access dev (8607)
Digestive malignancy (APR,2403)
Hx of irradiation (V153)
Anemia in neoplastic dis (28522)
Mal neo lymph-intrathor (1961)
Hx-malig skin melanoma (V1082)
High-dose infusion il-2 (15)
Respiratory malignancy (APR,1364)
Chemotherapy (APR,6934)
chemotherapi
Digestive malignancy (APR,2404)
Adv eff antineoplastic (E9331)
Endosc destruc lung les (3228)
Hx of kidney malignancy (V1052)
Respiratory neoplasms w mcc (MS,180)
melanoma
palliat
Respiratory malignancy (APR,1364)
oncolog
Secondary malig neo lung (1970)
Lymphatic struct biopsy (4011)
Inject ca chemother nec (9925)
Radiotherapeut proc nec (9229)
Second malig neo liver (1977)
chemo
neoplasm
Secondary malig neo bone (1985)
carcinoma
metastat
Sec mal neo brain/spine (1983)
Malignant neoplasm nos (1991)
Clos endoscopic lung bx (3327)
xrt
Respiratory malignancy (APR,1363)
Respiratory neoplasms (HCFA,82)
Second malig neo adrenal (1987)
Neoplasm related pain (3383)
Secondary malig neo nec (19889)
Morphine sulfate (059940,409606211)
Prochlorperazine,prochlorperazine (003837,641049125)
Sec mal neo peritoneum (1976)
Malignancy of hepatobiliary system & pancreas (APR,2814)
Mal neo bronch/lung nec (1628)
Digestive malignancy w mcc (MS,374)
Hx antineoplastic chemo (V8741)
Respiratory malignancy (APR,1363)
Prochlorperazine,prochlorperazine maleate (003846,51079054220)
Heparin flush (100 units/ml),heparin flush (100 units/ml) (006532,64253033335)
Digestive malignancy with complications, comorbidities (HCFA,172)
Nystatin oral suspension,nystatin oral suspension (009537,472500360)

Topic 7

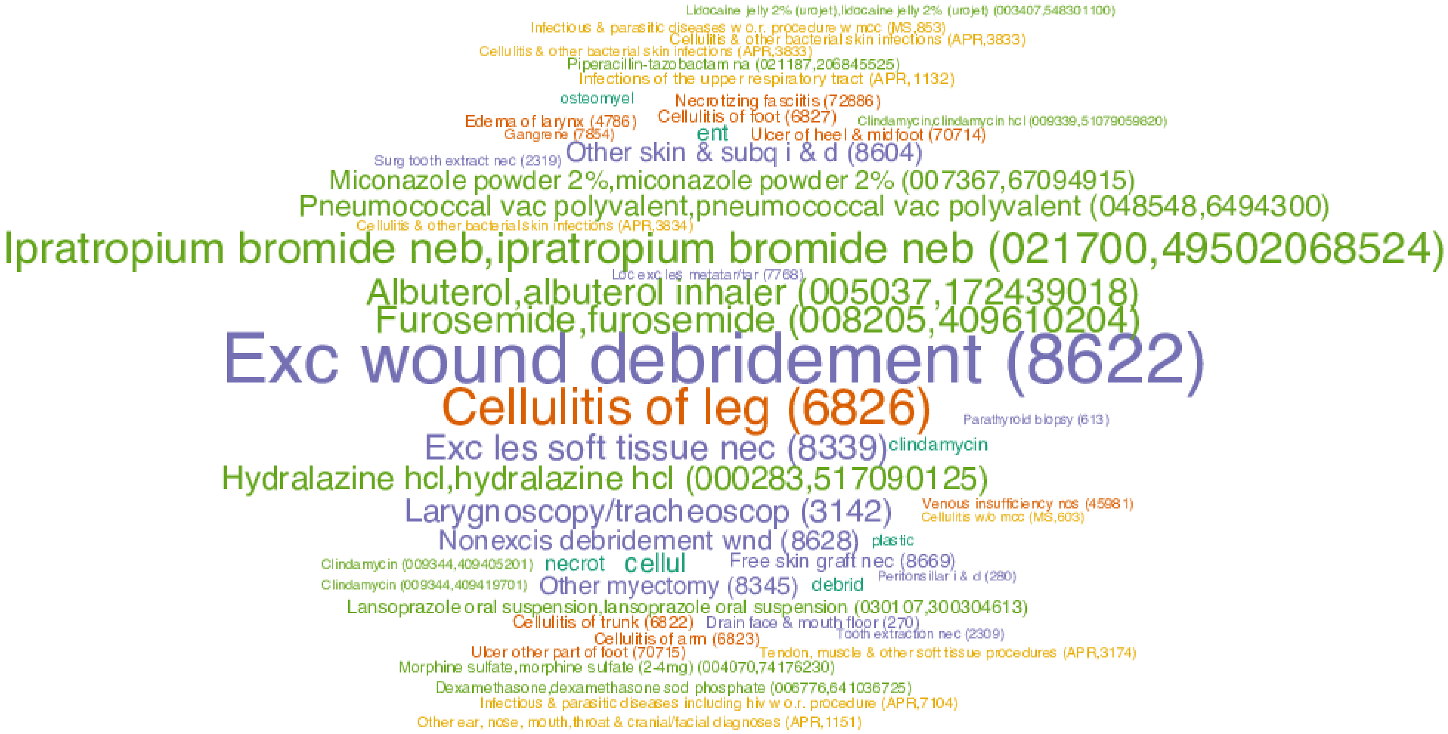
Lidocaine jelly 2% (urojet),lidocaine jelly 2% (urojet) (003407,548301100)
Infectious & parasitic diseases w o.r. procedure w mcc (MS,853)
Cellulitis & other bacterial skin infections (APR,3833)
Cellulitis & other bacterial skin infections (APR,3833)
Piperacillin-tazobactam na (021187,206845525)
Infections of the upper respiratory tract (APR,1132)
osteomyel
Necrotizing fasciitis (72886)
Edema of larynx (4786)
Cellulitis of foot (6827)
Clindamycin,clindamycin hcl (009339,51079059820)
Gangrene (7854)
ent
Ulcer of heel & midfoot (70714)
Surg tooth extract nec (2319)
Other skin & subq i & d (8604)
Miconazole powder 2%,miconazole powder 2% (007367,67094915)
Pneumococcal vac polyvalent,pneumococcal vac polyvalent (048548,6494300)
Cellulitis & other bacterial skin infections (APR,3834)
Ipratropium bromide neb,ipratropium bromide neb (021700,49502068524)
Loc exc les metatar/tar (7768)
Albuterol,albuterol inhaler (005037,172439018)
Furosemide,furosemide (008205,409610204)
Exc wound debridement (8622)
Cellulitis of leg (6826)
Parathyroid biopsy (613)
Exc les soft tissue nec (8339)
clindamycin
Hydralazine hcl,hydralazine hcl (000283,517090125)
Larygnoscopy/tracheoscop (3142)
Venous insufficiency nos (45981)
Cellulitis w/o mcc (MS,603)
Nonexcis debridement wnd (8628)
plastic
Clindamycin (009344,409405201)
necrot
cellul
Free skin graft nec (8669)
Clindamycin (009344,409419701)
Other myectomy (8345)
debrid
Peritonsillar i & d (280)
Lansoprazole oral suspension,lansoprazole oral suspension (030107,300304613)
Cellulitis of trunk (6822)
Drain face & mouth floor (270)
Tooth extraction nec (2309)
Cellulitis of arm (6823)
Ulcer other part of foot (70715)
Tendon, muscle & other soft tissue procedures (APR,3174)
Morphine sulfate,morphine sulfate (2-4mg) (004070,74176230)
Dexamethasone,dexamethasone sod phosphate (006776,641036725)
Infectious & parasitic diseases including hiv w o.r. procedure (APR,7104)
Other ear, nose, mouth,throat & cranial/facial diagnoses (APR,1151)

Topic 8

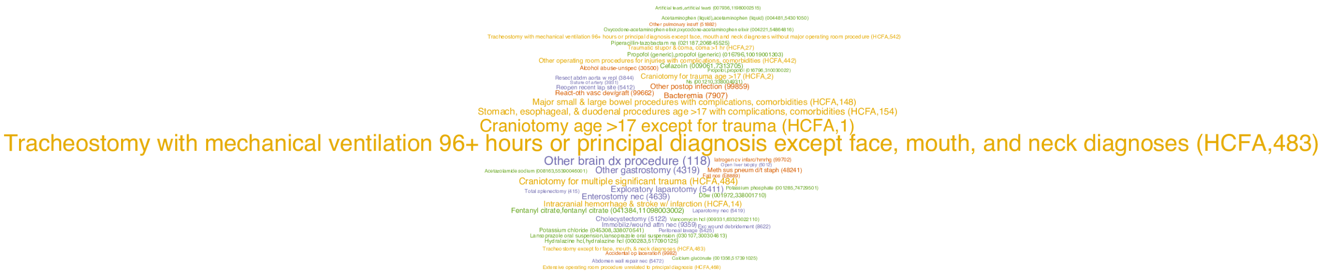
Artificial tears,artificial tears (007936,11980002515)
Acetaminophen (liquid),acetaminophen (liquid) (004481,54301050)
Other pulmonary insuff (51882)
Oxycodone-acetaminophen elixir,oxycodone-acetaminophen elixir (004221,54864816)
Tracheostomy with mechanical ventilation 96+ hours or principal diagnosis except face, mouth and neck diagnoses without major operating room procedure (HCFA,542)
Piperacillin-tazobactam na (021187,206845525)
Traumatic stupor & coma, coma >1 hr (HCFA,27)
Propofol (generic),propofol (generic) (016796,10019001303)
Other operating room procedures for injuries with complications, comorbidities (HCFA,442)
Alcohol abuse-unspec (30500)
Cefazolin (009061,7313705)
Propofol,propofol (016796,310030022)
Resect abdm aorta w repl (3844)
Craniotomy for trauma age >17 (HCFA,2)
Suture of artery (3931)
Ns (001210,338004931)
Reopen recent lap site (5412)
Other postop infection (99859)
React-oth vasc dev/graft (99662)
Bacteremia (7907)
Major small & large bowel procedures with complications, comorbidities (HCFA,148)
Stomach, esophageal, & duodenal procedures age >17 with complications, comorbidities (HCFA,154)
Craniotomy age >17 except for trauma (HCFA,1)
Tracheostomy with mechanical ventilation 96+ hours or principal diagnosis except face, mouth, and neck diagnoses (HCFA,483)
Other brain dx procedure (118)
Iatrogen cv infarc/hmrhg (99702)
Open liver biopsy (5012)
Acetazolamide sodium (008163,55390046001)
Other gastrostomy (4319)
Meth sus pneum d/t staph (48241)
Fall nos (E8889)
Craniotomy for multiple significant trauma (HCFA,484)
Total splenectomy (415)
Exploratory laparotomy (5411)
Potassium phosphate (001285,74729501)
Enterostomy nec (4639)
D5w (001972,338001710)
Intracranial hemorrhage & stroke w/ infarction (HCFA,14)
Fentanyl citrate,fentanyl citrate (041384,11098003002)
Laparotomy nec (5419)
Cholecystectomy (5122)
Vancomycin hcl (009331,63323022110)
Immobiliz/wound attn nec (9359)
Exc wound debridement (8622)
Potassium chloride (045308,338070541)
Peritoneal lavage (5425)
Lansoprazole oral suspension,lansoprazole oral suspension (030107,300304613)
Hydralazine hcl,hydralazine hcl (000283,517090125)
Tracheostomy except for face, mouth, & neck diagnoses (HCFA,483)
Accidental op laceration (9982)
Calcium gluconate (001356,517391025)
Abdomen wall repair nec (5472)
Extensive operating room procedure unrelated to principal diagnosis (HCFA,468)

Topic 9

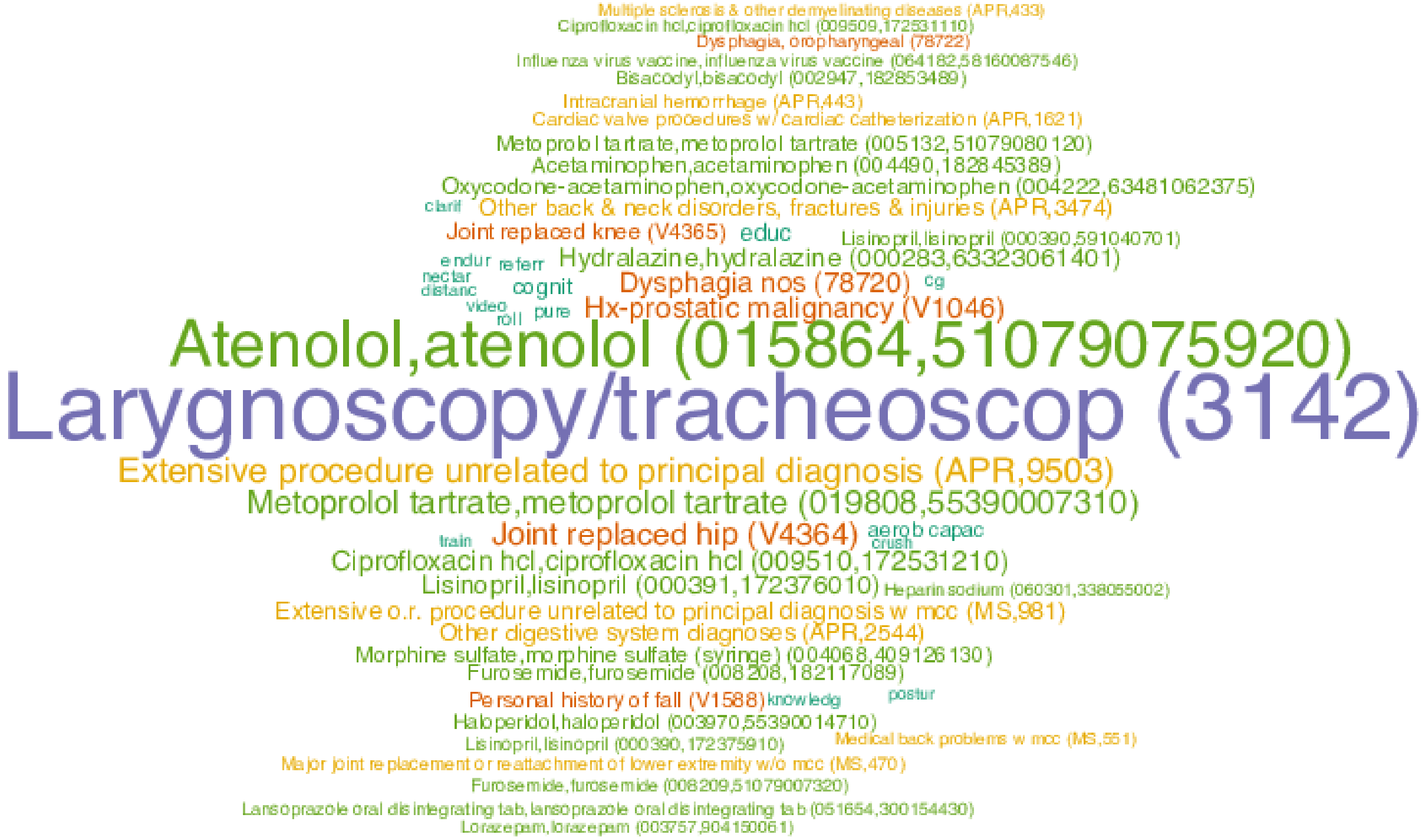

Multiple sclerosis & other demyelinating diseases (APR,433)
Ciprofloxacin hcl,ciprofloxacin hcl (009509,172531110)
Dysphagia, oropharyngeal (78722)
Influenza virus vaccine,influenza virus vaccine (064182,58160087546)
Bisacodyl,bisacodyl (002947,182853489)
Intracranial hemorrhage (APR,443)
Cardiac valve procedures w/ cardiac catheterization (APR,1621)
Metoprolol tartrate,metoprolol tartrate (005132,51079080120)
Acetaminophen,acetaminophen (004490,182845389)
Oxycodone-acetaminophen,oxycodone-acetaminophen (004222,63481062375)
clarif
Other back & neck disorders, fractures & injuries (APR,3474)
Joint replaced knee (V4365)
educ
Lisinopril,lisinopril (000390,591040701)
endur
referr
Hydralazine,hydralazine (000283,63323061401)
nectar
distanc
cognit
Dysphagia nos (78720)
cg
video
roll
pure
Hx-prostatic malignancy (V1046)
Atenolol,atenolol (015864,51079075920)
Larygnoscopy/tracheoscop (3142)
Extensive procedure unrelated to principal diagnosis (APR,9503)
Metoprolol tartrate,metoprolol tartrate (019808,55390007310)
train
Joint replaced hip (V4364)
aerob capac
crush
Ciprofloxacin hcl,ciprofloxacin hcl (009510,172531210)
Lisinopril,lisinopril (000391,172376010)
Heparin sodium (060301,338055002)
Extensive o.r. procedure unrelated to principal diagnosis w mcc (MS,981)
Other digestive system diagnoses (APR,2544)
Morphine sulfate,morphine sulfate (syringe) (004068,409126130)
Furosemide,furosemide (008208,182117089)
Personal history of fall (V1588)
knowledg
postur
Haloperidol,haloperidol (003970,55390014710)
Lisinopril,lisinopril (000390,172375910)
Medical back problems w mcc (MS,551)
Major joint replacement or reattachment of lower extremity w/o mcc (MS,470)
Furosemide,furosemide (008209,51079007320)
Lansoprazole oral disintegrating tab,lansoprazole oral disintegrating tab (051654,300154430)
Lorazepam,lorazepam (003757,904150061)

Topic 10

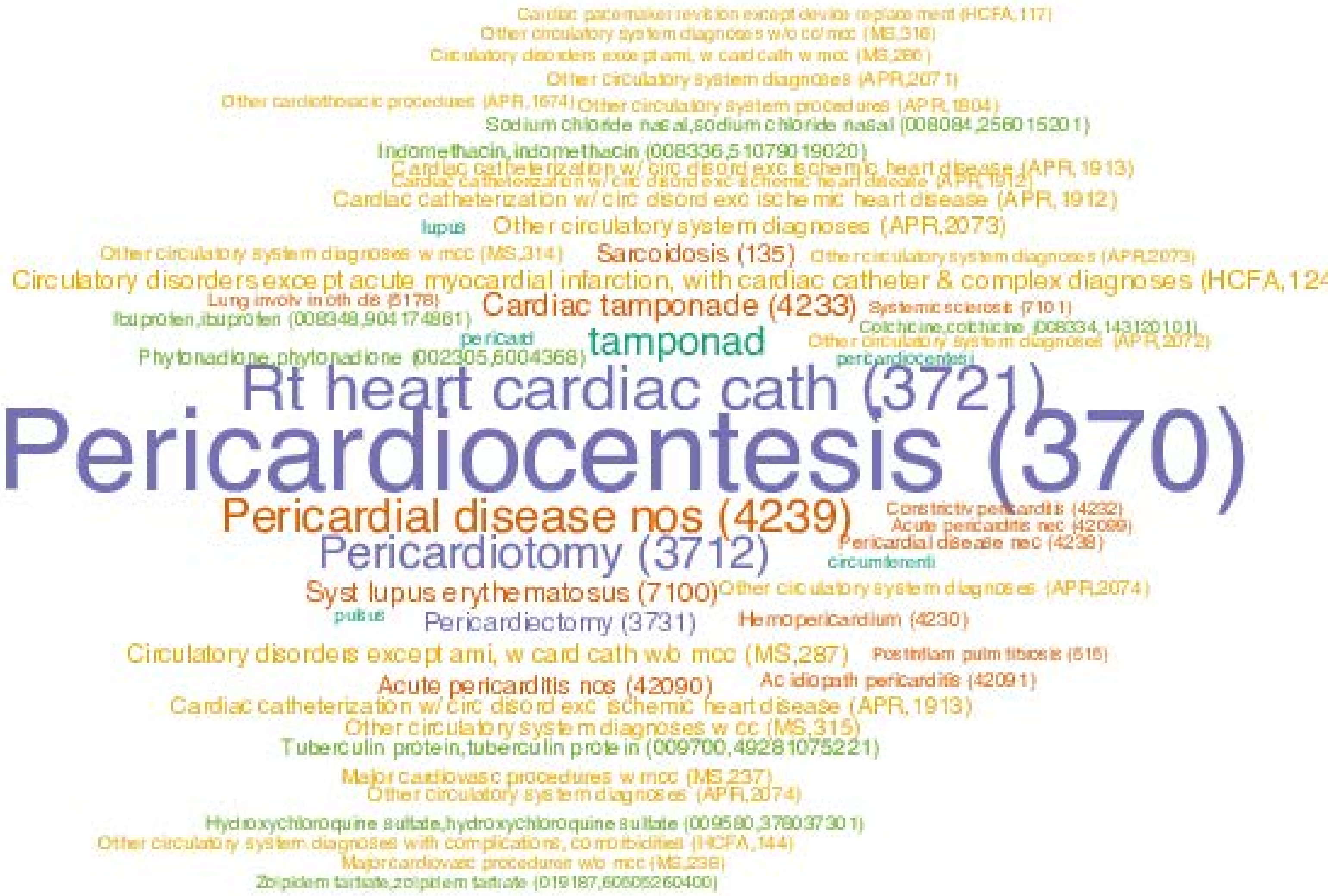
Pericardiocentesis (370)
Rt heart cardiac cath (3721)
Pericardial disease nos (4239)
Pericardiotomy (3712)
tamponad
Cardiac tamponade (4233)
Circulatory disorders except acute myocardial infarction, with cardiac catheter & complex diagnoses (HCFA,124
Sarcoidosis (135)
Other circulatory system diagnoses (APR,2073)
Cardiac catheterization w/ circ disord exc ischemic heart disease (APR,1912)
Syst lupus erythematosus (7100)
Pericardiectomy (3731)
Circulatory disorders except ami, w card cath w/o mcc (MS,287)
Acute pericarditis nos (42090)
Cardiac catheterization w/ circ disord exc ischemic heart disease (APR,1913)
Other circulatory system diagnoses w cc (MS,315)
Tuberculin protein,tuberculin protein (009700,49281075221)
Hemopericardium (4230)
Other circulatory system diagnoses (APR,2074)
Indomethacin,indomethacin (008336,51079019020)
Sodium chloride nasal,sodium chloride nasal (008084,25601520 1)
Phytonadione,phytonadione (002305,6004368)

Topic 11

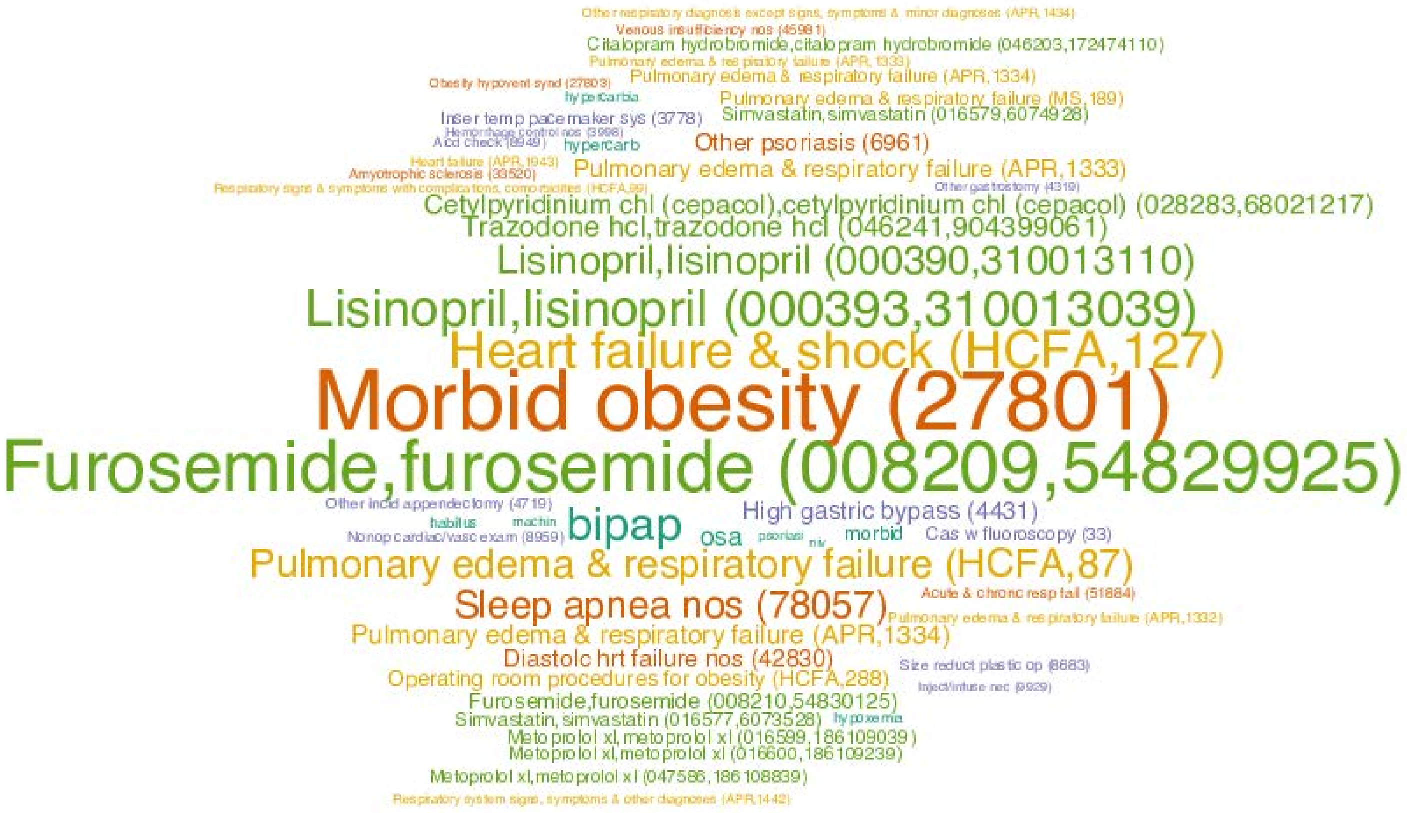
Other respiratory diagnosis except signs, symptoms & minor diagnoses (APR,1434)
Venous insufficiency nos (45981)
Citalopram hydrobromide,citalopram hydrobromide (046203,172474110)
Pulmonary edema & respiratory failure (APR,1333)
Pulmonary edema & respiratory failure (APR,1334)
Obesity hypovent synd (27803)
hypercarbia
Pulmonary edema & respiratory failure (MS,189)
Inser temp pacemaker sys (3778)
Simvastatin,simvastatin (016579,60749 28)
Hemorrhage control nos (3998)
Acd check (8949)
hypercarb
Other psoriasis (6961)
Heart failure (APR,1943)
Amyotrophic sclerosis (33520)
Pulmonary edema & respiratory failure (APR,1333)
Respiratory signs & symptoms with complications, comorbidities (HCFA,99)
Other gastrostomy (4319)
Cetylpyridinium chl (cepacol),cetylpyridinium chl (cepacol) (028283,68021217)
Trazodone hcl,trazodone hcl (046241,904399061)
Lisinopril,lisinopril (000390,310013110)
Lisinopril,lisinopril (000393,310013039)
Heart failure & shock (HCFA,127)
Morbid obesity (27801)
Furosemide,furosemide (008209,54829925)
Other incid appendectomy (4719)
High gastric bypass (4431)
habitus
machin
bipap
osa
Nonop cardiac/vasc exam (8959)
psoriasi
niv
morbid
Cas w fluoroscopy (33)
Pulmonary edema & respiratory failure (HCFA,87)
Acute & chronc resp fail (51884)
Sleep apnea nos (78057)
Pulmonary edema & respiratory failure (APR,1332)
Pulmonary edema & respiratory failure (APR,1334)
Diastolc hrt failure nos (42830)
Size reduct plastic op (8683)
Operating room procedures for obesity (HCFA,288)
Inject/infuse nec (9929)
Furosemide,furosemide (008210,54830125)
Simvastatin,simvastatin (016577,6073528)
hypoxemia
Metoprolol xl,metoprolol xl (016599,186109039)
Metoprolol xl,metoprolol xl (016600,186109239)
Metoprolol xl,metoprolol xl (047586,186108839)
Respiratory system signs, symptoms & other diagnoses (APR,1442)

Topic 12

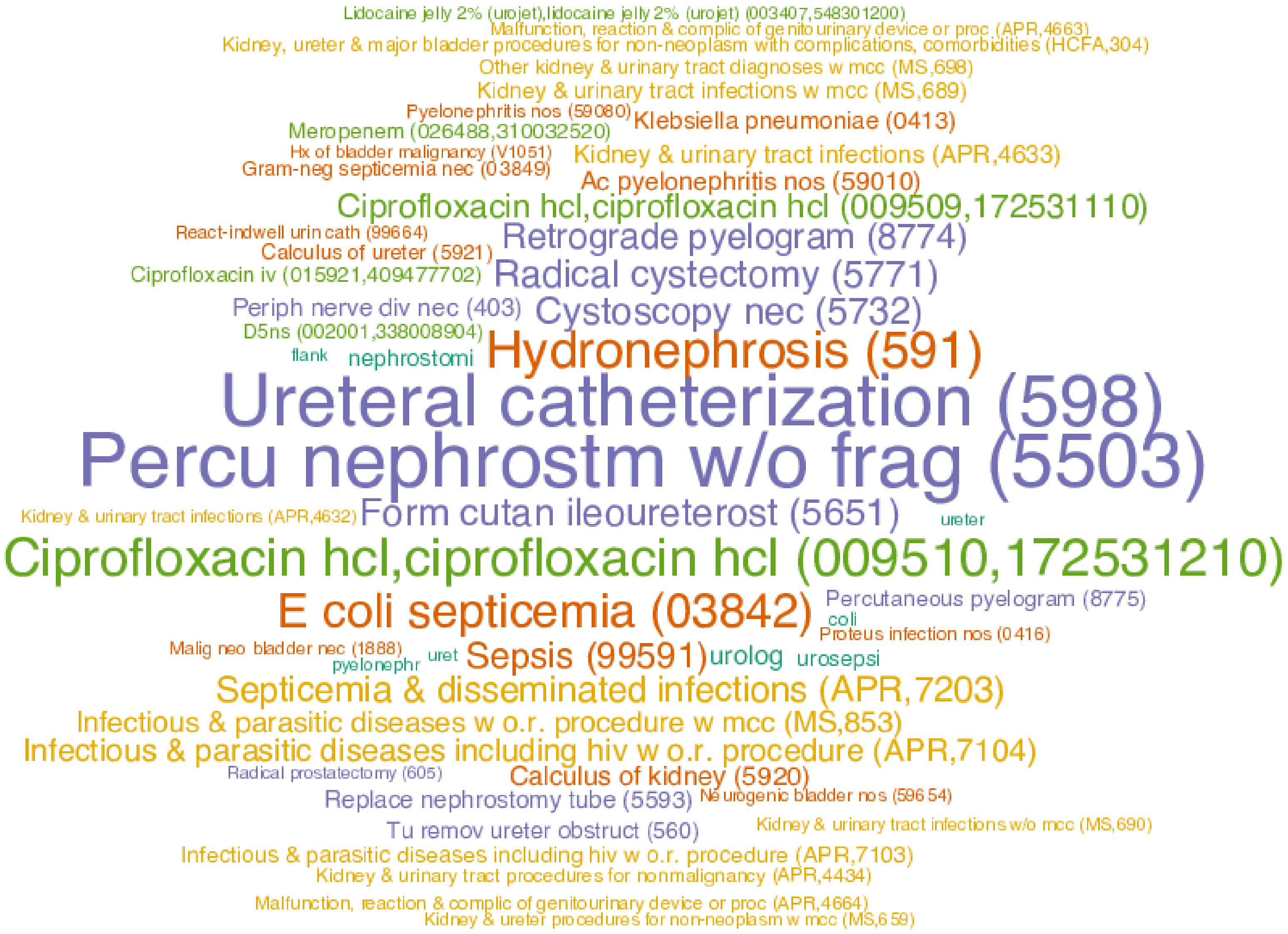
Lidocaine jelly 2% (urojet),lidocaine jelly 2% (urojet) (003407,548301200)
Malfunction, reaction & complic of genitourinary device or proc (APR,4663)
Kidney, ureter & major bladder procedures for non-neoplasm with complications, comorbidities (HCFA,304)
Other kidney & urinary tract diagnoses w mcc (MS,698)
Kidney & urinary tract infections w mcc (MS,689)
Pyelonephritis nos (59080)
Klebsiella pneumoniae (0413)
Meropenem (026488,310032520)
Hx of bladder malignancy (V1051)
Kidney & urinary tract infections (APR,4633)
Gram-neg septicemia nec (03849)
Ac pyelonephritis nos (59010)
Ciprofloxacin hcl,ciprofloxacin hcl (009509,172531110)
React-indwell urin cath (99664)
Retrograde pyelogram (8774)
Calculus of ureter (5921)
Ciprofloxacin iv (015921,409477702)
Radical cystectomy (5771)
Periph nerve div nec (403)
Cystoscopy nec (5732)
D5ns (002001,338008904)
flank
nephrostomi
Hydronephrosis (591)
Ureteral catheterization (598)
Percu nephrostm w/o frag (5503)
Kidney & urinary tract infections (APR,4632)
Form cutan ileoureterost (5651)
ureter
Ciprofloxacin hcl,ciprofloxacin hcl (009510,172531210)
E coli septicemia (03842)
Percutaneous pyelogram (8775)
coli
Proteus infection nos (0416)
Malig neo bladder nec (1888)
uret
pyelonephr
Sepsis (99591)
urolog
urosepsi
Septicemia & disseminated infections (APR,7203)
Infectious & parasitic diseases w o.r. procedure w mcc (MS,853)
Infectious & parasitic diseases including hiv w o.r. procedure (APR,7104)
Radical prostatectomy (605)
Calculus of kidney (5920)
Replace nephrostomy tube (5593)
Neurogenic bladder nos (59654)
Kidney & urinary tract infections w/o mcc (MS,690)
Tu remov ureter obstruct (560)
Infectious & parasitic diseases including hiv w o.r. procedure (APR,7103)
Kidney & urinary tract procedures for nonmalignancy (APR,4434)
Malfunction, reaction & complic of genitourinary device or proc (APR,4664)
Kidney & ureter procedures for non-neoplasm w mcc (MS,659)

Topic 13

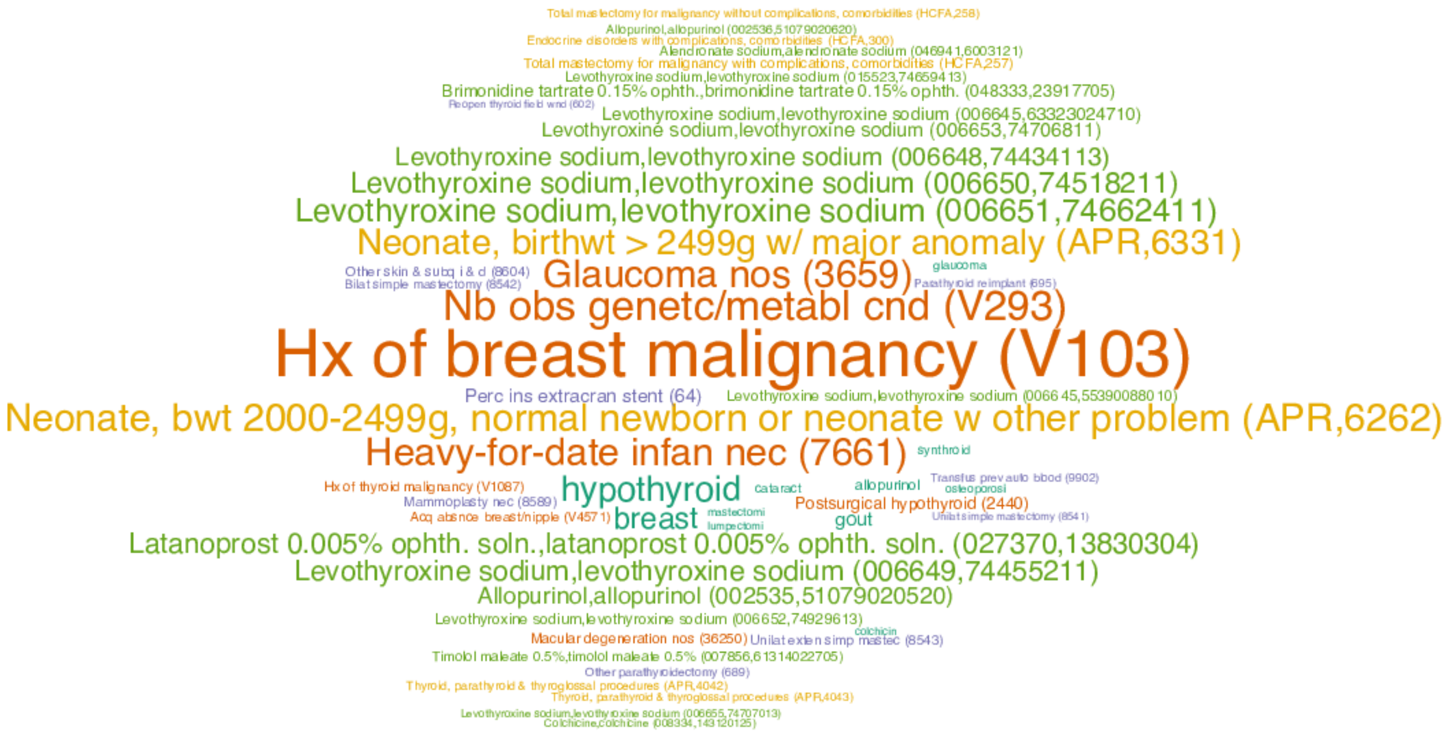

Total mastectomy for malignancy without complications, comorbidities (HCFA,258)
Allopurinol,allopurinol (002536,51079020620)
Endocrine disorders with complications, comorbidities (HCFA,300)
Alendronate sodium,alendronate sodium (046941,6003121)
Total mastectomy for malignancy with complications, comorbidities (HCFA,257)
Levothyroxine sodium,levothyroxine sodium (015523,74659413)
Brimonidine tartrate 0.15% ophth.,brimonidine tartrate 0.15% ophth. (048333,23917705)
Reopen thyroid field wnd (602)
Levothyroxine sodium,levothyroxine sodium (006645,63323024710)
Levothyroxine sodium,levothyroxine sodium (006653,74706811)
Levothyroxine sodium,levothyroxine sodium (006648,74434113)
Levothyroxine sodium,levothyroxine sodium (006650,74518211)
Levothyroxine sodium,levothyroxine sodium (006651,74662411)
Neonate, birthwt > 2499g w/ major anomaly (APR,6331)
Other skin & subq i & d (8604)
Bilat simple mastectomy (8542)
Glaucoma nos (3659)
glaucoma
Parathyroid reimplant (695)
Nb obs genetc/metabl cnd (V293)
Hx of breast malignancy (V103)
Perc ins extracran stent (64)
Levothyroxine sodium,levothyroxine sodium (006645,55390088010)
Neonate, bwt 2000-2499g, normal newborn or neonate w other problem (APR,6262)
Heavy-for-date infan nec (7661)
synthroid
Hx of thyroid malignancy (V1087)
hypothyroid
cataract
allopurinol
Transfus prev auto blood (9902)
osteoporosi
Mammoplasty nec (8589)
Postsurgical hypothyroid (2440)
Acq absnce breast/nipple (V4571)
breast
mastectomi
lumpectomi
gout
Unilat simple mastectomy (8541)
Latanoprost 0.005% ophth. soln.,latanoprost 0.005% ophth. soln. (027370,13830304)
Levothyroxine sodium,levothyroxine sodium (006649,74455211)
Allopurinol,allopurinol (002535,51079020520)
Levothyroxine sodium,levothyroxine sodium (006652,74929613)
colchicin
Macular degeneration nos (36250)
Unilat exten simp mastec (8543)
Timolol maleate 0.5%,timolol maleate 0.5% (007856,61314022705)
Other parathyroidectomy (689)
Thyroid, parathyroid & thyroglossal procedures (APR,4042)
Thyroid, parathyroid & thyroglossal procedures (APR,4043)
Levothyroxine sodium,levothyroxine sodium (006655,74707013)
Colchicine,colchicine (008334,143120125)

Topic 14

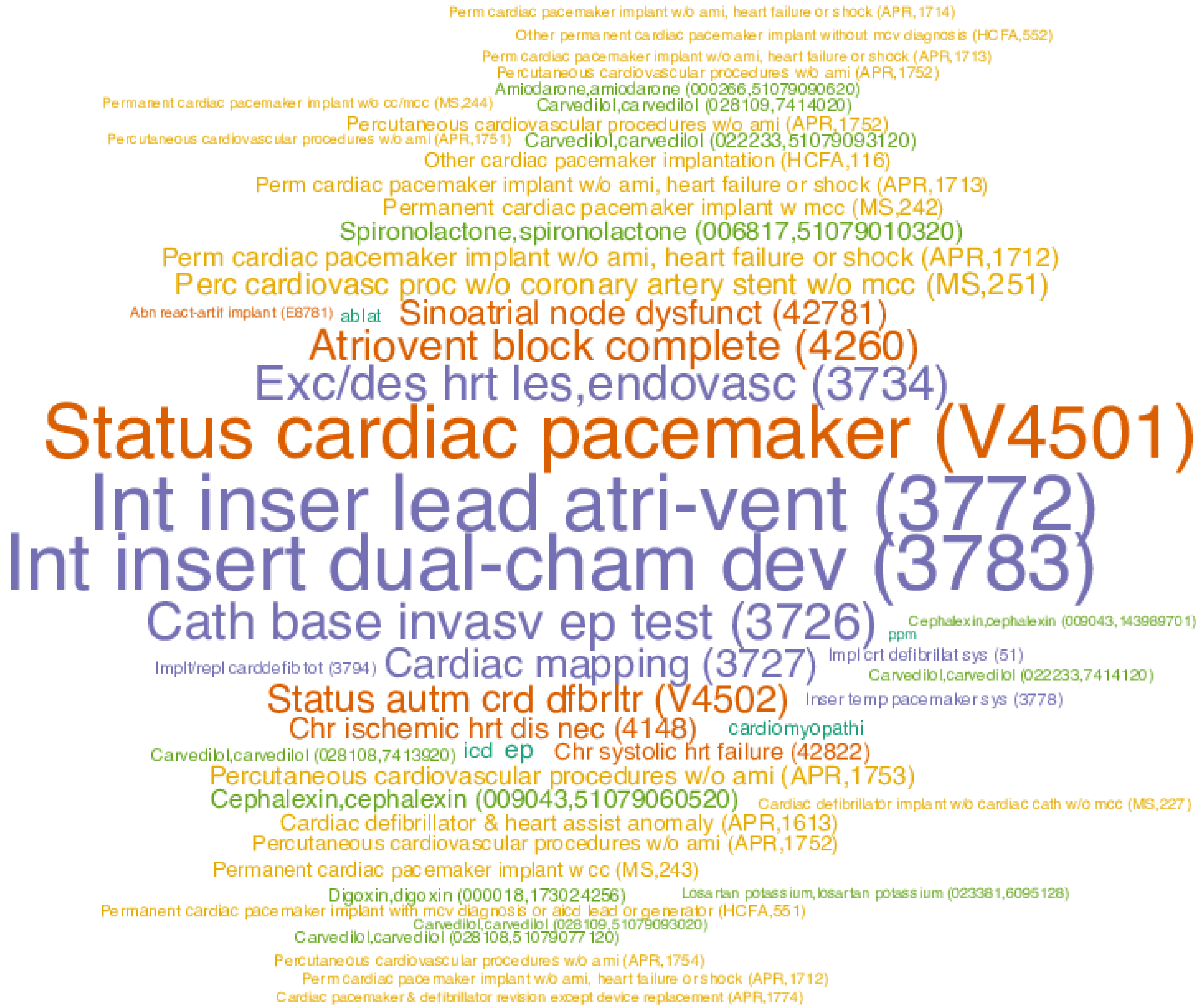

Perm cardiac pacemaker implant w/o ami, heart failure or shock (APR,1714)
Other permanent cardiac pacemaker implant without mcv diagnosis (HCFA,552)
Perm cardiac pacemaker implant w/o ami, heart failure or shock (APR,1713)
Percutaneous cardiovascular procedures w/o ami (APR,1752)
Amiodarone,amiodarone (000266,51079090620)
Permanent cardiac pacemaker implant w/o cc/mcc (MS,244)
Carvedilol,carvedilol (028109,7414020)
Percutaneous cardiovascular procedures w/o ami (APR,1752)
Percutaneous cardiovascular procedures w/o ami (APR,1751)
Carvedilol,carvedilol (022233,51079093120)
Other cardiac pacemaker implantation (HCFA,116)
Perm cardiac pacemaker implant w/o ami, heart failure or shock (APR,1713)
Permanent cardiac pacemaker implant w mcc (MS,242)
Spironolactone,spironolactone (006817,51079010320)
Perm cardiac pacemaker implant w/o ami, heart failure or shock (APR,1712)
Perc cardiovasc proc w/o coronary artery stent w/o mcc (MS,251)
Abn react-artif implant (E8781)
ablat
Sinoatrial node dysfunct (42781)
Atriovent block complete (4260)
Exc/des hrt les,endovasc (3734)
Status cardiac pacemaker (V4501)
Int inser lead atri-vent (3772)
Int insert dual-cham dev (3783)
Cath base invasv ep test (3726)
Cephalexin,cephalexin (009043,143989701)
ppm
Implt/repl carddefib tot (3794)
Cardiac mapping (3727)
Impl crt defibrillat sys (51)
Carvedilol,carvedilol (022233,7414120)
Status autm crd dfbrltr (V4502)
Inser temp pacemaker sys (3778)
Chr ischemic hrt dis nec (4148)
cardiomyopathi
Carvedilol,carvedilol (028108,7413920)
icd
ep
Chr systolic hrt failure (42822)
Percutaneous cardiovascular procedures w/o ami (APR,1753)
Cephalexin,cephalexin (009043,51079060520)
Cardiac defibrillator implant w/o cardiac cath w/o mcc (MS,227)
Cardiac defibrillator & heart assist anomaly (APR,1613)
Percutaneous cardiovascular procedures w/o ami (APR,1752)
Permanent cardiac pacemaker implant w cc (MS,243)
Digoxin,digoxin (000018,173024256)
Losartan potassium,losartan potassium (023381,6095128)
Permanent cardiac pacemaker implant with mcv diagnosis or aicd lead or generator (HCFA,551)
Carvedilol,carvedilol (028109,51079093020)
Carvedilol,carvedilol (028108,51079077120)
Percutaneous cardiovascular procedures w/o ami (APR,1754)
Perm cardiac pacemaker implant w/o ami, heart failure or shock (APR,1712)
Cardiac pacemaker & defibrillator revision except device replacement (APR,1774)

Topic 15

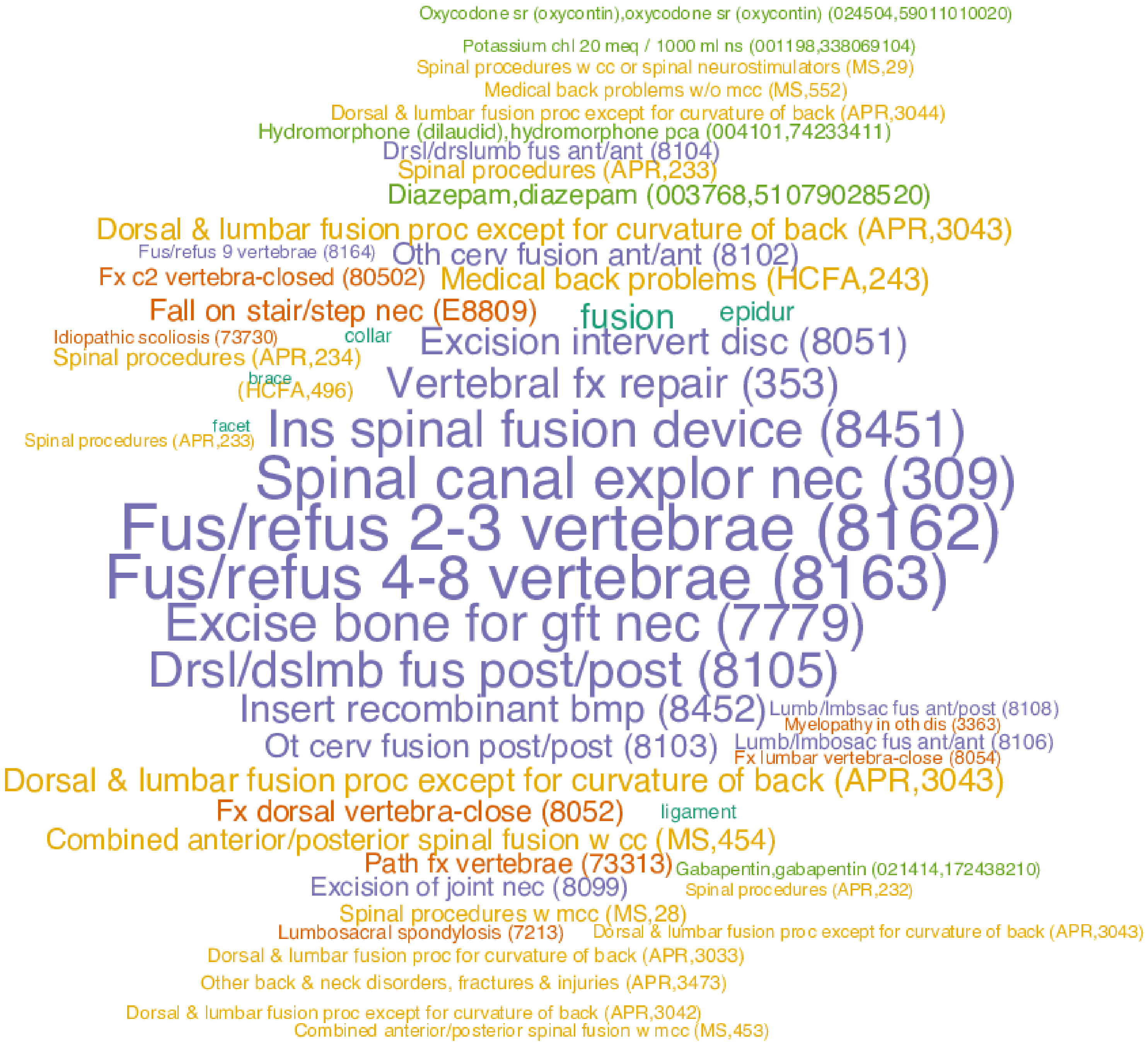

Oxycodone sr (oxycontin),oxycodone sr (oxycontin) (024504,59011010020)
Potassium chl 20 meq / 1000 ml ns (001198,338069104)
Spinal procedures w cc or spinal neurostimulators (MS,29)
Medical back problems w/o mcc (MS,552)
Dorsal & lumbar fusion proc except for curvature of back (APR,3044)
Hydromorphone (dilaudid),hydromorphone pca (004101,74233411)
Drsl/drslumb fus ant/ant (8104)
Spinal procedures (APR,233)
Diazepam,diazepam (003768,51079028520)
Dorsal & lumbar fusion proc except for curvature of back (APR,3043)
Fus/refus 9 vertebrae (8164)
Oth cerv fusion ant/ant (8102)
Fx c2 vertebra-closed (80502)
Medical back problems (HCFA,243)
Fall on stair/step nec (E8809)
fusion
epidur
Idiopathic scoliosis (73730)
collar
Excision intervert disc (8051)
Spinal procedures (APR,234)
brace
(HCFA,496)
Vertebral fx repair (353)
facet
Spinal procedures (APR,233)
Ins spinal fusion device (8451)
Spinal canal explor nec (309)
Fus/refus 2-3 vertebrae (8162)
Fus/refus 4-8 vertebrae (8163)
Excise bone for gft nec (7779)
Drsl/dslmb fus post/post (8105)
Insert recombinant bmp (8452)
Lumb/lmbsac fus ant/post (8108)
Myelopathy in oth dis (3363)
Ot cerv fusion post/post (8103)
Lumb/lmbosac fus ant/ant (8106)
Fx lumbar vertebra-close (8054)
Dorsal & lumbar fusion proc except for curvature of back (APR,3043)
Fx dorsal vertebra-close (8052)
ligament
Combined anterior/posterior spinal fusion w cc (MS,454)
Path fx vertebrae (73313)
Gabapentin,gabapentin (021414,172438210)
Excision of joint nec (8099)
Spinal procedures (APR,232)
Spinal procedures w mcc (MS,28)
Lumbosacral spondylosis (7213)
Dorsal & lumbar fusion proc except for curvature of back (APR,3043)
Dorsal & lumbar fusion proc for curvature of back (APR,3033)
Other back & neck disorders, fractures & injuries (APR,3473)
Dorsal & lumbar fusion proc except for curvature of back (APR,3042)
Combined anterior/posterior spinal fusion w mcc (MS,453)

Topic 16

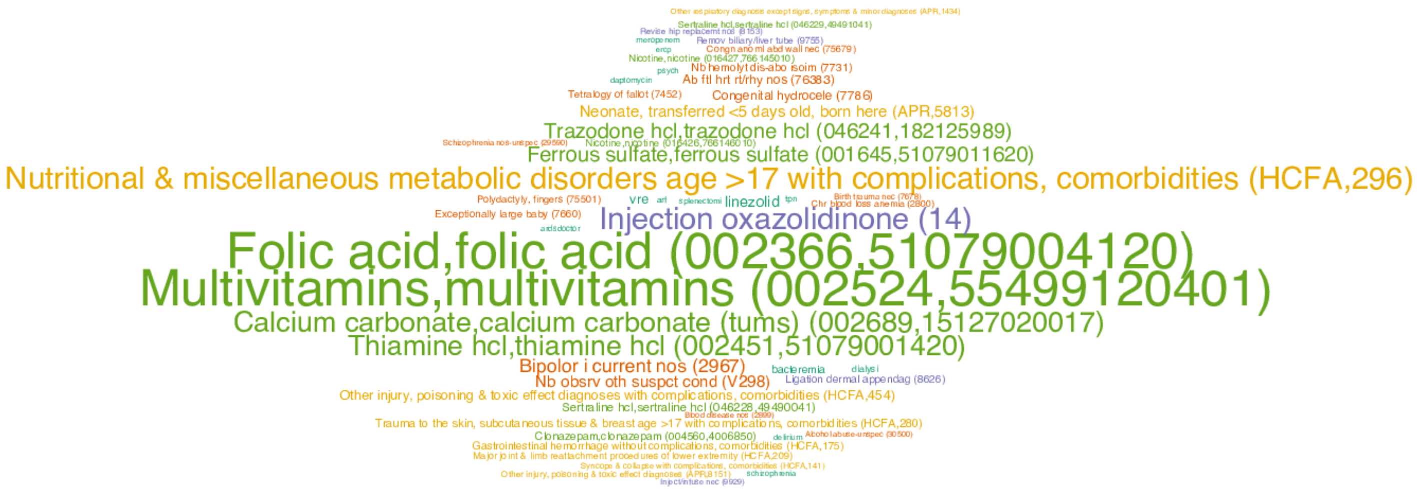

Other respiratory diagnosis except signs, symptoms & minor diagnoses (APR,1434)
Sertraline hcl,sertraline hcl (046229,49491041)
Revise hip replacemt nos (8153)
meropenem
Remov biliary/liver tube (9755)
ercp
Congn anoml abd wall nec (75679)
Nicotine,nicotine (016427,766145010)
Nb hemolyt dis-abo isoim (7731)
psych
daptomycin
Ab ftl hrt rt/rhy nos (76383)
Tetralogy of fallot (7452)
Congenital hydrocele (7786)
Neonate, transferred <5 days old, born here (APR,5813)
Trazodone hcl,trazodone hcl (046241,182125989)
Schizophrenia nos-unspec (29590)
Nicotine,nicotine (016426,766146010)
Ferrous sulfate,ferrous sulfate (001645,51079011620)
Nutritional & miscellaneous metabolic disorders age >17 with complications, comorbidities (HCFA,296)
Polydactyly, fingers (75501)
vre
art
splenectomi
linezolid
tpn
Birth trauma nec (7678)
Chr blood loss anemia (2800)
Exceptionally large baby (7660)
Injection oxazolidinone (14)
aidsdoctor
Folic acid,folic acid (002366,51079004120)
Multivitamins,multivitamins (002524,55499120401)
Calcium carbonate,calcium carbonate (tums) (002689,15127020017)
Thiamine hcl,thiamine hcl (002451,51079001420)
Bipolor i current nos (2967)
bacteremia
dialysi
Nb obsrv oth suspct cond (V298)
Ligation dermal appendag (8626)
Other injury, poisoning & toxic effect diagnoses with complications, comorbidities (HCFA,454)
Sertraline hcl,sertraline hcl (046228,49490041)
Blood disease nos (2899)
Trauma to the skin, subcutaneous tissue & breast age >17 with complications, comorbidities (HCFA,280)
Clonazepam,clonazepam (004560,4006850)
delirium
Alcohol abuse-unspec (30500)
Gastrointestinal hemorrhage without complications, comorbidities (HCFA,175)
Major joint & limb reattachment procedures of lower extremity (HCFA,209)
Syncope & collapse with complications, comorbidities (HCFA,141)
Other injury, poisoning & toxic effect diagnoses (APR,8151)
schizophrenia
Inject/infuse nec (9929)

Topic 17

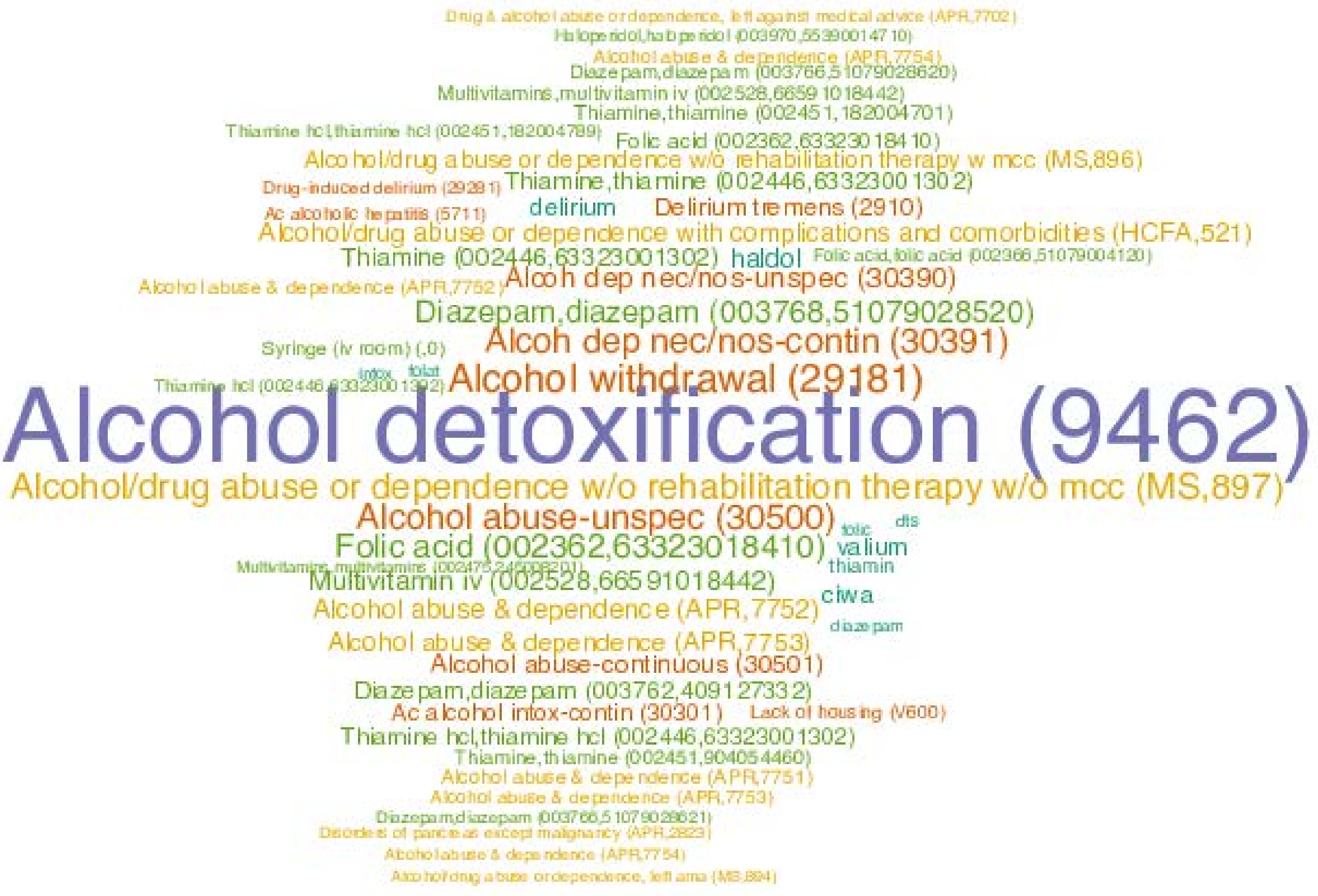

Drug & alcohol abuse or dependence, left against medical advice (APR,7702)
Haloperidol,haloperidol (003970,55390014710)
Alcohol abuse & dependence (APR,7754)
Diazepam,diazepam (003766,51079028620)
Multivitamins,multivitamin iv (002528,66591018442)
Thiamine,thiamine (002451,182004701)
Thiamine hcl,thiamine hcl (002451,182004789)
Folic acid (002362,63323018410)
Alcohol/drug abuse or dependence w/o rehabilitation therapy w mcc (MS,896)
Drug-induced delirium (29281)
Thiamine,thiamine (002446,63323001302)
Ac alcoholic hepatitis (5711)
delirium
Delirium tremens (2910)
Alcohol/drug abuse or dependence with complications and comorbidities (HCFA,521)
Thiamine (002446,63323001302)
haldol
Folic acid,folic acid (002366,51079004120)
Alcohol abuse & dependence (APR,7752)
Alcoh dep nec/nos-unspec (30390)
Diazepam,diazepam (003768,51079028520)
Syringe (iv room) (,0)
Alcoh dep nec/nos-contin (30391)
intox
folat
Alcohol withdrawal (29181)
Alcohol detoxification (9462)
Alcohol/drug abuse or dependence w/o rehabilitation therapy w/o mcc (MS,897)
Alcohol abuse-unspec (30500)
folic
dts
Folic acid (002362,63323018410)
valium
thiamin
Multivitamin iv (002528,66591018442)
ciwa
Alcohol abuse & dependence (APR,7752)
diazepam
Alcohol abuse & dependence (APR,7753)
Alcohol abuse-continuous (30501)
Diazepam,diazepam (003762,409127332)
Ac alcohol intox-contin (30301)
Lack of housing (V600)
Thiamine hcl,thiamine hcl (002446,63323001302)
Thiamine,thiamine (002451,904054460)
Alcohol abuse & dependence (APR,7751)
Alcohol abuse & dependence (APR,7753)
Diazepam,diazepam (003766,51079028621)
Disorders of pancreas except malignancy (APR,2823)
Alcohol abuse & dependence (APR,7754)

Topic 18

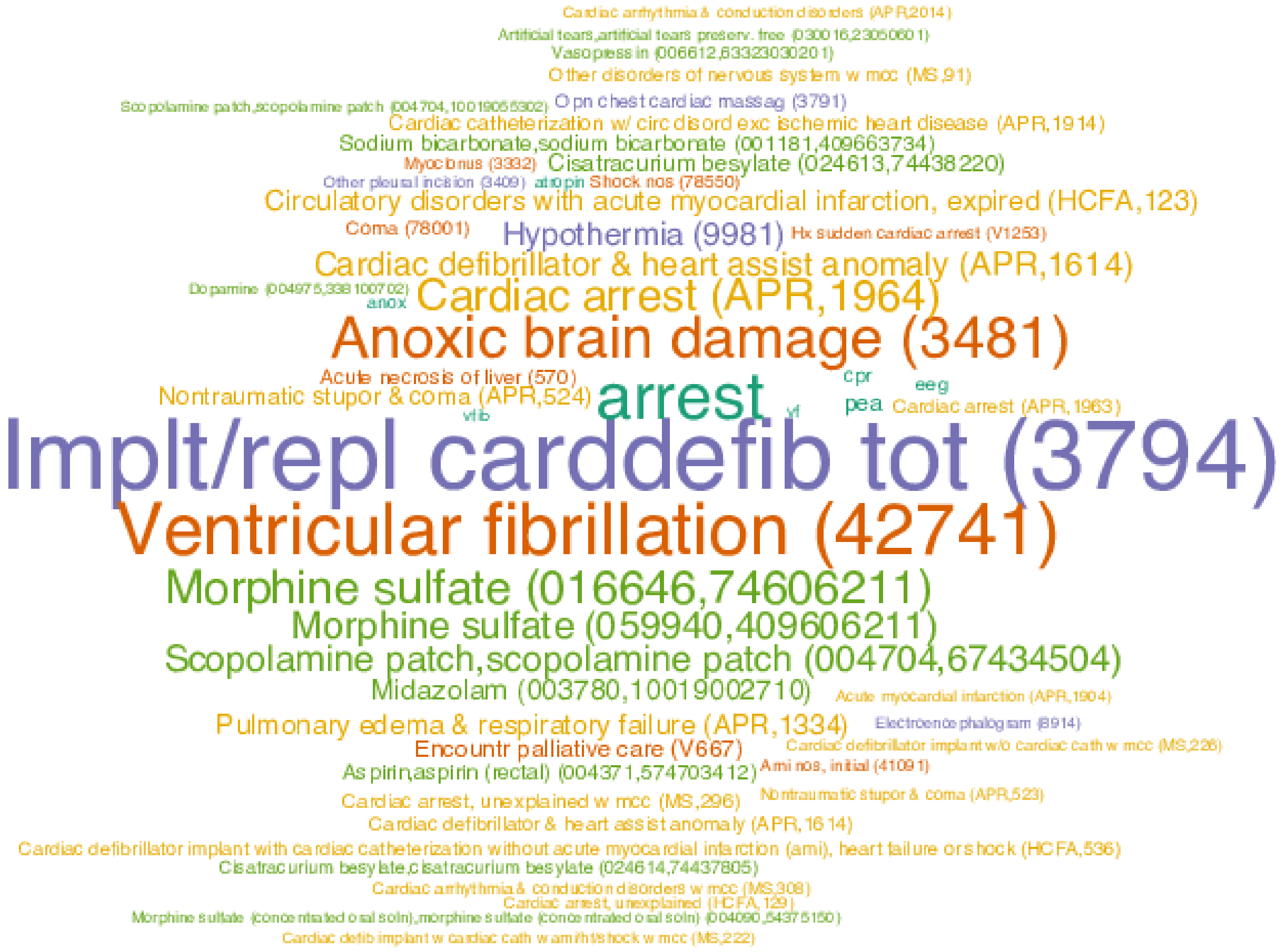
Cardiac arrhythmia & conduction disorders (APR,2014)
Artificial tears,artificial tears preserv. free (030016,23050601)
Vasopressin (006612,63323030201)
Other disorders of nervous system w mcc (MS,91)
Scopolamine patch,scopolamine patch (004704,10019055302)
Opn chest cardiac massag (3791)
Cardiac catheterization w/ circ disord exc ischemic heart disease (APR,1914)
Sodium bicarbonate,sodium bicarbonate (001181,409663734)
Myoclonus (3332)
Cisatracurium besylate (024613,74438220)
Other pleural incision (3409)
atropin
Shock nos (78550)
Circulatory disorders with acute myocardial infarction, expired (HCFA,123)
Coma (78001)
Hypothermia (9981)
Hx sudden cardiac arrest (V1253)
Cardiac defibrillator & heart assist anomaly (APR,1614)
Dopamine (004975,338100702)
anox
Cardiac arrest (APR,1964)
Anoxic brain damage (3481)
Acute necrosis of liver (570)
cpr
eeg
Nontraumatic stupor & coma (APR,524)
arrest
vfib
vf
pea
Cardiac arrest (APR,1963)
Implt/repl carddefib tot (3794)
Ventricular fibrillation (42741)
Morphine sulfate (016646,74606211)
Morphine sulfate (059940,409606211)
Scopolamine patch,scopolamine patch (004704,67434504)
Midazolam (003780,10019002710)
Acute myocardial infarction (APR,1904)
Pulmonary edema & respiratory failure (APR,1334)
Electroencephalogram (8914)
Encountr palliative care (V667)
Cardiac defibrillator implant w/o cardiac cath w mcc (MS,226)
Aspirin,aspirin (rectal) (004371,57470341 2)
Ami nos, initial (41091)
Cardiac arrest, unexplained w mcc (MS,296)
Nontraumatic stupor & coma (APR,523)
Cardiac defibrillator & heart assist anomaly (APR,1614)
Cardiac defibrillator implant with cardiac catheterization without acute myocardial infarction (ami), heart failure or shock (HCFA,536)
Cisatracurium besylate,cisatracurium besylate (024614,74437805)
Cardiac arrhythmia & conduction disorders w mcc (MS,308)
Cardiac arrest, unexplained (HCFA,129)
Morphine sulfate (concentrated oral soln),morphine sulfate (concentrated oral soln) (004090,54375150)
Cardiac defib implant w cardiac cath w ami/hf/shock w mcc (MS,222)

Topic 19

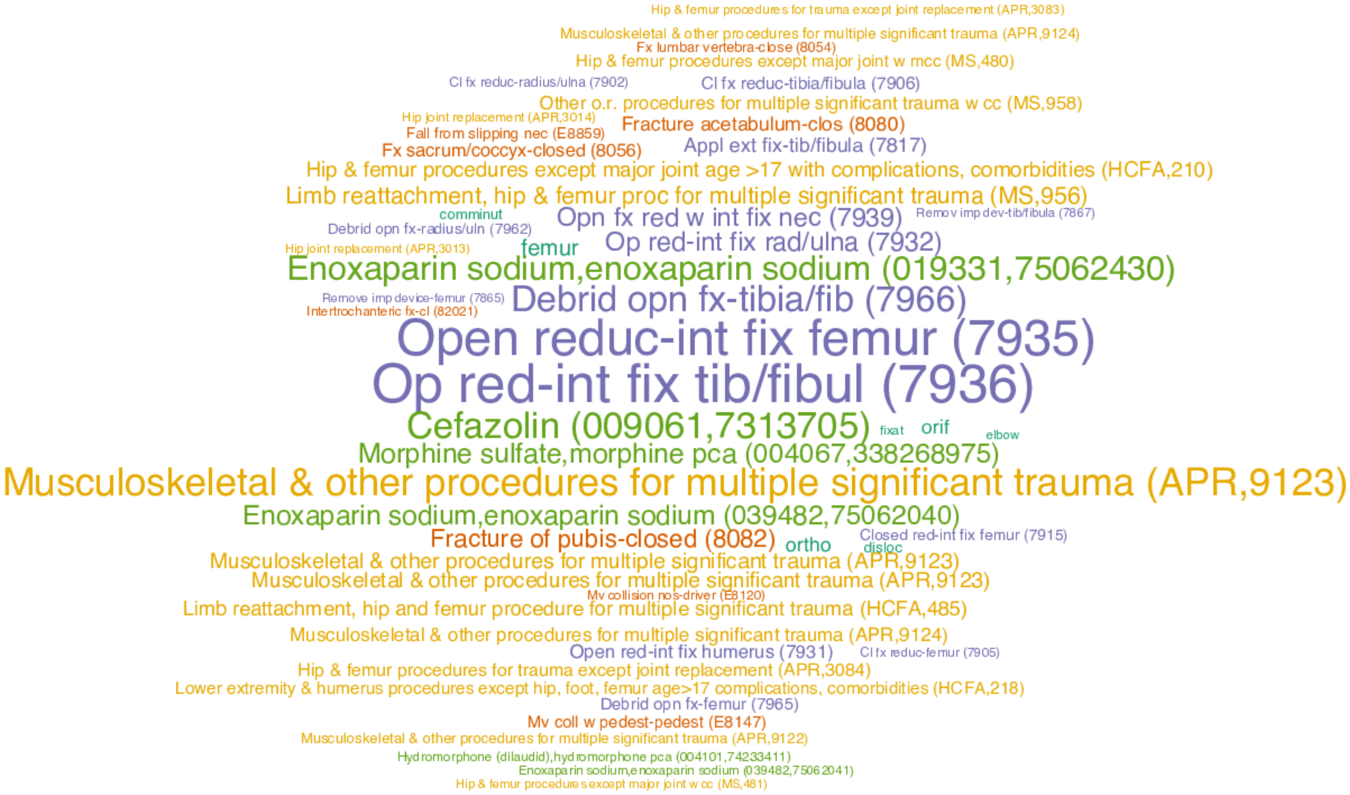
Hip & femur procedures for trauma except joint replacement (APR,3083)
Musculoskeletal & other procedures for multiple significant trauma (APR,9124)
Fx lumbar vertebra-close (8054)
Hip & femur procedures except major joint w mcc (MS,480)
Cl fx reduc-radius/ulna (7902)
Cl fx reduc-tibia/fibula (7906)
Other o.r. procedures for multiple significant trauma w cc (MS,958)
Hip joint replacement (APR,3014)
Fracture acetabulum-clos (8080)
Fall from slipping nec (E8859)
Fx sacrum/coccyx-closed (8056)
Appl ext fix-tib/fibula (7817)
Hip & femur procedures except major joint age >17 with complications, comorbidities (HCFA,210)
Limb reattachment, hip & femur proc for multiple significant trauma (MS,956)
comminut
Opn fx red w int fix nec (7939)
Remov imp dev-tib/fibula (7867)
Debrid opn fx-radius/uln (7962)
Hip joint replacement (APR,3013)
femur
Op red-int fix rad/ulna (7932)
Enoxaparin sodium,enoxaparin sodium (019331,75062430)
Remove imp device-femur (7865)
Debrid opn fx-tibia/fib (7966)
Intertrochanteric fx-cl (82021)
Open reduc-int fix femur (7935)
Op red-int fix tib/fibul (7936)
Cefazolin (009061,7313705)
fixat
orif
elbow
Morphine sulfate,morphine pca (004067,338268975)
Musculoskeletal & other procedures for multiple significant trauma (APR,9123)
Enoxaparin sodium,enoxaparin sodium (039482,75062040)
Fracture of pubis-closed (8082)
ortho
Closed red-int fix femur (7915)
disloc
Musculoskeletal & other procedures for multiple significant trauma (APR,9123)
Musculoskeletal & other procedures for multiple significant trauma (APR,9123)
Mv collision nos-driver (E8120)
Limb reattachment, hip and femur procedure for multiple significant trauma (HCFA,485)
Musculoskeletal & other procedures for multiple significant trauma (APR,9124)
Open red-int fix humerus (7931)
Cl fx reduc-femur (7905)
Hip & femur procedures for trauma except joint replacement (APR,3084)
Lower extremity & humerus procedures except hip, foot, femur age>17 complications, comorbidities (HCFA,218)
Debrid opn fx-femur (7965)
Mv coll w pedest-pedest (E8147)
Musculoskeletal & other procedures for multiple significant trauma (APR,9122)
Hydromorphone (dilaudid),hydromorphone pca (004101,74233411)
Enoxaparin sodium,enoxaparin sodium (039482,75062041)
Hip & femur procedures except major joint w cc (MS,481)

Topic 20

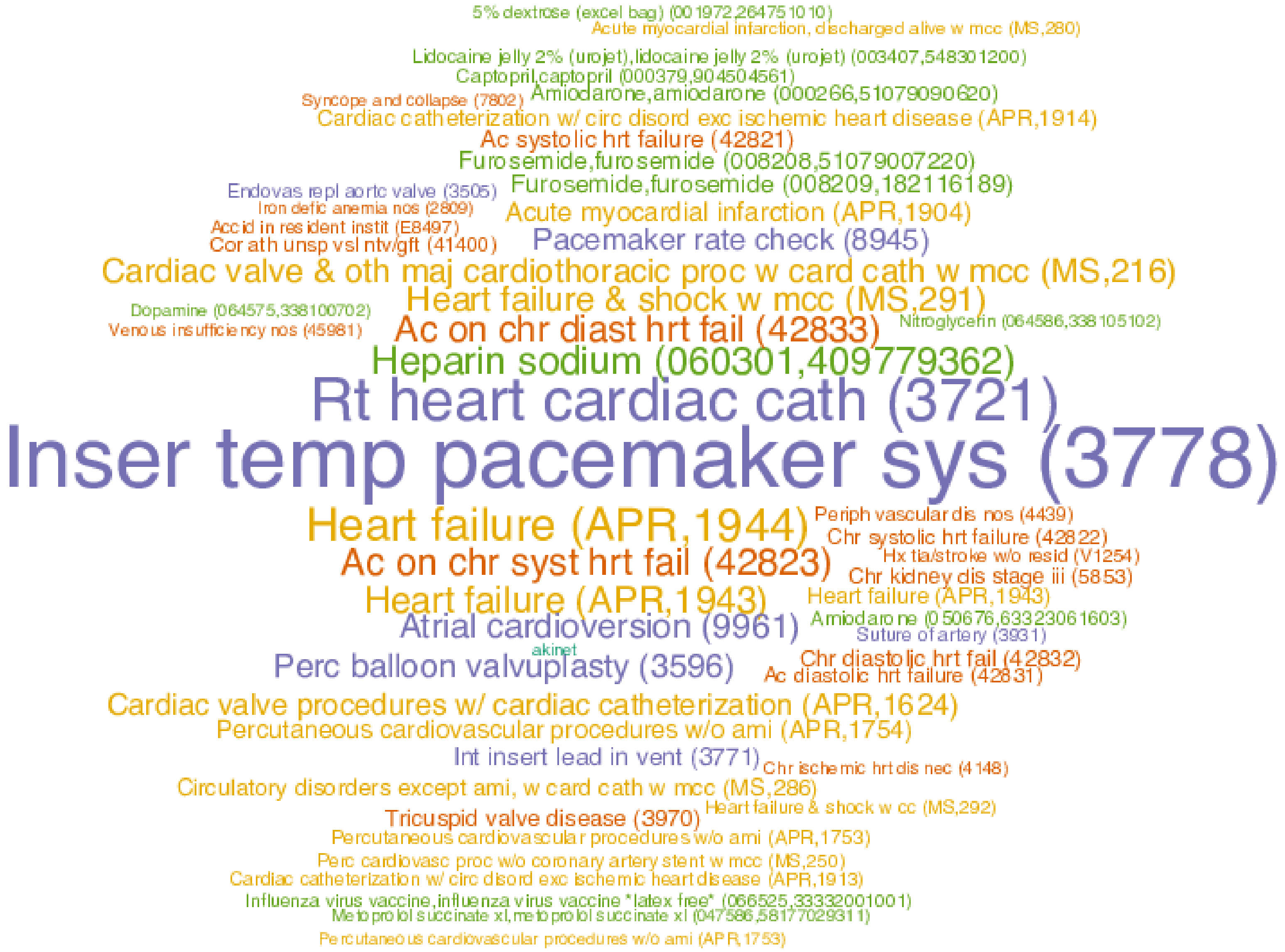

5% dextrose (excel bag) (001972,264751010)
Acute myocardial infarction, discharged alive w mcc (MS,280)
Lidocaine jelly 2% (urojet),lidocaine jelly 2% (urojet) (003407,548301200)
Captopril,captopril (000379,904504561)
Syncope and collapse (7802)
Amiodarone,amiodarone (000266,51079090620)
Cardiac catheterization w/ circ disord exc ischemic heart disease (APR,1914)
Ac systolic hrt failure (42821)
Furosemide,furosemide (008208,51079007220)
Endovas repl aortc valve (3505)
Furosemide,furosemide (008209,182116189)
Iron defic anemia nos (2809)
Acute myocardial infarction (APR,1904)
Accid in resident instit (E8497)
Cor ath unsp vsl ntv/gft (41400)
Pacemaker rate check (8945)
Cardiac valve & oth maj cardiothoracic proc w card cath w mcc (MS,216)
Heart failure & shock w mcc (MS,291)
Dopamine (064575,338100702)
Venous insufficiency nos (45981)
Ac on chr diast hrt fail (42833)
Nitroglycerin (064586,338105102)
Heparin sodium (060301,409779362)
Rt heart cardiac cath (3721)
Inser temp pacemaker sys (3778)
Heart failure (APR,1944)
Periph vascular dis nos (4439)
Chr systolic hrt failure (42822)
Ac on chr syst hrt fail (42823)
Hx tia/stroke w/o resid (V1254)
Chr kidney dis stage iii (5853)
Heart failure (APR,1943)
Heart failure (APR,1943)
Amiodarone (050676,63323061603)
Atrial cardioversion (9961)
Suture of artery (3931)
akinet
Perc balloon valvuplasty (3596)
Chr diastolic hrt fail (42832)
Ac diastolic hrt failure (42831)
Cardiac valve procedures w/ cardiac catheterization (APR,1624)
Percutaneous cardiovascular procedures w/o ami (APR,1754)
Int insert lead in vent (3771)
Chr ischemic hrt dis nec (4148)
Circulatory disorders except ami, w card cath w mcc (MS,286)
Heart failure & shock w cc (MS,292)
Tricuspid valve disease (3970)
Percutaneous cardiovascular procedures w/o ami (APR,1753)
Perc cardiovasc proc w/o coronary artery stent w mcc (MS,250)
Cardiac catheterization w/ circ disord exc ischemic heart disease (APR,1913)
Influenza virus vaccine,influenza virus vaccine *latex free* (066525,33332001001)
Metoprolol succinate xl,metoprolol succinate xl (047586,58177029311)
Percutaneous cardiovascular procedures w/o ami (APR,1753)

Topic 21

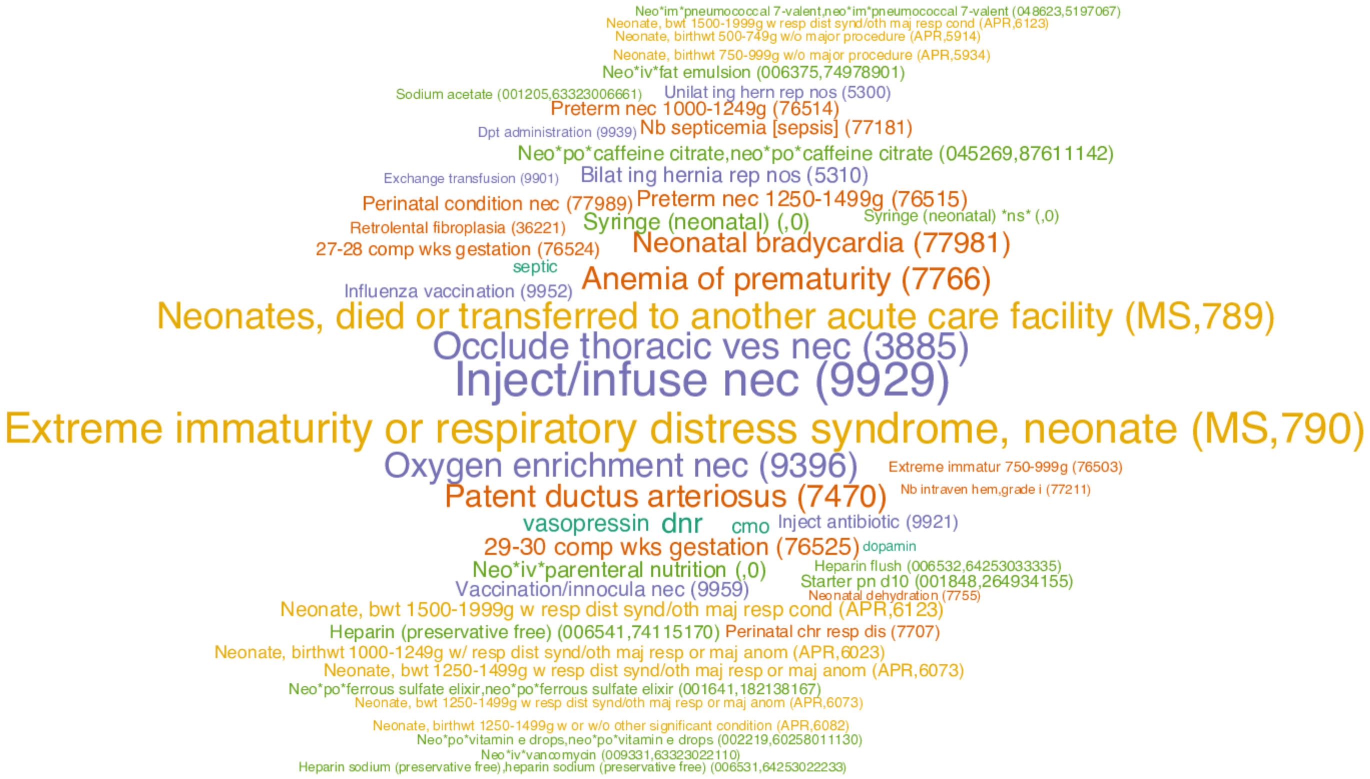
Neo*im*pneumococcal 7-valent,neo*im*pneumococcal 7-valent (048623,5197067)
Neonate, bwt 1500-1999g w resp dist synd/oth maj resp cond (APR,6123)
Neonate, birthwt 500-749g w/o major procedure (APR,5914)
Neonate, birthwt 750-999g w/o major procedure (APR,5934)
Neo*iv*fat emulsion (006375,74978901)
Sodium acetate (001205,63323006661)
Unilat ing hern rep nos (5300)
Preterm nec 1000-1249g (76514)
Dpt administration (9939)
Nb septicemia [sepsis] (77181)
Neo*po*caffeine citrate,neo*po*caffeine citrate (045269,87611142)
Exchange transfusion (9901)
Bilat ing hernia rep nos (5310)
Perinatal condition nec (77989)
Preterm nec 1250-1499g (76515)
Retrolental fibroplasia (36221)
Syringe (neonatal) (,0)
Syringe (neonatal) *ns* (,0)
27-28 comp wks gestation (76524)
Neonatal bradycardia (77981)
septic
Anemia of prematurity (7766)
Influenza vaccination (9952)
Neonates, died or transferred to another acute care facility (MS,789)
Occlude thoracic ves nec (3885)
Inject/infuse nec (9929)
Extreme immaturity or respiratory distress syndrome, neonate (MS,790)
Oxygen enrichment nec (9396)
Extreme immatur 750-999g (76503)
Patent ductus arteriosus (7470)
Nb intraven hem,grade i (77211)
vasopressin
dnr
cmo
Inject antibiotic (9921)
29-30 comp wks gestation (76525)
dopamin
Neo*iv*parenteral nutrition (,0)
Heparin flush (006532,64253033335)
Starter pn d10 (001848,264934155)
Vaccination/innocula nec (9959)
Neonatal dehydration (7755)
Neonate, bwt 1500-1999g w resp dist synd/oth maj resp cond (APR,6123)
Heparin (preservative free) (006541,74115170)
Perinatal chr resp dis (7707)
Neonate, birthwt 1000-1249g w/ resp dist synd/oth maj resp or maj anom (APR,6023)
Neonate, bwt 1250-1499g w resp dist synd/oth maj resp or maj anom (APR,6073)
Neo*po*ferrous sulfate elixir,neo*po*ferrous sulfate elixir (001641,182138167)
Neonate, bwt 1250-1499g w resp dist synd/oth maj resp or maj anom (APR,6073)
Neonate, birthwt 1250-1499g w or w/o other significant condition (APR,6082)
Neo*po*vitamin e drops,neo*po*vitamin e drops (002219,60258011130)
Neo*iv*vancomycin (009331,63323022110)
Heparin sodium (preservative free),heparin sodium (preservative free) (006531,64253022233)

Topic 22

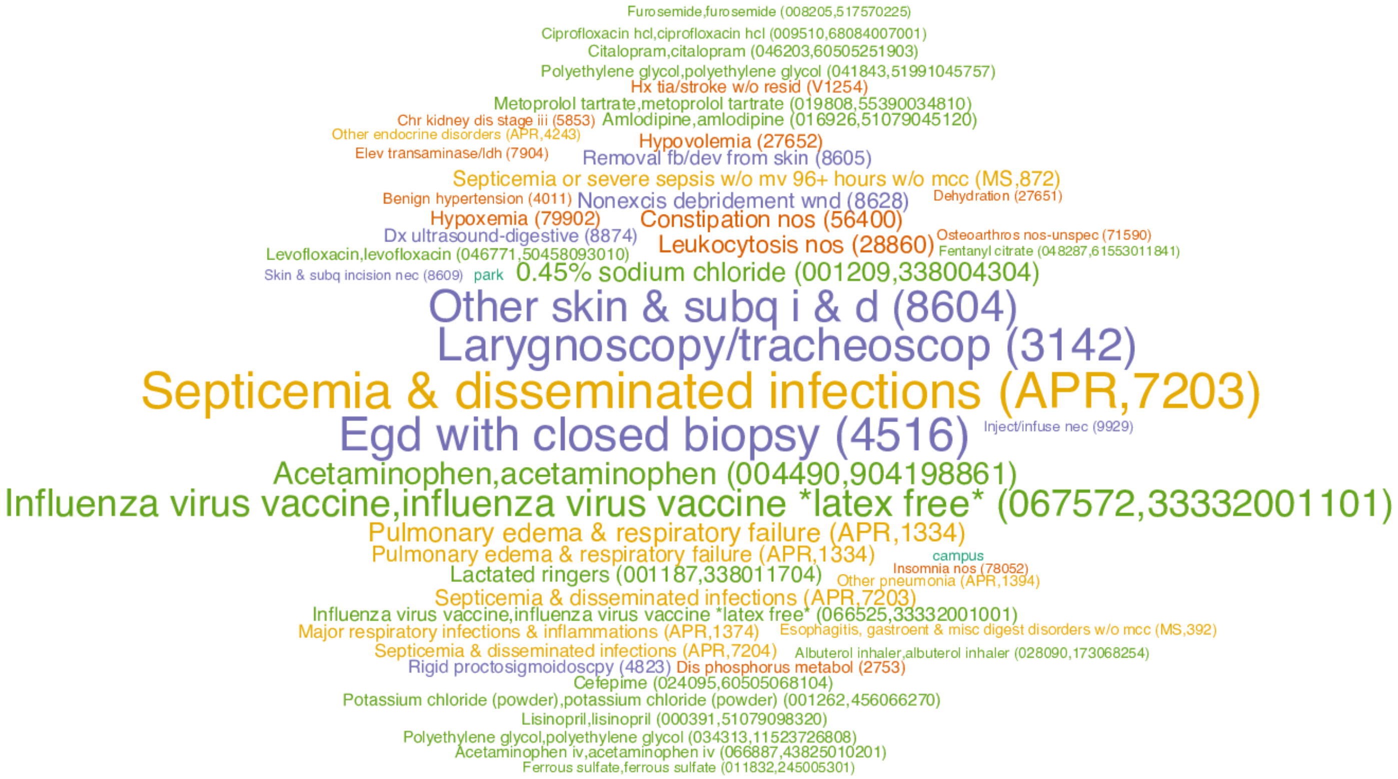

Furosemide,furosemide (008205,517570225)
Ciprofloxacin hcl,ciprofloxacin hcl (009510,68084007001)
Citalopram,citalopram (046203,60505251903)
Polyethylene glycol,polyethylene glycol (041843,51991045757)
Hx tia/stroke w/o resid (V1254)
Metoprolol tartrate,metoprolol tartrate (019808,55390034810)
Chr kidney dis stage iii (5853)
Amlodipine,amlodipine (016926,51079045120)
Other endocrine disorders (APR,4243)
Hypovolemia (27652)
Elev transaminase/ldh (7904)
Removal fb/dev from skin (8605)
Septicemia or severe sepsis w/o mv 96+ hours w/o mcc (MS,872)
Benign hypertension (4011)
Nonexcis debridement wnd (8628)
Dehydration (27651)
Hypoxemia (79902)
Constipation nos (56400)
Dx ultrasound-digestive (8874)
Leukocytosis nos (28860)
Osteoarthros nos-unspec (71590)
Levofloxacin,levofloxacin (046771,50458093010)
Fentanyl citrate (048287,61553011841)
Skin & subq incision nec (8609)
park
0.45% sodium chloride (001209,338004304)
Other skin & subq i & d (8604)
Larygnoscopy/tracheoscop (3142)
Septicemia & disseminated infections (APR,7203)
Egd with closed biopsy (4516)
Inject/infuse nec (9929)
Acetaminophen,acetaminophen (004490,904198861)
Influenza virus vaccine,influenza virus vaccine *latex free* (067572,33332001101)
Pulmonary edema & respiratory failure (APR,1334)
Pulmonary edema & respiratory failure (APR,1334)
campus
Lactated ringers (001187,338011704)
Insomnia nos (78052)
Other pneumonia (APR,1394)
Septicemia & disseminated infections (APR,7203)
Influenza virus vaccine,influenza virus vaccine *latex free* (066525,33332001001)
Major respiratory infections & inflammations (APR,1374)
Esophagitis, gastroent & misc digest disorders w/o mcc (MS,392)
Septicemia & disseminated infections (APR,7204)
Albuterol inhaler,albuterol inhaler (028090,173068254)
Rigid proctosigmoidoscpy (4823)
Dis phosphorus metabol (2753)
Cefepime (024095,60505068104)
Potassium chloride (powder),potassium chloride (powder) (001262,456066270)
Lisinopril,lisinopril (000391,51079098320)
Polyethylene glycol,polyethylene glycol (034313,11523726808)
Acetaminophen iv,acetaminophen iv (066887,43825010201)
Ferrous sulfate,ferrous sulfate (011832,245005301)

Topic 23

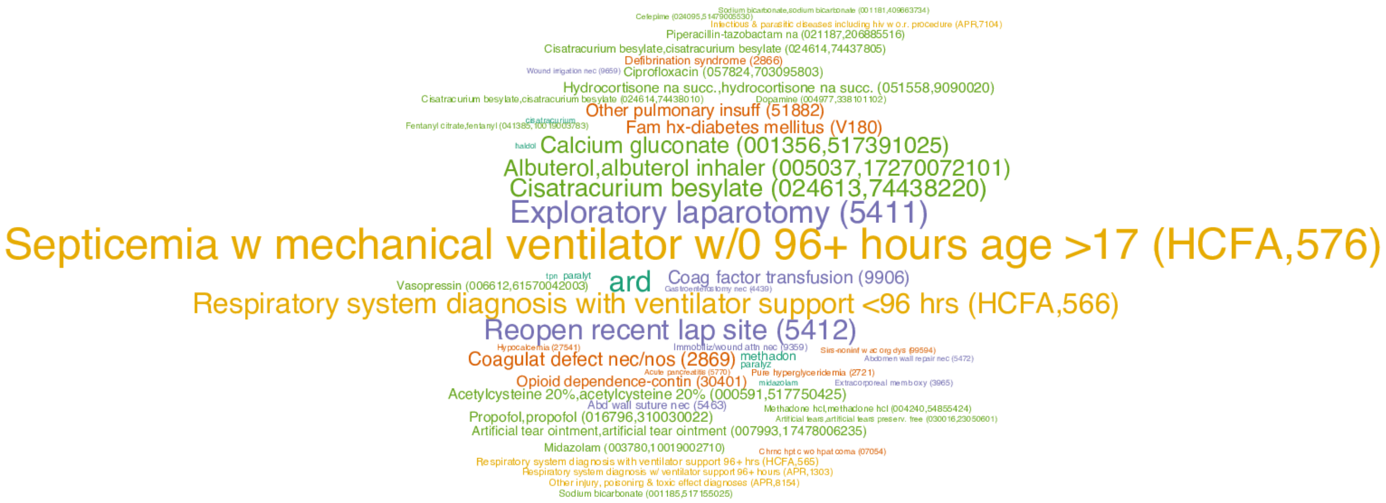

Sodium bicarbonate,sodium bicarbonate (001181,409663734)
Cefepime (024095,51479005530)
Infectious & parasitic diseases including hiv w o.r. procedure (APR,7104)
Piperacillin-tazobactam na (021187,206885516)
Cisatracurium besylate,cisatracurium besylate (024614,74437805)
Defibrination syndrome (2866)
Wound irrigation nec (9659)
Ciprofloxacin (057824,703095803)
Hydrocortisone na succ.,hydrocortisone na succ. (051558,9090020)
Cisatracurium besylate,cisatracurium besylate (024614,74438010)
Dopamine (004977,338101102)
Other pulmonary insuff (51882)
cisatracurium
Fentanyl citrate,fentanyl (041385,10019003783)
Fam hx-diabetes mellitus (V180)
haldol
Calcium gluconate (001356,517391025)
Albuterol,albuterol inhaler (005037,17270072101)
Cisatracurium besylate (024613,74438220)
Exploratory laparotomy (5411)
Septicemia w mechanical ventilator w/0 96+ hours age >17 (HCFA,576)
tpn
paralyt
Vasopressin (006612,61570042003)
ard
Coag factor transfusion (9906)
Gastroenterostomy nec (4439)
Respiratory system diagnosis with ventilator support <96 hrs (HCFA,566)
Reopen recent lap site (5412)
Hypocalcemia (27541)
Immobiliz/wound attn nec (9359)
Sirs-noninf w ac org dys (99594)
Coagulat defect nec/nos (2869)
methadon
paralyz
Abdomen wall repair nec (5472)
Acute pancreatitis (5770)
Pure hyperglyceridemia (2721)
Opioid dependence-contin (30401)
midazolam
Extracorporeal memb oxy (3965)
Acetylcysteine 20%,acetylcysteine 20% (000591,517750425)
Abd wall suture nec (5463)
Methadone hcl,methadone hcl (004240,54855424)
Propofol,propofol (016796,310030022)
Artificial tears,artificial tears preserv. free (030016,23050601)
Artificial tear ointment,artificial tear ointment (007993,17478006235)
Midazolam (003780,10019002710)
Chrnc hpt c wo hpat coma (07054)
Respiratory system diagnosis with ventilator support 96+ hrs (HCFA,565)
Respiratory system diagnosis w/ ventilator support 96+ hours (APR,1303)
Other injury, poisoning & toxic effect diagnoses (APR,8154)
Sodium bicarbonate (001185,517155025)

Topic 24

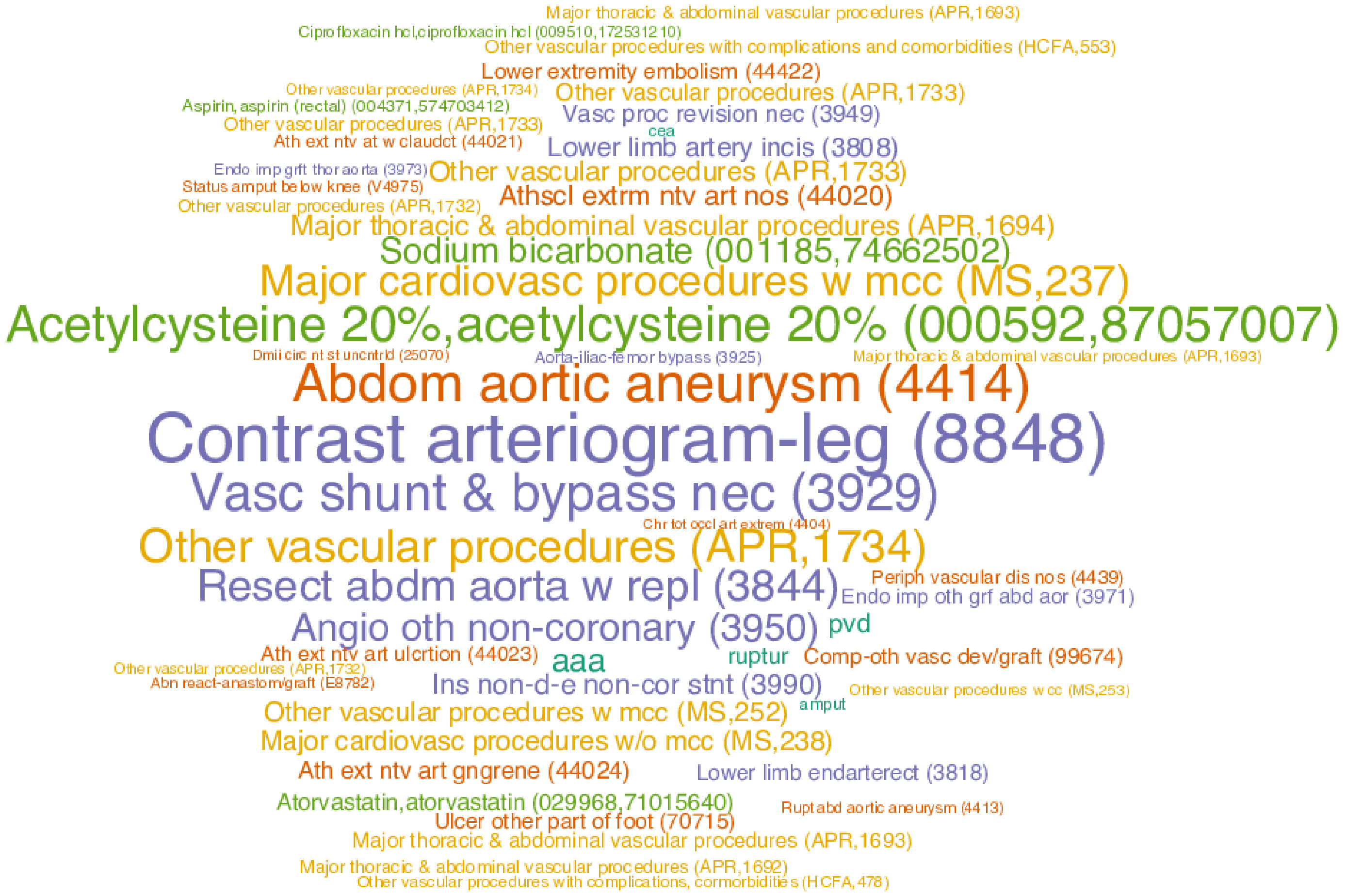

Major thoracic & abdominal vascular procedures (APR,1693)
Ciprofloxacin hcl,ciprofloxacin hcl (009510,172531210)
Other vascular procedures with complications and comorbidities (HCFA,553)
Lower extremity embolism (44422)
Other vascular procedures (APR,1734)
Other vascular procedures (APR,1733)
Aspirin,aspirin (rectal) (004371,574703412)
Vasc proc revision nec (3949)
Other vascular procedures (APR,1733)
cea
Ath ext ntv at w claudct (44021)
Lower limb artery incis (3808)
Endo imp grft thoraorta (3973)
Other vascular procedures (APR,1733)
Status amput below knee (V4975)
Athscl extrm ntv art nos (44020)
Other vascular procedures (APR,1732)
Major thoracic & abdominal vascular procedures (APR,1694)
Sodium bicarbonate (001185,74662502)
Major cardiovasc procedures w mcc (MS,237)
Acetylcysteine 20%,acetylcysteine 20% (000592,87057007)
Dmii circ nt st uncntrld (25070)
Aorta-iliac-femor bypass (3925)
Major thoracic & abdominal vascular procedures (APR,1693)
Abdom aortic aneurysm (4414)
Contrast arteriogram-leg (8848)
Vasc shunt & bypass nec (3929)
Chr tot occl art extrem (4404)
Other vascular procedures (APR,1734)
Resect abdm aorta w repl (3844)
Periph vascular dis nos (4439)
Endo imp oth grf abd aor (3971)
Angio oth non-coronary (3950)
pvd
Ath ext ntv art ulcrtion (44023)
aaa
ruptur
Comp-oth vasc dev/graft (99674)
Other vascular procedures (APR,1732)
Abn react-anastom/graft (E8782)
Ins non-d-e non-cor stnt (3990)
Other vascular procedures w cc (MS,253)
amput
Other vascular procedures w mcc (MS,252)
Major cardiovasc procedures w/o mcc (MS,238)
Ath ext ntv art gngrene (44024)
Lower limb endarterect (3818)
Atorvastatin,atorvastatin (029968,71015640)
Rupt abd aortic aneurysm (4413)
Ulcer other part of foot (70715)
Major thoracic & abdominal vascular procedures (APR,1693)
Major thoracic & abdominal vascular procedures (APR,1692)
Other vascular procedures with complications, comorbidities (HCFA,478)

Topic 25

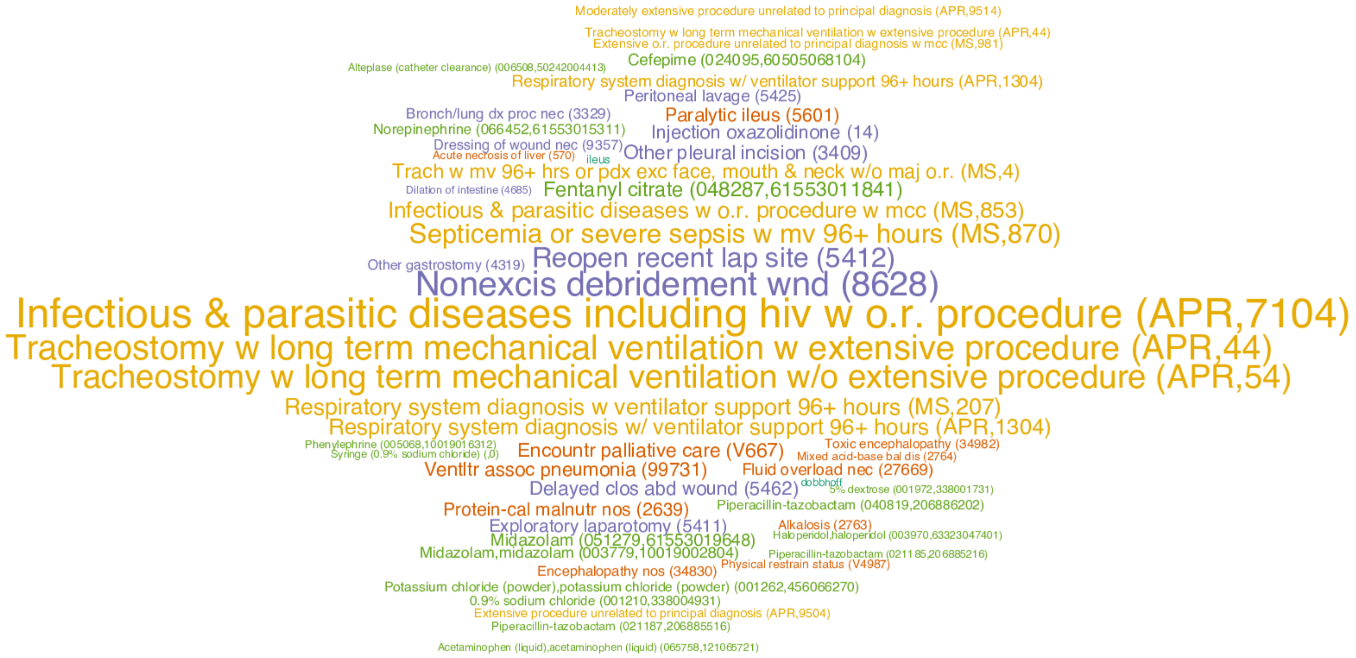

Moderately extensive procedure unrelated to principal diagnosis (APR,9514)
Tracheostomy w long term mechanical ventilation w extensive procedure (APR,44)
Extensive o.r. procedure unrelated to principal diagnosis w mcc (MS,981)
Alteplase (catheter clearance) (006508,50242004413)
Cefepime (024095,60505068104)
Respiratory system diagnosis w/ ventilator support 96+ hours (APR,1304)
Peritoneal lavage (5425)
Bronch/lung dx proc nec (3329)
Paralytic ileus (5601)
Norepinephrine (066452,61553015311)
Injection oxazolidinone (14)
Dressing of wound nec (9357)
Acute necrosis of liver (570)
ileus
Other pleural incision (3409)
Trach w mv 96+ hrs or pdx exc face, mouth & neck w/o maj o.r. (MS,4)
Dilation of intestine (4685)
Fentanyl citrate (048287,61553011841)
Infectious & parasitic diseases w o.r. procedure w mcc (MS,853)
Septicemia or severe sepsis w mv 96+ hours (MS,870)
Other gastrostomy (4319)
Reopen recent lap site (5412)
Nonexcis debridement wnd (8628)
Infectious & parasitic diseases including hiv w o.r. procedure (APR,7104)
Tracheostomy w long term mechanical ventilation w extensive procedure (APR,44)
Tracheostomy w long term mechanical ventilation w/o extensive procedure (APR,54)
Respiratory system diagnosis w ventilator support 96+ hours (MS,207)
Respiratory system diagnosis w/ ventilator support 96+ hours (APR,1304)
Phenylephrine (005068,10019016312)
Syringe (0.9% sodium chloride) (,0)
Encountr palliative care (V667)
Toxic encephalopathy (34982)
Mixed acid-base bal dis (2764)
Ventltr assoc pneumonia (99731)
Fluid overload nec (27669)
Delayed clos abd wound (5462)
dobbhoff
5% dextrose (001972,338001731)
Protein-cal malnutr nos (2639)
Piperacillin-tazobactam (040819,206886202)
Exploratory laparotomy (5411)
Alkalosis (2763)
Midazolam (051279,61553019648)
Haloperidol,haloperidol (003970,63323047401)
Midazolam,midazolam (003779,10019002804)
Piperacillin-tazobactam (021185,206885216)
Physical restrain status (V4987)
Encephalopathy nos (34830)
Potassium chloride (powder),potassium chloride (powder) (001262,456066270)
0.9% sodium chloride (001210,338004931)
Extensive procedure unrelated to principal diagnosis (APR,9504)
Piperacillin-tazobactam (021187,206885516)
Acetaminophen (liquid),acetaminophen (liquid) (065758,121065721)

Topic 26

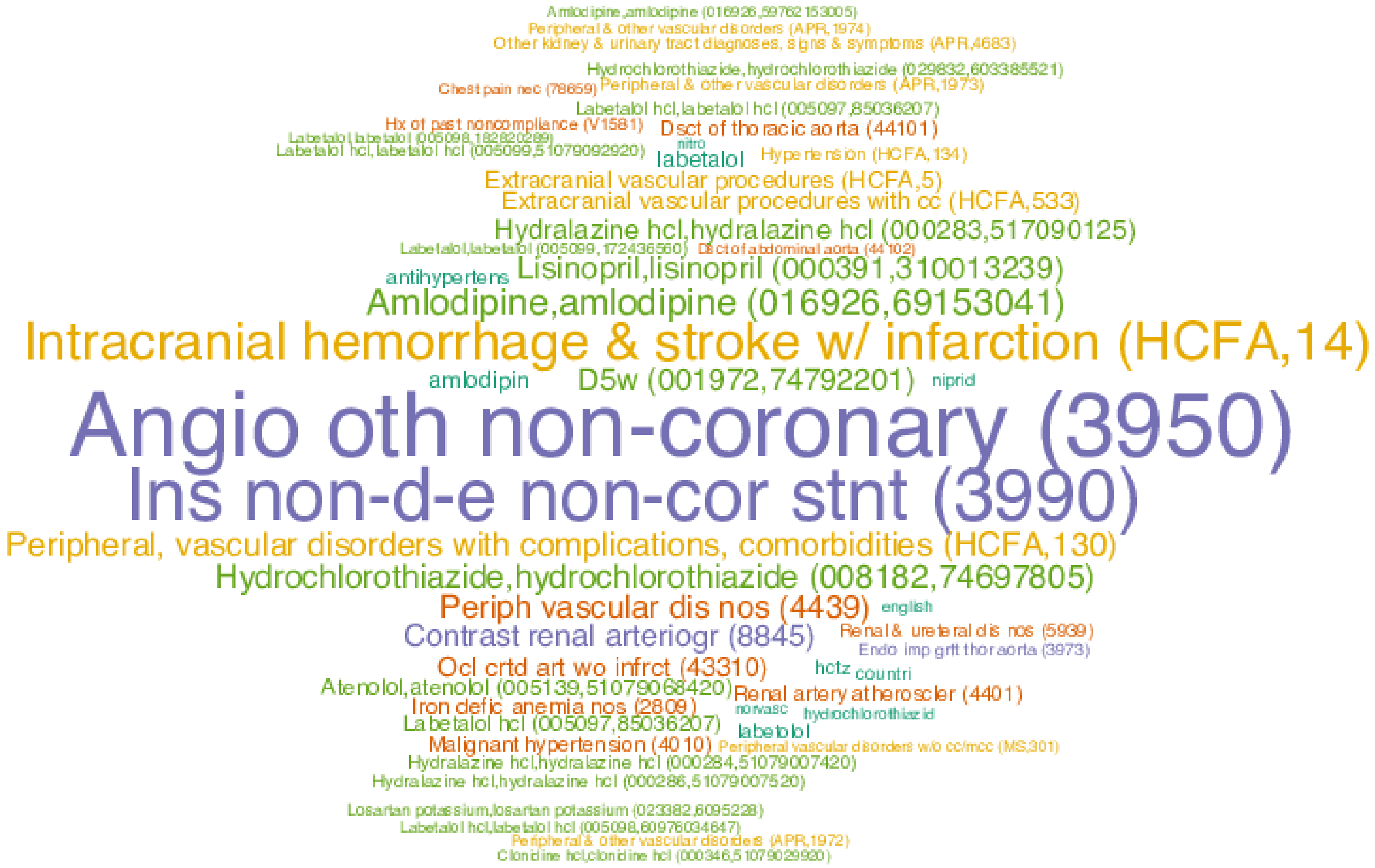

Amlodipine,amlodipine (016926,59762153005)
Peripheral & other vascular disorders (APR,1974)
Other kidney & urinary tract diagnoses, signs & symptoms (APR,4683)
Hydrochlorothiazide,hydrochlorothiazide (029832,603385521)
Chest pain nec (78659)
Peripheral & other vascular disorders (APR,1973)
Labetalol hcl,labetalol hcl (005097,85036207)
Hx of past noncompliance (V1581)
Dsct of thoracic aorta (44101)
Labetalol,labetalol (005098,182820289)
nitro
Labetalol hcl,labetalol hcl (005099,51079092920)
labetalol
Hypertension (HCFA,134)
Extracranial vascular procedures (HCFA,5)
Extracranial vascular procedures with cc (HCFA,533)
Hydralazine hcl,hydralazine hcl (000283,517090125)
Labetalol,labetalol (005099,172436560)
Dsct of abdominal aorta (44102)
antihypertens
Lisinopril,lisinopril (000391,310013239)
Amlodipine,amlodipine (016926,69153041)
Intracranial hemorrhage & stroke w/ infarction (HCFA,14)
amlodipin
D5w (001972,74792201)
niprid
Angio oth non-coronary (3950)
Ins non-d-e non-cor stnt (3990)
Peripheral, vascular disorders with complications, comorbidities (HCFA,130)
Hydrochlorothiazide,hydrochlorothiazide (008182,74697805)
Periph vascular dis nos (4439)
english
Contrast renal arteriogr (8845)
Renal & ureteral dis nos (5939)
Endo imp grft thor aorta (3973)
Ocl crtd art wo infrct (43310)
hctz
countri
Atenolol,atenolol (005139,51079068420)
Renal artery atheroscler (4401)
Iron defic anemia nos (2809)
norvasc
hydrochlorothiazid
Labetalol hcl (005097,85036207)
labetolol
Malignant hypertension (4010)
Peripheral vascular disorders w/o cc/mcc (MS,301)
Hydralazine hcl,hydralazine hcl (000284,51079007420)
Hydralazine hcl,hydralazine hcl (000286,51079007520)
Losartan potassium,losartan potassium (023382,6095228)
Labetalol hcl,labetalol hcl (005098,60976034647)
Peripheral & other vascular disorders (APR,1972)
Clonidine hcl,clonidine hcl (000346,51079029920)

Topic 27

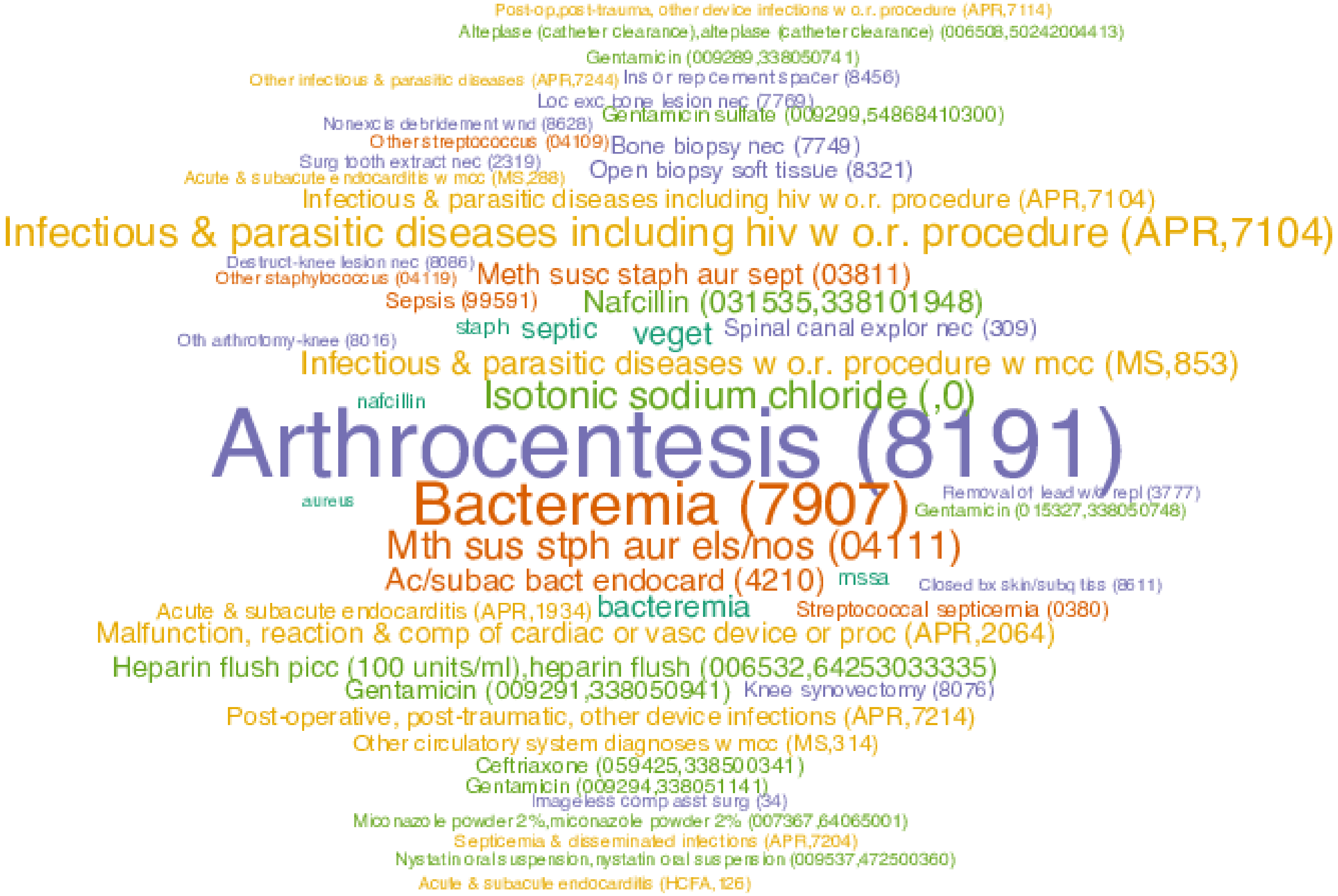

Post-op,post-trauma, other device infections w o.r. procedure (APR,7114)
Alteplase (catheter clearance),alteplase (catheter clearance) (006508,50242004413)
Gentamicin (009289,338050741)
Other infectious & parasitic diseases (APR,7244)
Ins or repcement spacer (8456)
Loc exc bone lesion nec (7769)
Gentamicin sulfate (009299,54868410300)
Nonexcis debridement wnd (8628)
Other streptococcus (04109)
Bone biopsy nec (7749)
Surg tooth extract nec (2319)
Acute & subacute endocarditis w mcc (MS,288)
Open biopsy soft tissue (8321)
Infectious & parasitic diseases including hiv w o.r. procedure (APR,7104)
Infectious & parasitic diseases including hiv w o.r. procedure (APR,7104)
Destruct-knee lesion nec (8086)
Other staphylococcus (04119)
Meth susc staph aur sept (03811)
Sepsis (99591)
Nafcillin (031535,338101948)
staph
septic
veget
Spinal canal explor nec (309)
Oth arthrotomy-knee (8016)
Infectious & parasitic diseases w o.r. procedure w mcc (MS,853)
nafcillin
Isotonic sodium chloride (,0)
Arthrocentesis (8191)
Removal of lead w/o repl (3777)
aureus
Bacteremia (7907)
Gentamicin (015327,338050748)
Mth sus stph aur els/nos (04111)
Ac/subac bact endocard (4210)
mssa
Closed bx skin/subq tiss (8611)
Acute & subacute endocarditis (APR,1934)
bacteremia
Streptococcal septicemia (0380)
Malfunction, reaction & comp of cardiac or vasc device or proc (APR,2064)
Heparin flush picc (100 units/ml),heparin flush (006532,64253033335)
Gentamicin (009291,338050941)
Knee synovectomy (8076)
Post-operative, post-traumatic, other device infections (APR,7214)
Other circulatory system diagnoses w mcc (MS,314)
Ceftriaxone (059425,338500341)
Gentamicin (009294,338051141)
Imageless comp asst surg (34)
Miconazole powder 2%,miconazole powder 2% (007367,64065001)
Septicemia & disseminated infections (APR,7204)
Nystatin oral suspension,nystatin oral suspension (009537,472500360)
Acute & subacute endocarditis (HCFA,126)

Topic 28

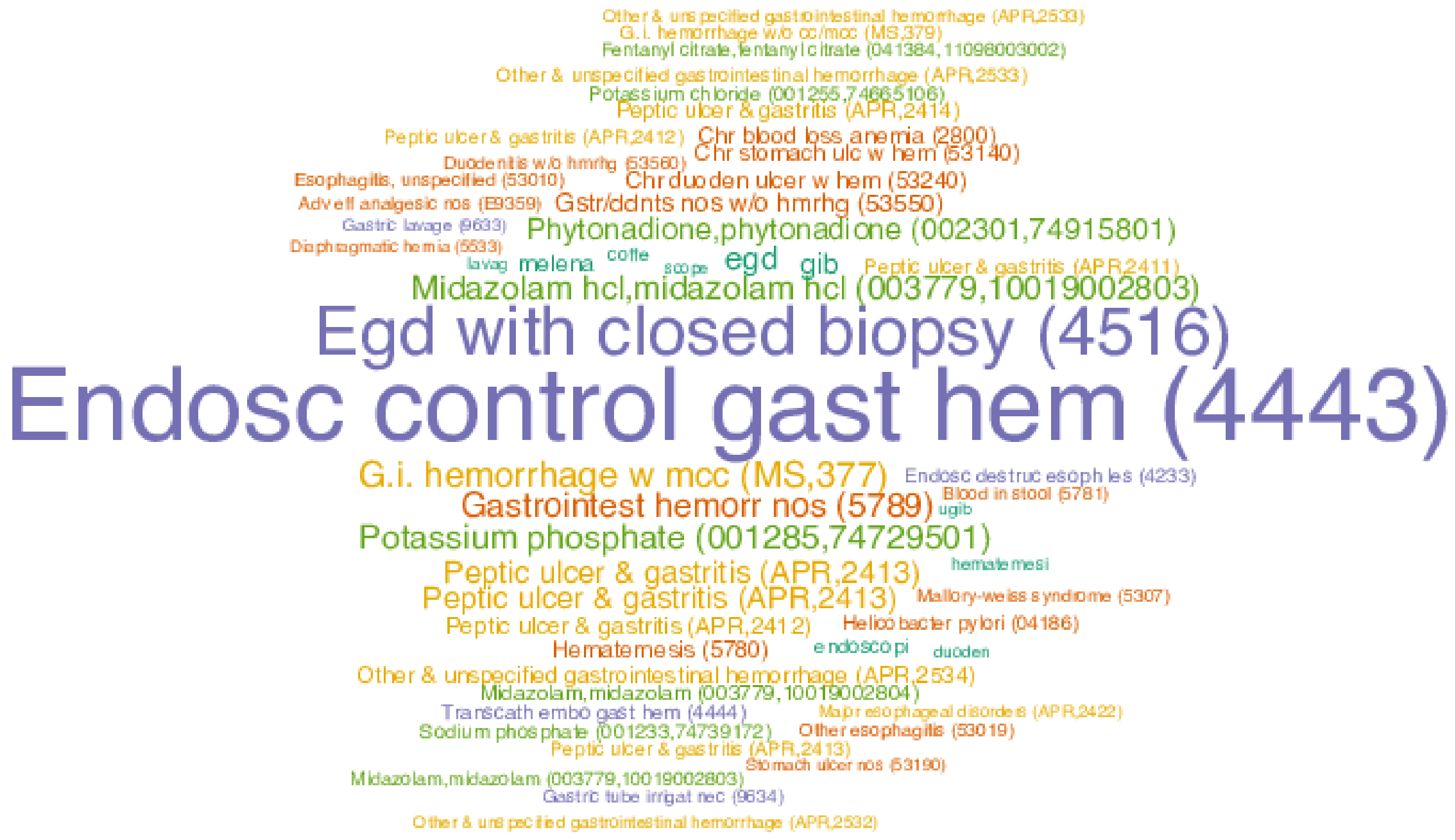
Other & unspecified gastrointestinal hemorrhage (APR,2533)
G.i. hemorrhage w/o cc/mcc (MS,379)
Fentanyl citrate,fentanyl citrate (041384,11098003002)
Other & unspecified gastrointestinal hemorrhage (APR,2533)
Potassium chloride (001255,74665106)
Peptic ulcer & gastritis (APR,2414)
Peptic ulcer & gastritis (APR,2412)
Chr blood loss anemia (2800)
Chr stomach ulc w hem (53140)
Duodenitis w/o hmrhg (53560)
Esophagitis, unspecified (53010)
Chr duoden ulcer w hem (53240)
Adv eff analgesic nos (E9359)
Gstr/ddnts nos w/o hmrhg (53550)
Gastric lavage (9633)
Phytonadione,phytonadione (002301,74915801)
Diaphragmatic hernia (5533)
lavag
melena
coffe
scope
egd
gib
Peptic ulcer & gastritis (APR,2411)
Midazolam hcl,midazolam hcl (003779,10019002803)
Egd with closed biopsy (4516)
Endosc control gast hem (4443)
G.i. hemorrhage w mcc (MS,377)
Endosc destruc esoph les (4233)
Blood in stool (5781)
Gastrointest hemorr nos (5789)
ugib
Potassium phosphate (001285,74729501)
Peptic ulcer & gastritis (APR,2413)
hematemesi
Peptic ulcer & gastritis (APR,2413)
Mallory-weiss syndrome (5307)
Peptic ulcer & gastritis (APR,2412)
Helicobacter pylori (04186)
Hematemesis (5780)
endoscopi
duoden
Other & unspecified gastrointestinal hemorrhage (APR,2534)
Midazolam,midazolam (003779,10019002804)
Transcath embo gast hem (4444)
Major esophageal disorders (APR,2422)
Sodium phosphate (001233,74739172)
Other esophagitis (53019)
Peptic ulcer & gastritis (APR,2413)
Stomach ulcer nos (53190)
Midazolam,midazolam (003779,10019002803)
Gastric tube irrigat nec (9634)
Other & unspecified gastrointestinal hemorrhage (APR,2532)

Topic 29

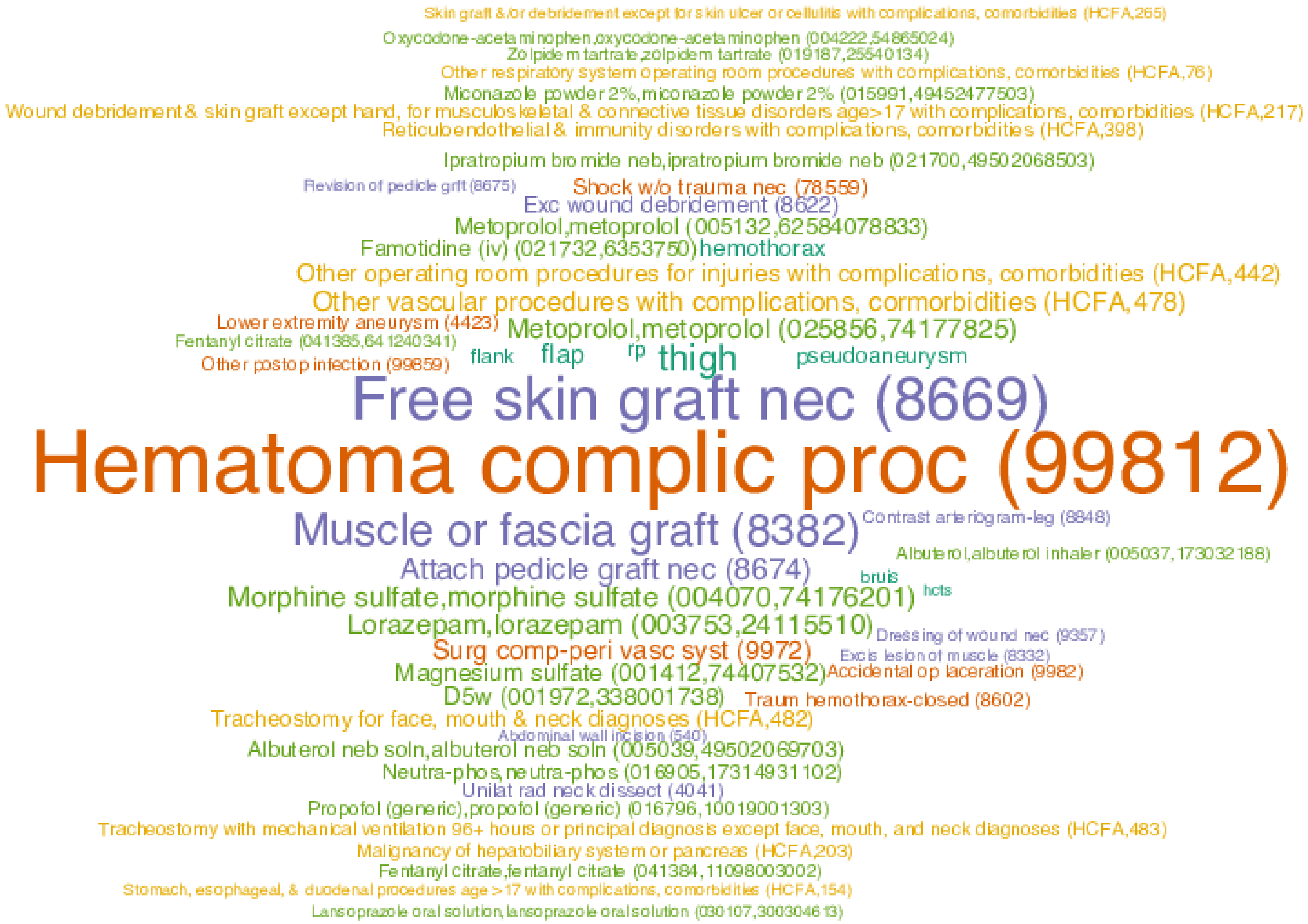
Skin graft &/or debridement except for skin ulcer or cellulitis with complications, comorbidities (HCFA,265)
Oxycodone-acetaminophen,oxycodone-acetaminophen (004222,54865024)
Zolpidem tartrate,zolpidem tartrate (019187,25540134)
Other respiratory system operating room procedures with complications, comorbidities (HCFA,76)
Miconazole powder 2%,miconazole powder 2% (015991,49452477503)
Wound debridement & skin graft except hand, for musculoskeletal & connective tissue disorders age>17 with complications, comorbidities (HCFA,217)
Reticuloendothelial & immunity disorders with complications, comorbidities (HCFA,398)
Ipratropium bromide neb,ipratropium bromide neb (021700,49502068503)
Revision of pedicle grft (8675)
Shock w/o trauma nec (78559)
Exc wound debridement (8622)
Metoprolol,metoprolol (005132,62584078833)
Famotidine (iv) (021732,6353750)
hemothorax
Other operating room procedures for injuries with complications, comorbidities (HCFA,442)
Other vascular procedures with complications, cormorbidities (HCFA,478)
Lower extremity aneurysm (4423)
Metoprolol,metoprolol (025856,74177825)
Fentanyl citrate (041385,641240341)
flank
flap
rp
thigh
pseudoaneurysm
Other postop infection (99859)
Free skin graft nec (8669)
Hematoma complic proc (99812)
Muscle or fascia graft (8382)
Contrast arteriogram-leg (8848)
Albuterol,albuterol inhaler (005037,173032188)
Attach pedicle graft nec (8674)
bruis
Morphine sulfate,morphine sulfate (004070,74176201)
hcts
Lorazepam,lorazepam (003753,24115510)
Dressing of wound nec (9357)
Surg comp-peri vasc syst (9972)
Excis lesion of muscle (8332)
Magnesium sulfate (001412,74407532)
Accidental op laceration (9982)
D5w (001972,338001738)
Traum hemothorax-closed (8602)
Tracheostomy for face, mouth & neck diagnoses (HCFA,482)
Abdominal wall incision (540)
Albuterol neb soln,albuterol neb soln (005039,49502069703)
Neutra-phos,neutra-phos (016905,17314931102)
Unilat rad neck dissect (4041)
Propofol (generic),propofol (generic) (016796,10019001303)
Tracheostomy with mechanical ventilation 96+ hours or principal diagnosis except face, mouth, and neck diagnoses (HCFA,483)
Malignancy of hepatobiliary system or pancreas (HCFA,203)
Fentanyl citrate,fentanyl citrate (041384,11098003002)
Stomach, esophageal, & duodenal procedures age>17 with complications, comorbidities (HCFA,154)
Lansoprazole oral solution,lansoprazole oral solution (030107,300304613)

Topic 30

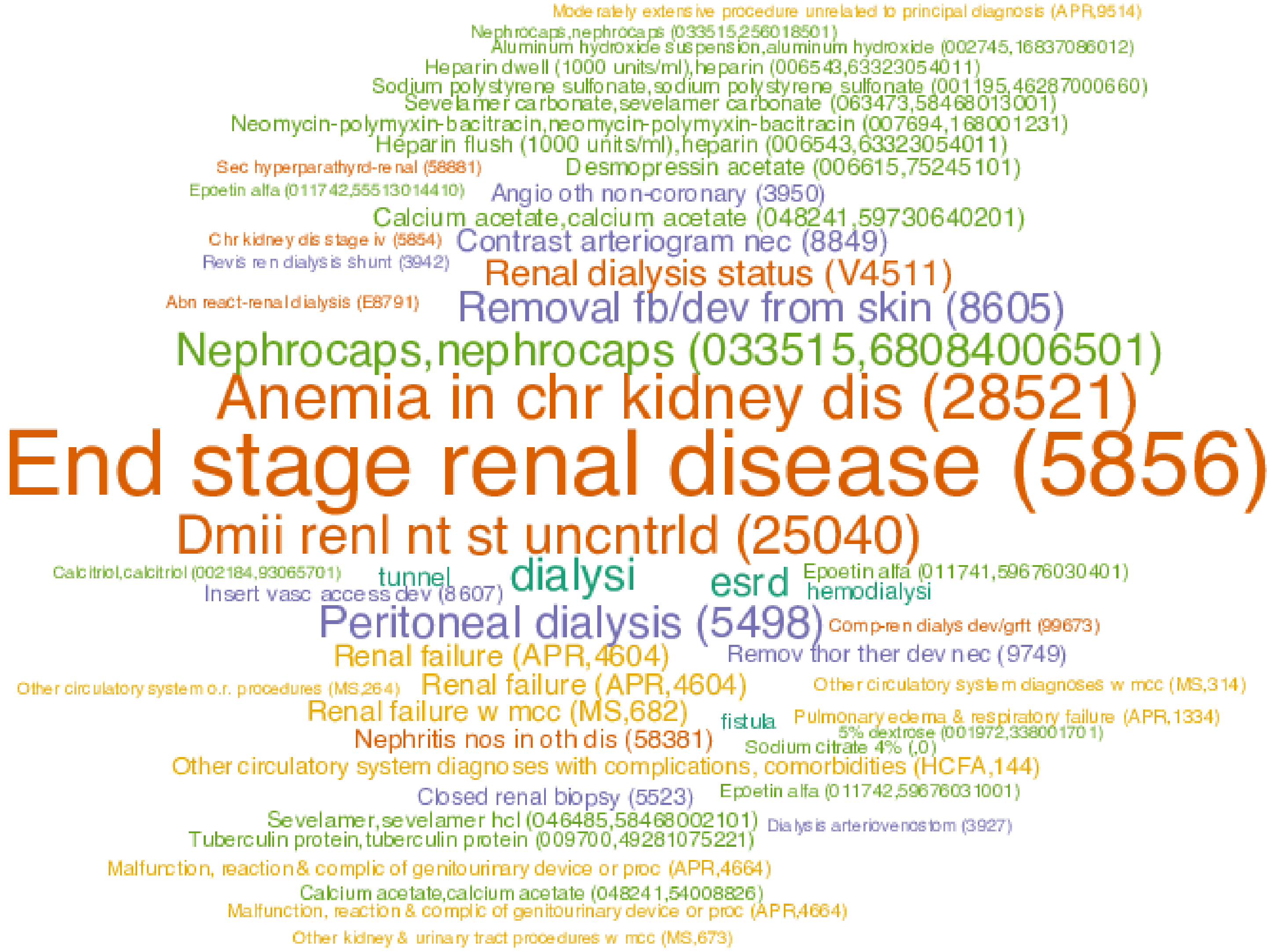
Moderately extensive procedure unrelated to principal diagnosis (APR,9514)
Nephrocaps,nephrocaps (033515,256018501)
Aluminum hydroxide suspension,aluminum hydroxide (002745,16837086012)
Heparin dwell (1000 units/ml),heparin (006543,63323054011)
Sodium polystyrene sulfonate,sodium polystyrene sulfonate (001195,46287000660)
Sevelamer carbonate,sevelamer carbonate (063473,58468013001)
Neomycin-polymyxin-bacitracin,neomycin-polymyxin-bacitracin (007694,168001231)
Heparin flush (1000 units/ml),heparin (006543,63323054011)
Sec hyperparathyrd-renal (58881)
Desmopressin acetate (006615,75245101)
Epoetin alfa (011742,55513014410)
Angio oth non-coronary (3950)
Calcium acetate,calcium acetate (048241,59730640201)
Chr kidney dis stage iv (5854)
Contrast arteriogram nec (8849)
Revis ren dialysis shunt (3942)
Renal dialysis status (V4511)
Abn react-renal dialysis (E8791)
Removal fb/dev from skin (8605)
Nephrocaps,nephrocaps (033515,68084006501)
Anemia in chr kidney dis (28521)
End stage renal disease (5856)
Dmii renl nt st uncntrld (25040)
Calcitriol,calcitriol (002184,93065701)
tunnel
dialysi
esrd
Epoetin alfa (011741,59676030401)
Insert vasc access dev (8607)
hemodialysi
Peritoneal dialysis (5498)
Comp-ren dialys dev/grft (99673)
Renal failure (APR,4604)
Remov thor ther dev nec (9749)
Other circulatory system o.r. procedures (MS,264)
Renal failure (APR,4604)
Other circulatory system diagnoses w mcc (MS,314)
Renal failure w mcc (MS,682)
fistula
Pulmonary edema & respiratory failure (APR,1334)
5% dextrose (001972,338001701)
Nephritis nos in oth dis (58381)
Sodium citrate 4% (,0)
Other circulatory system diagnoses with complications, comorbidities (HCFA,144)
Closed renal biopsy (5523)
Epoetin alfa (011742,59676031001)
Sevelamer,sevelamer hcl (046485,58468002101)
Dialysis arteriovenostom (3927)
Tuberculin protein,tuberculin protein (009700,49281075221)
Malfunction, reaction & complic of genitourinary device or proc (APR,4664)
Calcium acetate,calcium acetate (048241,54008826)
Malfunction, reaction & complic of genitourinary device or proc (APR,4664)
Other kidney & urinary tract procedures w mcc (MS,673)

Topic 31

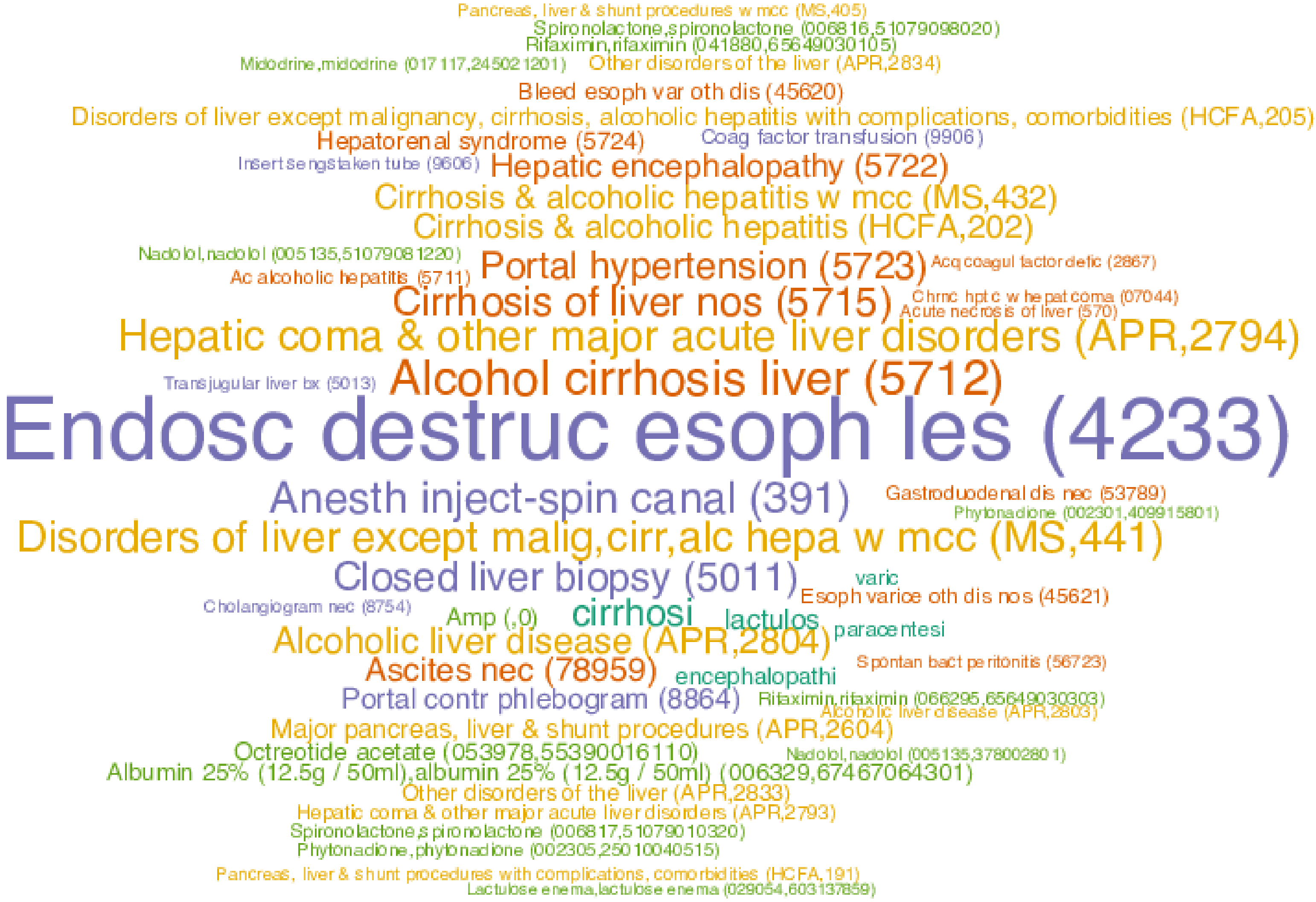

Pancreas, liver & shunt procedures w mcc (MS,405)
Spironolactone,spironolactone (006816,51079098020)
Rifaximin,rifaximin (041880,65649030105)
Midodrine,midodrine (017117,245021201)
Other disorders of the liver (APR,2834)
Bleed esoph var oth dis (45620)
Disorders of liver except malignancy, cirrhosis, alcoholic hepatitis with complications, comorbidities (HCFA,205)
Hepatorenal syndrome (5724)
Coag factor transfusion (9906)
Insert sengstaken tube (9606)
Hepatic encephalopathy (5722)
Cirrhosis & alcoholic hepatitis w mcc (MS,432)
Cirrhosis & alcoholic hepatitis (HCFA,202)
Nadolol,nadolol (005135,51079081220)
Ac alcoholic hepatitis (5711)
Portal hypertension (5723)
Acq coagul factor defic (2867)
Cirrhosis of liver nos (5715)
Chrnc hptc w hepat coma (07044)
Acute necrosis of liver (570)
Hepatic coma & other major acute liver disorders (APR,2794)
Transjugular liver bx (5013)
Alcohol cirrhosis liver (5712)
Endosc destruc esoph les (4233)
Anesth inject-spin canal (391)
Gastroduodenal dis nec (53789)
Phytonadione (002301,409915801)
Disorders of liver except malig,cirr,alc hepa w mcc (MS,441)
Closed liver biopsy (5011)
varic
Esoph varice oth dis nos (45621)
Cholangiogram nec (8754)
Amp (,0)
cirrhosi
lactulos
paracentesi
Alcoholic liver disease (APR,2804)
Ascites nec (78959)
Spontan bact peritonitis (56723)
encephalopathi
Portal contr phlebogram (8864)
Rifaximin,rifaximin (066295,65649030303)
Alcoholic liver disease (APR,2803)
Major pancreas, liver & shunt procedures (APR,2604)
Octreotide acetate (053978,55390016110)
Nadolol,nadolol (005135,378002801)
Albumin 25% (12.5g / 50ml),albumin 25% (12.5g / 50ml) (006329,67467064301)
Other disorders of the liver (APR,2833)
Hepatic coma & other major acute liver disorders (APR,2793)
Spironolactone,spironolactone (006817,51079010320)
Phytonadione,phytonadione (002305,25010040515)
Pancreas, liver & shunt procedures with complications, comorbidities (HCFA,191)
Lactulose enema,lactulose enema (029054,603137859)

Topic 32

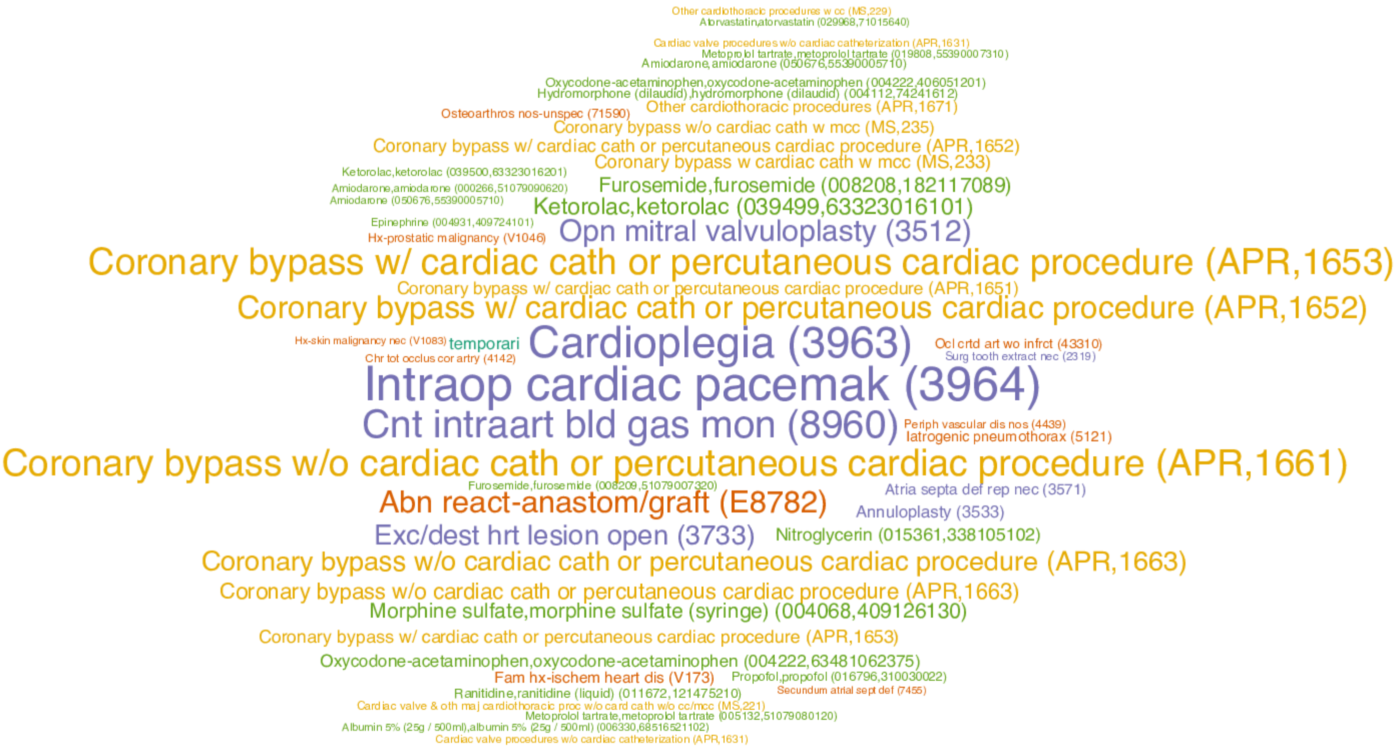
Other cardiothoracic procedures w cc (MS,229)
Atorvastatin,atorvastatin (029968,71015640)
Cardiac valve procedures w/o cardiac catheterization (APR,1631)
Metoprolol tartrate,metoprolol tartrate (019808,55390007310)
Amiodarone,amiodarone (050676,55390005710)
Oxycodone-acetaminophen,oxycodone-acetaminophen (004222,406051201)
Hydromorphone (dilaudid),hydromorphone (dilaudid) (004112,74241612)
Osteoarthros nos-unspec (71590)
Other cardiothoracic procedures (APR,1671)
Coronary bypass w/o cardiac cath w mcc (MS,235)
Coronary bypass w/ cardiac cath or percutaneous cardiac procedure (APR,1652)
Ketorolac,ketorolac (039500,63323016201)
Coronary bypass w cardiac cath w mcc (MS,233)
Amiodarone,amiodarone (000266,51079090620)
Furosemide,furosemide (008208,182117089)
Amiodarone (050676,55390005710)
Ketorolac,ketorolac (039499,63323016101)
Epinephrine (004931,409724101)
Opn mitral valvuloplasty (3512)
Hx-prostatic malignancy (V1046)
Coronary bypass w/ cardiac cath or percutaneous cardiac procedure (APR,1653)
Coronary bypass w/ cardiac cath or percutaneous cardiac procedure (APR,1651)
Coronary bypass w/ cardiac cath or percutaneous cardiac procedure (APR,1652)
Hx-skin malignancy nec (V1083)
temporari
Cardioplegia (3963)
Ocl crtd art wo infrct (43310)
Chr tot occlus cor artry (4142)
Surg tooth extract nec (2319)
Intraop cardiac pacemak (3964)
Cnt intraart bld gas mon (8960)
Periph vascular dis nos (4439)
Iatrogenic pneumothorax (5121)
Coronary bypass w/o cardiac cath or percutaneous cardiac procedure (APR,1661)
Furosemide,furosemide (008209,51079007320)
Atria septa def rep nec (3571)
Abn react-anastom/graft (E8782)
Annuloplasty (3533)
Exc/dest hrt lesion open (3733)
Nitroglycerin (015361,338105102)
Coronary bypass w/o cardiac cath or percutaneous cardiac procedure (APR,1663)
Coronary bypass w/o cardiac cath or percutaneous cardiac procedure (APR,1663)
Morphine sulfate,morphine sulfate (syringe) (004068,409126130)
Coronary bypass w/ cardiac cath or percutaneous cardiac procedure (APR,1653)
Oxycodone-acetaminophen,oxycodone-acetaminophen (004222,63481062375)
Fam hx-ischem heart dis (V173)
Propofol,propofol (016796,310030022)
Ranitidine,ranitidine (liquid) (011672,121475210)
Secundum atrial sept def (7455)
Cardiac valve & oth maj cardiothoracic proc w/o card cath w/o cc/mcc (MS,221)
Metoprolol tartrate,metoprolol tartrate (005132,51079080120)
Albumin 5% (25g / 500ml),albumin 5% (25g / 500ml) (006330,68516521102)
Cardiac valve procedures w/o cardiac catheterization (APR,1631)

Topic 33

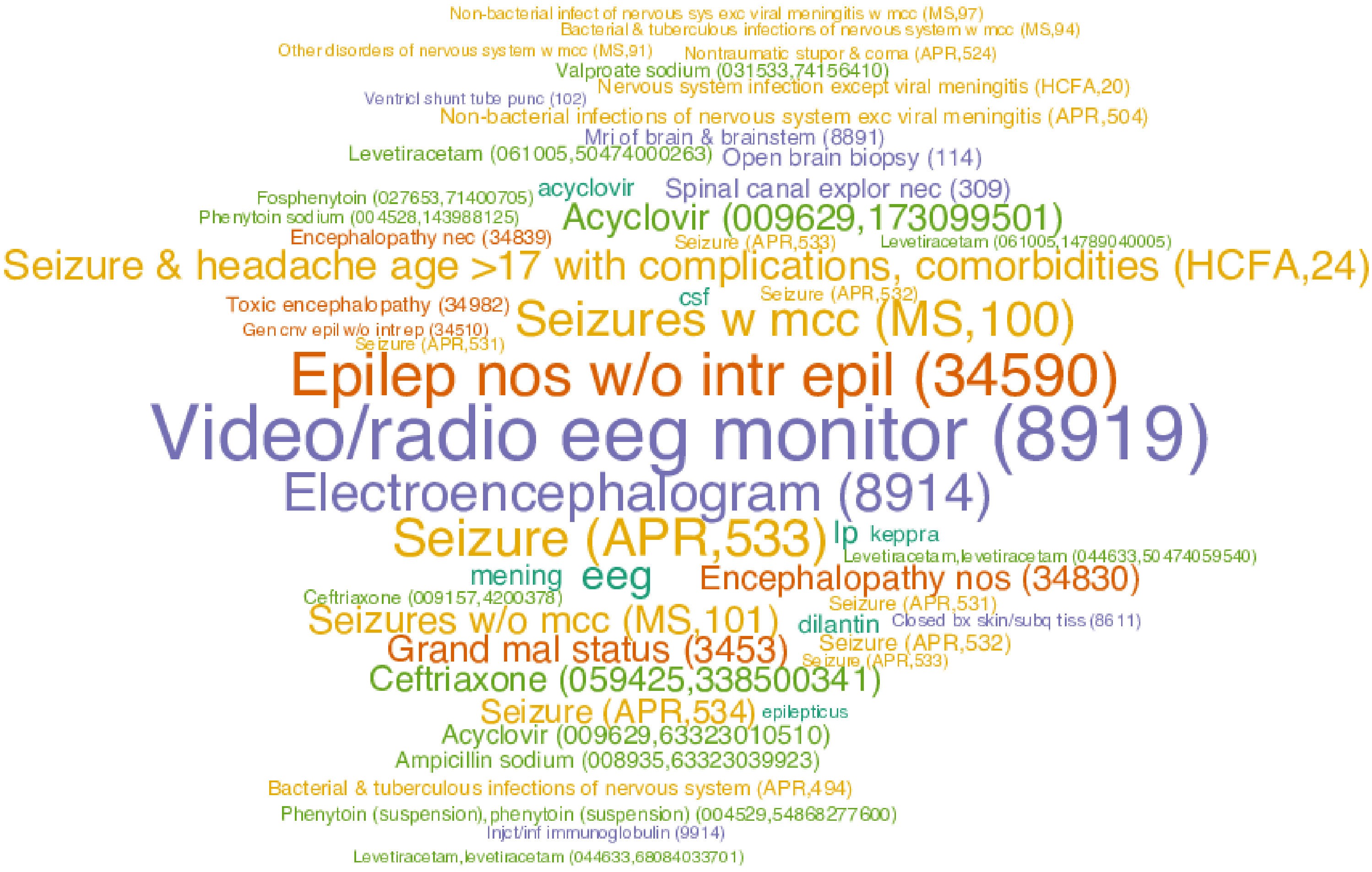
Non-bacterial infect of nervous sys exc viral meningitis w mcc (MS,97)
Bacterial & tuberculous infections of nervous system w mcc (MS,94)
Other disorders of nervous system w mcc (MS,91)
Nontraumatic stupor & coma (APR,524)
Valproate sodium (031533,74156410)
Ventricl shunt tube punc (102)
Nervous system infection except viral meningitis (HCFA,20)
Non-bacterial infections of nervous system exc viral meningitis (APR,504)
Mri of brain & brainstem (8891)
Levetiracetam (061005,50474000263)
Open brain biopsy (114)
Fosphenytoin (027653,71400705)
acyclovir
Spinal canal explor nec (309)
Phenytoin sodium (004528,143988125)
Acyclovir (009629,173099501)
Encephalopathy nec (34839)
Seizure (APR,533)
Levetiracetam (061005,14789040005)
Seizure & headache age >17 with complications, comorbidities (HCFA,24)
Toxic encephalopathy (34982)
csf
Seizure (APR,532)
Gen cnv epil w/o intr ep (34510)
Seizures w mcc (MS,100)
Seizure (APR,531)
Epilep nos w/o intr epil (34590)
Video/radio eeg monitor (8919)
Electroencephalogram (8914)
Seizure (APR,533)
lp
keppra
Levetiracetam,levetiracetam (044633,50474059540)
mening
eeg
Encephalopathy nos (34830)
Ceftriaxone (009157,4200378)
Seizure (APR,531)
Seizures w/o mcc (MS,101)
dilantin
Closed bx skin/subq tiss (8611)
Grand mal status (3453)
Seizure (APR,532)
Seizure (APR,533)
Ceftriaxone (059425,338500341)
Seizure (APR,534)
epilepticus
Acyclovir (009629,63323010510)
Ampicillin sodium (008935,63323039923)
Bacterial & tuberculous infections of nervous system (APR,494)
Phenytoin (suspension),phenytoin (suspension) (004529,54868277600)
Injct/inf immunoglobulin (9914)
Levetiracetam,levetiracetam (044633,68084033701)

Topic 34

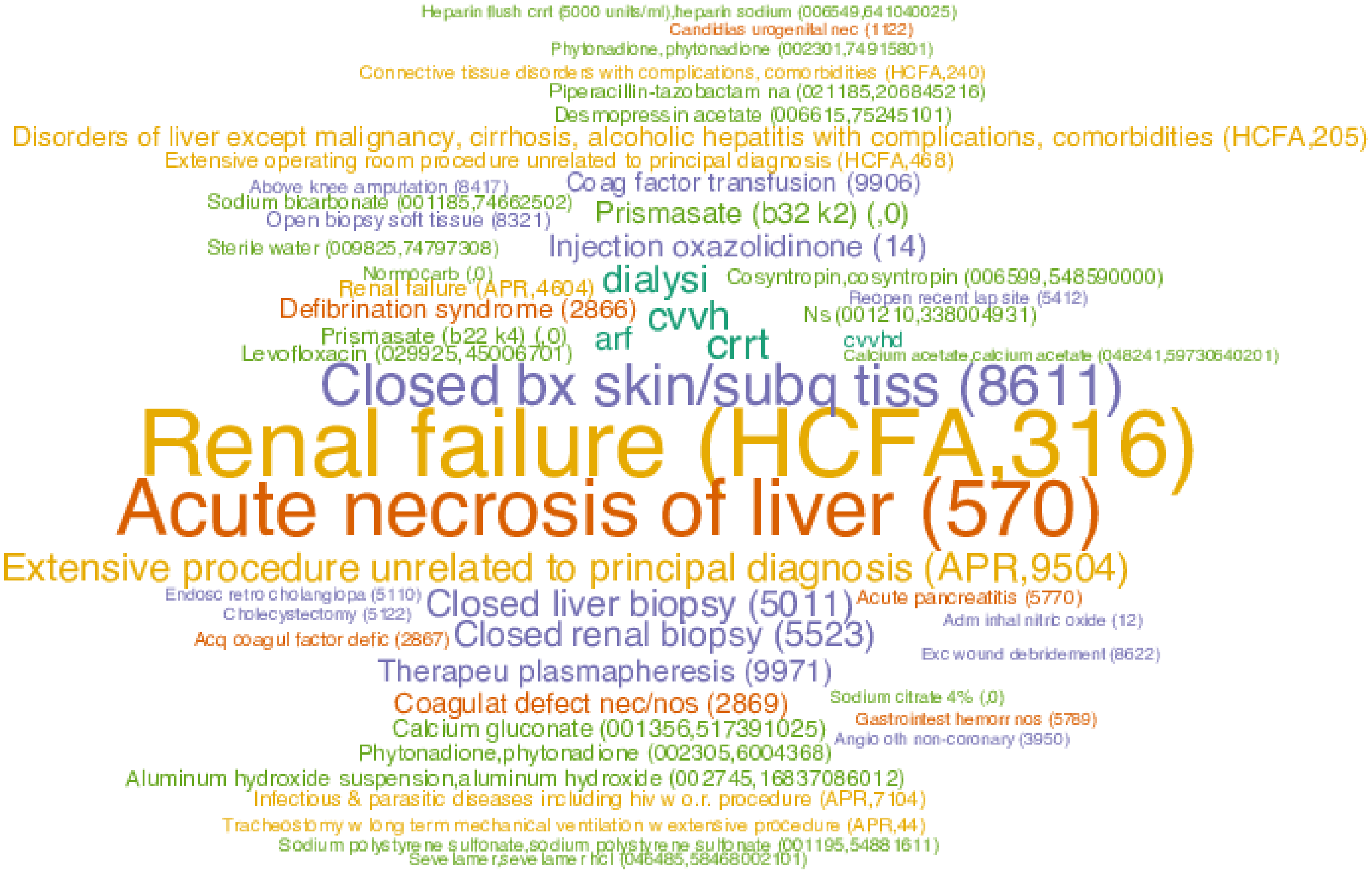
Heparin flush crrt (5000 units/ml),heparin sodium (006549,64104025)
Candidias urogenital nec (1122)
Phytonadione,phytonadione (002301,74915801)
Connective tissue disorders with complications, comorbidities (HCFA,240)
Piperacillin-tazobactam na (021185,206845216)
Desmopressin acetate (006615,75245101)
Disorders of liver except malignancy, cirrhosis, alcoholic hepatitis with complications, comorbidities (HCFA,205)
Extensive operating room procedure unrelated to principal diagnosis (HCFA,468)
Above knee amputation (8417)
Coag factor transfusion (9906)
Sodium bicarbonate (001185,74662502)
Open biopsy soft tissue (8321)
Prismasate (b32 k2) (,0)
Sterile water (009825,74797308)
Injection oxazolidinone (14)
Normocarb (,0)
Renal failure (APR,4604)
dialysi
Cosyntropin,cosyntropin (006599,548590000)
Reopen recent lap site (5412)
Defibrination syndrome (2866)
cvvh
Ns (001210,338004931)
Prismasate (b22 k4) (,0)
arf
crrt
cvvhd
Levofloxacin (029925,45006701)
Calcium acetate,calcium acetate (048241,597300640201)
Closed bx skin/subq tiss (8611)
Renal failure (HCFA,316)
Acute necrosis of liver (570)
Extensive procedure unrelated to principal diagnosis (APR,9504)
Endosc retro cholangiopa (5110)
Closed liver biopsy (5011)
Acute pancreatitis (5770)
Cholecystectomy (5122)
Adm inhal nitric oxide (12)
Acq coagul factor defic (2867)
Closed renal biopsy (5523)
Exc wound debridement (8622)
Therapeu plasmapheresis (9971)
Coagulat defect nec/nos (2869)
Sodium citrate 4% (,0)
Gastrointest hemorr nos (5789)
Calcium gluconate (001356,517391025)
Angio oth non-coronary (3950)
Phytonadione,phytonadione (002305,6004368)
Aluminum hydroxide suspension,aluminum hydroxide (002745,16837086012)
Infectious & parasitic diseases including hiv w o.r. procedure (APR,7104)
Tracheostomy w long term mechanical ventilation w extensive procedure (APR,44)
Sodium polystyrene sulfonate,sodium polystyrene sulfonate (001195,54881611)
Sevelamer,sevelamer hcl (046485,58468002101)

Topic 35

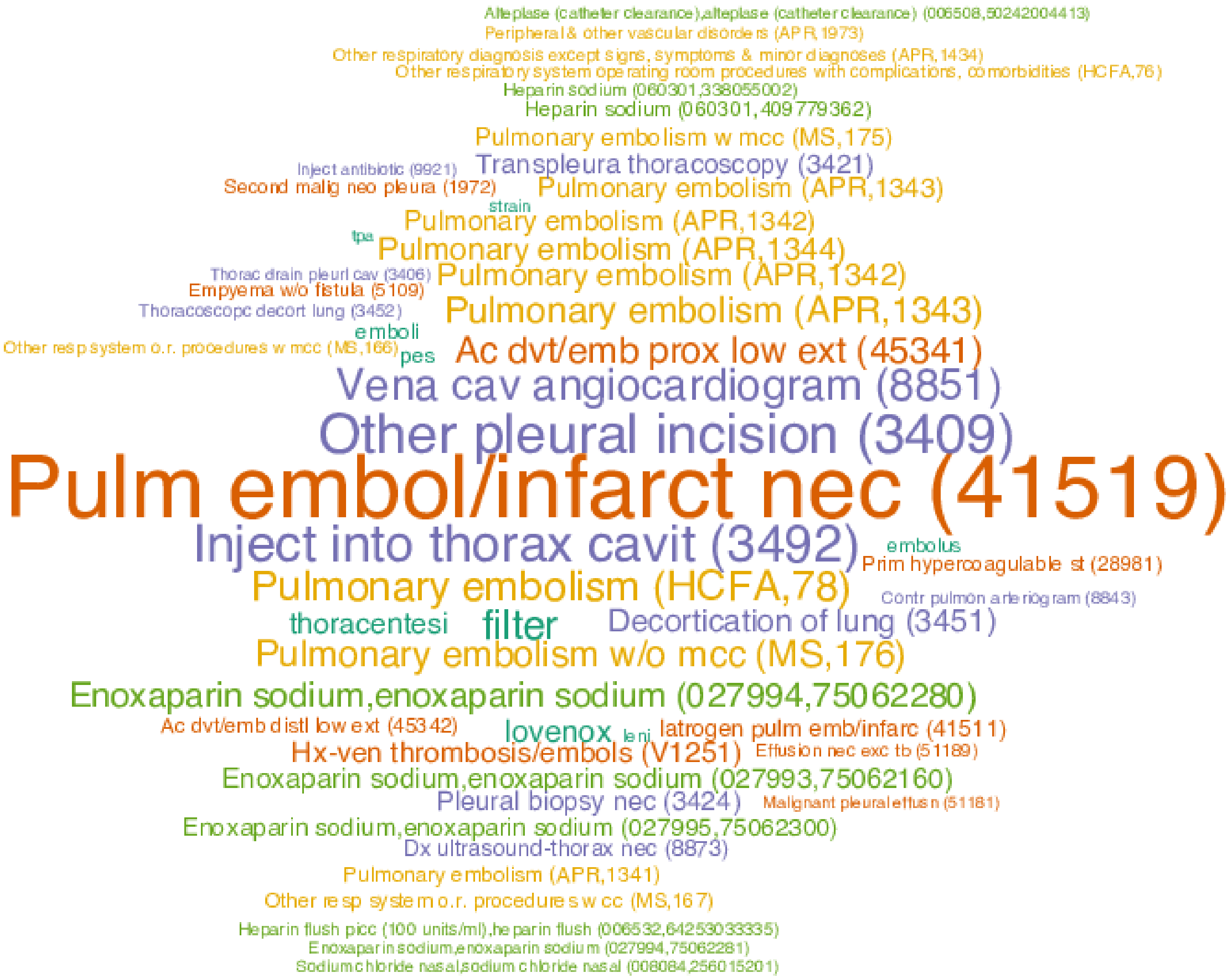

Alteplase (catheter clearance),alteplase (catheter clearance) (006508,50242004413)
Peripheral & other vascular disorders (APR,1973)
Other respiratory diagnosis except signs, symptoms & minor diagnoses (APR,1434)
Other respiratory system operating room procedures with complications, comorbidities (HCFA,76)
Heparin sodium (060301,338055002)
Heparin sodium (060301,409779362)
Pulmonary embolism w mcc (MS,175)
Inject antibiotic (9921)
Transpleura thoracoscopy (3421)
Second malig neo pleura (1972)
Pulmonary embolism (APR,1343)
strain
Pulmonary embolism (APR,1342)
tpa
Pulmonary embolism (APR,1344)
Thorac drain pleurl cav (3406)
Pulmonary embolism (APR,1342)
Empyema w/o fistula (5109)
Thoracoscopc decort lung (3452)
Pulmonary embolism (APR,1343)
emboli
Other resp system o.r. procedures w mcc (MS,166)
pes
Ac dvt/emb prox low ext (45341)
Vena cav angiocardiogram (8851)
Other pleural incision (3409)
Pulm embol/infarct nec (41519)
Inject into thorax cavit (3492)
embolus
Prim hypercoagulable st (28981)
Pulmonary embolism (HCFA,78)
Contr pulmon arteriogram (8843)
thoracentesi
filter
Decortication of lung (3451)
Pulmonary embolism w/o mcc (MS,176)
Enoxaparin sodium,enoxaparin sodium (027994,75062280)
Ac dvt/emb distl low ext (45342)
lovenox
leni
Iatrogen pulm emb/infarc (41511)
Hx-ven thrombosis/embols (V1251)
Effusion nec exc tb (51189)
Enoxaparin sodium,enoxaparin sodium (027993,75062160)
Pleural biopsy nec (3424)
Malignant pleural effusn (51181)
Enoxaparin sodium,enoxaparin sodium (027995,75062300)
Dx ultrasound-thorax nec (8873)
Pulmonary embolism (APR,1341)
Other resp system o.r. procedures w cc (MS,167)
Heparin flush picc (100 units/ml),heparin flush (006532,64253033335)
Enoxaparin sodium,enoxaparin sodium (027994,75062281)
Sodium chloride nasal,sodium chloride nasal (008084,25601520 1)

Topic 36

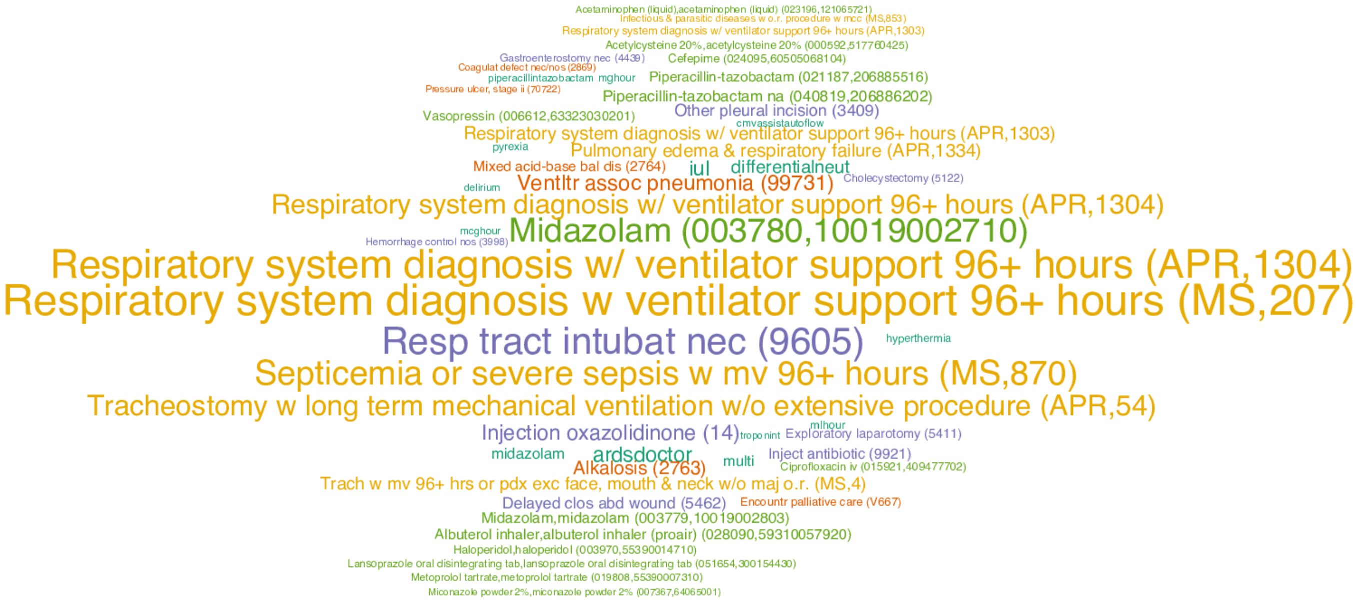

Acetaminophen (liquid),acetaminophen (liquid) (023196,121065721)
Infectious & parasitic diseases w o.r. procedure w mcc (MS,853)
Respiratory system diagnosis w/ ventilator support 96+ hours (APR,1303)
Acetylcysteine 20%,acetylcysteine 20% (000592,517760425)
Gastroenterostomy nec (4439)
Cefepime (024095,60505068104)
Coagulat defect nec/nos (2869)
piperacillintazobactam
mghour
Piperacillin-tazobactam (021187,206885516)
Pressure ulcer, stage ii (70722)
Piperacillin-tazobactam na (040819,206886202)
Vasopressin (006612,63323030201)
Other pleural incision (3409)
cmvassistautoflow
Respiratory system diagnosis w/ ventilator support 96+ hours (APR,1303)
pyrexia
Pulmonary edema & respiratory failure (APR,1334)
Mixed acid-base bal dis (2764)
iul
differentialneut
Cholecystectomy (5122)
delirium
Ventltr assoc pneumonia (99731)
Respiratory system diagnosis w/ ventilator support 96+ hours (APR,1304)
mcghour
Hemorrhage control nos (3998)
Midazolam (003780,10019002710)
Respiratory system diagnosis w/ ventilator support 96+ hours (APR,1304)
Respiratory system diagnosis w ventilator support 96+ hours (MS,207)
Resp tract intubat nec (9605)
hyperthermia
Septicemia or severe sepsis w mv 96+ hours (MS,870)
Tracheostomy w long term mechanical ventilation w/o extensive procedure (APR,54)
Injection oxazolidinone (14)
troponint
mlhour
Exploratory laparotomy (5411)
midazolam
ardsdoctor
multi
Inject antibiotic (9921)
Alkalosis (2763)
Ciprofloxacin iv (015921,409477702)
Trach w mv 96+ hrs or pdx exc face, mouth & neck w/o maj o.r. (MS,4)
Delayed clos abd wound (5462)
Encountr palliative care (V667)
Midazolam,midazolam (003779,10019002803)
Albuterol inhaler,albuterol inhaler (proair) (028090,59310057920)
Haloperidol,haloperidol (003970,55390014710)
Lansoprazole oral disintegrating tab,lansoprazole oral disintegrating tab (051654,300154430)
Metoprolol tartrate,metoprolol tartrate (019808,55390007310)
Miconazole powder 2%,miconazole powder 2% (007367,64065001)

Topic 37

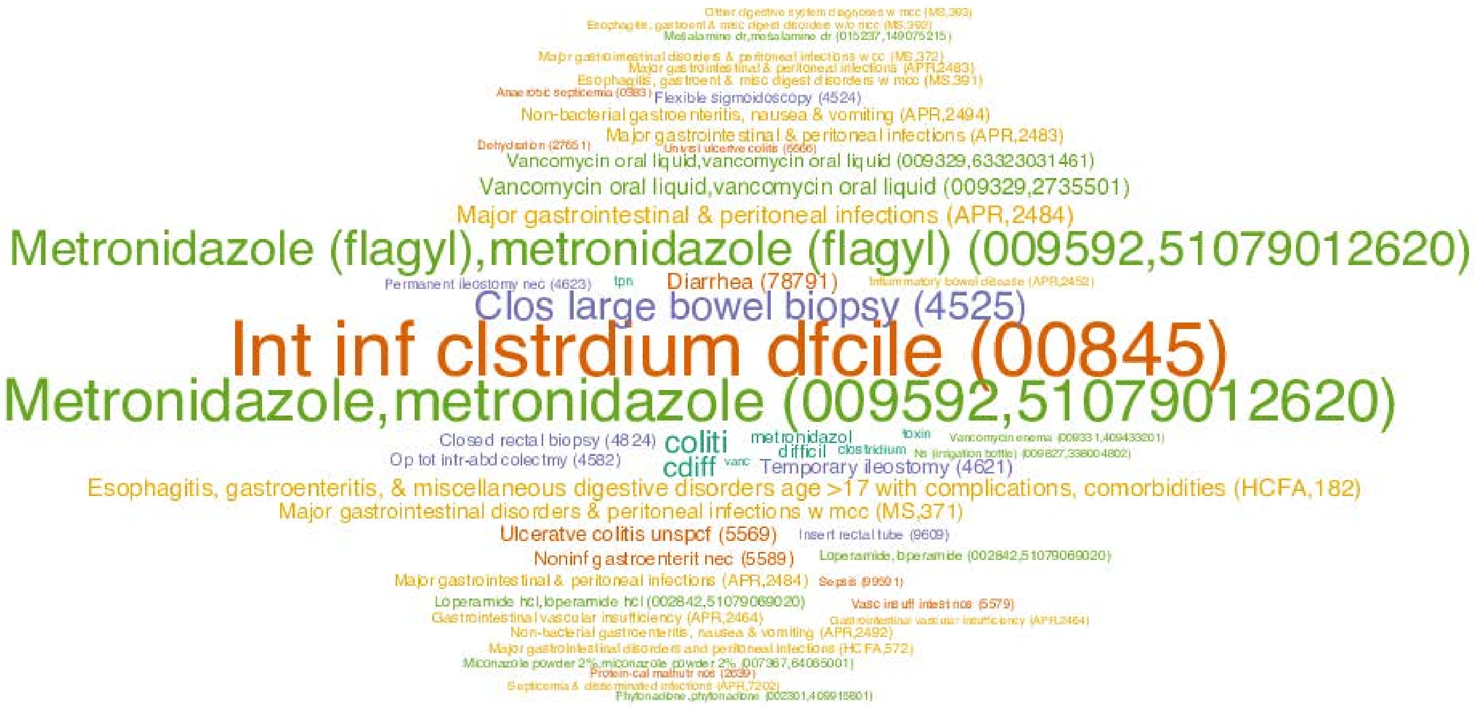
Metronidazole (flagyl),metronidazole (flagyl) (009592,51079012620)
Clos large bowel biopsy (4525)
Int inf clstrdium dfcile (00845)
Metronidazole,metronidazole (009592,51079012620)
Diarrhea (78791)
Major gastrointestinal & peritoneal infections (APR,2484)
Vancomycin oral liquid,vancomycin oral liquid (009329,2735501)
Vancomycin oral liquid,vancomycin oral liquid (009329,63323031461)
Major gastrointestinal & peritoneal infections (APR,2483)
Non-bacterial gastroenteritis, nausea & vomiting (APR,2494)
Flexible sigmoidoscopy (4524)
Esophagitis, gastroenteritis, & miscellaneous digestive disorders age >17 with complications, comorbidities (HCFA,182)
Major gastrointestinal disorders & peritoneal infections w mcc (MS,371)
Ulceratve colitis unspcf (5569)
Noninf gastroenterit nec (5589)
Closed rectal biopsy (4824)
Op tot intr-abd colectmy (4582)
coliti
cdiff
metronidazol
difficil
Temporary ileostomy (4621)
Permanent ileostomy nec (4623)
Major gastrointestinal & peritoneal infections (APR,2484)
Loperamide hcl,loperamide hcl (002842,51079069020)
Gastrointestinal vascular insufficiency (APR,2464)
Non-bacterial gastroenteritis, nausea & vomiting (APR,2492)
Insert rectal tube (9609)
Loperamide,loperamide (002842,51079069020)

Topic 38

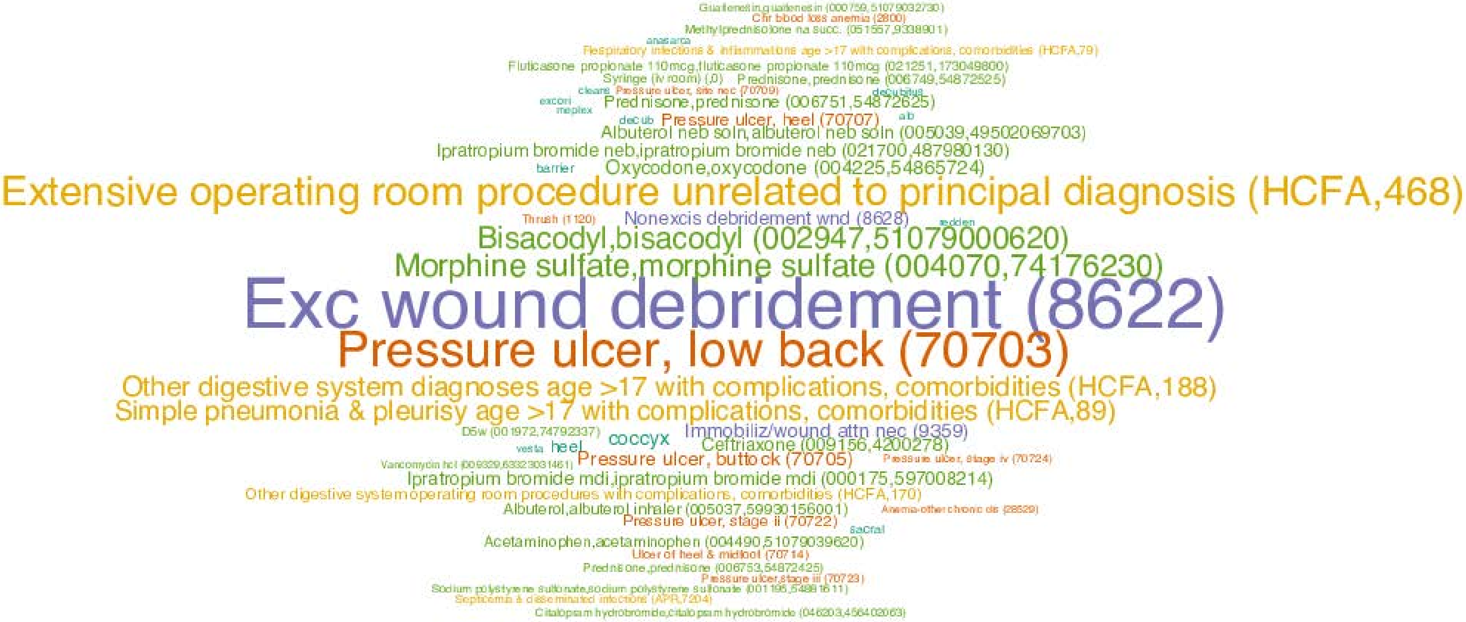

Methylprednisolone na succ. (051557,9338901)
anasarca
Respiratory infections & inflammations age >17 with complications, comorbidities (HCFA,79)
Fluticasone propionate 110mcg,fluticasone propionate 110mcg (021251,173049800)
Syringe (iv room) (,0)
Prednisone,prednisone (006749,54872525)
Pressure ulcer, site nec (70709)
decubitus
excori
mepilex
Prednisone,prednisone (006751,54872625)
decub
Pressure ulcer, heel (70707)
Albuterol neb soln,albuterol neb soln (005039,49502069703)
Ipratropium bromide neb,ipratropium bromide neb (021700,487980130)
barrier
Oxycodone,oxycodone (004225,54865724)
Extensive operating room procedure unrelated to principal diagnosis (HCFA,468)
Thrush (1120)
Nonexcis debridement wnd (8628)
Bisacodyl,bisacodyl (002947,51079000620)
Morphine sulfate,morphine sulfate (004070,74176230)
Exc wound debridement (8622)
Pressure ulcer, low back (70703)
Other digestive system diagnoses age >17 with complications, comorbidities (HCFA,188)
Simple pneumonia & pleurisy age >17 with complications, comorbidities (HCFA,89)
D5w (001972,74792337)
coccyx
Immobiliz/wound attn nec (9359)
vesta
heel
Ceftriaxone (009156,4200278)
Pressure ulcer, buttock (70705)
Pressure ulcer, stage iv (70724)
Ipratropium bromide mdi,ipratropium bromide mdi (000175,597008214)
Other digestive system operating room procedures with complications, comorbidities (HCFA,170)
Albuterol,albuterol inhaler (005037,59930156001)
Pressure ulcer, stage ii (70722)
sacral
Acetaminophen,acetaminophen (004490,51079039620)
Ulcer of heel & midfoot (70714)
Prednisone,prednisone (006753,54872425)
Pressure ulcer,stage iii (70723)
Sodium polystyrene sulfonate,sodium polystyrene sulfonate (001195,54881611)
Septicemia & disseminated infections (APR,7204)
Citalopram hydrobromide,citalopram hydrobromide (046203,456402063)

Topic 39

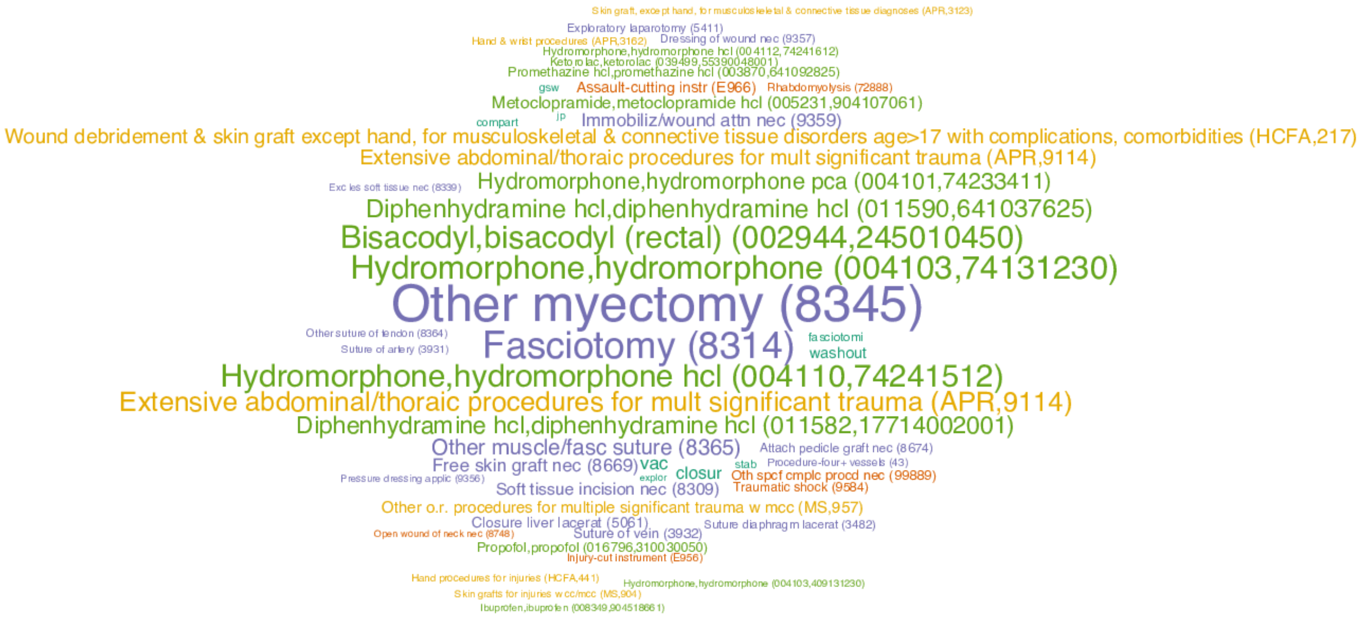
Skin graft, except hand, for musculoskeletal & connective tissue diagnoses (APR,3123)
Exploratory laparotomy (5411)
Hand & wrist procedures (APR,3162)
Dressing of wound nec (9357)
Hydromorphone,hydromorphone hcl (004112,74241612)
Ketorolac,ketorolac (039499,55390048001)
Promethazine hcl,promethazine hcl (003870,641092825)
gsw
Assault-cutting instr (E966)
Rhabdomyolysis (72888)
Metoclopramide,metoclopramide hcl (005231,904107061)
compart
jp
Immobiliz/wound attn nec (9359)
Wound debridement & skin graft except hand, for musculoskeletal & connective tissue disorders age>17 with complications, comorbidities (HCFA,217)
Extensive abdominal/thoracic procedures for mult significant trauma (APR,9114)
Exc les soft tissue nec (8339)
Hydromorphone,hydromorphone pca (004101,74233411)
Diphenhydramine hcl,diphenhydramine hcl (011590,641037625)
Bisacodyl,bisacodyl (rectal) (002944,245010450)
Hydromorphone,hydromorphone (004103,74131230)
Other myectomy (8345)
Other suture of tendon (8364)
Suture of artery (3931)
Fasciotomy (8314)
fasciotomi
washout
Hydromorphone,hydromorphone hcl (004110,74241512)
Extensive abdominal/thoracic procedures for mult significant trauma (APR,9114)
Diphenhydramine hcl,diphenhydramine hcl (011582,17714002001)
Other muscle/fasc suture (8365)
Attach pedicle graft nec (8674)
Free skin graft nec (8669)
vac
stab
Procedure-four+ vessels (43)
Pressure dressing applic (9356)
explor
closur
Oth spcf cmplc procd nec (99889)
Soft tissue incision nec (8309)
Traumatic shock (9584)
Other o.r. procedures for multiple significant trauma w mcc (MS,957)
Closure liver lacerat (5061)
Suture diaphragm lacerat (3482)
Open wound of neck nec (8748)
Suture of vein (3932)
Propofol,propofol (016796,310030050)
Injury-cut instrument (E956)
Hand procedures for injuries (HCFA,441)
Hydromorphone,hydromorphone (004103,409131230)
Skin grafts for injuries w cc/mcc (MS,904)
Ibuprofen,ibuprofen (008349,904518661)

Topic 40

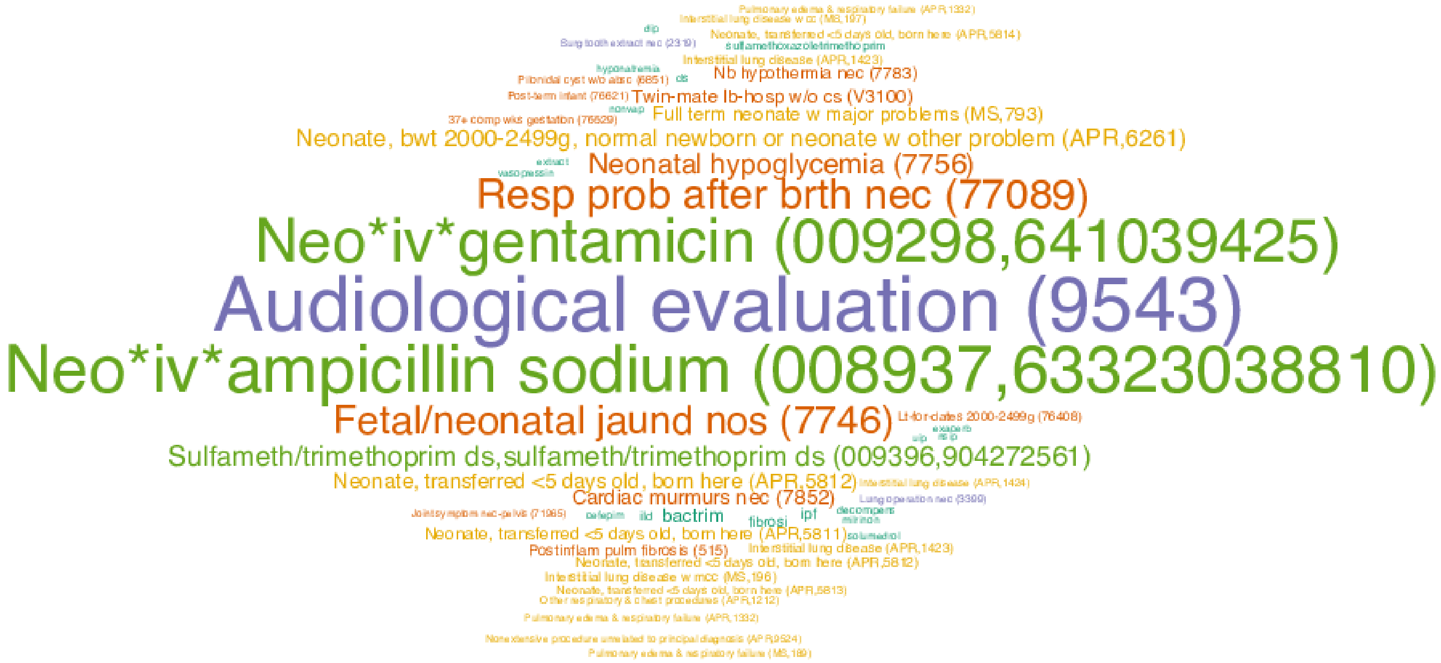
Neonate, bwt 2000-2499g, normal newborn or neonate w other problem (APR,6261)
Neonatal hypoglycemia (7756)
Resp prob after brth nec (77089)
Neo*iv*gentamicin (009298,641039425)
Audiological evaluation (9543)
Neo*iv*ampicillin sodium (008937,63323038810)
Fetal/neonatal jaund nos (7746)
Sulfameth/trimethoprim ds,sulfameth/trimethoprim ds (009396,904272561)
Neonate, transferred <5 days old, born here (APR,5812)
Cardiac murmurs nec (7852)
bactrim
Neonate, transferred <5 days old, born here (APR,5811)
Postinflam pulm fibrosis (515)
Full term neonate w major problems (MS,793)
Twin-mate lb-hosp w/o cs (V3100)
Nb hypothermia nec (7783)

Topic 41

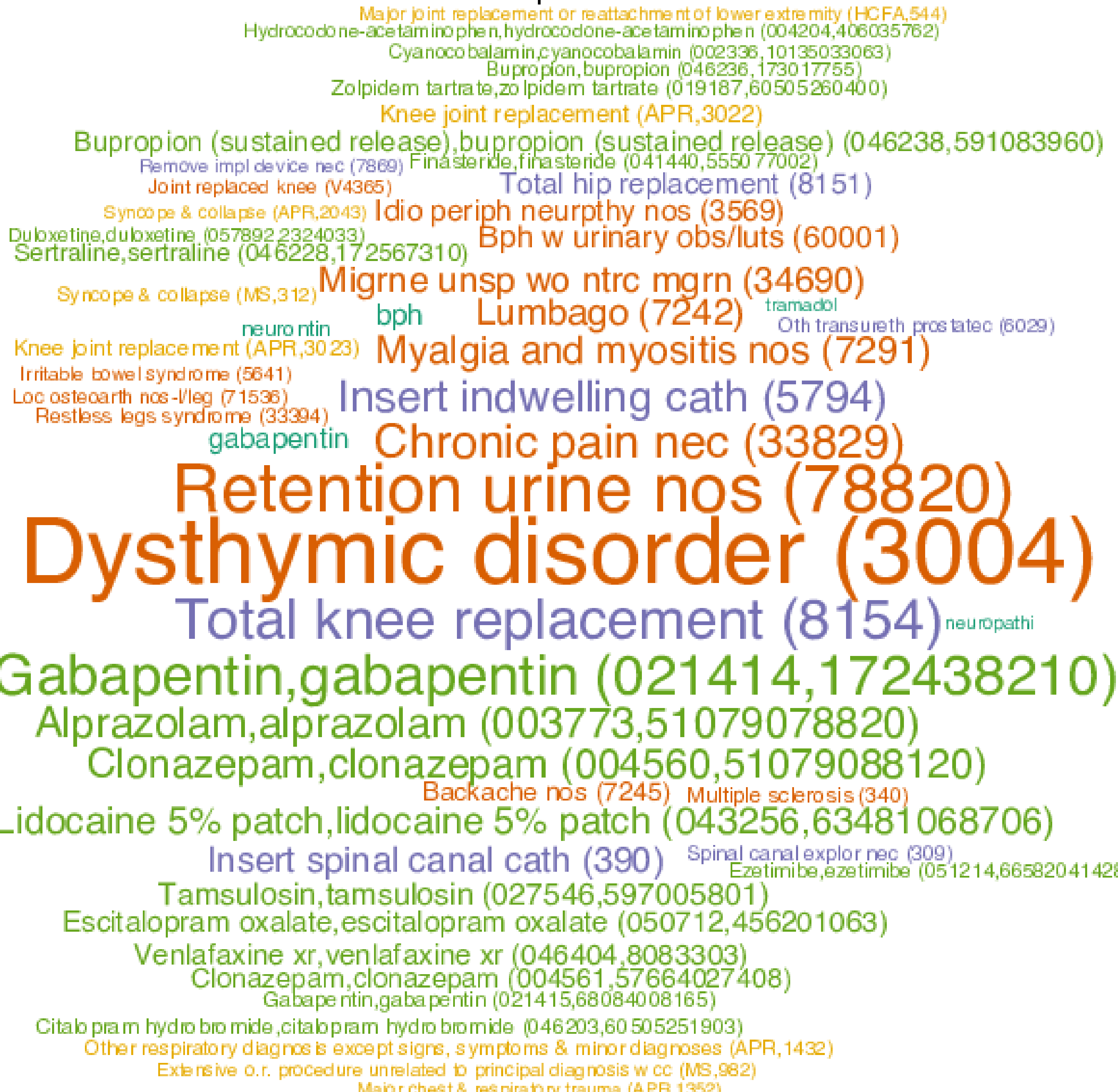
Major joint replacement or reattachment of lower extremity (HCFA,544)
Hydrocodone-acetaminophen,hydrocodone-acetaminophen (004204,406035762)
Cyanocobalamin,cyanocobalamin (002336,10135033063)
Bupropion,bupropion (046236,173017755)
Zolpidem tartrate,zolpidem tartrate (019187,60505260400)
Knee joint replacement (APR,3022)
Bupropion (sustained release),bupropion (sustained release) (046238,591083960)
Remove impl device nec (7869)
Finasteride,finasteride (041440,55077002)
Joint replaced knee (V4365)
Total hip replacement (8151)
Syncope & collapse (APR,2043)
Idio periph neurpthy nos (3569)
Duloxetine,duloxetine (057892,2324033)
Bph w urinary obs/luts (60001)
Sertraline,sertraline (046228,172567310)
Migrne unsp wo ntrc mgrn (34690)
Syncope & collapse (MS,312)
tramadol
bph
Lumbago (7242)
Oth transureth prostatec (6029)
neurontin
Knee joint replacement (APR,3023)
Myalgia and myositis nos (7291)
Irritable bowel syndrome (5641)
Insert indwelling cath (5794)
Loc osteoarth nos-l/leg (71536)
Restless legs syndrome (33394)
gabapentin
Chronic pain nec (33829)
Retention urine nos (78820)
Dysthymic disorder (3004)
Total knee replacement (8154)
neuropathi
Gabapentin,gabapentin (021414,172438210)
Alprazolam,alprazolam (003773,51079078820)
Clonazepam,clonazepam (004560,51079088120)
Backache nos (7245)
Multiple sclerosis (340)
Lidocaine 5% patch,lidocaine 5% patch (043256,63481068706)
Insert spinal canal cath (390)
Spinal canal explor nec (309)
Ezetimibe,ezetimibe (051214,66582041428
Tamsulosin,tamsulosin (027546,597005801)
Escitalopram oxalate,escitalopram oxalate (050712,456201063)
Venlafaxine xr,venlafaxine xr (046404,8083303)
Clonazepam,clonazepam (004561,57664027408)
Gabapentin,gabapentin (021415,68084008165)
Citalopram hydrobromide,citalopram hydrobromide (046203,60505251903)
Other respiratory diagnosis except signs, symptoms & minor diagnoses (APR,1432)
Extensive o.r. procedure unrelated to principal diagnosis w cc (MS,982)
Major chest & respiratory trauma (APR,1352)

Topic 42

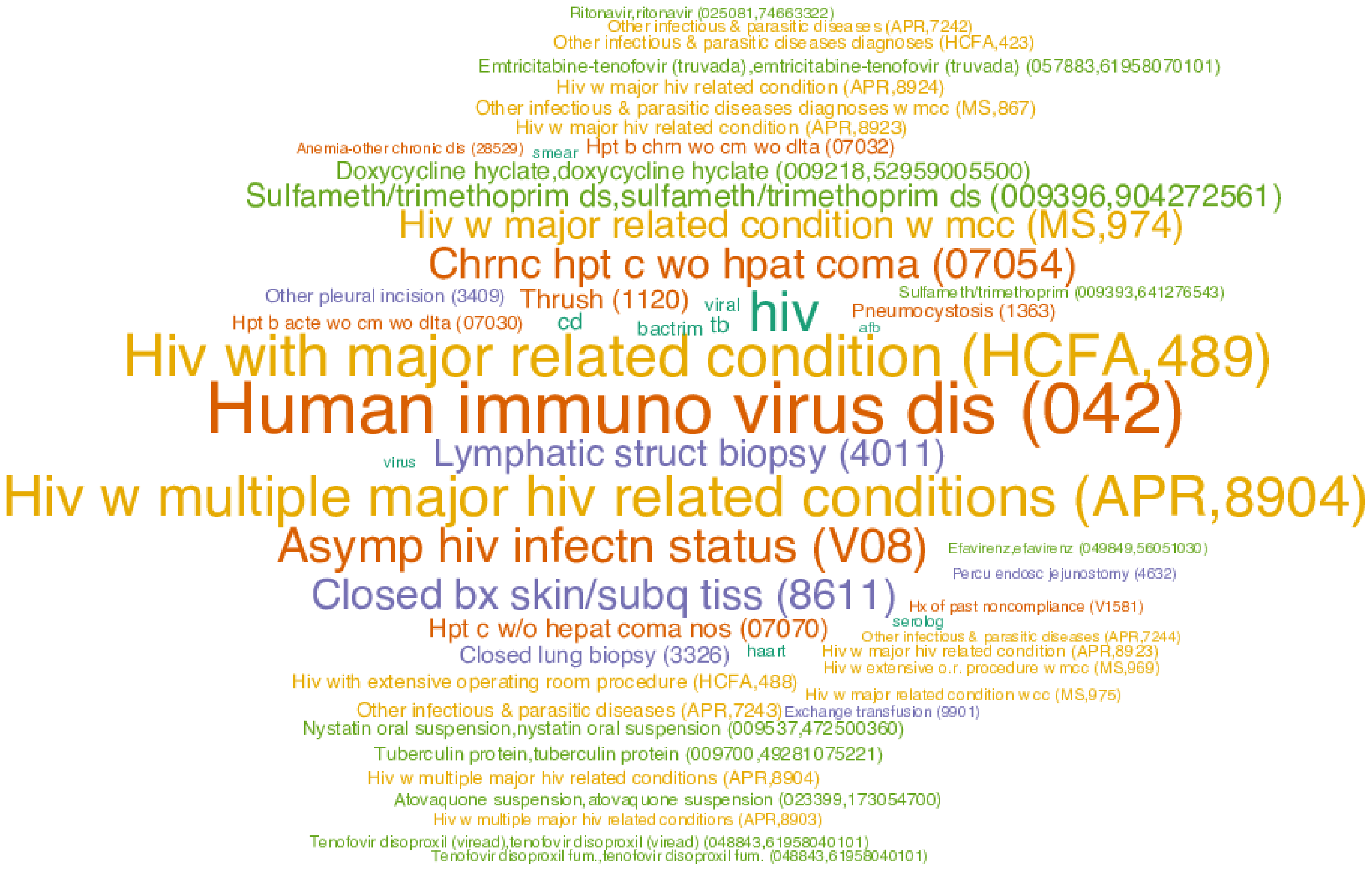
Ritonavir,ritonavir (025081,74663322)
Other infectious & parasitic diseases (APR,7242)
Other infectious & parasitic diseases diagnoses (HCFA,423)
Emtricitabine-tenofovir (truvada),emtricitabine-tenofovir (truvada) (057883,61958070101)
Hiv w major hiv related condition (APR,8924)
Other infectious & parasitic diseases diagnoses w mcc (MS,867)
Hiv w major hiv related condition (APR,8923)
Anemia-other chronic dis (28529)
smear
Hpt b chrn wo cm wo dlta (07032)
Doxycycline hyclate,doxycycline hyclate (009218,52959005500)
Sulfameth/trimethoprim ds,sulfameth/trimethoprim ds (009396,904272561)
Hiv w major related condition w mcc (MS,974)
Chrnc hpt c wo hpat coma (07054)
Other pleural incision (3409)
Thrush (1120)
viral
hiv
Sulfameth/trimethoprim (009393,641276543)
Pneumocystosis (1363)
Hpt b acte wo cm wo dlta (07030)
cd
bactrim
tb
afb
Hiv with major related condition (HCFA,489)
Human immuno virus dis (042)
virus
Lymphatic struct biopsy (4011)
Hiv w multiple major hiv related conditions (APR,8904)
Asymp hiv infectn status (V08)
Efavirenz,efavirenz (049849,56051030)
Percu endosc jejunostomy (4632)
Closed bx skin/subq tiss (8611)
Hx of past noncompliance (V1581)
serolog
Hpt c w/o hepat coma nos (07070)
Other infectious & parasitic diseases (APR,7244)
Closed lung biopsy (3326)
haart
Hiv w major hiv related condition (APR,8923)
Hiv w extensive o.r. procedure w mcc (MS,969)
Hiv with extensive operating room procedure (HCFA,488)
Hiv w major related condition w cc (MS,975)
Other infectious & parasitic diseases (APR,7243)
Exchange transfusion (9901)
Nystatin oral suspension,nystatin oral suspension (009537,472500360)
Tuberculin protein,tuberculin protein (009700,49281075221)
Hiv w multiple major hiv related conditions (APR,8904)
Atovaquone suspension,atovaquone suspension (023399,173054700)
Hiv w multiple major hiv related conditions (APR,8903)
Tenofovir disoproxil (viread),tenofovir disoproxil (viread) (048843,61958040101)
Tenofovir disoproxil fum.,tenofovir disoproxil fum. (048843,61958040101)

Topic 43

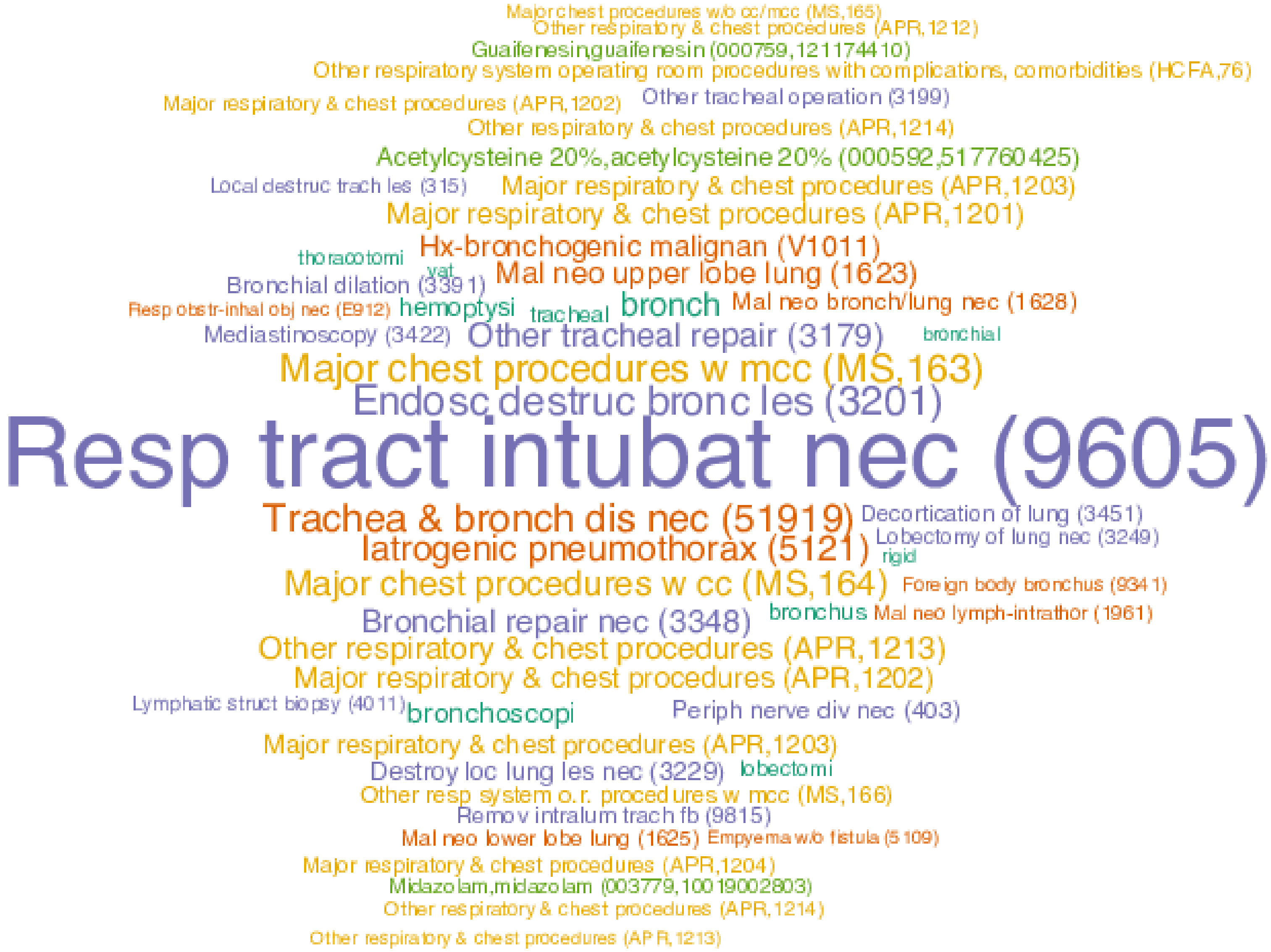
Major chest procedures w/o cc/mcc (MS,165)
Other respiratory & chest procedures (APR,1212)
Guaifenesin,guaifenesin (000759,121174410)
Other respiratory system operating room procedures with complications, comorbidities (HCFA,76)
Major respiratory & chest procedures (APR,1202)
Other tracheal operation (3199)
Other respiratory & chest procedures (APR,1214)
Acetylcysteine 20%,acetylcysteine 20% (000592,517760425)
Local destruc trach les (315)
Major respiratory & chest procedures (APR,1203)
Major respiratory & chest procedures (APR,1201)
thoracotomi
Hx-bronchogenic malignan (V1011)
vat
Mal neo upper lobe lung (1623)
Bronchial dilation (3391)
Resp obstr-inhal obj nec (E912)
hemoptysi
tracheal
bronch
Mal neo bronch/lung nec (1628)
Mediastinoscopy (3422)
Other tracheal repair (3179)
bronchial
Major chest procedures w mcc (MS,163)
Endosc destruc bronc les (3201)
Resp tract intubat nec (9605)
Trachea & bronch dis nec (51919)
Decortication of lung (3451)
Lobectomy of lung nec (3249)
Iatrogenic pneumothorax (5121)
rigid
Major chest procedures w cc (MS,164)
Foreign body bronchus (9341)
Bronchial repair nec (3348)
bronchus
Mal neo lymph-intrathor (1961)
Other respiratory & chest procedures (APR,1213)
Major respiratory & chest procedures (APR,1202)
Lymphatic struct biopsy (4011)
bronchoscopi
Periph nerve div nec (403)
Major respiratory & chest procedures (APR,1203)
Destroy loc lung les nec (3229)
lobectomi
Other resp system o.r. procedures w mcc (MS,166)
Remov intralum trach fb (9815)
Mal neo lower lobe lung (1625)
Empyema w/o fistula (5109)
Major respiratory & chest procedures (APR,1204)
Midazolam,midazolam (003779,10019002803)
Other respiratory & chest procedures (APR,1214)
Other respiratory & chest procedures (APR,1213)

Topic 44

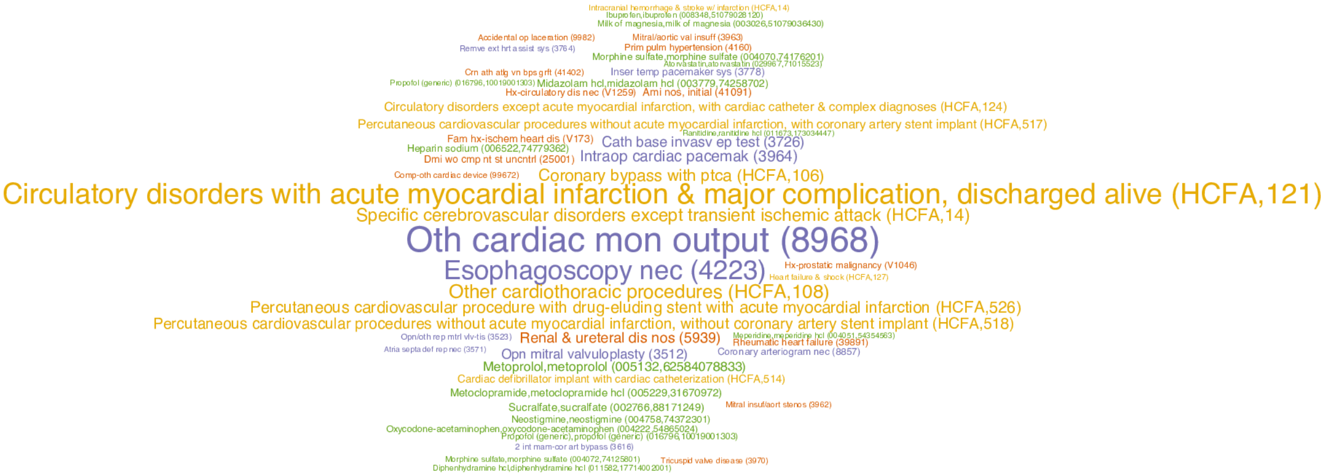
Intracranial hemorrhage & stroke w/ infarction (HCFA,14)
Ibuprofen,ibuprofen (008348,51079028120)
Milk of magnesia,milk of magnesia (003026,51079036430)
Accidental op laceration (9982)
Mitral/aortic val insuff (3963)
Remve ext hrt assist sys (3764)
Prim pulm hypertension (4160)
Morphine sulfate,morphine sulfate (004070,74176201)
Atorvastatin,atorvastatin (029967,71015523)
Crn ath atlg vn bps grft (41402)
Inser temp pacemaker sys (3778)
Propofol (generic) (016796,10019001303)
Midazolam hcl,midazolam hcl (003779,74258702)
Hx-circulatory dis nec (V1259)
Ami nos, initial (41091)
Circulatory disorders except acute myocardial infarction, with cardiac catheter & complex diagnoses (HCFA,124)
Percutaneous cardiovascular procedures without acute myocardial infarction, with coronary artery stent implant (HCFA,517)
Ranitidine,ranitidine hcl (011673,173034447)
Fam hx-ischem heart dis (V173)
Cath base invasv ep test (3726)
Heparin sodium (006522,74779362)
Intraop cardiac pacemak (3964)
Dmi wo cmp nt st uncntrl (25001)
Comp-oth cardiac device (99672)
Coronary bypass with ptca (HCFA,106)
Circulatory disorders with acute myocardial infarction & major complication, discharged alive (HCFA,121)
Specific cerebrovascular disorders except transient ischemic attack (HCFA,14)
Oth cardiac mon output (8968)
Esophagoscopy nec (4223)
Hx-prostatic malignancy (V1046)
Heart failure & shock (HCFA,127)
Other cardiothoracic procedures (HCFA,108)
Percutaneous cardiovascular procedure with drug-eluding stent with acute myocardial infarction (HCFA,526)
Percutaneous cardiovascular procedures without acute myocardial infarction, without coronary artery stent implant (HCFA,518)
Opn/oth rep mtrl vlv-tis (3523)
Renal & ureteral dis nos (5939)
Meperidine,meperidine hcl (004051,54354563)
Rheumatic heart failure (39891)
Atria septa def rep nec (3571)
Opn mitral valvuloplasty (3512)
Coronary arteriogram nec (8857)
Metoprolol,metoprolol (005132,62584078833)
Cardiac defibrillator implant with cardiac catheterization (HCFA,514)
Metoclopramide,metoclopramide hcl (005229,31670972)
Sucralfate,sucralfate (002766,88171249)
Mitral insuf/aort stenos (3962)
Neostigmine,neostigmine (004758,74372301)
Oxycodone-acetaminophen,oxycodone-acetaminophen (004222,54865024)
Propofol (generic),propofol (generic) (016796,10019001303)
2 int mam-cor art bypass (3616)
Morphine sulfate,morphine sulfate (004072,74125801)
Tricuspid valve disease (3970)
Diphenhydramine hcl,diphenhydramine hcl (011582,17714002001)

Topic 45

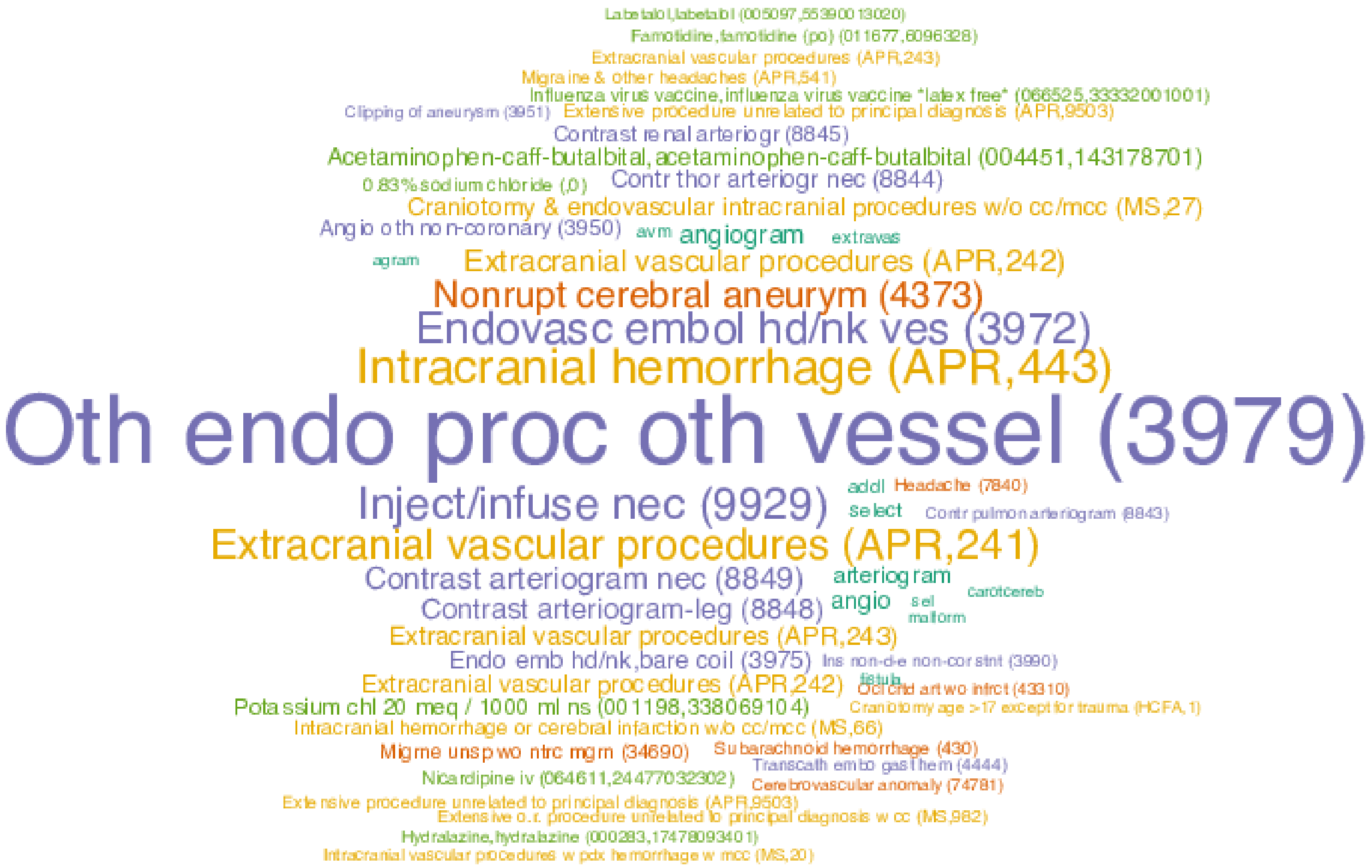
Labetalol,labetalol (005097,55390013020)
Famotidine,famotidine (po) (011677,6096328)
Extracranial vascular procedures (APR,243)
Migraine & other headaches (APR,541)
Influenza virus vaccine,influenza virus vaccine *latex free* (066525,33332001001)
Clipping of aneurysm (3951)
Extensive procedure unrelated to principal diagnosis (APR,9503)
Contrast renal arteriogr (8845)
Acetaminophen-caff-butalbital,acetaminophen-caff-butalbital (004451,143178701)
0.83% sodium chloride (,0)
Contr thor arteriogr nec (8844)
Craniotomy & endovascular intracranial procedures w/o cc/mcc (MS,27)
Angio oth non-coronary (3950)
avm
angiogram
extravas
agram
Extracranial vascular procedures (APR,242)
Nonrupt cerebral aneurym (4373)
Endovasc embol hd/nk ves (3972)
Intracranial hemorrhage (APR,443)
Oth endo proc oth vessel (3979)
addl
Headache (7840)
Inject/infuse nec (9929)
select
Contr pulmon arteriogram (8843)
Extracranial vascular procedures (APR,241)
Contrast arteriogram nec (8849)
arteriogram
carotcereb
Contrast arteriogram-leg (8848)
angio
sel
malform
Extracranial vascular procedures (APR,243)
Endo emb hd/nk,bare coil (3975)
Ins non-d-e non-cor stnt (3990)
Extracranial vascular procedures (APR,242)
fistula
Ocl crtd art wo infrct (43310)
Potassium chl 20 meq / 1000 ml ns (001198,338069104)
Craniotomy age >17 except for trauma (HCFA,1)
Intracranial hemorrhage or cerebral infarction w/o cc/mcc (MS,66)
Migrne unsp wo ntrc mgrn (34690)
Subarachnoid hemorrhage (430)
Transcath embo gast hem (4444)
Nicardipine iv (064611,24477032302)
Cerebrovascular anomaly (74781)
Extensive procedure unrelated to principal diagnosis (APR,9503)
Extensive o.r. procedure unrelated to principal diagnosis w cc (MS,982)
Hydralazine,hydralazine (000283,17478093401)
Intracranial vascular procedures w pdx hemorrhage w mcc (MS,20)

Topic 46

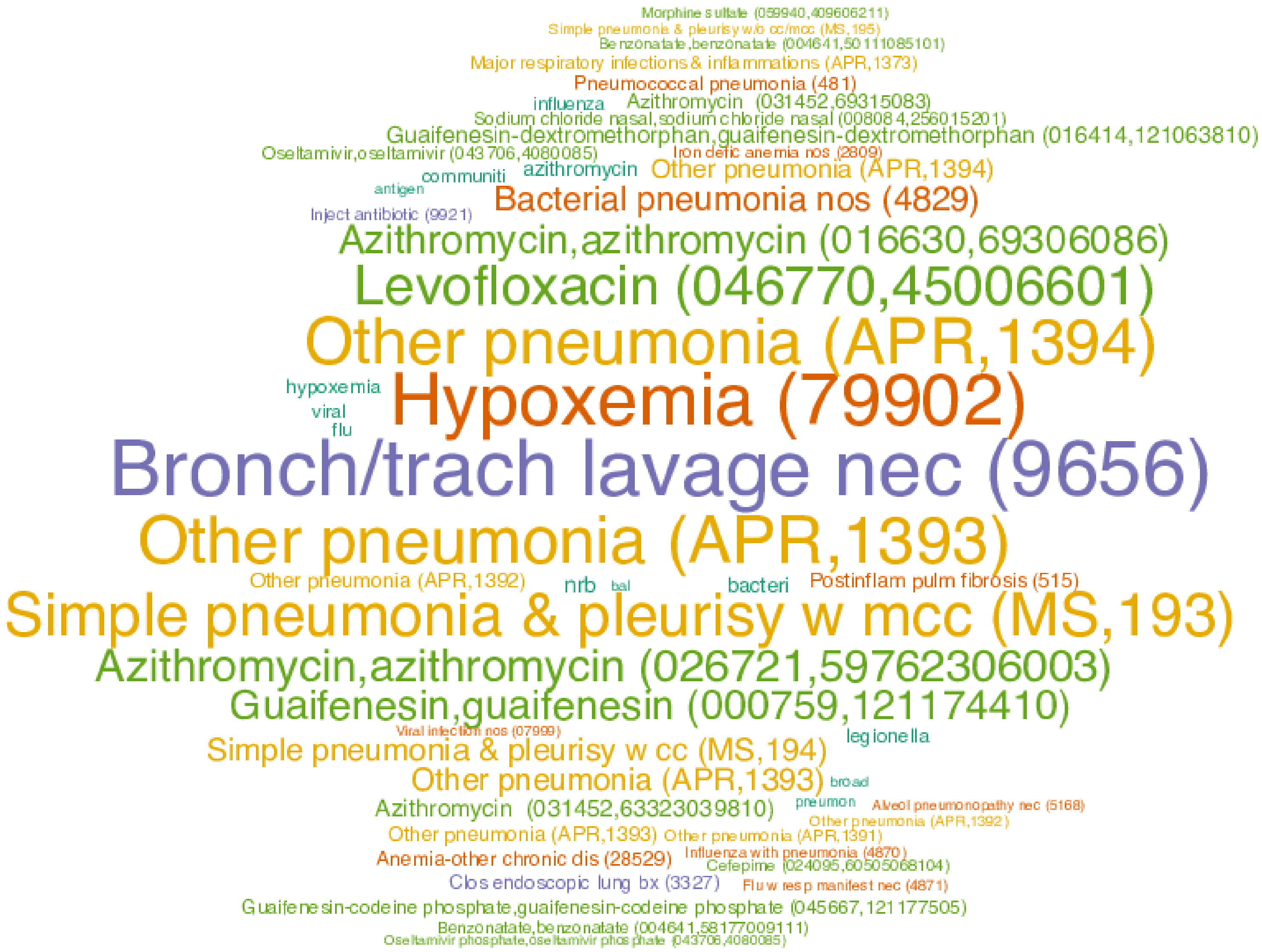
Morphine sulfate (059940,409606211)
Simple pneumonia & pleurisy w/o cc/mcc (MS,195)
Benzonatate,benzonatate (004641,50111085101)
Major respiratory infections & inflammations (APR,1373)
Pneumococcal pneumonia (481)
influenza
Azithromycin (031452,69315083)
Sodium chloride nasal,sodium chloride nasal (008084,256015201)
Guaifenesin-dextromethorphan,guaifenesin-dextromethorphan (016414,121063810)
Oseltamivir,oseltamivir (043706,4080085)
Iron defic anemia nos (2809)
communiti
azithromycin
Other pneumonia (APR,1394)
antigen
Bacterial pneumonia nos (4829)
Inject antibiotic (9921)
Azithromycin,azithromycin (016630,69306086)
Levofloxacin (046770,45006601)
Other pneumonia (APR,1394)
hypoxemia
Hypoxemia (79902)
viral
flu
Bronch/trach lavage nec (9656)
Other pneumonia (APR,1393)
Other pneumonia (APR,1392)
nrb
bal
bacteri
Postinflam pulm fibrosis (515)
Simple pneumonia & pleurisy w mcc (MS,193)
Azithromycin,azithromycin (026721,59762306003)
Guaifenesin,guaifenesin (000759,121174410)
Viral infection nos (07999)
legionella
Simple pneumonia & pleurisy w cc (MS,194)
Other pneumonia (APR,1393)
broad
Azithromycin (031452,63323039810)
pneumon
Alveol pneumonopathy nec (5168)
Other pneumonia (APR,1392)
Other pneumonia (APR,1393)
Other pneumonia (APR,1391)
Anemia-other chronic dis (28529)
Influenza with pneumonia (4870)
Cefepime (024095,60505068104)
Clos endoscopic lung bx (3327)
Flu w resp manifest nec (4871)
Guaifenesin-codeine phosphate,guaifenesin-codeine phosphate (045667,121177505)
Benzonatate,benzonatate (004641,58177009111)
Oseltamivir phosphate,oseltamivir phosphate (043706,4080085)

Topic 47

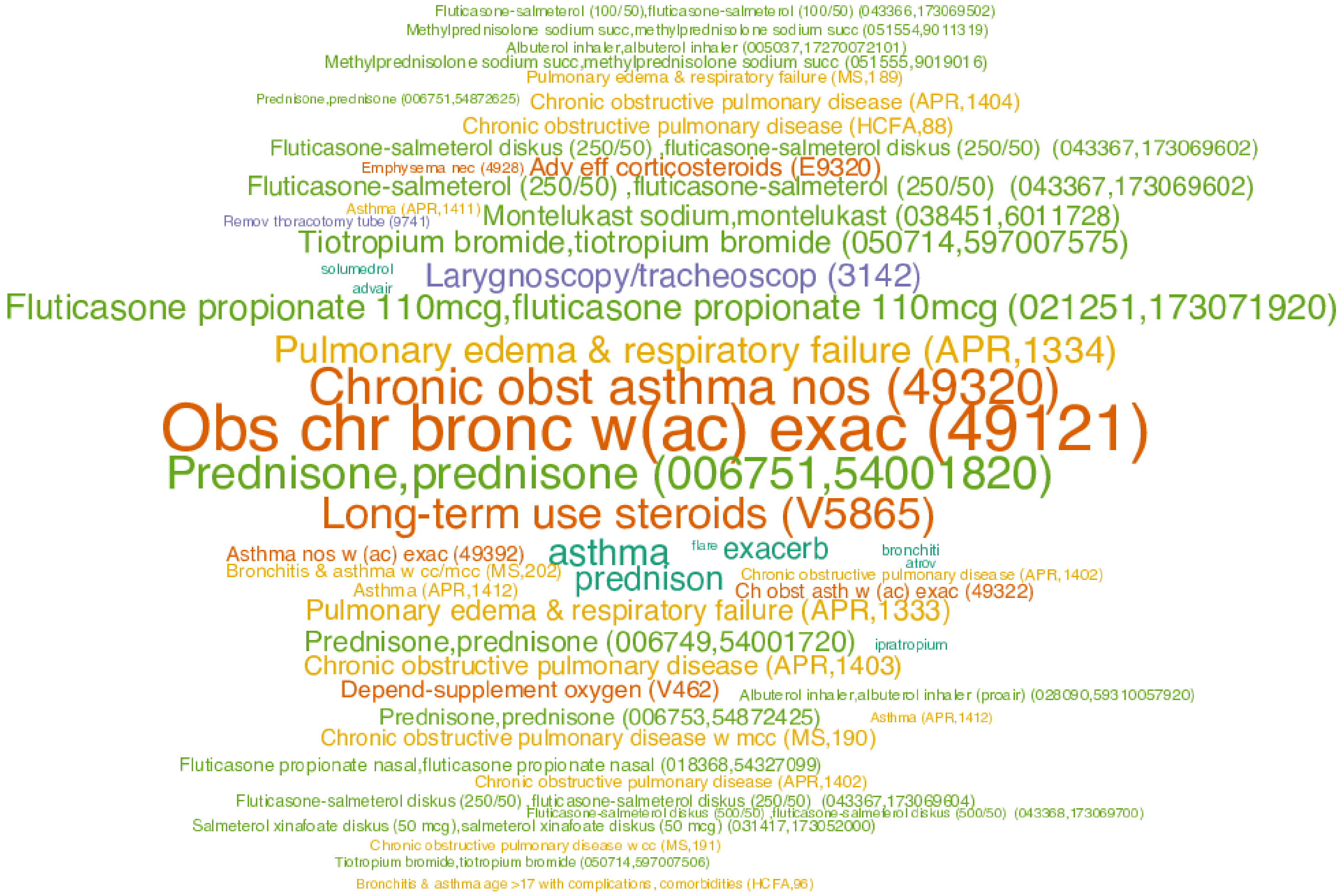

Fluticasone-salmeterol (100/50),fluticasone-salmeterol (100/50) (043366,173069502)
Methylprednisolone sodium succ,methylprednisolone sodium succ (051554,9011319)
Albuterol inhaler,albuterol inhaler (005037,17270072101)
Methylprednisolone sodium succ,methylprednisolone sodium succ (051555,9019016)
Pulmonary edema & respiratory failure (MS,189)
Prednisone,prednisone (006751,54872625)
Chronic obstructive pulmonary disease (APR,1404)
Chronic obstructive pulmonary disease (HCFA,88)
Fluticasone-salmeterol diskus (250/50) ,fluticasone-salmeterol diskus (250/50) (043367,173069602)
Emphysema nec (4928)
Adv eff corticosteroids (E9320)
Fluticasone-salmeterol (250/50) ,fluticasone-salmeterol (250/50) (043367,173069602)
Asthma (APR,1411)
Remov thoracotomy tube (9741)
Montelukast sodium,montelukast (038451,6011728)
Tiotropium bromide,tiotropium bromide (050714,597007575)
solumedrol
advair
Larygnoscopy/tracheoscop (3142)
Fluticasone propionate 110mcg,fluticasone propionate 110mcg (021251,173071920)
Pulmonary edema & respiratory failure (APR,1334)
Chronic obst asthma nos (49320)
Obs chr bronc w(ac) exac (49121)
Prednisone,prednisone (006751,54001820)
Long-term use steroids (V5865)
Asthma nos w (ac) exac (49392)
asthma
flare
exacerb
bronchiti
atrov
Bronchitis & asthma w cc/mcc (MS,202)
prednison
Chronic obstructive pulmonary disease (APR,1402)
Asthma (APR,1412)
Ch obst asth w (ac) exac (49322)
Pulmonary edema & respiratory failure (APR,1333)
Prednisone,prednisone (006749,54001720)
ipratropium
Chronic obstructive pulmonary disease (APR,1403)
Depend-supplement oxygen (V462)
Albuterol inhaler,albuterol inhaler (proair) (028090,59310057920)
Prednisone,prednisone (006753,54872425)
Asthma (APR,1412)
Chronic obstructive pulmonary disease w mcc (MS,190)
Fluticasone propionate nasal,fluticasone propionate nasal (018368,54327099)
Chronic obstructive pulmonary disease (APR,1402)
Fluticasone-salmeterol diskus (250/50) ,fluticasone-salmeterol diskus (250/50) (043367,173069604)
Fluticasone-salmeterol diskus (500/50) ,fluticasone-salmeterol diskus (500/50) (043368,173069700)
Salmeterol xinafoate diskus (50 mcg),salmeterol xinafoate diskus (50 mcg) (031417,173052000)
Chronic obstructive pulmonary disease w cc (MS,191)
Tiotropium bromide,tiotropium bromide (050714,597007506)
Bronchitis & asthma age >17 with complications, comorbidities (HCFA,96)

Topic 48

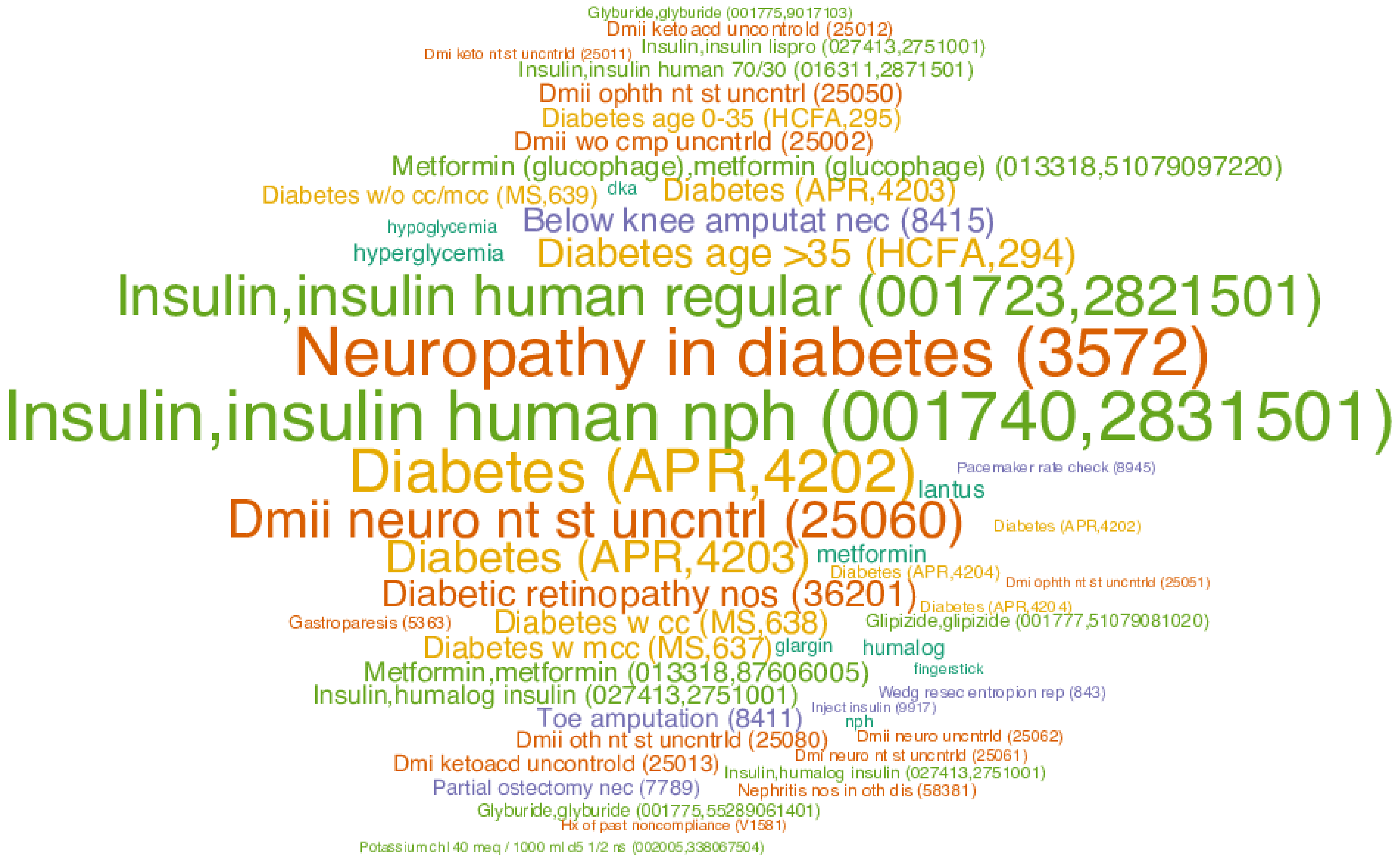
Glyburide,glyburide (001775,9017103)
Dmii ketoacd uncontrold (25012)
Dmi keto nt st uncntrld (25011)
Insulin,insulin lispro (027413,2751001)
Insulin,insulin human 70/30 (016311,2871501)
Dmii ophth nt st uncntrl (25050)
Diabetes age 0-35 (HCFA,295)
Dmii wo cmp uncntrld (25002)
Metformin (glucophage),metformin (glucophage) (013318,51079097220)
Diabetes w/o cc/mcc (MS,639)
dka
Diabetes (APR,4203)
hypoglycemia
Below knee amputat nec (8415)
hyperglycemia
Diabetes age >35 (HCFA,294)
Insulin,insulin human regular (001723,2821501)
Neuropathy in diabetes (3572)
Insulin,insulin human nph (001740,2831501)
Diabetes (APR,4202)
Pacemaker rate check (8945)
lantus
Dmii neuro nt st uncntrl (25060)
Diabetes (APR,4202)
Diabetes (APR,4203)
metformin
Diabetes (APR,4204)
Dmi ophth nt st uncntrld (25051)
Diabetic retinopathy nos (36201)
Diabetes (APR,4204)
Gastroparesis (5363)
Diabetes w cc (MS,638)
Glipizide,glipizide (001777,51079081020)
Diabetes w mcc (MS,637)
glargin
humalog
Metformin,metformin (013318,87606005)
fingerstick
Insulin,humalog insulin (027413,2751001)
Wedg resec entropion rep (843)
Inject insulin (9917)
Toe amputation (8411)
nph
Dmii oth nt st uncntrld (25080)
Dmii neuro uncntrld (25062)
Dmi neuro nt st uncntrld (25061)
Dmi ketoacd uncontrold (25013)
Insulin,humalog insulin (027413,2751001)
Partial ostectomy nec (7789)
Nephritis nos in oth dis (58381)
Glyburide,glyburide (001775,55289061401)
Hx of past noncompliance (V1581)
Potassium chl 40 meq / 1000 ml d5 1/2 ns (002005,338067504)

Topic 49

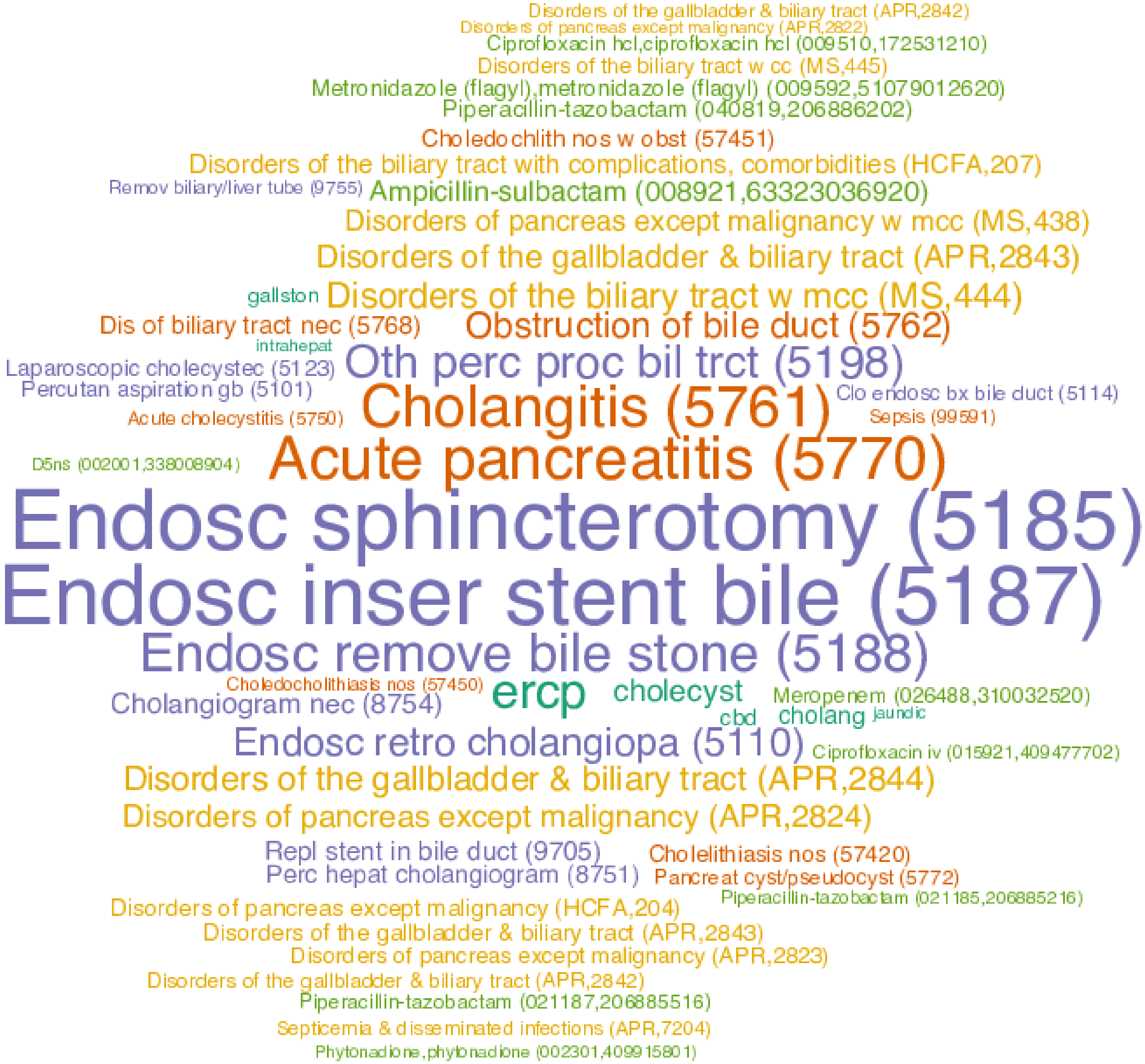

Disorders of the gallbladder & biliary tract (APR,2842)
Disorders of pancreas except malignancy (APR,2822)
Ciprofloxacin hcl,ciprofloxacin hcl (009510,172531210)
Disorders of the biliary tract w cc (MS,445)
Metronidazole (flagyl),metronidazole (flagyl) (009592,51079012620)
Piperacillin-tazobactam (040819,206886202)
Choledochlith nos w obst (57451)
Disorders of the biliary tract with complications, comorbidities (HCFA,207)
Remov biliary/liver tube (9755)
Ampicillin-sulbactam (008921,63323036920)
Disorders of pancreas except malignancy w mcc (MS,438)
Disorders of the gallbladder & biliary tract (APR,2843)
gallston
Disorders of the biliary tract w mcc (MS,444)
Dis of biliary tract nec (5768)
Obstruction of bile duct (5762)
intrahepat
Laparoscopic cholecystec (5123)
Oth perc proc bil trct (5198)
Percutan aspiration gb (5101)
Clo endosc bx bile duct (5114)
Acute cholecystitis (5750)
Cholangitis (5761)
Sepsis (99591)
D5ns (002001,338008904)
Acute pancreatitis (5770)
Endosc sphincterotomy (5185)
Endosc inser stent bile (5187)
Endosc remove bile stone (5188)
Choledocholithiasis nos (57450)
Cholangiogram nec (8754)
ercp
cholecyst
Meropenem (026488,310032520)
cbd
cholang
jaundic
Endosc retro cholangiopa (5110)
Ciprofloxacin iv (015921,409477702)
Disorders of the gallbladder & biliary tract (APR,2844)
Disorders of pancreas except malignancy (APR,2824)
Repl stent in bile duct (9705)
Cholelithiasis nos (57420)
Perc hepat cholangiogram (8751)
Pancreat cyst/pseudocyst (5772)
Piperacillin-tazobactam (021185,206885216)
Disorders of pancreas except malignancy (HCFA,204)
Disorders of the gallbladder & biliary tract (APR,2843)
Disorders of pancreas except malignancy (APR,2823)
Disorders of the gallbladder & biliary tract (APR,2842)
Piperacillin-tazobactam (021187,206885516)
Septicemia & disseminated infections (APR,7204)
Phytonadione,phytonadione (002301,409915801)

Topic 50

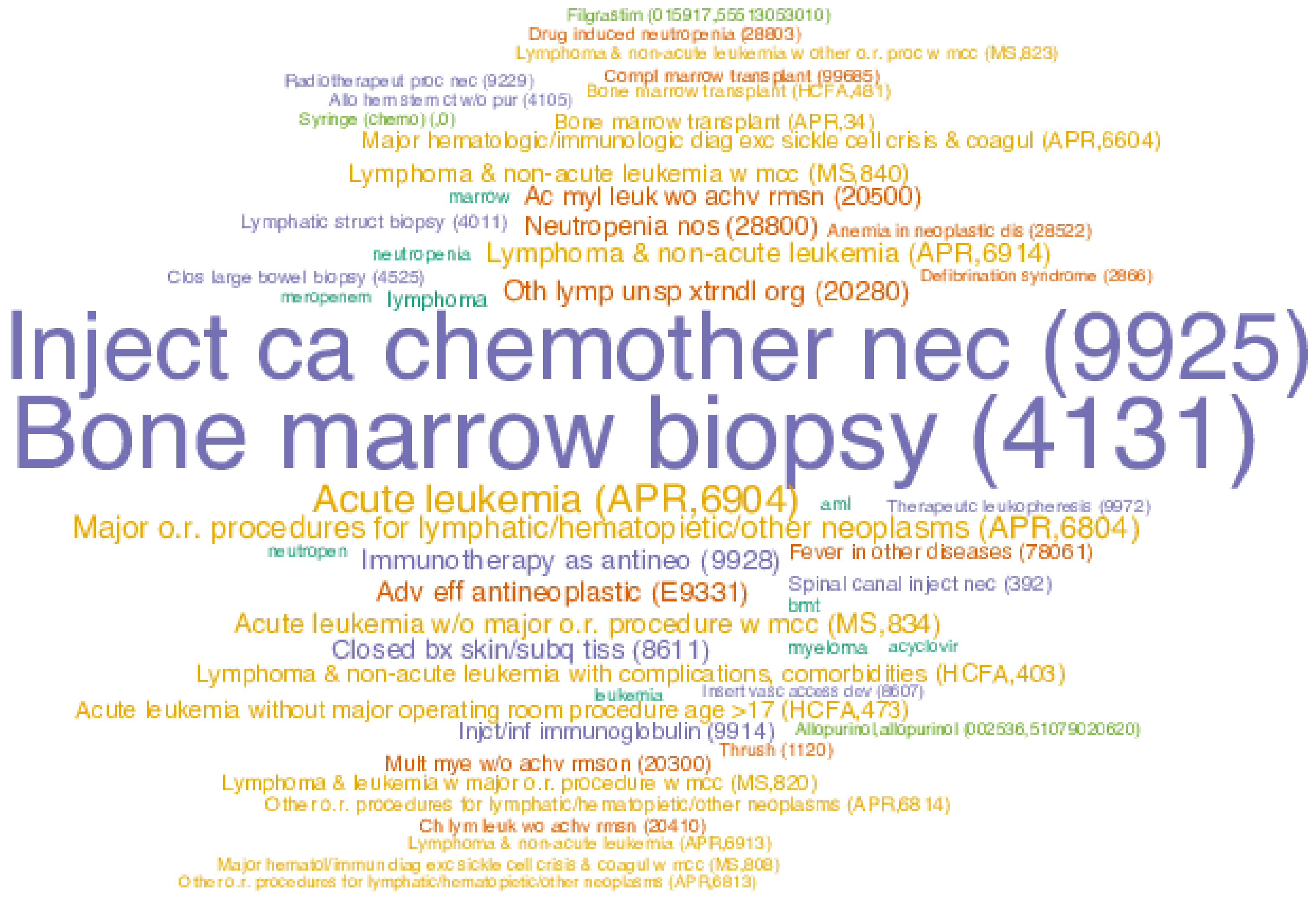
Filgrastim (015917,55513053010)
Drug induced neutropenia (28803)
Lymphoma & non-acute leukemia w other o.r. proc w mcc (MS,823)
Radiotherapeut proc nec (9229)
Compl marrow transplant (99685)
Bone marrow transplant (HCFA,481)
Allo hem stem ct w/o pur (4105)
Syringe (chemo) (,0)
Bone marrow transplant (APR,34)
Major hematologic/immunologic diag exc sickle cell crisis & coagul (APR,6604)
Lymphoma & non-acute leukemia w mcc (MS,840)
marrow
Ac myl leuk wo achv rmsn (20500)
Lymphatic struct biopsy (4011)
Neutropenia nos (28800)
Anemia in neoplastic dis (28522)
neutropenia
Lymphoma & non-acute leukemia (APR,6914)
Clos large bowel biopsy (4525)
Defibrination syndrome (2866)
meropenem
lymphoma
Oth lymp unsp xtrndl org (20280)
Inject ca chemother nec (9925)
Bone marrow biopsy (4131)
Acute leukemia (APR,6904)
aml
Therapeutc leukopheresis (9972)
Major o.r. procedures for lymphatic/hematopietic/other neoplasms (APR,6804)
neutropen
Immunotherapy as antineo (9928)
Fever in other diseases (78061)
Adv eff antineoplastic (E9331)
Spinal canal inject nec (392)
bmt
Acute leukemia w/o major o.r. procedure w mcc (MS,834)
Closed bx skin/subq tiss (8611)
myeloma
acyclovir
Lymphoma & non-acute leukemia with complications, comorbidities (HCFA,403)
leukemia
Insert vasc access dev (8607)
Acute leukemia without major operating room procedure age >17 (HCFA,473)
Injct/inf immunoglobulin (9914)
Allopurinol,allopurinol (002536,51079020620)
Thrush (1120)
Mult mye w/o achv rmson (20300)
Lymphoma & leukemia w major o.r. procedure w mcc (MS,820)
Other o.r. procedures for lymphatic/hematopietic/other neoplasms (APR,6814)
Ch lym leuk wo achv rmsn (20410)
Lymphoma & non-acute leukemia (APR,6913)
Major hematol/immun diag exc sickle cell crisis & coagul w mcc (MS,808)
Other o.r. procedures for lymphatic/hematopietic/other neoplasms (APR,6813)

Topic 51

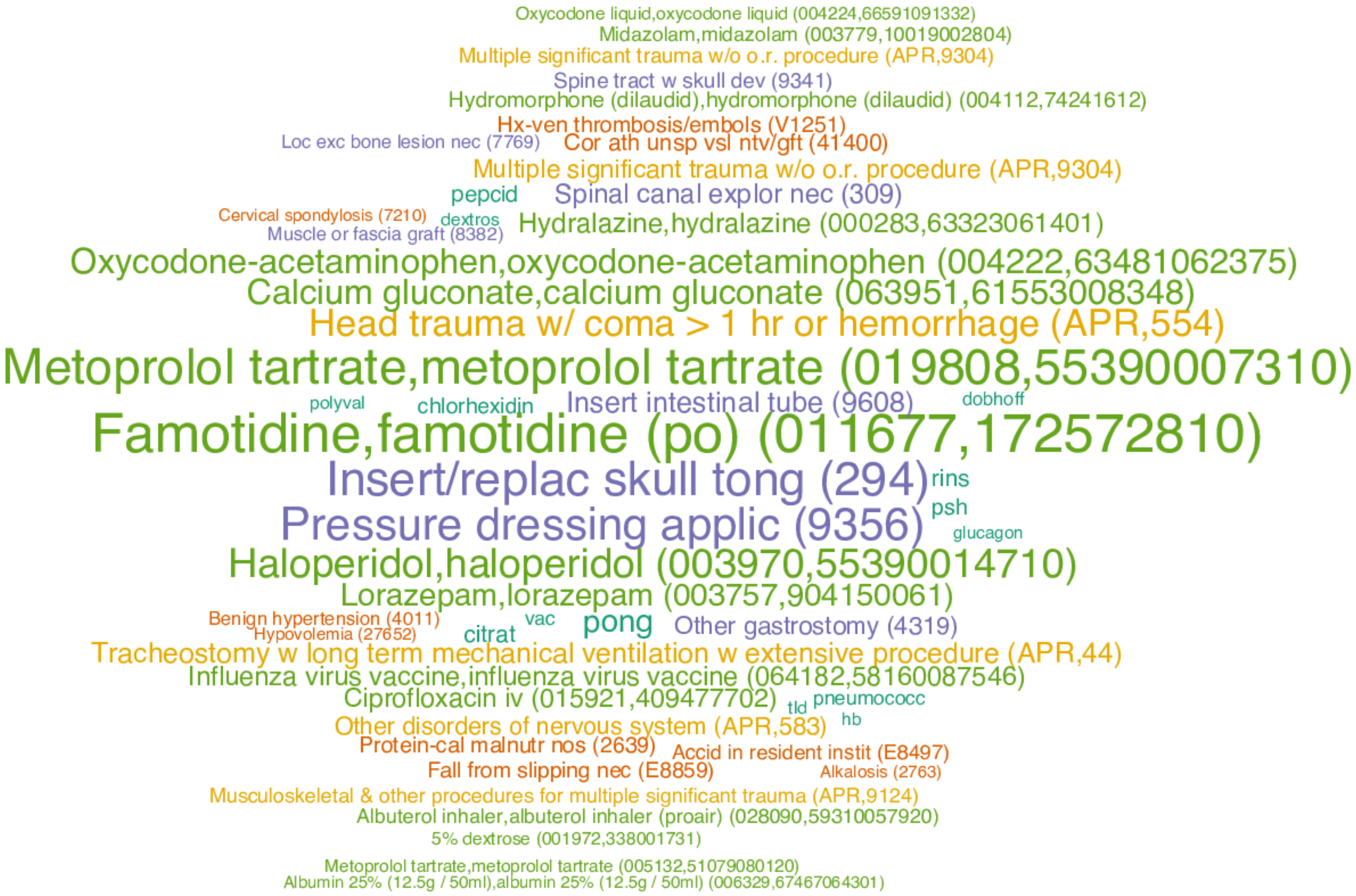
Oxycodone liquid,oxycodone liquid (004224,66591091332)
Midazolam,midazolam (003779,10019002804)
Multiple significant trauma w/o o.r. procedure (APR,9304)
Spine tract w skull dev (9341)
Hydromorphone (dilaudid),hydromorphone (dilaudid) (004112,74241612)
Hx-ven thrombosis/embols (V1251)
Loc exc bone lesion nec (7769)
Cor ath unsp vsl ntv/gft (41400)
Multiple significant trauma w/o o.r. procedure (APR,9304)
pepcid
Spinal canal explor nec (309)
Cervical spondylosis (7210)
dextros
Muscle or fascia graft (8382)
Hydralazine,hydralazine (000283,63323061401)
Oxycodone-acetaminophen,oxycodone-acetaminophen (004222,63481062375)
Calcium gluconate,calcium gluconate (063951,61553008348)
Head trauma w/ coma > 1 hr or hemorrhage (APR,554)
Metoprolol tartrate,metoprolol tartrate (019808,55390007310)
polyval
chlorhexidin
Insert intestinal tube (9608)
dobhoff
Famotidine,famotidine (po) (011677,172572810)
Insert/replac skull tong (294)
rins
Pressure dressing applic (9356)
psh
glucagon
Haloperidol,haloperidol (003970,55390014710)
Lorazepam,lorazepam (003757,904150061)
Benign hypertension (4011)
Hypovolemia (27652)
citrat
vac
pong
Other gastrostomy (4319)
Tracheostomy w long term mechanical ventilation w extensive procedure (APR,44)
Influenza virus vaccine,influenza virus vaccine (064182,58160087546)
Ciprofloxacin iv (015921,409477702)
tld
pneumococc
hb
Other disorders of nervous system (APR,583)
Protein-cal malnutr nos (2639)
Accid in resident instit (E8497)
Fall from slipping nec (E8859)
Alkalosis (2763)
Musculoskeletal & other procedures for multiple significant trauma (APR,9124)
Albuterol inhaler,albuterol inhaler (proair) (028090,59310057920)
5% dextrose (001972,338001731)
Metoprolol tartrate,metoprolol tartrate (005132,51079080120)
Albumin 25% (12.5g / 50ml),albumin 25% (12.5g / 50ml) (006329,67467064301)

Topic 52

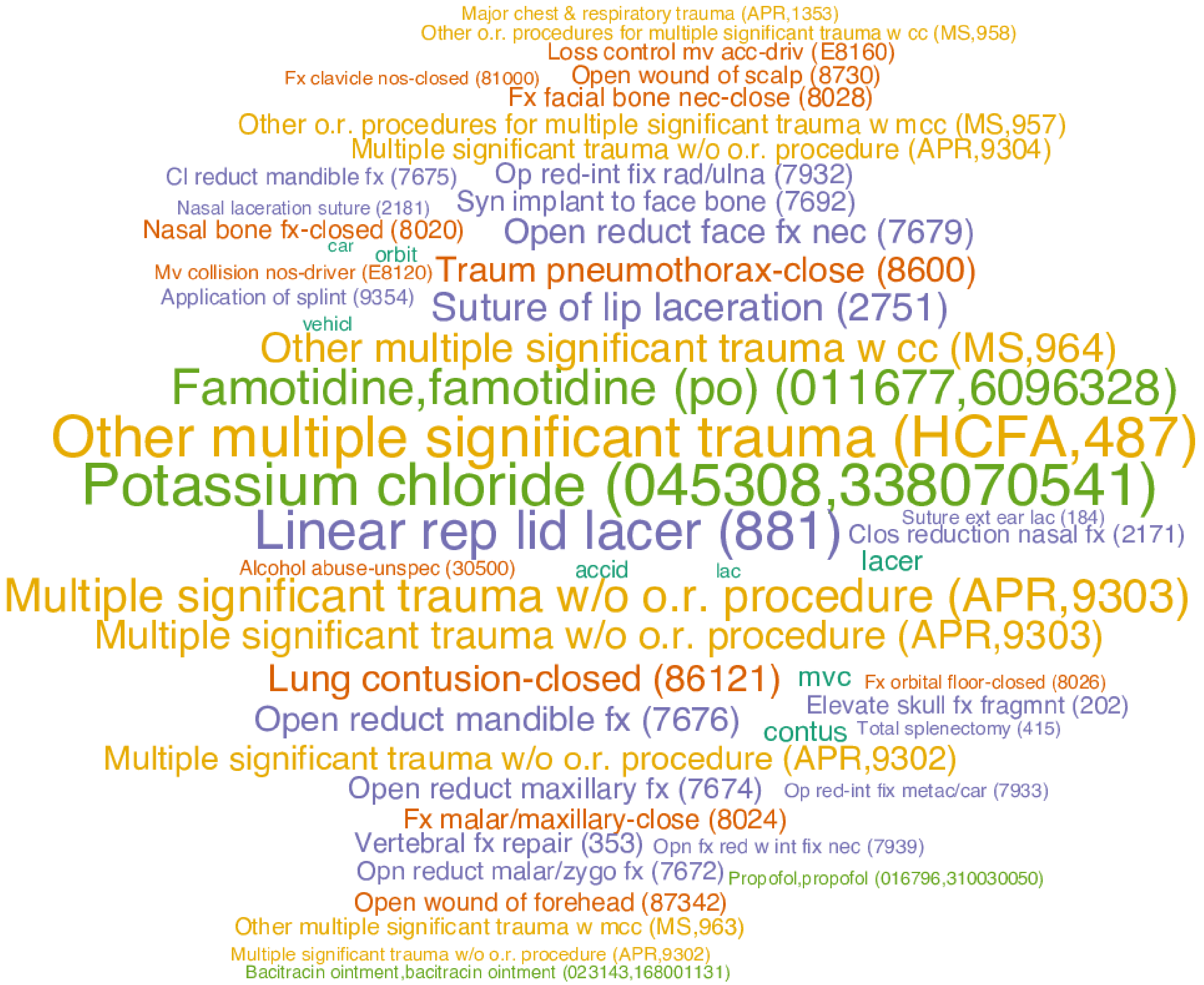

Major chest & respiratory trauma (APR,1353)
Other o.r. procedures for multiple significant trauma w cc (MS,958)
Loss control mv acc-driv (E8160)
Fx clavicle nos-closed (81000)
Open wound of scalp (8730)
Fx facial bone nec-close (8028)
Other o.r. procedures for multiple significant trauma w mcc (MS,957)
Multiple significant trauma w/o o.r. procedure (APR,9304)
Cl reduct mandible fx (7675)
Op red-int fix rad/ulna (7932)
Nasal laceration suture (2181)
Syn implant to face bone (7692)
Nasal bone fx-closed (8020)
Open reduct face fx nec (7679)
car
orbit
Mv collision nos-driver (E8120)
Traum pneumothorax-close (8600)
Application of splint (9354)
Suture of lip laceration (2751)
vehicl
Other multiple significant trauma w cc (MS,964)
Famotidine,famotidine (po) (011677,6096328)
Other multiple significant trauma (HCFA,487)
Potassium chloride (045308,338070541)
Linear rep lid lacer (881)
Suture ext ear lac (184)
Clos reduction nasal fx (2171)
Alcohol abuse-unspec (30500)
accid
lac
lacer
Multiple significant trauma w/o o.r. procedure (APR,9303)
Multiple significant trauma w/o o.r. procedure (APR,9303)
Lung contusion-closed (86121)
mvc
Fx orbital floor-closed (8026)
Elevate skull fx fragmnt (202)
Open reduct mandible fx (7676)
contus
Total splenectomy (415)
Multiple significant trauma w/o o.r. procedure (APR,9302)
Open reduct maxillary fx (7674)
Op red-int fix metac/car (7933)
Fx malar/maxillary-close (8024)
Vertebral fx repair (353)
Opn fx red w int fix nec (7939)
Opn reduct malar/zygo fx (7672)
Propofol,propofol (016796,310030050)
Open wound of forehead (87342)
Other multiple significant trauma w mcc (MS,963)
Multiple significant trauma w/o o.r. procedure (APR,9302)
Bacitracin ointment,bacitracin ointment (023143,168001131)

Topic 53

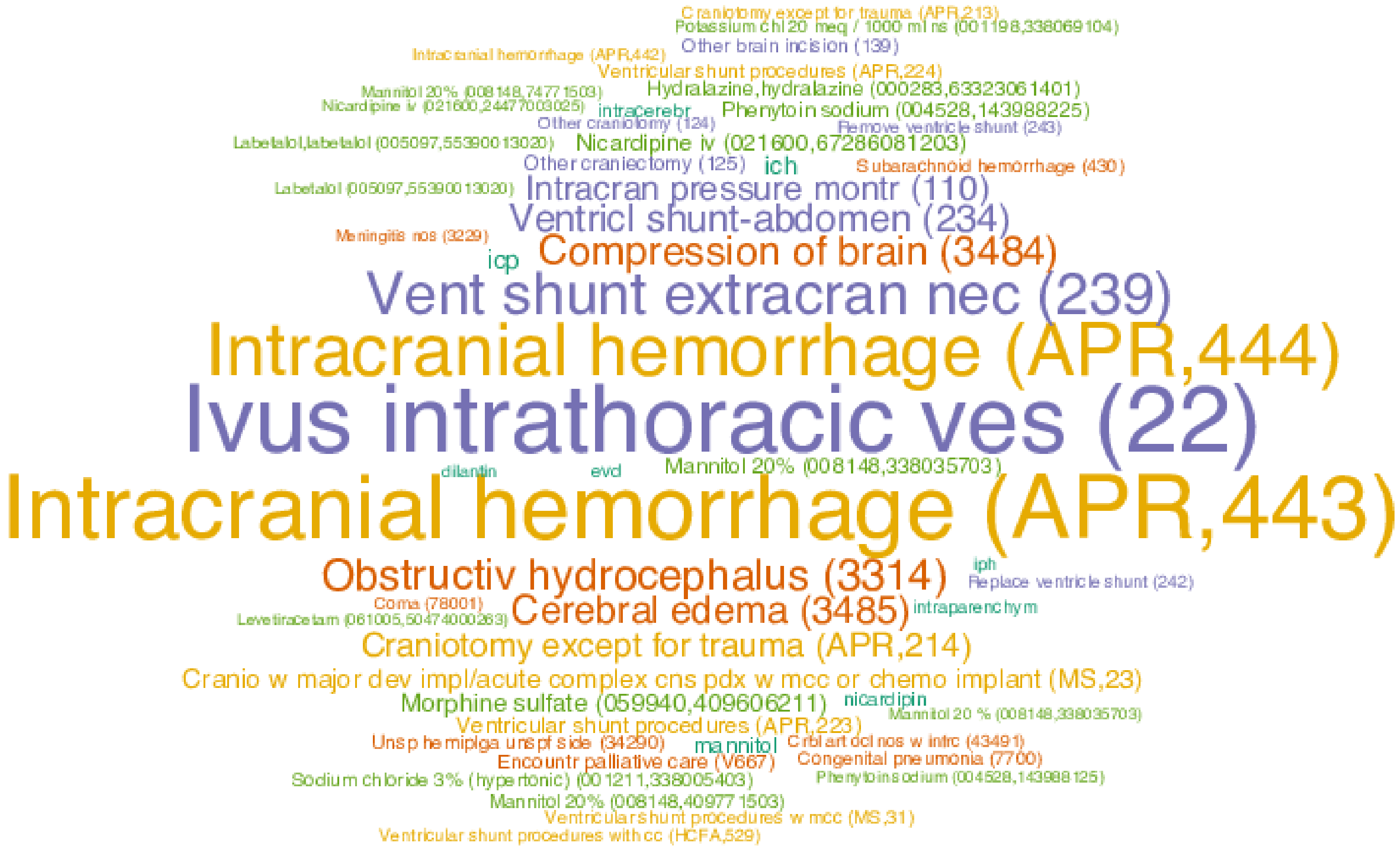
Craniotomy except for trauma (APR,213)
Potassium chl 20 meq / 1000 ml ns (001198,338069104)
Other brain incision (139)
Intracranial hemorrhage (APR,442)
Ventricular shunt procedures (APR,224)
Mannitol 20% (008148,74771503)
Hydralazine,hydralazine (000283,63323061401)
Nicardipine iv (021600,24477003025)
intracerebr
Phenytoin sodium (004528,143988225)
Other craniotomy (124)
Remove ventricle shunt (243)
Labetalol,labetalol (005097,55390013020)
Nicardipine iv (021600,67286081203)
Other craniectomy (125)
ich
Subarachnoid hemorrhage (430)
Labetalol (005097,55390013020)
Intracran pressure montr (110)
Ventricl shunt-abdomen (234)
Meningitis nos (3229)
icp
Compression of brain (3484)
Vent shunt extracran nec (239)
Intracranial hemorrhage (APR,444)
Ivus intrathoracic ves (22)
dilantin
evd
Mannitol 20% (008148,338035703)
Intracranial hemorrhage (APR,443)
iph
Obstructiv hydrocephalus (3314)
Replace ventricle shunt (242)
Coma (78001)
Levetiracetam (061005,50474000263)
Cerebral edema (3485)
intraparenchym
Craniotomy except for trauma (APR,214)
Cranio w major dev impl/acute complex cns pdx w mcc or chemo implant (MS,23)
Morphine sulfate (059940,409606211)
nicardipin
Mannitol 20 % (008148,338035703)
Ventricular shunt procedures (APR,223)
Unsp hemiplga unspf side (34290)
mannitol
Crbl art ocl nos w infrc (43491)
Encountr palliative care (V667)
Congenital pneumonia (7700)
Sodium chloride 3% (hypertonic) (001211,338005403)
Phenytoin sodium (004528,143988125)
Mannitol 20% (008148,409771503)
Ventricular shunt procedures w mcc (MS,31)
Ventricular shunt procedures with cc (HCFA,529)

Topic 54

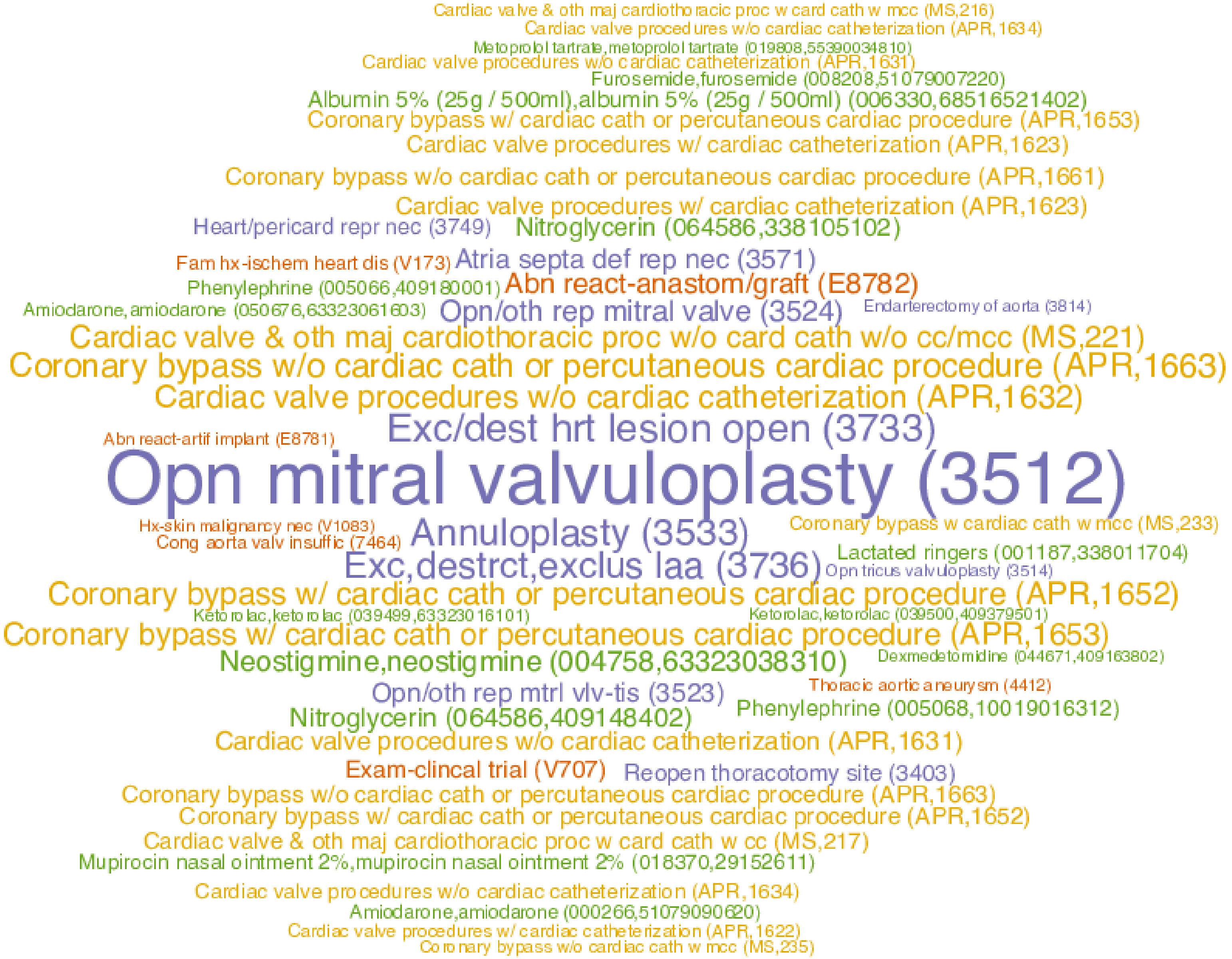
Cardiac valve & oth maj cardiothoracic proc w card cath w mcc (MS,216)
Cardiac valve procedures w/o cardiac catheterization (APR,1634)
Metoprolol tartrate,metoprolol tartrate (019808,55390034810)
Cardiac valve procedures w/o cardiac catheterization (APR,1631)
Furosemide,furosemide (008208,51079007220)
Albumin 5% (25g / 500ml),albumin 5% (25g / 500ml) (006330,68516521402)
Coronary bypass w/ cardiac cath or percutaneous cardiac procedure (APR,1653)
Cardiac valve procedures w/ cardiac catheterization (APR,1623)
Coronary bypass w/o cardiac cath or percutaneous cardiac procedure (APR,1661)
Cardiac valve procedures w/ cardiac catheterization (APR,1623)
Heart/pericard repr nec (3749)
Nitroglycerin (064586,338105102)
Fam hx-ischem heart dis (V173)
Atria septa def rep nec (3571)
Phenylephrine (005066,409180001)
Abn react-anastom/graft (E8782)
Amiodarone,amiodarone (050676,63323061603)
Opn/oth rep mitral valve (3524)
Endarterectomy of aorta (3814)
Cardiac valve & oth maj cardiothoracic proc w/o card cath w/o cc/mcc (MS,221)
Coronary bypass w/o cardiac cath or percutaneous cardiac procedure (APR,1663)
Cardiac valve procedures w/o cardiac catheterization (APR,1632)
Exc/dest hrt lesion open (3733)
Abn react-artif implant (E8781)
Opn mitral valvuloplasty (3512)
Hx-skin malignancy nec (V1083)
Cong aorta valv insuffic (7464)
Annuloplasty (3533)
Coronary bypass w cardiac cath w mcc (MS,233)
Lactated ringers (001187,338011704)
Exc,destrct,exclus laa (3736)
Opn tricus valvuloplasty (3514)
Coronary bypass w/ cardiac cath or percutaneous cardiac procedure (APR,1652)
Ketorolac,ketorolac (039499,63323016101)
Ketorolac,ketorolac (039500,409379501)
Coronary bypass w/ cardiac cath or percutaneous cardiac procedure (APR,1653)
Neostigmine,neostigmine (004758,63323038310)
Dexmedetomidine (044671,409163802)
Opn/oth rep mtrl vlv-tis (3523)
Thoracic aortic aneurysm (4412)
Nitroglycerin (064586,409148402)
Phenylephrine (005068,10019016312)
Cardiac valve procedures w/o cardiac catheterization (APR,1631)
Exam-clincal trial (V707)
Reopen thoracotomy site (3403)
Coronary bypass w/o cardiac cath or percutaneous cardiac procedure (APR,1663)
Coronary bypass w/ cardiac cath or percutaneous cardiac procedure (APR,1652)
Cardiac valve & oth maj cardiothoracic proc w card cath w cc (MS,217)
Mupirocin nasal ointment 2%,mupirocin nasal ointment 2% (018370,29152611)
Cardiac valve procedures w/o cardiac catheterization (APR,1634)
Amiodarone,amiodarone (000266,51079090620)
Cardiac valve procedures w/ cardiac catheterization (APR,1622)
Coronary bypass w/o cardiac cath w mcc (MS,235)

Topic 55

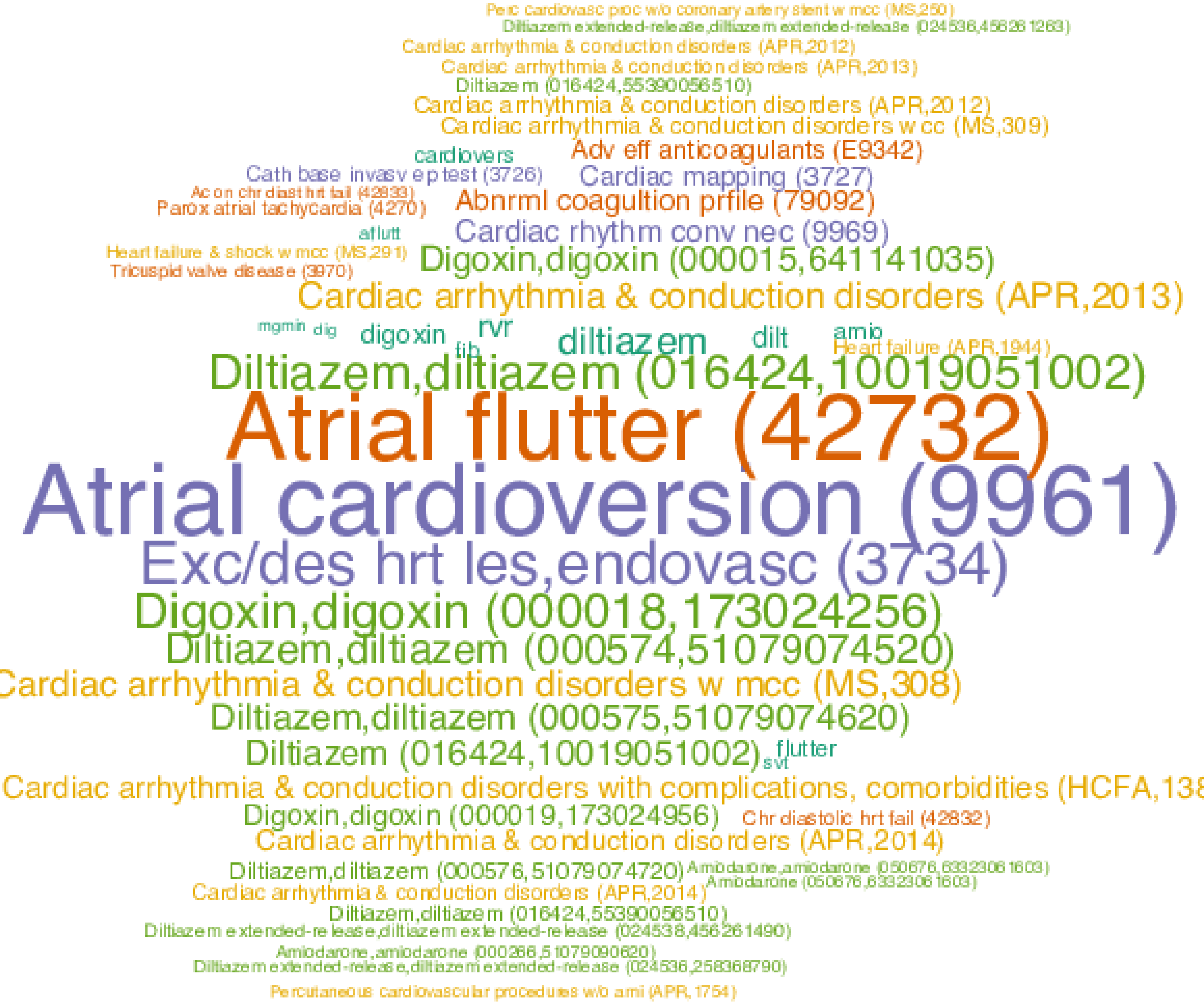
Perc cardiovasc proc w/o coronary artery stent w mcc (MS,250)
Diltiazem extended-release,diltiazem extended-release (024536,456261263)
Cardiac arrhythmia & conduction disorders (APR,2012)
Cardiac arrhythmia & conduction disorders (APR,2013)
Diltiazem (016424,55390056510)
Cardiac arrhythmia & conduction disorders (APR,2012)
Cardiac arrhythmia & conduction disorders w cc (MS,309)
cardiovers
Adv eff anticoagulants (E9342)
Cath base invasv ep test (3726)
Cardiac mapping (3727)
Ac on chr diast hrt fail (42833)
Abnrml coagultion prfile (79092)
Parox atrial tachycardia (4270)
aflutt
Cardiac rhythm conv nec (9969)
Heart failure & shock w mcc (MS,291)
Digoxin,digoxin (000015,641141035)
Tricuspid valve disease (3970)
Cardiac arrhythmia & conduction disorders (APR,2013)
mgmin
dig
digoxin
rvr
fib
diltiazem
dilt
amio
Heart failure (APR,1944)
Diltiazem,diltiazem (016424,10019051002)
Atrial flutter (42732)
Atrial cardioversion (9961)
Exc/des hrt les,endovasc (3734)
Digoxin,digoxin (000018,173024256)
Diltiazem,diltiazem (000574,51079074520)
Cardiac arrhythmia & conduction disorders w mcc (MS,308)
Diltiazem,diltiazem (000575,51079074620)
Diltiazem (016424,10019051002)
svt
flutter
Cardiac arrhythmia & conduction disorders with complications, comorbidities (HCFA,138
Digoxin,digoxin (000019,173024956)
Chr diastolic hrt fail (42832)
Cardiac arrhythmia & conduction disorders (APR,2014)
Diltiazem,diltiazem (000576,51079074720)
Amiodarone,amiodarone (050676,63323061603)
Amiodarone (050676,63323061603)
Cardiac arrhythmia & conduction disorders (APR,2014)
Diltiazem,diltiazem (016424,55390056510)
Diltiazem extended-release,diltiazem extended-release (024538,456261490)
Amiodarone,amiodarone (000266,51079090620)
Diltiazem extended-release,diltiazem extended-release (024536,258368790)
Percutaneous cardiovascular procedures w/o ami (APR,1754)

Topic 56

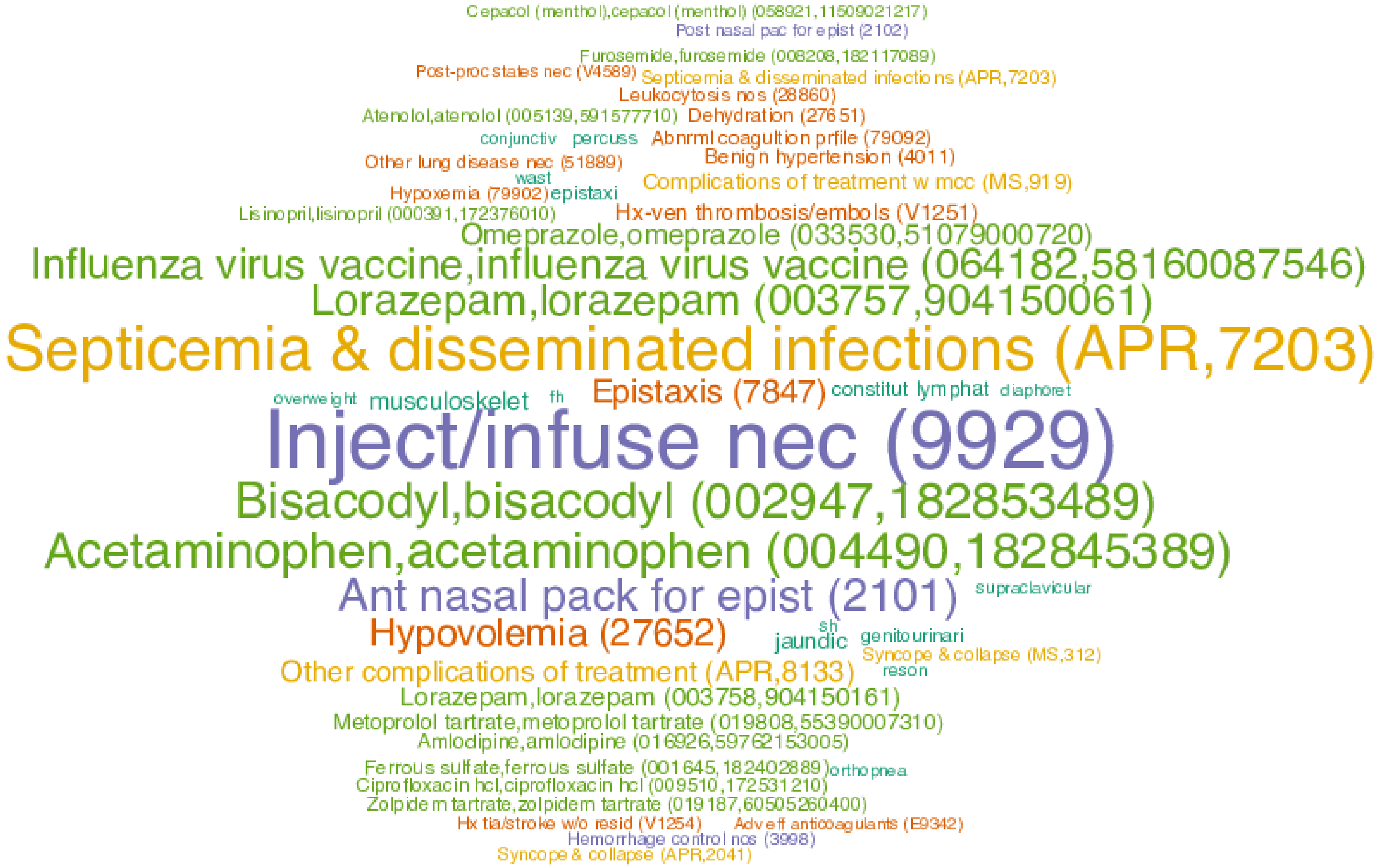

Cepacol (menthol),cepacol (menthol) (058921,11509021217)
Post nasal pac for epist (2102)
Furosemide,furosemide (008208,182117089)
Post-proc states nec (V4589)
Septicemia & disseminated infections (APR,7203)
Leukocytosis nos (28860)
Atenolol,atenolol (005139,591577710)
Dehydration (27651)
conjunctiv
percuss
Abnrml coagultion prfile (79092)
Benign hypertension (4011)
Other lung disease nec (51889)
wast
Complications of treatment w mcc (MS,919)
Hypoxemia (79902)
epistaxi
Lisinopril,lisinopril (000391,172376010)
Hx-ven thrombosis/embols (V1251)
Omeprazole,omeprazole (033530,51079000720)
Influenza virus vaccine,influenza virus vaccine (064182,58160087546)
Lorazepam,lorazepam (003757,904150061)
Septicemia & disseminated infections (APR,7203)
overweight
musculoskelet
fh
Epistaxis (7847)
constitut
lymphat
diaphoret
Inject/infuse nec (9929)
Bisacodyl,bisacodyl (002947,182853489)
Acetaminophen,acetaminophen (004490,182845389)
Ant nasal pack for epist (2101)
supraclavicular
Hypovolemia (27652)
sh
jaundic
genitourinari
Syncope & collapse (MS,312)
Other complications of treatment (APR,8133)
reson
Lorazepam,lorazepam (003758,904150161)
Metoprolol tartrate,metoprolol tartrate (019808,55390007310)
Amlodipine,amlodipine (016926,59762153005)
Ferrous sulfate,ferrous sulfate (001645,182402889)
orthopnea
Ciprofloxacin hcl,ciprofloxacin hcl (009510,172531210)
Zolpidem tartrate,zolpidem tartrate (019187,60505260400)
Hx tia/stroke w/o resid (V1254)
Adv eff anticoagulants (E9342)
Hemorrhage control nos (3998)
Syncope & collapse (APR,2041)

Topic 57

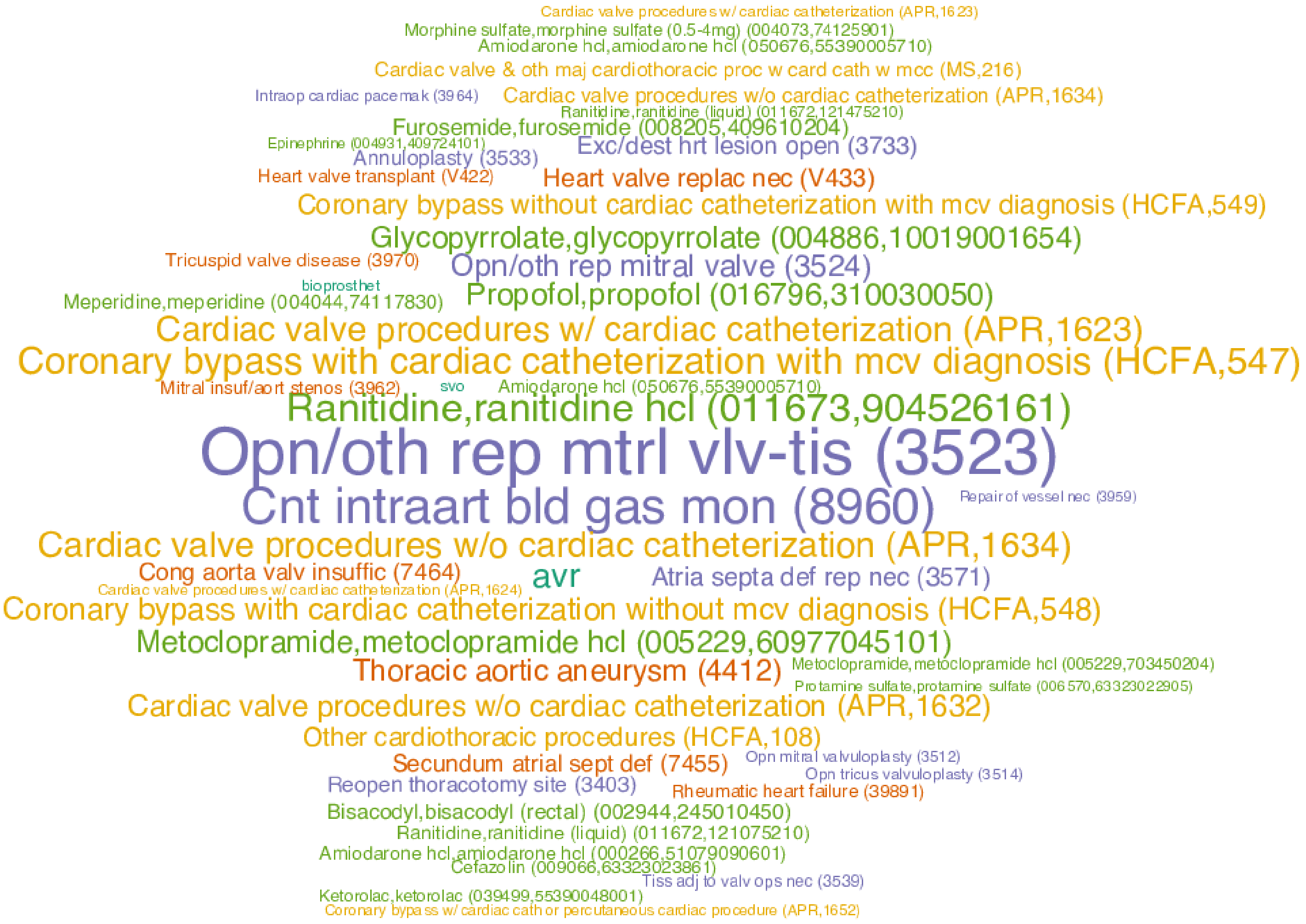
Cardiac valve procedures w/ cardiac catheterization (APR,1623)
Morphine sulfate,morphine sulfate (0.5-4mg) (004073,74125901)
Amiodarone hcl,amiodarone hcl (050676,55390005710)
Cardiac valve & oth maj cardiothoracic proc w card cath w mcc (MS,216)
Intraop cardiac pacemak (3964)
Cardiac valve procedures w/o cardiac catheterization (APR,1634)
Ranitidine,ranitidine (liquid) (011672,121475210)
Furosemide,furosemide (008205,409610204)
Epinephrine (004931,409724101)
Exc/dest hrt lesion open (3733)
Annuloplasty (3533)
Heart valve transplant (V422)
Heart valve replac nec (V433)
Coronary bypass without cardiac catheterization with mcv diagnosis (HCFA,549)
Glycopyrrolate,glycopyrrolate (004886,10019001654)
Tricuspid valve disease (3970)
Opn/oth rep mitral valve (3524)
bioprosthet
Meperidine,meperidine (004044,74117830)
Propofol,propofol (016796,310030050)
Cardiac valve procedures w/ cardiac catheterization (APR,1623)
Coronary bypass with cardiac catheterization with mcv diagnosis (HCFA,547)
Mitral insuf/aort stenos (3962)
svo
Amiodarone hcl (050676,55390005710)
Ranitidine,ranitidine hcl (011673,904526161)
Opn/oth rep mtrl vlv-tis (3523)
Cnt intraart bld gas mon (8960)
Repair of vessel nec (3959)
Cardiac valve procedures w/o cardiac catheterization (APR,1634)
Cong aorta valv insuffic (7464)
avr
Atria septa def rep nec (3571)
Cardiac valve procedures w/ cardiac catheterization (APR,1624)
Coronary bypass with cardiac catheterization without mcv diagnosis (HCFA,548)
Metoclopramide,metoclopramide hcl (005229,60977045101)
Thoracic aortic aneurysm (4412)
Metoclopramide,metoclopramide hcl (005229,703450204)
Protamine sulfate,protamine sulfate (006570,63323022905)
Cardiac valve procedures w/o cardiac catheterization (APR,1632)
Other cardiothoracic procedures (HCFA,108)
Secundum atrial sept def (7455)
Opn mitral valvuloplasty (3512)
Opn tricus valvuloplasty (3514)
Reopen thoracotomy site (3403)
Rheumatic heart failure (39891)
Bisacodyl,bisacodyl (rectal) (002944,245010450)
Ranitidine,ranitidine (liquid) (011672,121075210)
Amiodarone hcl,amiodarone hcl (000266,51079090601)
Cefazolin (009066,63323023861)
Tiss adj to valv ops nec (3539)
Ketorolac,ketorolac (039499,55390048001)
Coronary bypass w/ cardiac cath or percutaneous cardiac procedure (APR,1652)

Topic 58

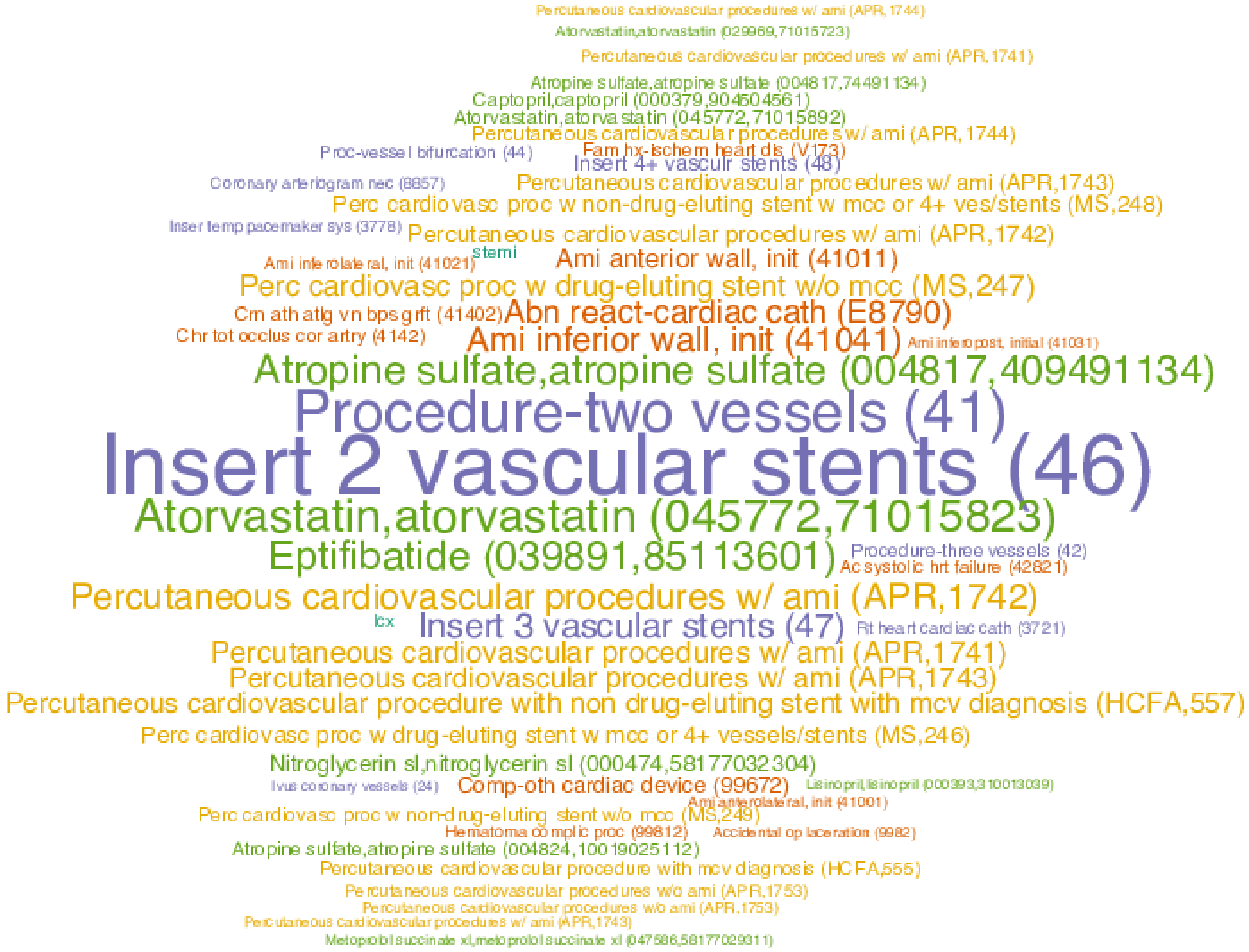

Percutaneous cardiovascular procedures w/ ami (APR,1744)
Atorvastatin,atorvastatin (029969,71015723)
Percutaneous cardiovascular procedures w/ ami (APR,1741)
Atropine sulfate,atropine sulfate (004817,74491134)
Captopril,captopril (000379,904504561)
Atorvastatin,atorvastatin (045772,71015892)
Percutaneous cardiovascular procedures w/ ami (APR,1744)
Proc-vessel bifurcation (44)
Fam hx-ischem heart dis (V173)
Insert 4+ vasculr stents (48)
Coronary arteriogram nec (8857)
Percutaneous cardiovascular procedures w/ ami (APR,1743)
Perc cardiovasc proc w non-drug-eluting stent w mcc or 4+ ves/stents (MS,248)
Inser temp pacemaker sys (3778)
Percutaneous cardiovascular procedures w/ ami (APR,1742)
Ami inferolateral, init (41021)
stemi
Ami anterior wall, init (41011)
Perc cardiovasc proc w drug-eluting stent w/o mcc (MS,247)
Crn ath atlg vn bps grft (41402)
Abn react-cardiac cath (E8790)
Chr tot occlus cor artry (4142)
Ami inferior wall, init (41041)
Ami inferopost, initial (41031)
Atropine sulfate,atropine sulfate (004817,409491134)
Procedure-two vessels (41)
Insert 2 vascular stents (46)
Atorvastatin,atorvastatin (045772,71015823)
Eptifibatide (039891,85113601)
Procedure-three vessels (42)
Acsystolic hrt failure (42821)
Percutaneous cardiovascular procedures w/ ami (APR,1742)
lcx
Insert 3 vascular stents (47)
Rt heart cardiac cath (3721)
Percutaneous cardiovascular procedures w/ ami (APR,1741)
Percutaneous cardiovascular procedures w/ ami (APR,1743)
Percutaneous cardiovascular procedure with non drug-eluting stent with mcv diagnosis (HCFA,557)
Perc cardiovasc proc w drug-eluting stent w mcc or 4+ vessels/stents (MS,246)
Nitroglycerin sl,nitroglycerin sl (000474,58177032304)
Ivus coronary vessels (24)
Comp-oth cardiac device (99672)
Lisinopril,lisinopril (000093,310013039)
Ami anterolateral, init (41001)
Perc cardiovasc proc w non-drug-eluting stent w/o mcc (MS,249)
Hematoma complic proc (99812)
Accidental op laceration (9982)
Atropine sulfate,atropine sulfate (004824,10019025112)
Percutaneous cardiovascular procedure with mcv diagnosis (HCFA,555)
Percutaneous cardiovascular procedures w/o ami (APR,1753)
Percutaneous cardiovascular procedures w/o ami (APR,1753)
Percutaneous cardiovascular procedures w/ ami (APR,1743)
Metoprolol succinate xl,metoprolol succinate xl (047586,58177029311)

Topic 59

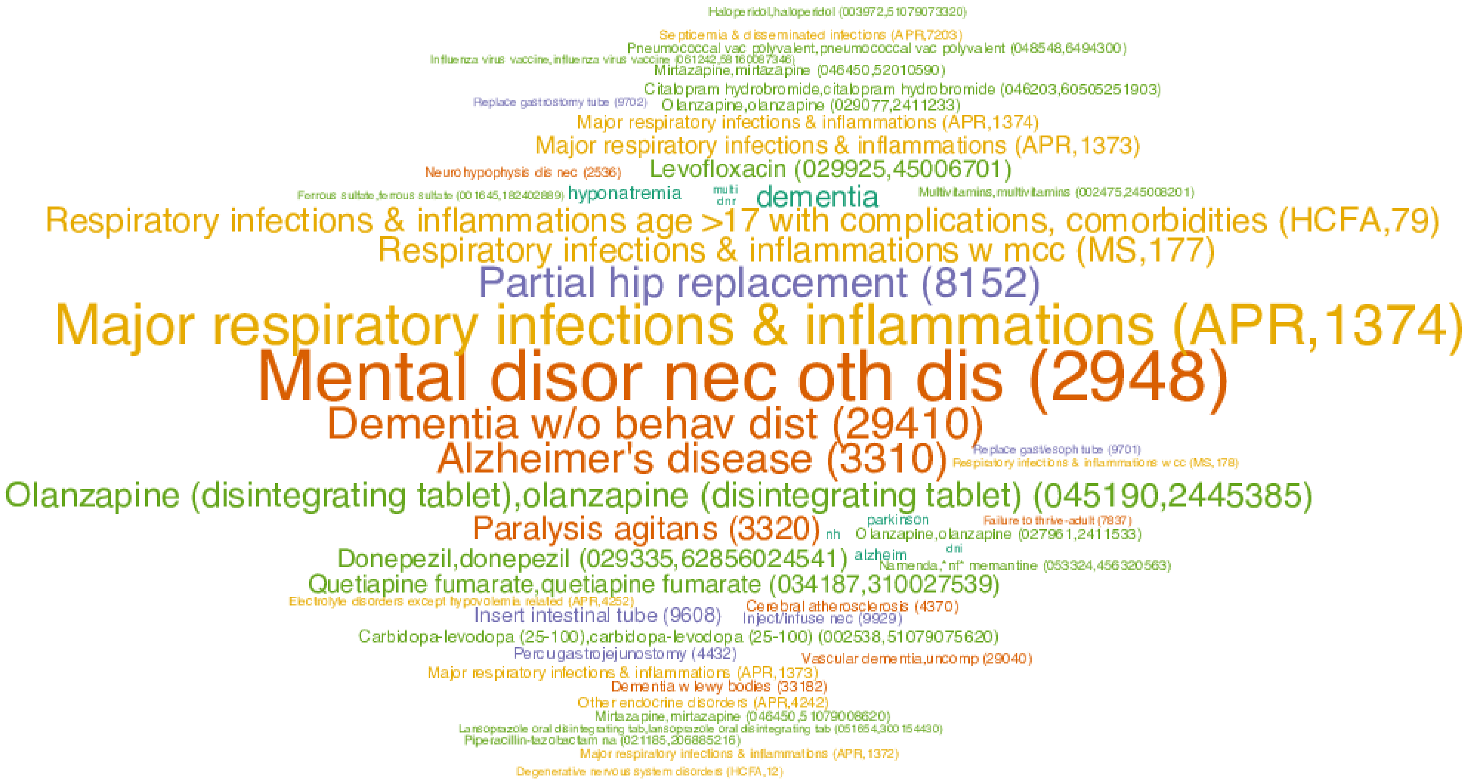
Haloperidol,haloperidol (003972,51079073320)
Septicemia & disseminated infections (APR,7203)
Pneumococcal vac polyvalent,pneumococcal vac polyvalent (048548,6494300)
Influenza virus vaccine,influenza virus vaccine (061242,58160087346)
Mirtazapine,mirtazapine (046450,52010590)
Citalopram hydrobromide,citalopram hydrobromide (046203,60505251903)
Replace gastrostomy tube (9702)
Olanzapine,olanzapine (029077,2411233)
Major respiratory infections & inflammations (APR,1374)
Major respiratory infections & inflammations (APR,1373)
Neurohypophysis dis nec (2536)
Levofloxacin (029925,45006701)
Ferrous sulfate,ferrous sulfate (001645,182402889)
hyponatremia
multi
dnr
dementia
Multivitamins,multivitamins (002475,245008201)
Respiratory infections & inflammations age >17 with complications, comorbidities (HCFA,79)
Respiratory infections & inflammations w mcc (MS,177)
Partial hip replacement (8152)
Major respiratory infections & inflammations (APR,1374)
Mental disor nec oth dis (2948)
Dementia w/o behav dist (29410)
Replace gast/esoph tube (9701)
Alzheimer's disease (3310)
Respiratory infections & inflammations w cc (MS,178)
Olanzapine (disintegrating tablet),olanzapine (disintegrating tablet) (045190,2445385)
Paralysis agitans (3320)
nh
parkinson
Failure to thrive-adult (7837)
Olanzapine,olanzapine (027961,2411533)
dni
Donepezil,donepezil (029335,62856024541)
alzheim
Namenda,*nf* memantine (053324,456320563)
Quetiapine fumarate,quetiapine fumarate (034187,310027539)
Electrolyte disorders except hypovolemia related (APR,4252)
Cerebral atherosclerosis (4370)
Insert intestinal tube (9608)
Inject/infuse nec (9929)
Carbidopa-levodopa (25-100),carbidopa-levodopa (25-100) (002538,51079075620)
Percugastrojejunostomy (4432)
Vascular dementia,uncomp (29040)
Major respiratory infections & inflammations (APR,1373)
Dementia w lewy bodies (33182)
Other endocrine disorders (APR,4242)
Mirtazapine,mirtazapine (046450,51079008620)
Lansoprazole oral disintegrating tab,lansoprazole oral disintegrating tab (051654,300154430)
Piperacillin-tazobactam na (021185,206885216)
Major respiratory infections & inflammations (APR,1372)
Degenerative nervous system disorders (HCFA,12)

Topic 60

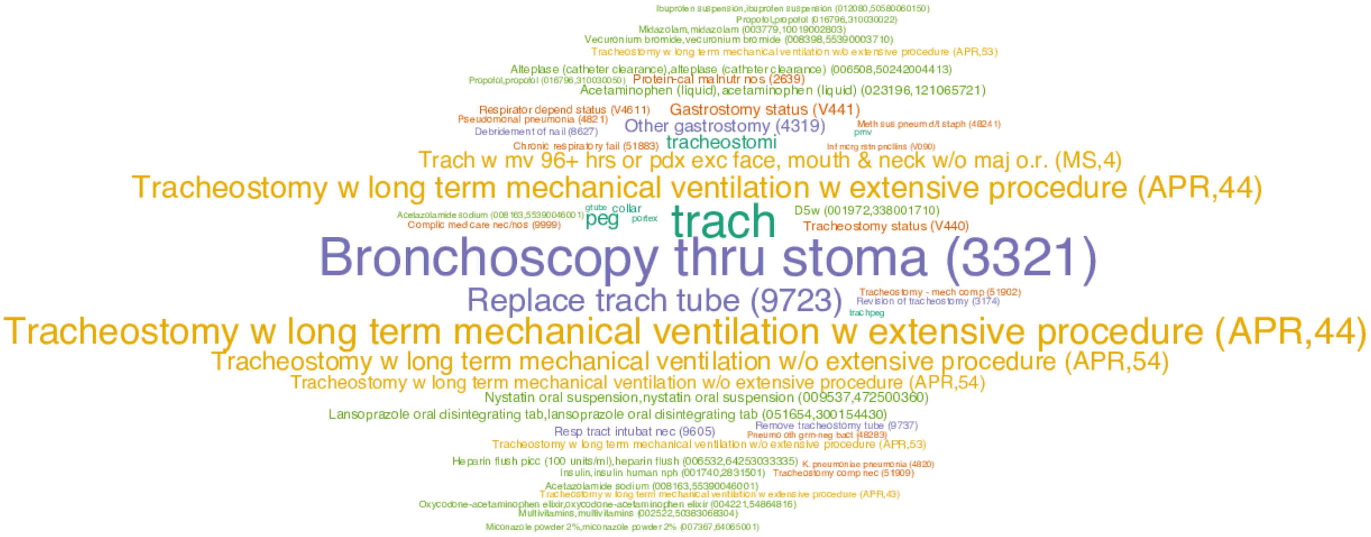

Ibuprofen suspension,ibuprofen suspension (012080,50580060150)
Propofol,propofol (016796,310030022)
Midazolam,midazolam (003779,10019002803)
Vecuronium bromide,vecuronium bromide (008398,55390003710)
Tracheostomy w long term mechanical ventilation w/o extensive procedure (APR,53)
Alteplase (catheter clearance),alteplase (catheter clearance) (006508,50242004413)
Propofol,propofol (016796,310030050)
Protein-cal malnutr nos (2639)
Acetaminophen (liquid),acetaminophen (liquid) (023196,121065721)
Respirator depend status (V4611)
Gastrostomy status (V441)
Pseudomonal pneumonia (4821)
Other gastrostomy (4319)
Meth sus pneum d/t staph (48241)
Debridement of nail (8627)
pmv
Chronic respiratory fail (51883)
tracheostomi
Inf mcrg rstn pnclins (V090)
Trach w mv 96+ hrs or pdx exc face, mouth & neck w/o maj o.r. (MS,4)
Tracheostomy w long term mechanical ventilation w extensive procedure (APR,44)
Acetazolamide sodium (008163,55390046001)
Complic med care nec/nos (9999)
gtube
collar
peg
portex
trach
D5w (001972,338001710)
Tracheostomy status (V440)
Bronchoscopy thru stoma (3321)
Replace trach tube (9723)
Tracheostomy - mech comp (51902)
Revision of tracheostomy (3174)
trachpeg
Tracheostomy w long term mechanical ventilation w extensive procedure (APR,44)
Tracheostomy w long term mechanical ventilation w/o extensive procedure (APR,54)
Tracheostomy w long term mechanical ventilation w/o extensive procedure (APR,54)
Nystatin oral suspension,nystatin oral suspension (009537,472500360)
Lansoprazole oral disintegrating tab,lansoprazole oral disintegrating tab (051654,300154430)
Remove tracheostomy tube (9737)
Resp tract intubat nec (9605)
Pneumo oth grm-neg bact (48283)
Tracheostomy w long term mechanical ventilation w/o extensive procedure (APR,53)
Heparin flush picc (100 units/ml),heparin flush (006532,64253033335)
K. pneumoniae pneumonia (4820)
Insulin,insulin human nph (001740,2831501)
Tracheostomy comp nec (51909)
Acetazolamide sodium (008163,55390046001)
Tracheostomy w long term mechanical ventilation w extensive procedure (APR,43)
Oxycodone-acetaminophen elixir,oxycodone-acetaminophen elixir (004221,54864816)
Multivitamins,multivitamins (002522,50383068304)
Miconazole powder 2%,miconazole powder 2% (007367,64065001)

Topic 61

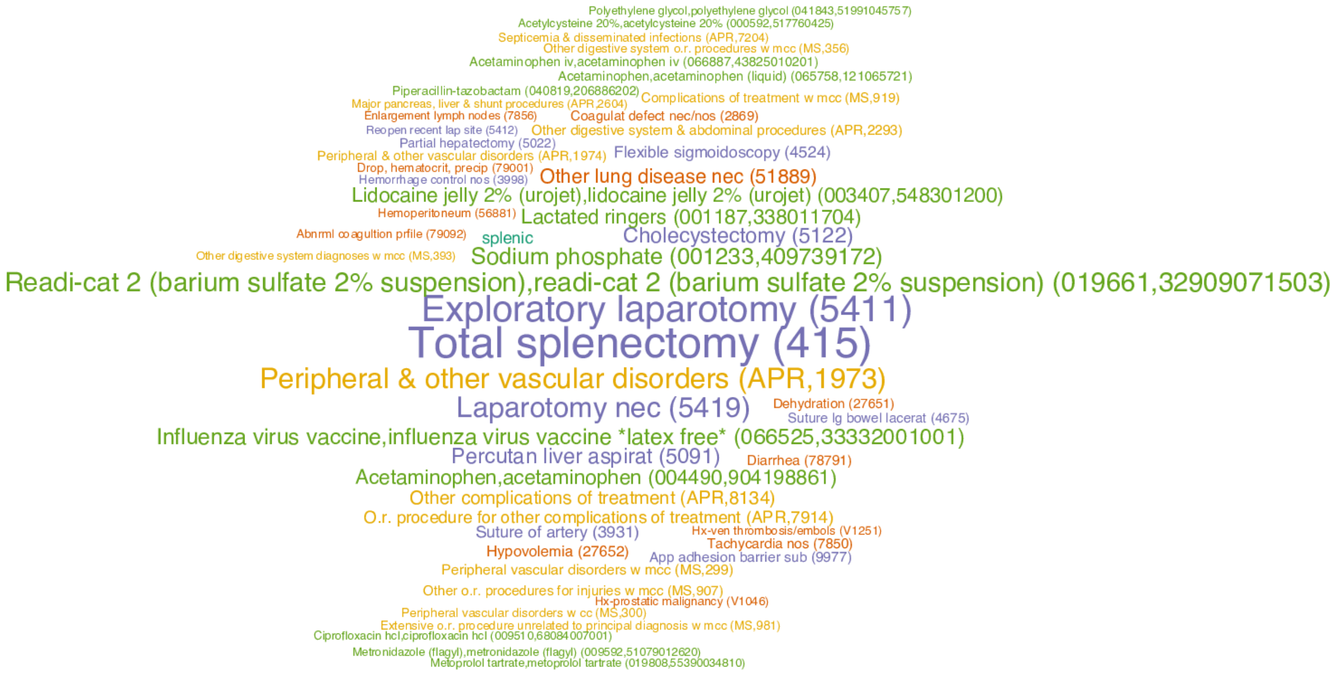

Polyethylene glycol,polyethylene glycol (041843,51991045757)
Acetylcysteine 20%,acetylcysteine 20% (000592,517760425)
Septicemia & disseminated infections (APR,7204)
Other digestive system o.r. procedures w mcc (MS,356)
Acetaminophen iv,acetaminophen iv (066887,43825010201)
Acetaminophen,acetaminophen (liquid) (065758,121065721)
Piperacillin-tazobactam (040819,206886202)
Complications of treatment w mcc (MS,919)
Major pancreas, liver & shunt procedures (APR,2604)
Enlargement lymph nodes (7856)
Coagulat defect nec/nos (2869)
Reopen recent lap site (5412)
Other digestive system & abdominal procedures (APR,2293)
Partial hepatectomy (5022)
Peripheral & other vascular disorders (APR,1974)
Flexible sigmoidoscopy (4524)
Drop, hematocrit, precip (79001)
Hemorrhage control nos (3998)
Other lung disease nec (51889)
Lidocaine jelly 2% (urojet),lidocaine jelly 2% (urojet) (003407,548301200)
Hemoperitoneum (56881)
Lactated ringers (001187,338011704)
Abnrml coagultion prfile (79092)
splenic
Cholecystectomy (5122)
Other digestive system diagnoses w mcc (MS,393)
Sodium phosphate (001233,409739172)
Readi-cat 2 (barium sulfate 2% suspension),readi-cat 2 (barium sulfate 2% suspension) (019661,32909071503)
Exploratory laparotomy (5411)
Total splenectomy (415)
Peripheral & other vascular disorders (APR,1973)
Laparotomy nec (5419)
Dehydration (27651)
Suture lg bowel lacerat (4675)
Influenza virus vaccine,influenza virus vaccine *latex free* (066525,33332001001)
Percutan liver aspirat (5091)
Diarrhea (78791)
Acetaminophen,acetaminophen (004490,904198861)
Other complications of treatment (APR,8134)
O.r. procedure for other complications of treatment (APR,7914)
Suture of artery (3931)
Hx-ven thrombosis/embols (V1251)
Tachycardia nos (7850)
Hypovolemia (27652)
App adhesion barrier sub (9977)
Peripheral vascular disorders w mcc (MS,299)
Other o.r. procedures for injuries w mcc (MS,907)
Hx-prostatic malignancy (V1046)
Peripheral vascular disorders w cc (MS,300)
Extensive o.r. procedure unrelated to principal diagnosis w mcc (MS,981)
Ciprofloxacin hcl,ciprofloxacin hcl (009510,68084007001)
Metronidazole (flagyl),metronidazole (flagyl) (009592,51079012620)
Metoprolol tartrate,metoprolol tartrate (019808,55390034810)

Topic 62

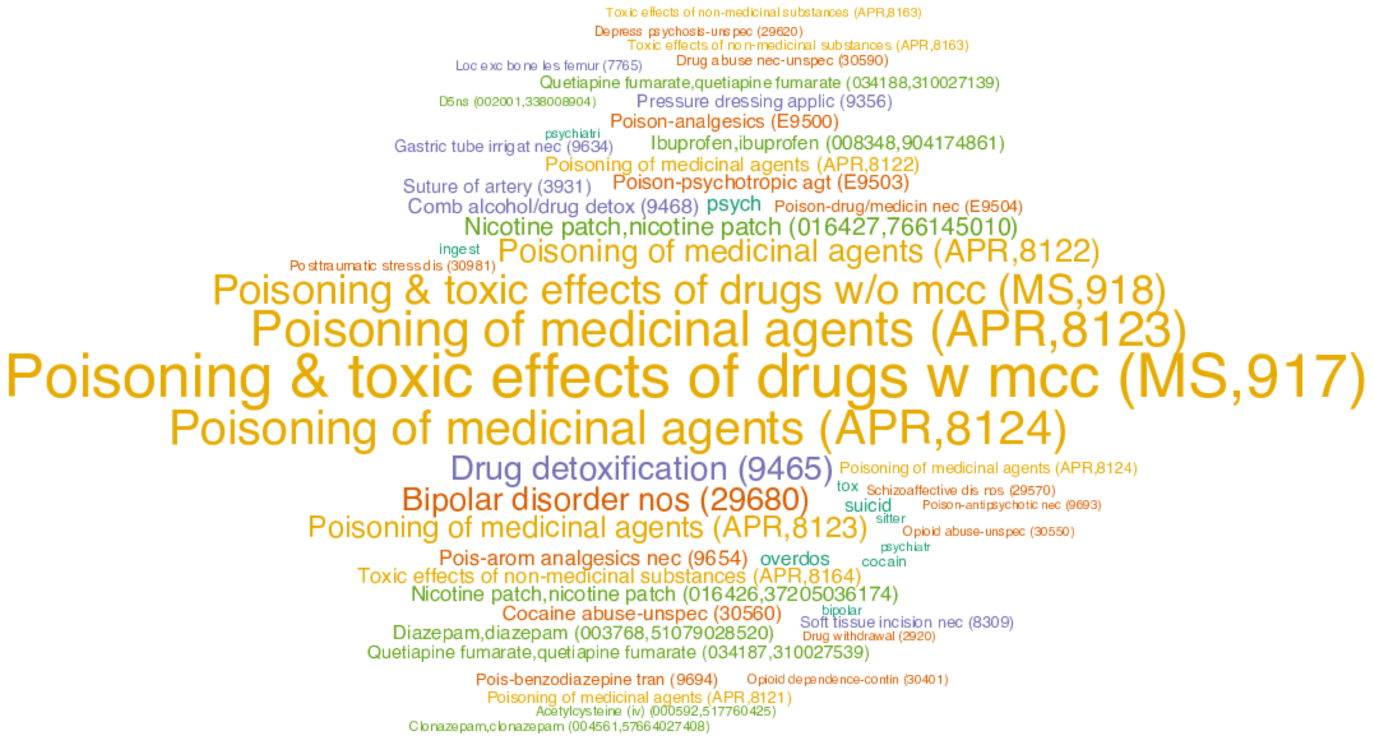
Toxic effects of non-medicinal substances (APR,8163)
Depress psychosis-unspec (29620)
Toxic effects of non-medicinal substances (APR,8163)
Loc exc bone les femur (7765)
Drug abuse nec-unspec (30590)
Quetiapine fumarate,quetiapine fumarate (034188,310027139)
D5ns (002001,338008904)
Pressure dressing applic (9356)
Poison-analgesics (E9500)
psychiatri
Gastric tube irrigat nec (9634)
Ibuprofen,ibuprofen (008348,904174861)
Poisoning of medicinal agents (APR,8122)
Suture of artery (3931)
Poison-psychotropic agt (E9503)
Comb alcohol/drug detox (9468)
psych
Poison-drug/medicin nec (E9504)
Nicotine patch,nicotine patch (016427,766145010)
ingest
Posttraumatic stress dis (30981)
Poisoning of medicinal agents (APR,8122)
Poisoning & toxic effects of drugs w/o mcc (MS,918)
Poisoning of medicinal agents (APR,8123)
Poisoning & toxic effects of drugs w mcc (MS,917)
Poisoning of medicinal agents (APR,8124)
Drug detoxification (9465)
Poisoning of medicinal agents (APR,8124)
tox
Schizoaffective dis nos (29570)
Bipolar disorder nos (29680)
suicid
Poison-antipsychotic nec (9693)
sitter
Poisoning of medicinal agents (APR,8123)
Opioid abuse-unspec (30550)
psychiatr
Pois-arom analgesics nec (9654)
overdos
cocain
Toxic effects of non-medicinal substances (APR,8164)
Nicotine patch,nicotine patch (016426,37205036174)
Cocaine abuse-unspec (30560)
bipolar
Soft tissue incision nec (8309)
Diazepam,diazepam (003768,51079028520)
Drug withdrawal (2920)
Quetiapine fumarate,quetiapine fumarate (034187,310027539)
Pois-benzodiazepine tran (9694)
Opioid dependence-contin (30401)
Poisoning of medicinal agents (APR,8121)
Acetylcysteine (iv) (000592,517760425)
Clonazepam,clonazepam (004561,57664027408)

Topic 63

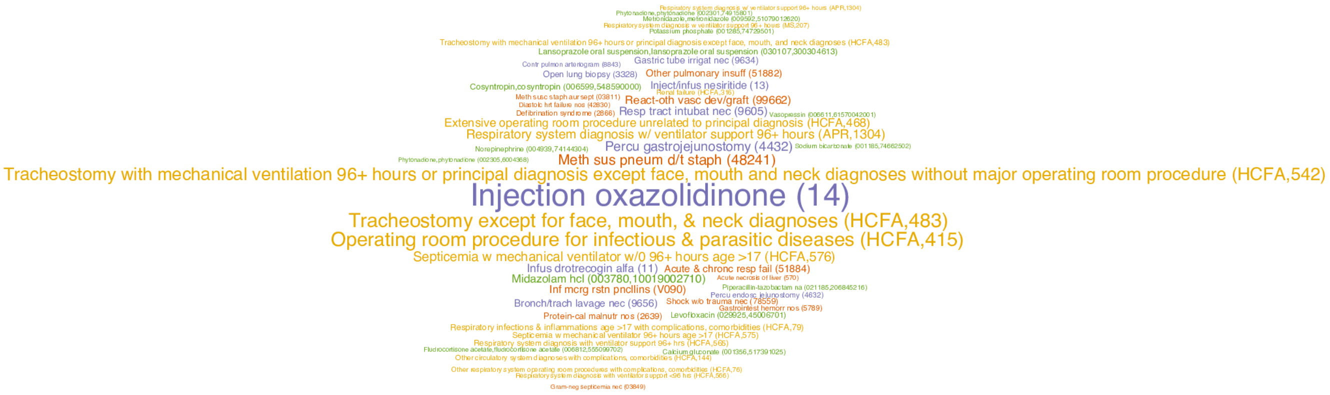
Respiratory system diagnosis w/ ventilator support 96+ hours (APR,1304)
Phytonadione,phytonadione (002301,74915801)
Metronidazole,metronidazole (009592,51079012620)
Respiratory system diagnosis w ventilator support 96+ hours (MS,207)
Potassium phosphate (001285,74729501)
Tracheostomy with mechanical ventilation 96+ hours or principal diagnosis except face, mouth, and neck diagnoses (HCFA,483)
Lansoprazole oral suspension,lansoprazole oral suspension (030107,300304613)
Contr pulmon arteriogram (8843)
Gastric tube irrigat nec (9634)
Open lung biopsy (3328)
Other pulmonary insuff (51882)
Cosyntropin,cosyntropin (006599,548590000)
Inject/infus nesiritide (13)
Renal failure (HCFA,316)
Meth susc staph aur sept (03811)
React-oth vasc dev/graft (99662)
Diastolc hrt failure nos (42830)
Defibrination syndrome (2866)
Resp tract intubat nec (9605)
Vasopressin (006611,61570042001)
Extensive operating room procedure unrelated to principal diagnosis (HCFA,468)
Respiratory system diagnosis w/ ventilator support 96+ hours (APR,1304)
Norepinephrine (004939,74144304)
Percu gastrojejunostomy (4432)
Sodium bicarbonate (001185,74662502)
Phytonadione,phytonadione (002305,6004368)
Meth sus pneum d/t staph (48241)
Tracheostomy with mechanical ventilation 96+ hours or principal diagnosis except face, mouth and neck diagnoses without major operating room procedure (HCFA,542)
Injection oxazolidinone (14)
Tracheostomy except for face, mouth, & neck diagnoses (HCFA,483)
Operating room procedure for infectious & parasitic diseases (HCFA,415)
Septicemia w mechanical ventilator w/0 96+ hours age >17 (HCFA,576)
Infus drotrecogin alfa (11)
Acute & chronc resp fail (51884)
Midazolam hcl (003780,10019002710)
Acute necrosis of liver (570)
Inf mcrg rstn pncllins (V090)
Piperacillin-tazobactam na (021185,206845216)
Percu endosc jejunostomy (4632)
Bronch/trach lavage nec (9656)
Shock w/o trauma nec (78559)
Gastrointest hemorr nos (5789)
Protein-cal malnutr nos (2639)
Levofloxacin (029925,45006701)
Respiratory infections & inflammations age >17 with complications, comorbidities (HCFA,79)
Septicemia w mechanical ventilator 96+ hours age >17 (HCFA,575)
Respiratory system diagnosis with ventilator support 96+ hrs (HCFA,565)
Fludrocortisone acetate,fludrocortisone acetate (006812,555099702)
Calcium gluconate (001356,517391025)
Other circulatory system diagnoses with complications, comorbidities (HCFA,144)
Other respiratory system operating room procedures with complications, comorbidities (HCFA,76)
Respiratory system diagnosis with ventilator support <96 hrs (HCFA,566)
Gram-neg septicemia nec (03849)

Topic 64

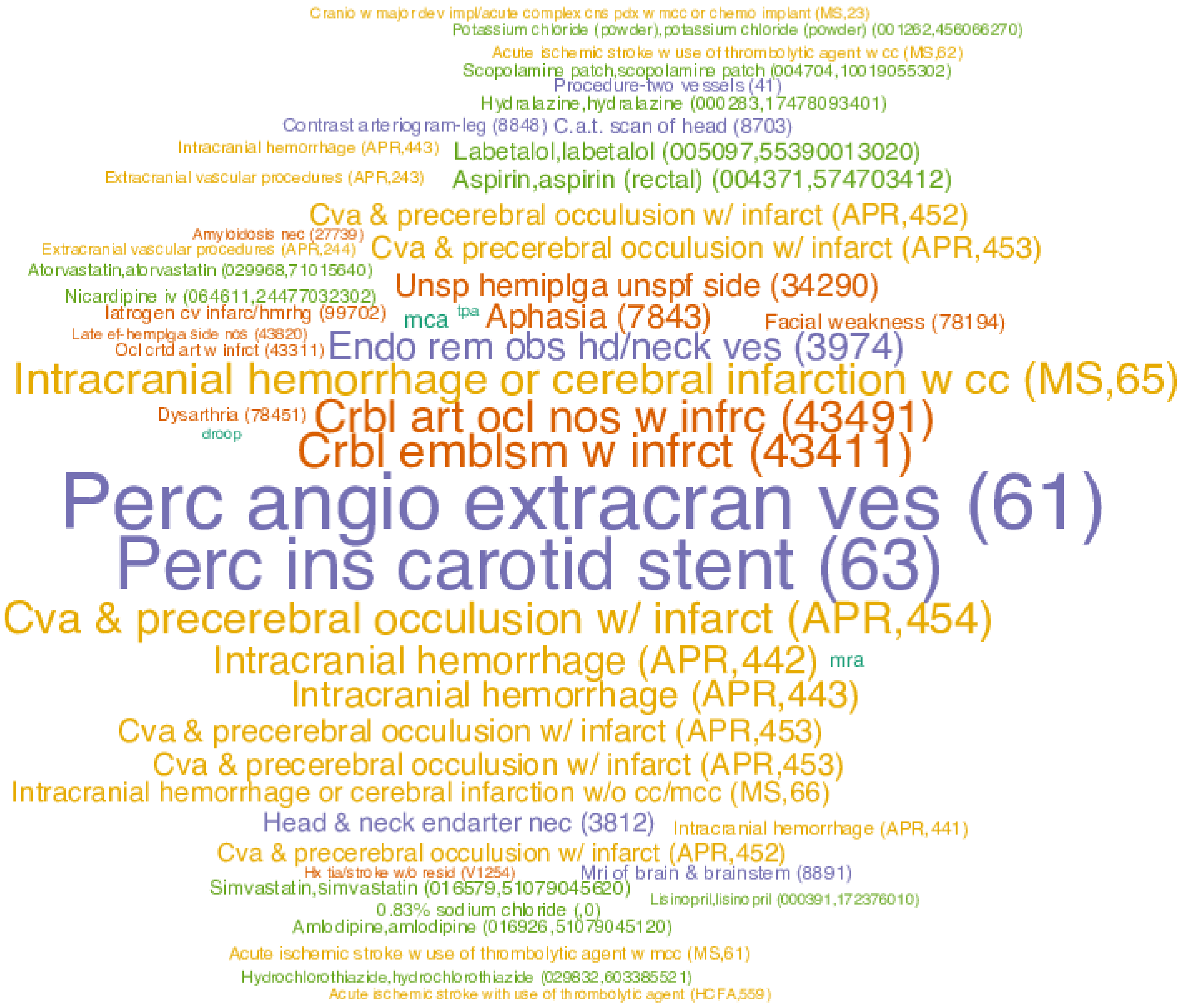
Cranio w major dev impl/acute complex cns pdx w mcc or chemo implant (MS,23)
Potassium chloride (powder),potassium chloride (powder) (001262,456066270)
Acute ischemic stroke w use of thrombolytic agent w cc (MS,62)
Scopolamine patch,scopolamine patch (004704,10019055302)
Procedure-two vessels (41)
Hydralazine,hydralazine (000283,17478093401)
Contrast arteriogram-leg (8848)
C.a.t. scan of head (8703)
Intracranial hemorrhage (APR,443)
Labetalol,labetalol (005097,55390013020)
Extracranial vascular procedures (APR,243)
Aspirin,aspirin (rectal) (004371,574703412)
Cva & precerebral occulusion w/ infarct (APR,452)
Amyloidosis nec (27739)
Extracranial vascular procedures (APR,244)
Cva & precerebral occulusion w/ infarct (APR,453)
Atorvastatin,atorvastatin (029968,71015640)
Unsp hemiplga unspf side (34290)
Nicardipine iv (064611,24477032302)
Iatrogen cv infarc/hmrhg (99702)
mca
tpa
Aphasia (7843)
Facial weakness (78194)
Late ef-hemplga side nos (43820)
Ocl crtd art w infrct (43311)
Endo rem obs hd/neck ves (3974)
Intracranial hemorrhage or cerebral infarction w cc (MS,65)
Dysarthria (78451)
Crbl art ocl nos w infrc (43491)
droop
Crbl emblsm w infrct (43411)
Perc angio extracran ves (61)
Perc ins carotid stent (63)
Cva & precerebral occulusion w/ infarct (APR,454)
Intracranial hemorrhage (APR,442)
mra
Intracranial hemorrhage (APR,443)
Cva & precerebral occulusion w/ infarct (APR,453)
Cva & precerebral occulusion w/ infarct (APR,453)
Intracranial hemorrhage or cerebral infarction w/o cc/mcc (MS,66)
Head & neck endarter nec (3812)
Intracranial hemorrhage (APR,441)
Cva & precerebral occulusion w/ infarct (APR,452)
Hx tia/stroke w/o resid (V1254)
Mri of brain & brainstem (8891)
Simvastatin,simvastatin (016579,51079045620)
Lisinopril,lisinopril (000391,172376010)
0.83% sodium chloride (,0)
Amlodipine,amlodipine (016926,51079045120)
Acute ischemic stroke w use of thrombolytic agent w mcc (MS,61)
Hydrochlorothiazide,hydrochlorothiazide (029832,603385521)
Acute ischemic stroke with use of thrombolytic agent (HCFA,559)

Topic 65

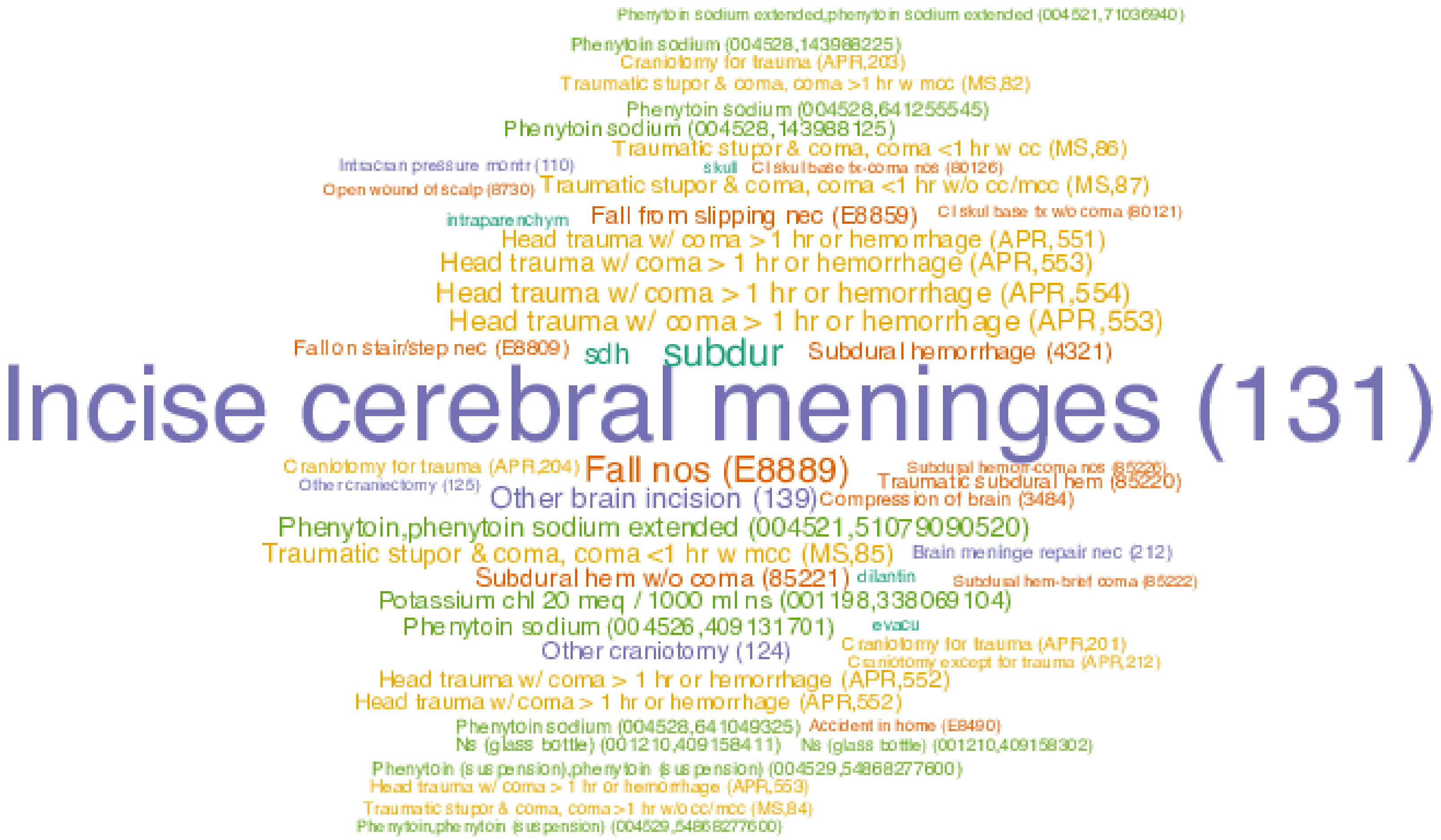

Phenytoin sodium extended,phenytoin sodium extended (004521,71036940)
Phenytoin sodium (004528,143988225)
Craniotomy for trauma (APR,203)
Traumatic stupor & coma, coma >1 hr w mcc (MS,82)
Phenytoin sodium (004528,641255545)
Phenytoin sodium (004528,143988125)
Traumatic stupor & coma, coma <1 hr w cc (MS,86)
Intracran pressure monitr (110)
skull
Cl skul base fx-coma nos (80126)
Open wound of scalp (8730)
Traumatic stupor & coma, coma <1 hr w/o cc/mcc (MS,87)
intraparenchym
Fall from slipping nec (E8859)
Cl skul base fx w/o coma (80121)
Head trauma w/ coma >1 hr or hemorrhage (APR,551)
Head trauma w/ coma > 1 hr or hemorrhage (APR,553)
Head trauma w/ coma > 1 hr or hemorrhage (APR,554)
Head trauma w/ coma > 1 hr or hemorrhage (APR,553)
Fall on stair/step nec (E8809)
sdh
subdur
Subdural hemorrhage (4321)
Incise cerebral meninges (131)
Craniotomy for trauma (APR,204)
Fall nos (E8889)
Subdural hemorr-coma nos (85226)
Other craniectomy (125)
Traumatic subdural hem (85220)
Other brain incision (139)
Compression of brain (3484)
Phenytoin,phenytoin sodium extended (004521,51079090520)
Traumatic stupor & coma, coma <1 hr w mcc (MS,85)
Brain meninge repair nec (212)
Subdural hem w/o coma (85221)
dilantin
Subdural hem-brief coma (85222)
Potassium chl 20 meq / 1000 ml ns (001198,338069104)
Phenytoin sodium (004526,409131701)
evacu
Other craniotomy (124)
Craniotomy for trauma (APR,201)
Craniotomy except for trauma (APR,212)
Head trauma w/ coma > 1 hr or hemorrhage (APR,552)
Head trauma w/ coma > 1 hr or hemorrhage (APR,552)
Phenytoin sodium (004528,641049325)
Accident in home (E8490)
Ns (glass bottle) (001210,409158411)
Ns (glass bottle) (001210,409158302)
Phenytoin (suspension),phenytoin (suspension) (004529,54868277600)
Head trauma w/ coma > 1 hr or hemorrhage (APR,553)
Traumatic stupor & coma, coma >1 hr w/o cc/mcc (MS,84)
Phenytoin,phenytoin (suspension) (004529,54868277600)

Topic 66

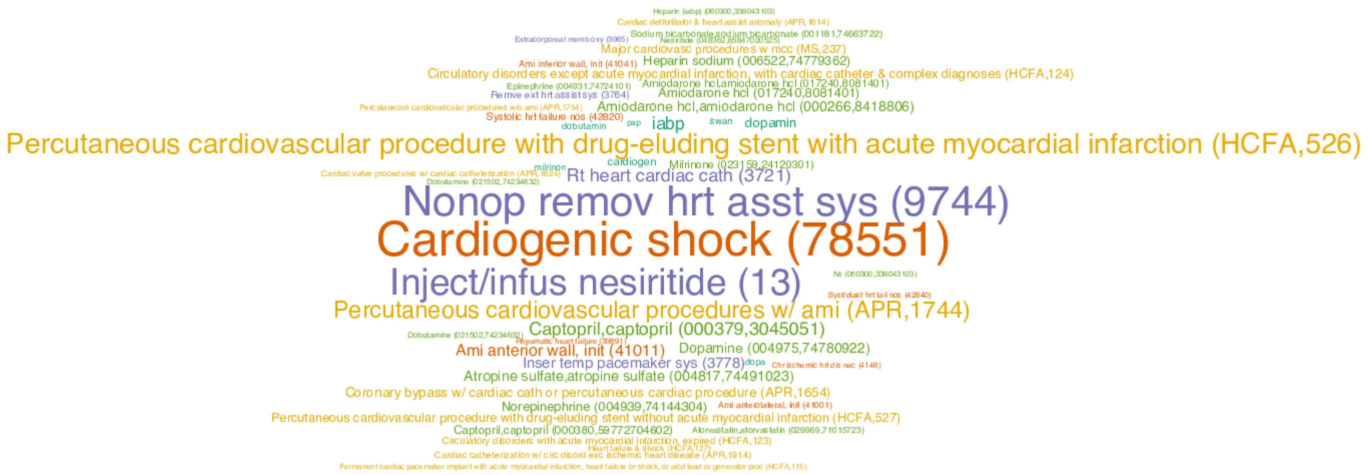
Heparin (iabp) (060300,338043103)
Cardiac defibrillator & heart assist anomaly (APR,1614)
Sodium bicarbonate,sodium bicarbonate (001181,74663722)
Extracorporeal memboxy (3965)
Nesiritide (048362,65847020525)
Major cardiovasc procedures w mcc (MS,237)
Ami inferior wall, init (41041)
Heparin sodium (006522,74779362)
Circulatory disorders except acute myocardial infarction, with cardiac catheter & complex diagnoses (HCFA,124)
Epinephrine (004931,74724101)
Amiodarone hcl,amiodarone hcl (017240,8081401)
Remve ext hrt assist sys (3764)
Amiodarone hcl (017240,8081401)
Percutaneous cardiovascular procedures w/o ami (APR,1754)
Amiodarone hcl,amiodarone hcl (000266,8418806)
Systolic hrt failure nos (42820)
dobutamin
pap
iabp
swan
dopamin
Percutaneous cardiovascular procedure with drug-eluding stent with acute myocardial infarction (HCFA,526)
cardiogen
milrinon
Milrinone (023159,24120301)
Cardiac valve procedures w/ cardiac catheterization (APR,1624)
Rt heart cardiac cath (3721)
Dobutamine (021502,74234632)
Nonop remov hrt asst sys (9744)
Cardiogenic shock (78551)
Inject/infus nesiritide (13)
Ns (060300,338043103)
Syst/diast hrt fail nos (42840)
Percutaneous cardiovascular procedures w/ ami (APR,1744)
Captopril,captopril (000379,3045051)
Dobutamine (021502,74234632)
Rheumatic heart failure (39891)
Ami anterior wall, init (41011)
Dopamine (004975,74780922)
Inser temp pacemaker sys (3778)
dopa
Chr ischemic hrt dis nec (4148)
Atropine sulfate,atropine sulfate (004817,74491023)
Coronary bypass w/ cardiac cath or percutaneous cardiac procedure (APR,1654)
Norepinephrine (004939,74144304)
Ami anterolateral, init (41001)
Percutaneous cardiovascular procedure with drug-eluding stent without acute myocardial infarction (HCFA,527)
Captopril,captopril (000380,59772704602)
Atorvastatin,atorvastatin (029969,71015723)
Circulatory disorders with acute myocardial infarction, expired (HCFA,123)
Heart failure & shock (HCFA,127)
Cardiac catheterization w/ circ disord exc ischemic heart disease (APR,1914)
Permanent cardiac pacemaker implant with acute myocardial infarction, heart failure or shock, or aicd lead or generator proc (HCFA,115)

Topic 67

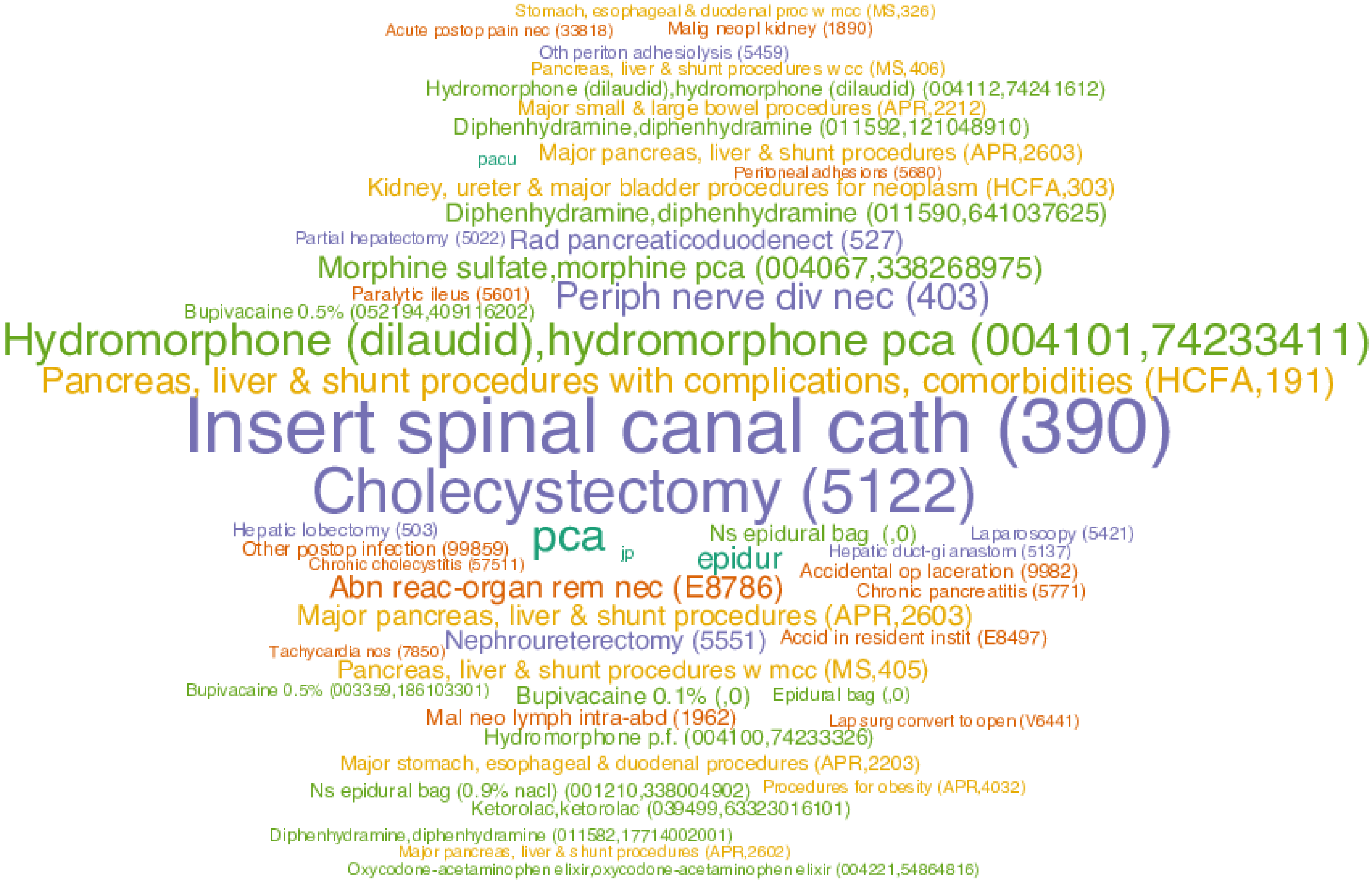
Stomach, esophageal & duodenal proc w mcc (MS,326)
Acute postop pain nec (33818)
Malig neopl kidney (1890)
Oth periton adhesiolysis (5459)
Pancreas, liver & shunt procedures w cc (MS,406)
Hydromorphone (dilaudid),hydromorphone (dilaudid) (004112,74241612)
Major small & large bowel procedures (APR,2212)
Diphenhydramine,diphenhydramine (011592,121048910)
pacu
Major pancreas, liver & shunt procedures (APR,2603)
Peritoneal adhesions (5680)
Kidney, ureter & major bladder procedures for neoplasm (HCFA,303)
Diphenhydramine,diphenhydramine (011590,641037625)
Partial hepatectomy (5022)
Rad pancreaticoduodenect (527)
Morphine sulfate,morphine pca (004067,338268975)
Paralytic ileus (5601)
Periph nerve div nec (403)
Bupivacaine 0.5% (052194,409116202)
Hydromorphone (dilaudid),hydromorphone pca (004101,74233411)
Pancreas, liver & shunt procedures with complications, comorbidities (HCFA,191)
Insert spinal canal cath (390)
Cholecystectomy (5122)
Hepatic lobectomy (503)
pca
Ns epidural bag (,0)
Laparoscopy (5421)
Other postop infection (99859)
jp
Hepatic duct-gi anastom (5137)
Chronic cholecystitis (57511)
epidur
Accidental op laceration (9982)
Abn reac-organ rem nec (E8786)
Chronic pancreatitis (5771)
Major pancreas, liver & shunt procedures (APR,2603)
Nephroureterectomy (5551)
Accid in resident instit (E8497)
Tachycardia nos (7850)
Pancreas, liver & shunt procedures w mcc (MS,405)
Bupivacaine 0.5% (003359,186103301)
Bupivacaine 0.1% (,0)
Epidural bag (,0)
Mal neo lymph intra-abd (1962)
Lap surg convert to open (V6441)
Hydromorphone p.f. (004100,74233326)
Major stomach, esophageal & duodenal procedures (APR,2203)
Ns epidural bag (0.9% nacl) (001210,338004902)
Procedures for obesity (APR,4032)
Ketorolac,ketorolac (039499,63323016101)
Diphenhydramine,diphenhydramine (011582,17714002001)
Major pancreas, liver & shunt procedures (APR,2602)
Oxycodone-acetaminophen elixir,oxycodone-acetaminophen elixir (004221,54864816)

Topic 68

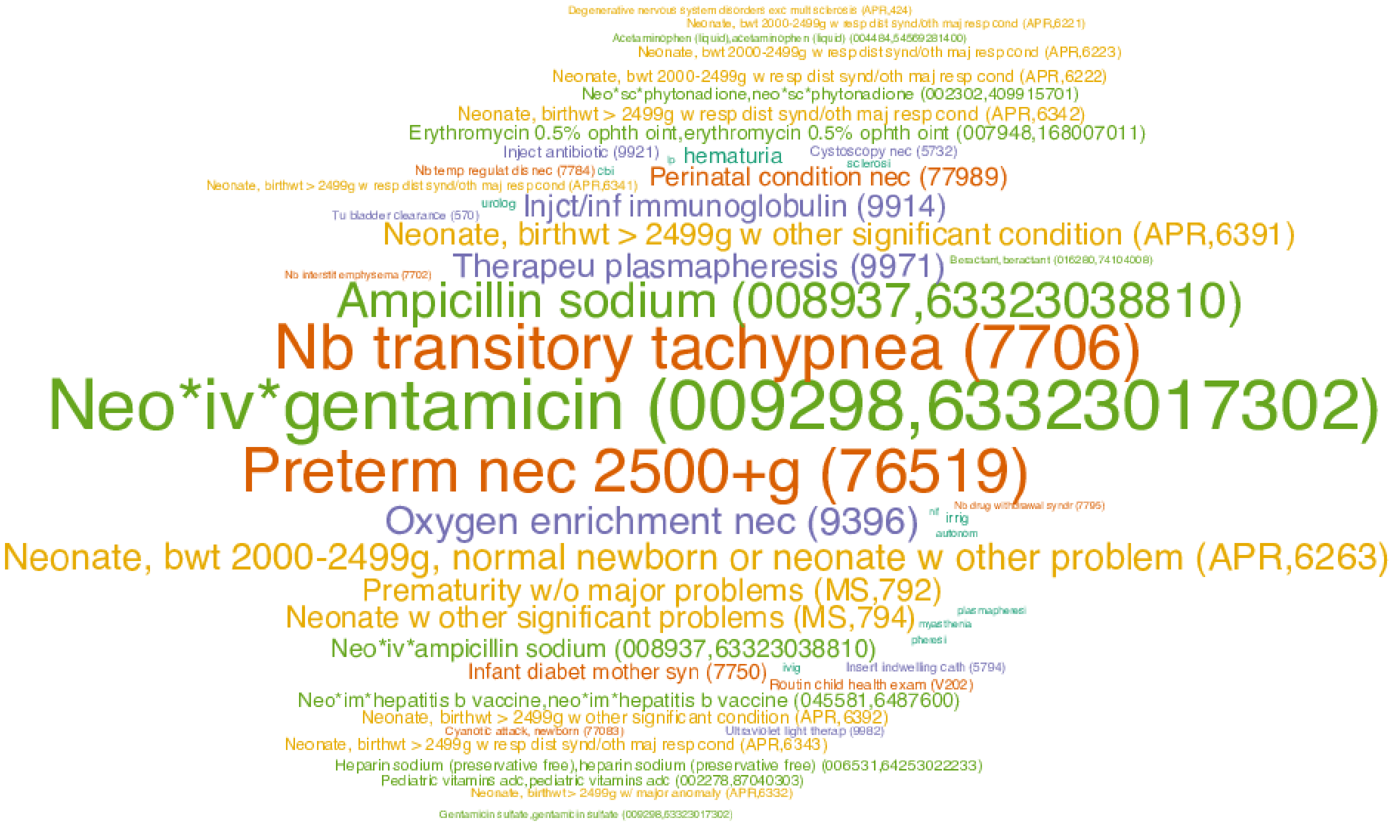

Degenerative nervous system disorders exc mult sclerosis (APR,424)
Neonate, bwt 2000-2499g w resp dist synd/oth maj resp cond (APR,6221)
Acetaminophen (liquid),acetaminophen (liquid) (004484,54569281400)
Neonate, bwt 2000-2499g w resp dist synd/oth maj resp cond (APR,6223)
Neonate, bwt 2000-2499g w resp dist synd/oth maj resp cond (APR,6222)
Neo*sc*phytonadione,neo*sc*phytonadione (002302,409915701)
Neonate, birthwt > 2499g w resp dist synd/oth maj resp cond (APR,6342)
Erythromycin 0.5% ophth oint,erythromycin 0.5% ophth oint (007948,168007011)
Inject antibiotic (9921)
hematuria
Cystoscopy nec (5732)
sclerosi
Nb temp regulat dis nec (7784)
cbi
Perinatal condition nec (77989)
Neonate, birthwt > 2499g w resp dist synd/oth maj resp cond (APR,6341)
urolog
Injct/inf immunoglobulin (9914)
Tu bladder clearance (570)
Neonate, birthwt > 2499g w other significant condition (APR,6391)
Therapeu plasmapheresis (9971)
Beractant,beractant (016280,74104008)
Nb interstit emphysema (7702)
Ampicillin sodium (008937,63323038810)
Nb transitory tachypnea (7706)
Neo*iv*gentamicin (009298,63323017302)
Preterm nec 2500+g (76519)
Oxygen enrichment nec (9396)
irrig
autonom
Neonate, bwt 2000-2499g, normal newborn or neonate w other problem (APR,6263)
Prematurity w/o major problems (MS,792)
Neonate w other significant problems (MS,794)
plasmapheresi
myasthenia
pheresi
Neo*iv*ampicillin sodium (008937,63323038810)
Infant diabet mother syn (7750)
ivig
Insert indwelling cath (5794)
Routin child health exam (V202)
Neo*im*hepatitis b vaccine,neo*im*hepatitis b vaccine (045581,6487600)
Neonate, birthwt > 2499g w other significant condition (APR,6392)
Cyanotic attack, newborn (77083)
Ultraviolet light therap (9982)
Neonate, birthwt > 2499g w resp dist synd/oth maj resp cond (APR,6343)
Heparin sodium (preservative free),heparin sodium (preservative free) (006531,64253022233)
Pediatric vitamins adc,pediatric vitamins adc (002278,87040303)
Neonate, birthwt > 2499g w/ major anomaly (APR,6332)
Gentamicin sulfate,gentamicin sulfate (009298,63323017302)

Topic 69

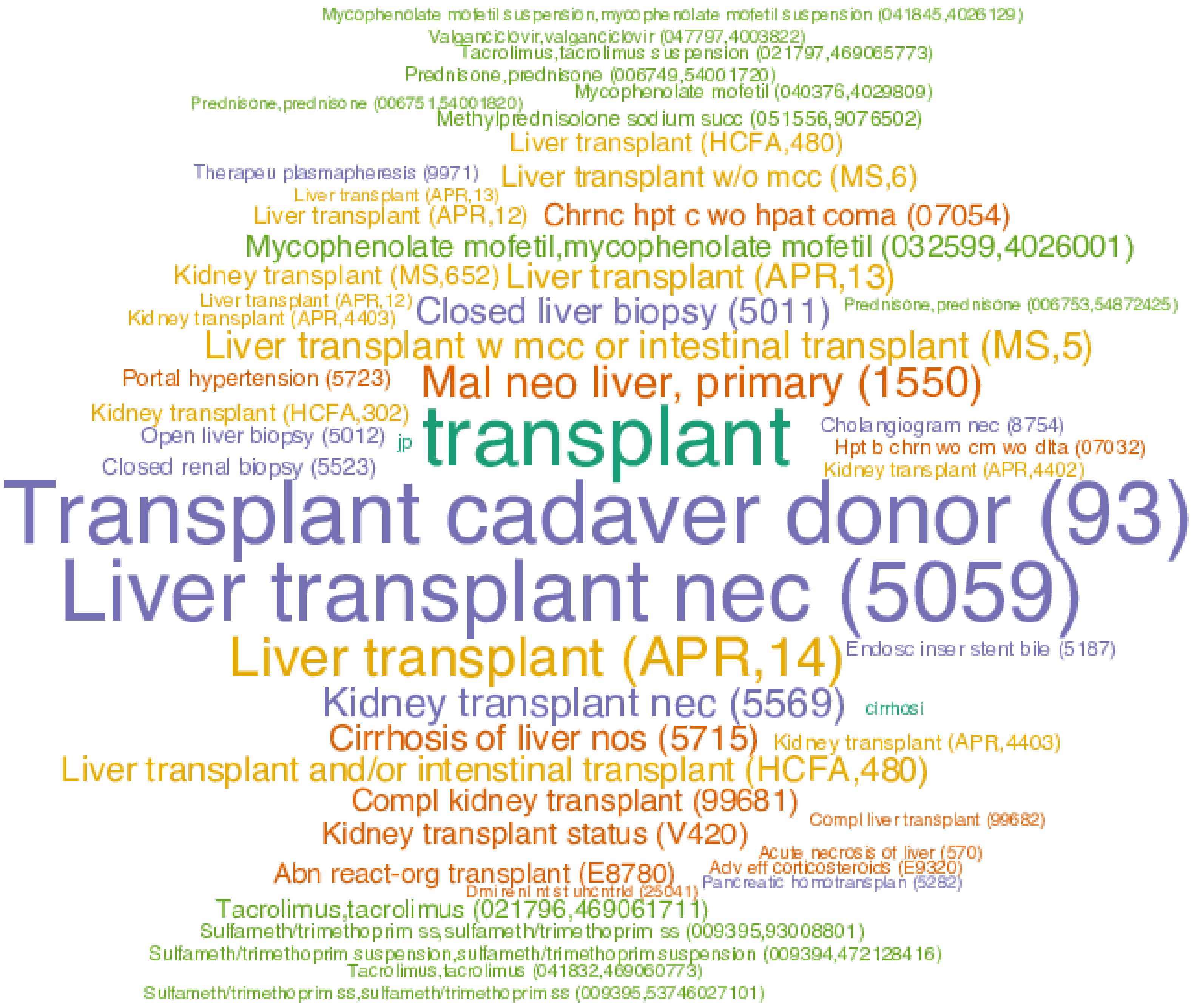

Mycophenolate mofetil suspension,mycophenolate mofetil suspension (041845,4026129)
Valganciclovir,valganciclovir (047797,4003822)
Tacrolimus,tacrolimus suspension (021797,469065773)
Prednisone,prednisone (006749,54001720)
Mycophenolate mofetil (040376,4029809)
Prednisone,prednisone (006751,54001820)
Methylprednisolone sodium succ (051556,9076502)
Liver transplant (HCFA,480)
Therapeu plasmapheresis (9971)
Liver transplant w/o mcc (MS,6)
Liver transplant (APR,13)
Liver transplant (APR,12)
Chrnc hpt c wo hpat coma (07054)
Mycophenolate mofetil,mycophenolate mofetil (032599,4026001)
Kidney transplant (MS,652)
Liver transplant (APR,13)
Liver transplant (APR,12)
Kidney transplant (APR,4403)
Closed liver biopsy (5011)
Prednisone,prednisone (006753,54872425)
Liver transplant w mcc or intestinal transplant (MS,5)
Portal hypertension (5723)
Mal neo liver, primary (1550)
Kidney transplant (HCFA,302)
Open liver biopsy (5012)
jp
transplant
Cholangiogram nec (8754)
Hpt b chrn wo cm wo dlta (07032)
Closed renal biopsy (5523)
Kidney transplant (APR,4402)
Transplant cadaver donor (93)
Liver transplant nec (5059)
Liver transplant (APR,14)
Endosc inser stent bile (5187)
Kidney transplant nec (5569)
cirrhosi
Cirrhosis of liver nos (5715)
Kidney transplant (APR,4403)
Liver transplant and/or intenstinal transplant (HCFA,480)
Compl kidney transplant (99681)
Compl liver transplant (99682)
Kidney transplant status (V420)
Acute necrosis of liver (570)
Abn react-org transplant (E8780)
Adv eff corticosteroids (E9320)
Pancreatic homotransplan (5282)
Dmi renl nt st uncntrld (25041)
Tacrolimus,tacrolimus (021796,469061711)
Sulfameth/trimethoprim ss,sulfameth/trimethoprim ss (009395,93008801)
Sulfameth/trimethoprim suspension,sulfameth/trimethoprim suspension (009394,47212841 6)
Tacrolimus,tacrolimus (041832,469060773)
Sulfameth/trimethoprim ss,sulfameth/trimethoprim ss (009395,53746027101)

Topic 70

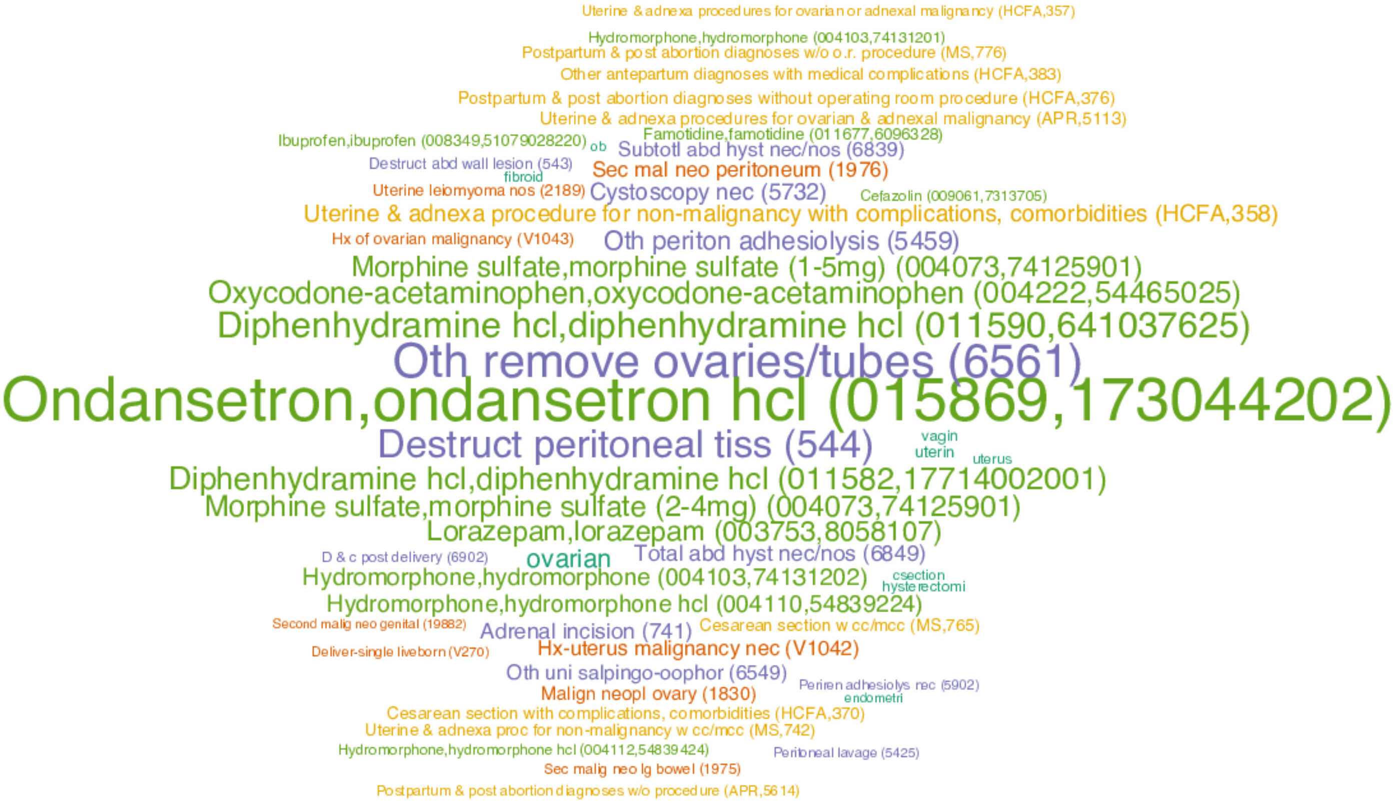
Uterine & adnexa procedures for ovarian or adnexal malignancy (HCFA,357)
Hydromorphone,hydromorphone (004103,74131201)
Postpartum & post abortion diagnoses w/o o.r. procedure (MS,776)
Other antepartum diagnoses with medical complications (HCFA,383)
Postpartum & post abortion diagnoses without operating room procedure (HCFA,376)
Uterine & adnexa procedures for ovarian & adnexal malignancy (APR,5113)
Famotidine,famotidine (011677,6096328)
Ibuprofen,ibuprofen (008349,51079028220)
ob
Subtotl abd hyst nec/nos (6839)
Destruct abd wall lesion (543)
Sec mal neo peritoneum (1976)
fibroid
Uterine leiomyoma nos (2189)
Cystoscopy nec (5732)
Cefazolin (009061,7313705)
Uterine & adnexa procedure for non-malignancy with complications, comorbidities (HCFA,358)
Hx of ovarian malignancy (V1043)
Oth periton adhesiolysis (5459)
Morphine sulfate,morphine sulfate (1-5mg) (004073,74125901)
Oxycodone-acetaminophen,oxycodone-acetaminophen (004222,54465025)
Diphenhydramine hcl,diphenhydramine hcl (011590,641037625)
Oth remove ovaries/tubes (6561)
Ondansetron,ondansetron hcl (015869,173044202)
vagin
Destruct peritoneal tiss (544)
uterin
uterus
Diphenhydramine hcl,diphenhydramine hcl (011582,17714002001)
Morphine sulfate,morphine sulfate (2-4mg) (004073,74125901)
Lorazepam,lorazepam (003753,8058107)
D & c post delivery (6902)
ovarian
Total abd hyst nec/nos (6849)
Hydromorphone,hydromorphone (004103,74131202)
csection
hysterectomi
Hydromorphone,hydromorphone hcl (004110,54839224)
Second malig neo genital (19882)
Adrenal incision (741)
Cesarean section w cc/mcc (MS,765)
Deliver-single liveborn (V270)
Hx-uterus malignancy nec (V1042)
Oth uni salpingo-oophor (6549)
Periren adhesiolys nec (5902)
Malign neopl ovary (1830)
endometri
Cesarean section with complications, comorbidities (HCFA,370)
Uterine & adnexa proc for non-malignancy w cc/mcc (MS,742)
Hydromorphone,hydromorphone hcl (004112,54839424)
Peritoneal lavage (5425)
Sec malig neo lg bowel (1975)
Postpartum & post abortion diagnoses w/o procedure (APR,5614)

Topic 71

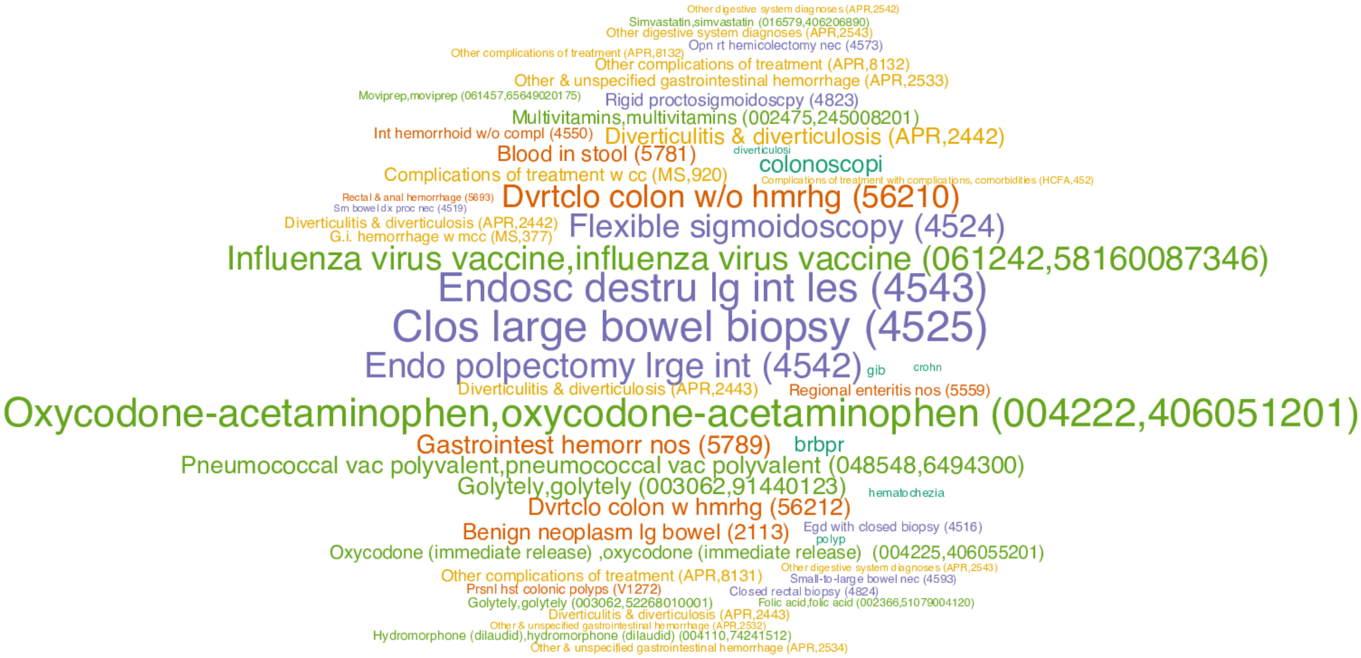

Other digestive system diagnoses (APR,2542)
Simvastatin,simvastatin (016579,406206890)
Other digestive system diagnoses (APR,2543)
Opn rt hemicolectomy nec (4573)
Other complications of treatment (APR,8132)
Other complications of treatment (APR,8132)
Other & unspecified gastrointestinal hemorrhage (APR,2533)
Moviprep,moviprep (061457,65649020175)
Rigid proctosigmoidoscpy (4823)
Multivitamins,multivitamins (002475,245008201)
Int hemorrhoid w/o compl (4550)
Diverticulitis & diverticulosis (APR,2442)
Blood in stool (5781)
diverticulosi
colonoscopi
Complications of treatment w cc (MS,920)
Complications of treatment with complications, comorbidities (HCFA,452)
Rectal & anal hemorrhage (5693)
Sm bowel dx proc nec (4519)
Dvrtclo colon w/o hmrhg (56210)
Diverticulitis & diverticulosis (APR,2442)
G.i. hemorrhage w mcc (MS,377)
Flexible sigmoidoscopy (4524)
Influenza virus vaccine,influenza virus vaccine (061242,58160087346)
Endosc destru lg int les (4543)
Clos large bowel biopsy (4525)
Endo polpectomy lrge int (4542)
gib
crohn
Diverticulitis & diverticulosis (APR,2443)
Regional enteritis nos (5559)
Oxycodone-acetaminophen,oxycodone-acetaminophen (004222,406051201)
Gastrointest hemorr nos (5789)
brbpr
Pneumococcal vac polyvalent,pneumococcal vac polyvalent (048548,6494300)
Golytely,golytely (003062,91440123)
hematochezia
Dvrtclo colon w hmrhg (56212)
Benign neoplasm lg bowel (2113)
Egd with closed biopsy (4516)
polyp
Oxycodone (immediate release) ,oxycodone (immediate release) (004225,406055201)
Other digestive system diagnoses (APR,2543)
Other complications of treatment (APR,8131)
Small-to-large bowel nec (4593)
Prsnl hst colonic polyps (V1272)
Closed rectal biopsy (4824)
Golytely,golytely (003062,52268010001)
Folic acid,folic acid (002366,51079004120)
Diverticulitis & diverticulosis (APR,2443)
Other & unspecified gastrointestinal hemorrhage (APR,2532)
Hydromorphone (dilaudid),hydromorphone (dilaudid) (004110,74241512)
Other & unspecified gastrointestinal hemorrhage (APR,2534)

Topic 72

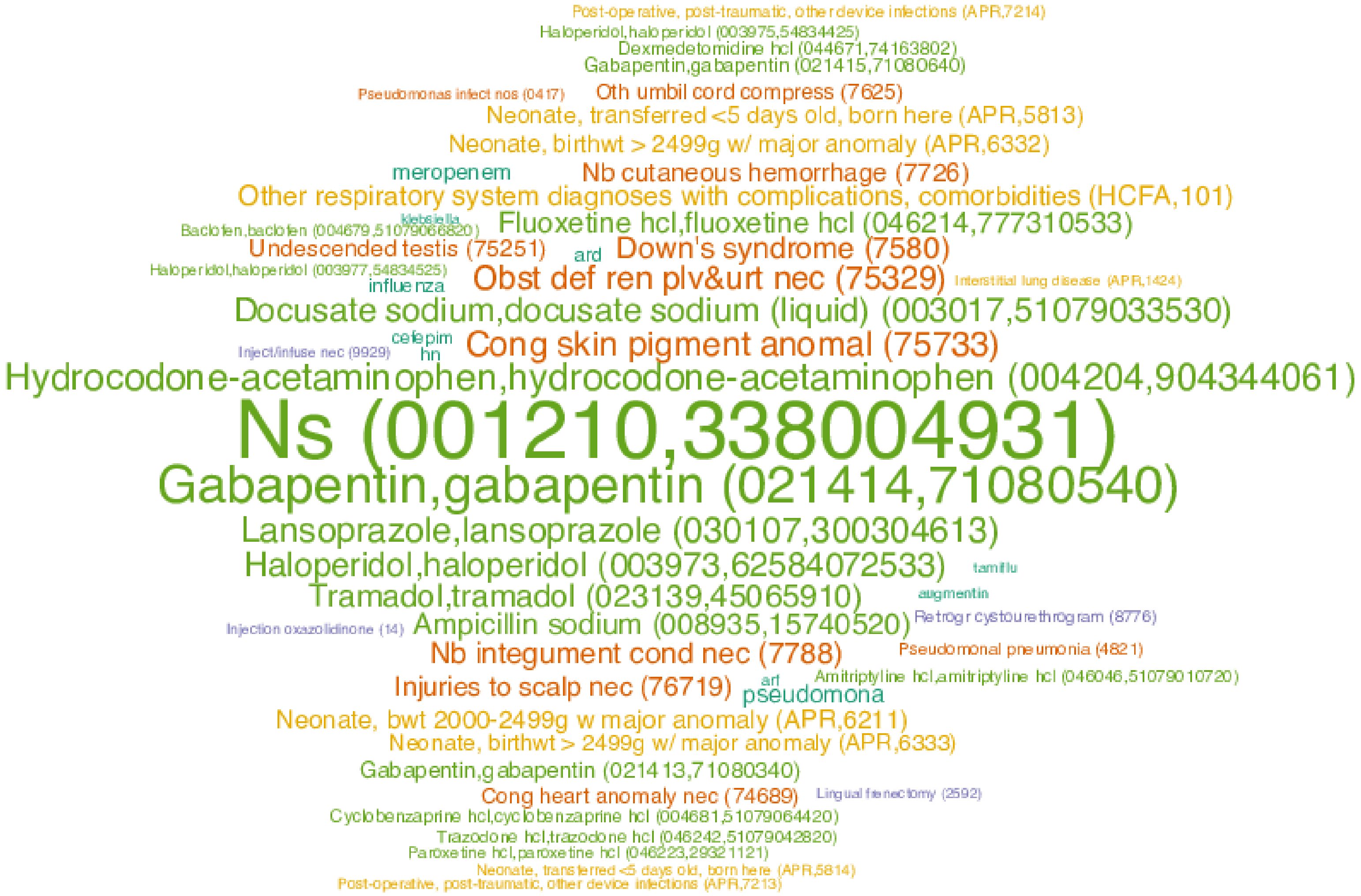
Post-operative, post-traumatic, other device infections (APR,7214)
Haloperidol,haloperidol (003975,54834425)
Dexmedetomidine hcl (044671,74163802)
Gabapentin,gabapentin (021415,71080640)
Pseudomonas infect nos (0417)
Oth umbil cord compress (7625)
Neonate, transferred <5 days old, born here (APR,5813)
Neonate, birthwt > 2499g w/ major anomaly (APR,6332)
meropenem
Nb cutaneous hemorrhage (7726)
Other respiratory system diagnoses with complications, comorbidities (HCFA,101)
klebsiella
Baclofen,baclofen (004679,51079066820)
Fluoxetine hcl,fluoxetine hcl (046214,777310533)
Undescended testis (75251)
ard
Down's syndrome (7580)
Haloperidol,haloperidol (003977,54834525)
influenza
Obst def ren plv&urt nec (75329)
Interstitial lung disease (APR,1424)
Docusate sodium,docusate sodium (liquid) (003017,51079033530)
cefepim
Inject/infuse nec (9929)
hn
Cong skin pigment anomal (75733)
Hydrocodone-acetaminophen,hydrocodone-acetaminophen (004204,904344061)
Ns (001210,338004931)
Gabapentin,gabapentin (021414,71080540)
Lansoprazole,lansoprazole (030107,300304613)
Haloperidol,haloperidol (003973,62584072533)
tamiflu
Tramadol,tramadol (023139,45065910)
augmentin
Injection oxazolidinone (14)
Ampicillin sodium (008935,15740520)
Retrogr cystourethrogram (8776)
Nb integument cond nec (7788)
Pseudomonal pneumonia (4821)
art
Amitriptyline hcl,amitriptyline hcl (046046,51079010720)
Injuries to scalp nec (76719)
pseudomona
Neonate, bwt 2000-2499g w major anomaly (APR,6211)
Neonate, birthwt > 2499g w/ major anomaly (APR,6333)
Gabapentin,gabapentin (021413,71080340)
Cong heart anomaly nec (74689)
Lingual frenectomy (2592)
Cyclobenzaprine hcl,cyclobenzaprine hcl (004681,51079064420)
Trazodone hcl,trazodone hcl (046242,51079042820)
Paroxetine hcl,paroxetine hcl (046223,29321121)
Neonate, transferred <5 days old, born here (APR,5814)
Post-operative, post-traumatic, other device infections (APR,7213)

Topic 73

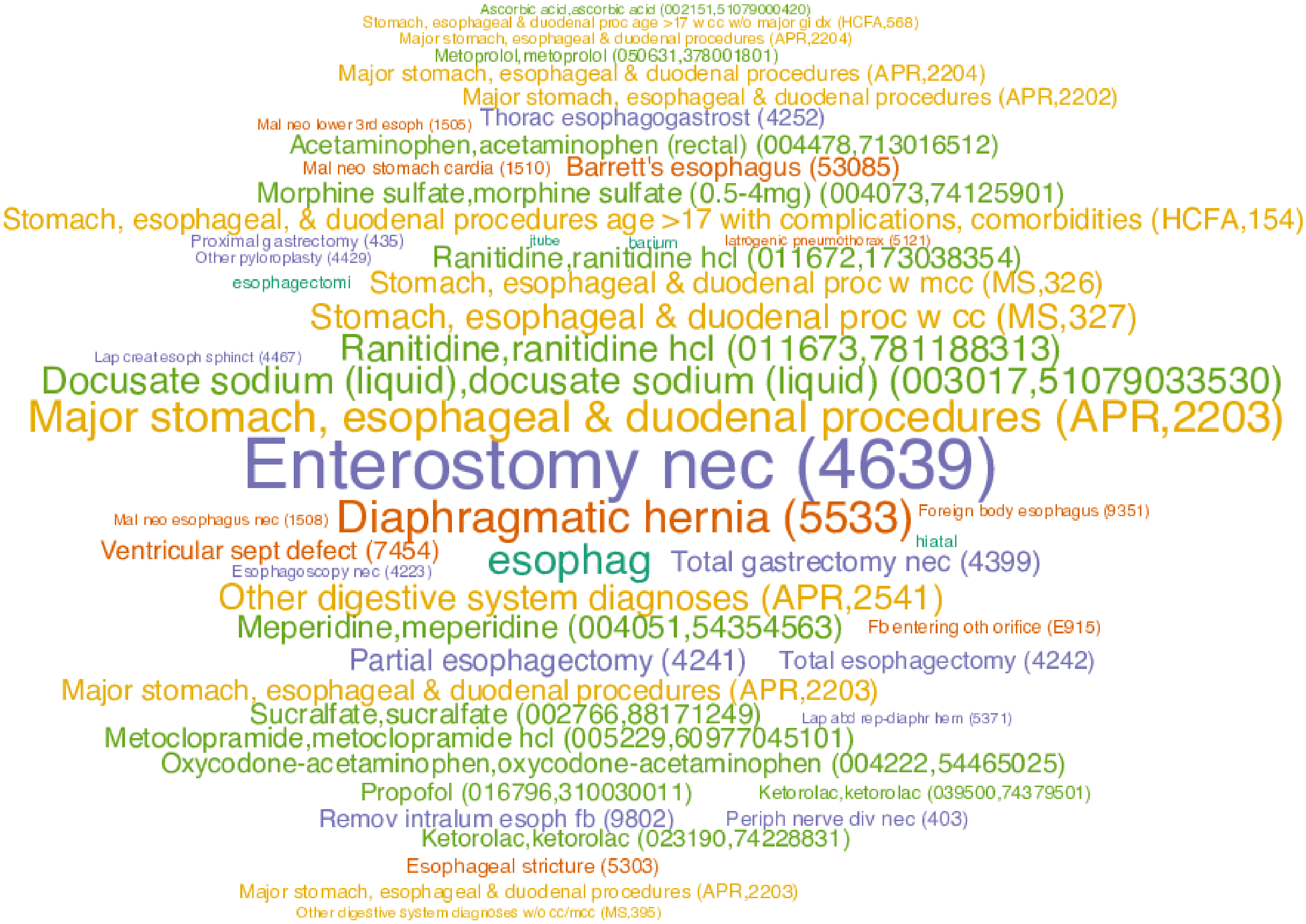

Ascorbic acid,ascorbic acid (002151,51079000420)
Stomach, esophageal & duodenal proc age >17 w cc w/o major gi dx (HCFA,568)
Major stomach, esophageal & duodenal procedures (APR,2204)
Metoprolol,metoprolol (050631,378001801)
Major stomach, esophageal & duodenal procedures (APR,2204)
Major stomach, esophageal & duodenal procedures (APR,2202)
Mal neo lower 3rd esoph (1505)
Thorac esophagogastrost (4252)
Acetaminophen,acetaminophen (rectal) (004478,713016512)
Mal neo stomach cardia (1510)
Barrett's esophagus (53085)
Morphine sulfate,morphine sulfate (0.5-4mg) (004073,74125901)
Stomach, esophageal, & duodenal procedures age >17 with complications, comorbidities (HCFA,154)
Proximal gastrectomy (435)
jtube
barium
Iatrogenic pneumothorax (5121)
Other pyloroplasty (4429)
Ranitidine,ranitidine hcl (011672,173038354)
esophagectomi
Stomach, esophageal & duodenal proc w mcc (MS,326)
Stomach, esophageal & duodenal proc w cc (MS,327)
Lap creat esoph sphinct (4467)
Ranitidine,ranitidine hcl (011673,781188313)
Docusate sodium (liquid),docusate sodium (liquid) (003017,51079033530)
Major stomach, esophageal & duodenal procedures (APR,2203)
Enterostomy nec (4639)
Mal neo esophagus nec (1508)
Diaphragmatic hernia (5533)
Foreign body esophagus (9351)
hiatal
Ventricular sept defect (7454)
esophag
Total gastrectomy nec (4399)
Esophagoscopy nec (4223)
Other digestive system diagnoses (APR,2541)
Meperidine,meperidine (004051,54354563)
Fb entering oth orifice (E915)
Partial esophagectomy (4241)
Total esophagectomy (4242)
Major stomach, esophageal & duodenal procedures (APR,2203)
Sucralfate,sucralfate (002766,88171249)
Lap abd rep-diaphr hern (5371)
Metoclopramide,metoclopramide hcl (005229,60977045101)
Oxycodone-acetaminophen,oxycodone-acetaminophen (004222,54465025)
Propofol (016796,310030011)
Ketorolac,ketorolac (039500,74379501)
Remov intralum esoph fb (9802)
Periph nerve div nec (403)
Ketorolac,ketorolac (023190,74228831)
Esophageal stricture (5303)
Major stomach, esophageal & duodenal procedures (APR,2203)
Other digestive system diagnoses w/o cc/mcc (MS,395)

Topic 74

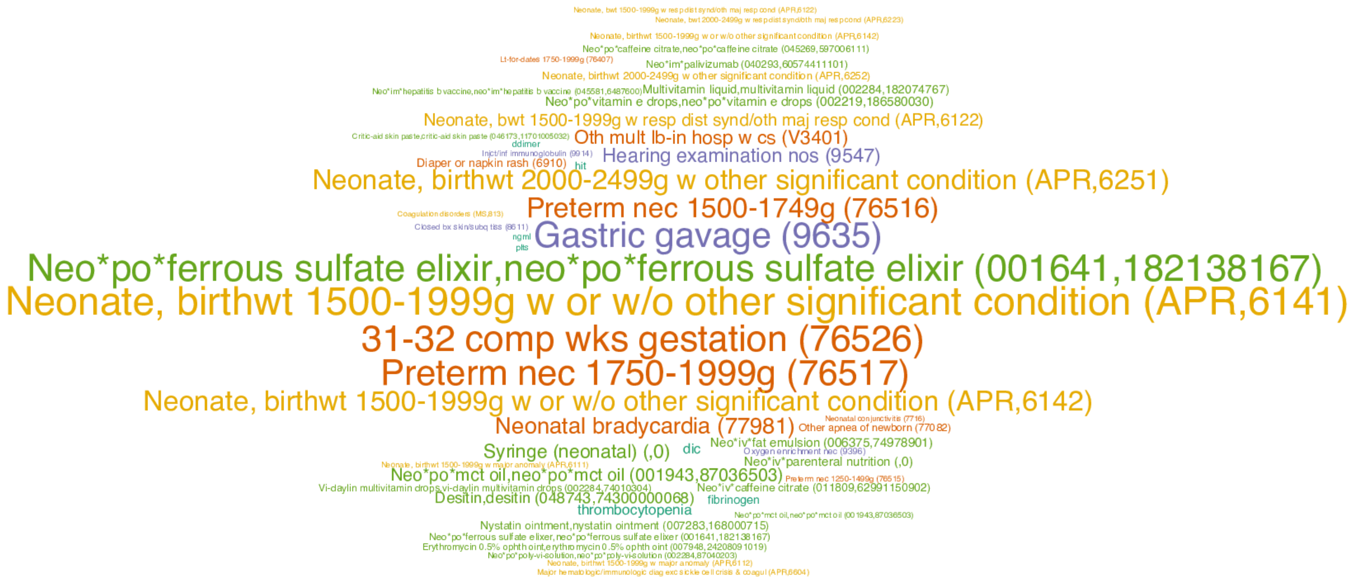
Neonate, bwt 1500-1999g w resp dist synd/oth maj resp cond (APR,6122)
Neonate, bwt 2000-2499g w resp dist synd/oth maj resp cond (APR,6223)
Neonate, birthwt 1500-1999g w or w/o other significant condition (APR,6142)
Neo*po*caffeine citrate,neo*po*caffeine citrate (045269,597006111)
Lt-for-dates 1750-1999g (76407)
Neo*im*palivizumab (040293,60574411101)
Neonate, birthwt 2000-2499g w other significant condition (APR,6252)
Neo*im*hepatitis b vaccine,neo*im*hepatitis b vaccine (045581,6487600)
Multivitamin liquid,multivitamin liquid (002284,182074767)
Neo*po*vitamin e drops,neo*po*vitamin e drops (002219,186580030)
Neonate, bwt 1500-1999g w resp dist synd/oth maj resp cond (APR,6122)
Critic-aid skin paste,critic-aid skin paste (046173,11701005032)
Oth mult lb-in hosp w cs (V3401)
ddimer
Injct/inf immunoglobulin (9914)
Hearing examination nos (9547)
Diaper or napkin rash (6910)
hit
Neonate, birthwt 2000-2499g w other significant condition (APR,6251)
Coagulation disorders (MS,813)
Preterm nec 1500-1749g (76516)
Closed bx skin/subq tiss (8611)
ngml
Gastric gavage (9635)
plts
Neo*po*ferrous sulfate elixir,neo*po*ferrous sulfate elixir (001641,182138167)
Neonate, birthwt 1500-1999g w or w/o other significant condition (APR,6141)
31-32 comp wks gestation (76526)
Preterm nec 1750-1999g (76517)
Neonate, birthwt 1500-1999g w or w/o other significant condition (APR,6142)
Neonatal conjunctivitis (7716)
Neonatal bradycardia (77981)
Other apnea of newborn (77082)
Syringe (neonatal) (,0)
dic
Neo*iv*fat emulsion (006375,74978901)
Oxygen enrichment nec (9396)
Neo*iv*parenteral nutrition (,0)
Neonate, birthwt 1500-1999g w major anomaly (APR,6111)
Neo*po*mct oil,neo*po*mct oil (001943,87036503)
Preterm nec 1250-1499g (76515)
Vi-daylin multivitamin drops,vi-daylin multivitamin drops (002284,74010304)
Neo*iv*caffeine citrate (011809,62991150902)
Desitin,desitin (048743,74300000068)
fibrinogen
thrombocytopenia
Neo*po*mct oil,neo*po*mct oil (001943,87036503)
Nystatin ointment,nystatin ointment (007283,168000715)
Neo*po*ferrous sulfate elixer,neo*po*ferrous sulfate elixer (001641,182138167)
Erythromycin 0.5% ophth oint,erythromycin 0.5% ophth oint (007948,24208091019)
Neo*po*poly-vi-solution,neo*po*poly-vi-solution (002284,87040203)
Neonate, birthwt 1500-1999g w major anomaly (APR,6112)
Major hematologic/immunologic diag exc sickle cell crisis & coagul (APR,6604)

Topic 75

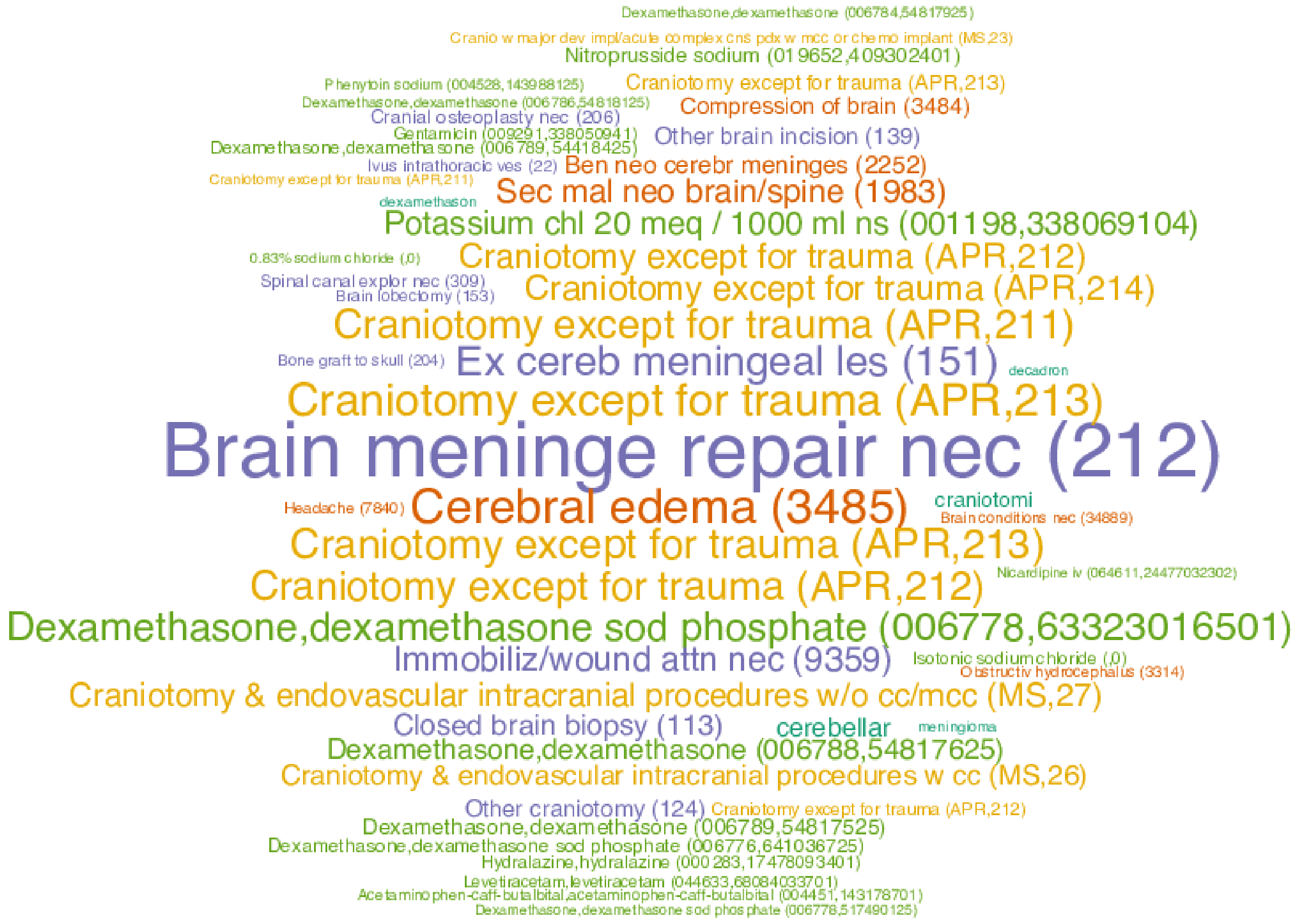
Dexamethasone,dexamethasone (006784,54817925)
Cranio w major dev impl/acute complex cns pdx w mcc or chemo implant (MS,23)
Nitroprusside sodium (019652,409302401)
Phenytoin sodium (004528,143988125)
Craniotomy except for trauma (APR,213)
Dexamethasone,dexamethasone (006786,54818125)
Compression of brain (3484)
Cranial osteoplasty nec (206)
Gentamicin (009291,338050941)
Other brain incision (139)
Dexamethasone,dexamethasone (006789,54418425)
Ivus intrathoracic ves (22)
Ben neo cerebr meninges (2252)
Craniotomy except for trauma (APR,211)
Sec mal neo brain/spine (1983)
dexamethason
Potassium chl 20 meq / 1000 ml ns (001198,338069104)
0.83% sodium chloride (,0)
Craniotomy except for trauma (APR,212)
Spinal canal explor nec (309)
Brain lobectomy (153)
Craniotomy except for trauma (APR,214)
Craniotomy except for trauma (APR,211)
Bone graft to skull (204)
Ex cereb meningeal les (151)
decadron
Craniotomy except for trauma (APR,213)
Brain meninge repair nec (212)
Headache (7840)
Cerebral edema (3485)
craniotomi
Brain conditions nec (34889)
Craniotomy except for trauma (APR,213)
Nicardipine iv (064611,24477032302)
Craniotomy except for trauma (APR,212)
Dexamethasone,dexamethasone sod phosphate (006778,63323016501)
Immobiliz/wound attn nec (9359)
Isotonic sodium chloride (,0)
Obstructiv hydrocephalus (3314)
Craniotomy & endovascular intracranial procedures w/o cc/mcc (MS,27)
Closed brain biopsy (113)
cerebellar
meningioma
Dexamethasone,dexamethasone (006788,54817625)
Craniotomy & endovascular intracranial procedures w cc (MS,26)
Other craniotomy (124)
Craniotomy except for trauma (APR,212)
Dexamethasone,dexamethasone (006789,54817525)
Dexamethasone,dexamethasone sod phosphate (006776,641036725)
Hydralazine,hydralazine (000283,17478093401)
Levetiracetam,levetiracetam (044633,68084033701)
Acetaminophen-caff-butalbital,acetaminophen-caff-butalbital (004451,143178701)
Dexamethasone,dexamethasone sod phosphate (006778,517490125)

Topic 1

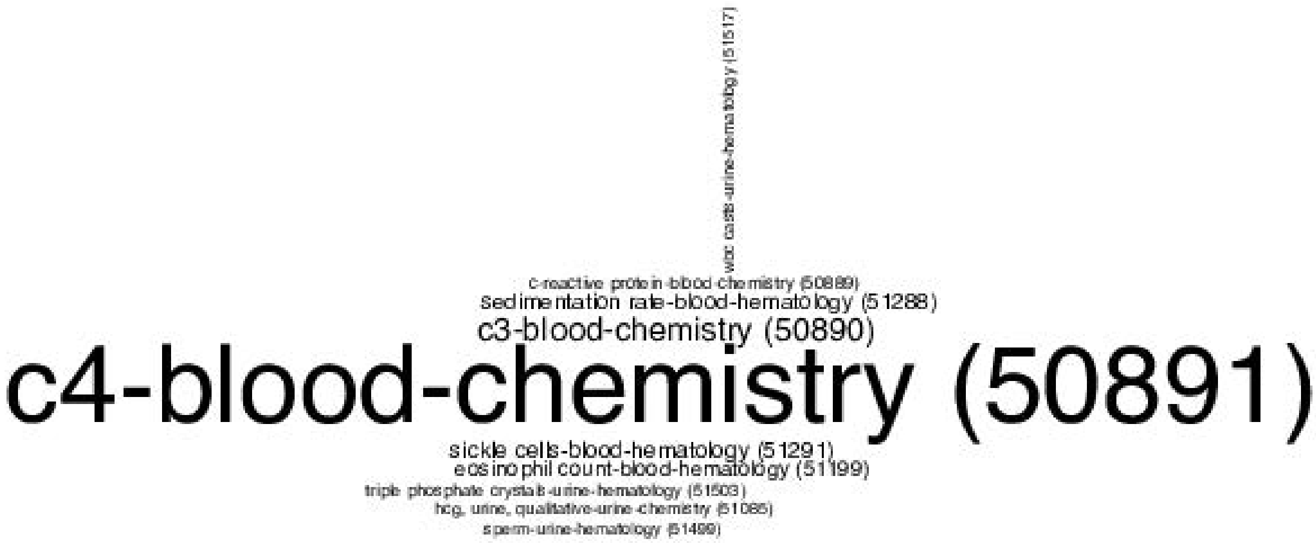
wbc casts-urine-hematology (51517)
c-reactive protein-blood-chemistry (50889)
sedimentation rate-blood-hematology (51288)
c3-blood-chemistry (50890)
c4-blood-chemistry (50891)
sickle cells-blood-hematology (51291)
eosinophil count-blood-hematology (51199)
triple phosphate crystals-urine-hematology (51503)
hcg, urine, qualitative-urine-chemistry (51085)
sperm-urine-hematology (51499)

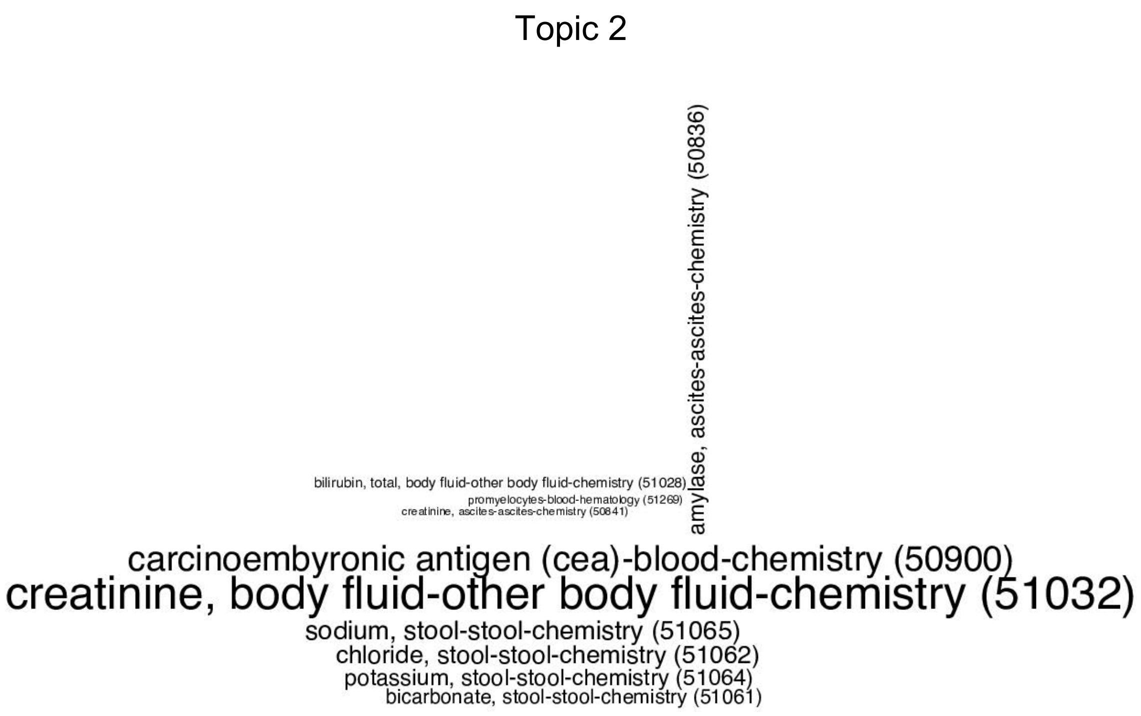
Topic 2
amylase, ascites-ascites-chemistry (50836)
bilirubin, total, body fluid-other body fluid-chemistry (51028)
promyelocytes-blood-hematology (51269)
creatinine, ascites-ascites-chemistry (50841)
carcinoembyronic antigen (cea)-blood-chemistry (50900)
creatinine, body fluid-other body fluid-chemistry (51032)
sodium, stool-stool-chemistry (51065)
chloride, stool-stool-chemistry (51062)
potassium, stool-stool-chemistry (51064)
bicarbonate, stool-stool-chemistry (51061)

Topic 3

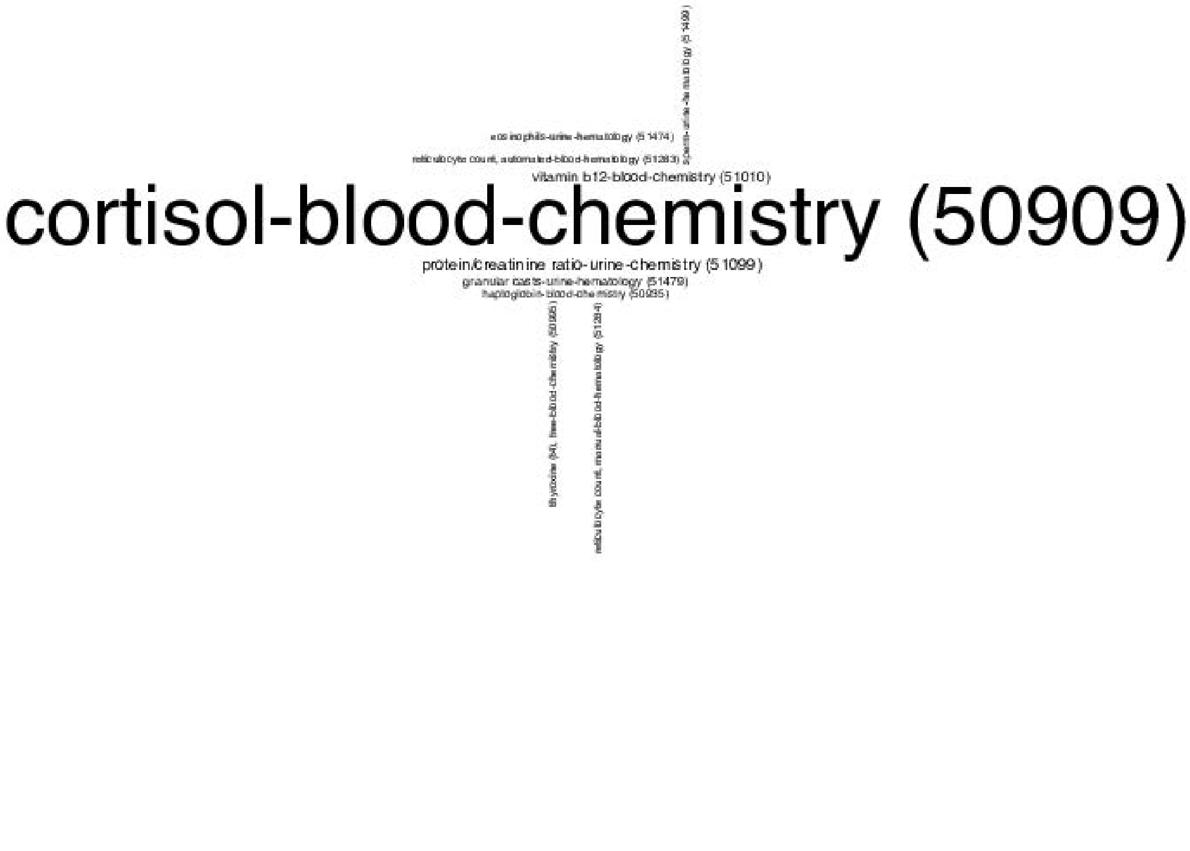
eosinophils-urine-hematology (51474)
reticulocyte count, automated-blood-hematology (51283)
vitamin b12-blood-chemistry (51010)
cortisol-blood-chemistry (50909)
protein/creatinine ratio-urine-chemistry (51099)
granular casts-urine-hematology (51479)
haptoglobin-blood-chemistry (50935)

Topic 4

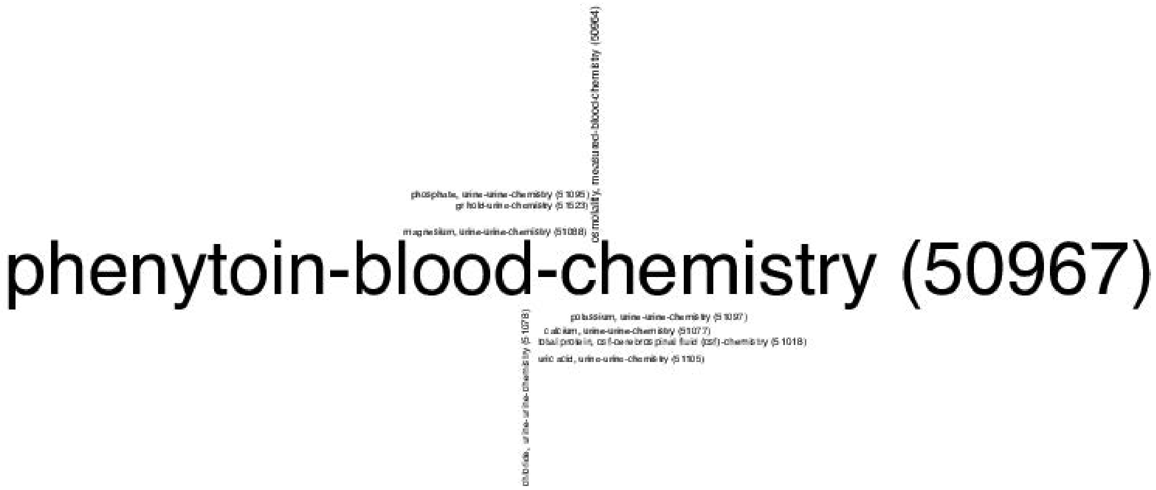

osmolality, measured-blood-chemistry (50964)
phosphate, urine-urine-chemistry (51095)
gr hold-urine-chemistry (51523)
magnesium, urine-urine-chemistry (51088)
phenytoin-blood-chemistry (50967)
potassium, urine-urine-chemistry (51097)
calcium, urine-urine-chemistry (51077)
total protein, csf-cerebrospinal fluid (csf)-chemistry (51018)
uric acid, urine-urine-chemistry (51105)
chloride, urine-urine-chemistry (51078)

Topic 5

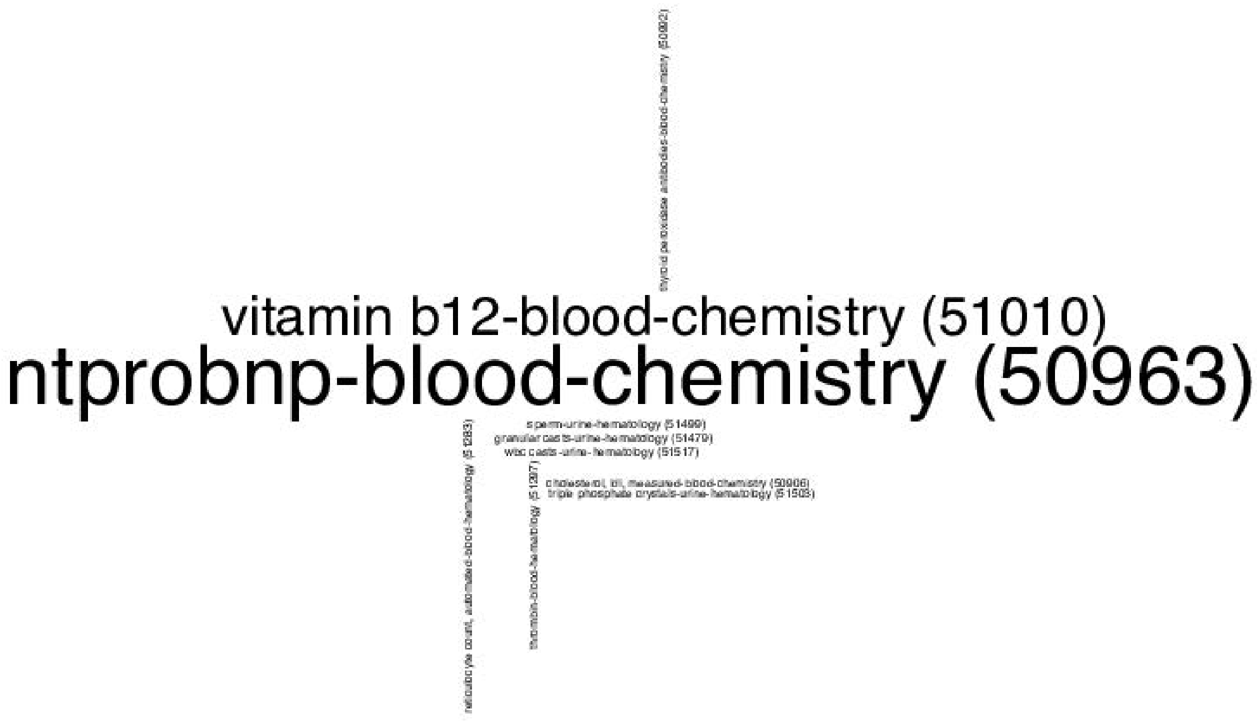
vitamin b12-blood-chemistry (51010)
ntprobnp-blood-chemistry (50963)
sperm-urine-hematology (51499)
granular casts-urine-hematology (51479)
wbc casts-urine-hematology (51517)
cholesterol, ldl, measured-blood-chemistry (50906)
triple phosphate crystals-urine-hematology (51503)

Topic 6

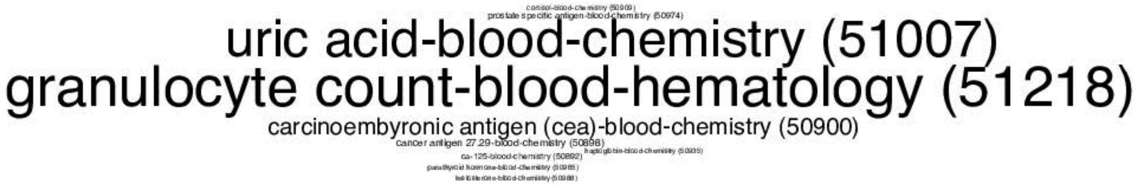
cortisol-blood-chemistry (50909)
prostate specific antigen-blood-chemistry (50974)
uric acid-blood-chemistry (51007)
granulocyte count-blood-hematology (51218)
carcinoembyronic antigen (cea)-blood-chemistry (50900)
cancer antigen 27.29-blood-chemistry (50898)
haptoglobin-blood-chemistry (50935)
ca-125-blood-chemistry (50892)

Topic 7

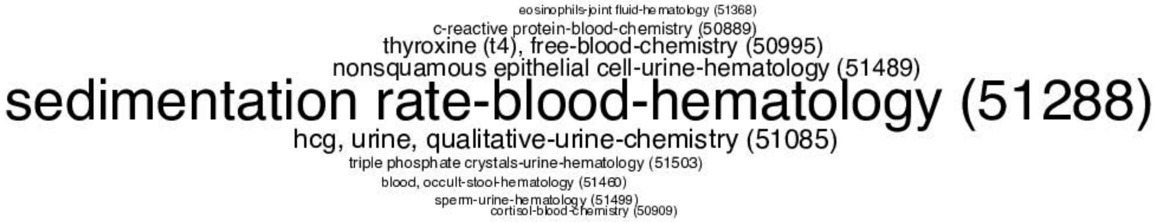
eosinophils-joint fluid-hematology (51368)
c-reactive protein-blood-chemistry (50889)
thyroxine (t4), free-blood-chemistry (50995)
nonsquamous epithelial cell-urine-hematology (51489)
sedimentation rate-blood-hematology (51288)
hcg, urine, qualitative-urine-chemistry (51085)
triple phosphate crystals-urine-hematology (51503)
blood, occult-stool-hematology (51460)
sperm-urine-hematology (51499)
cortisol-blood-chemistry (50909)

Topic 8

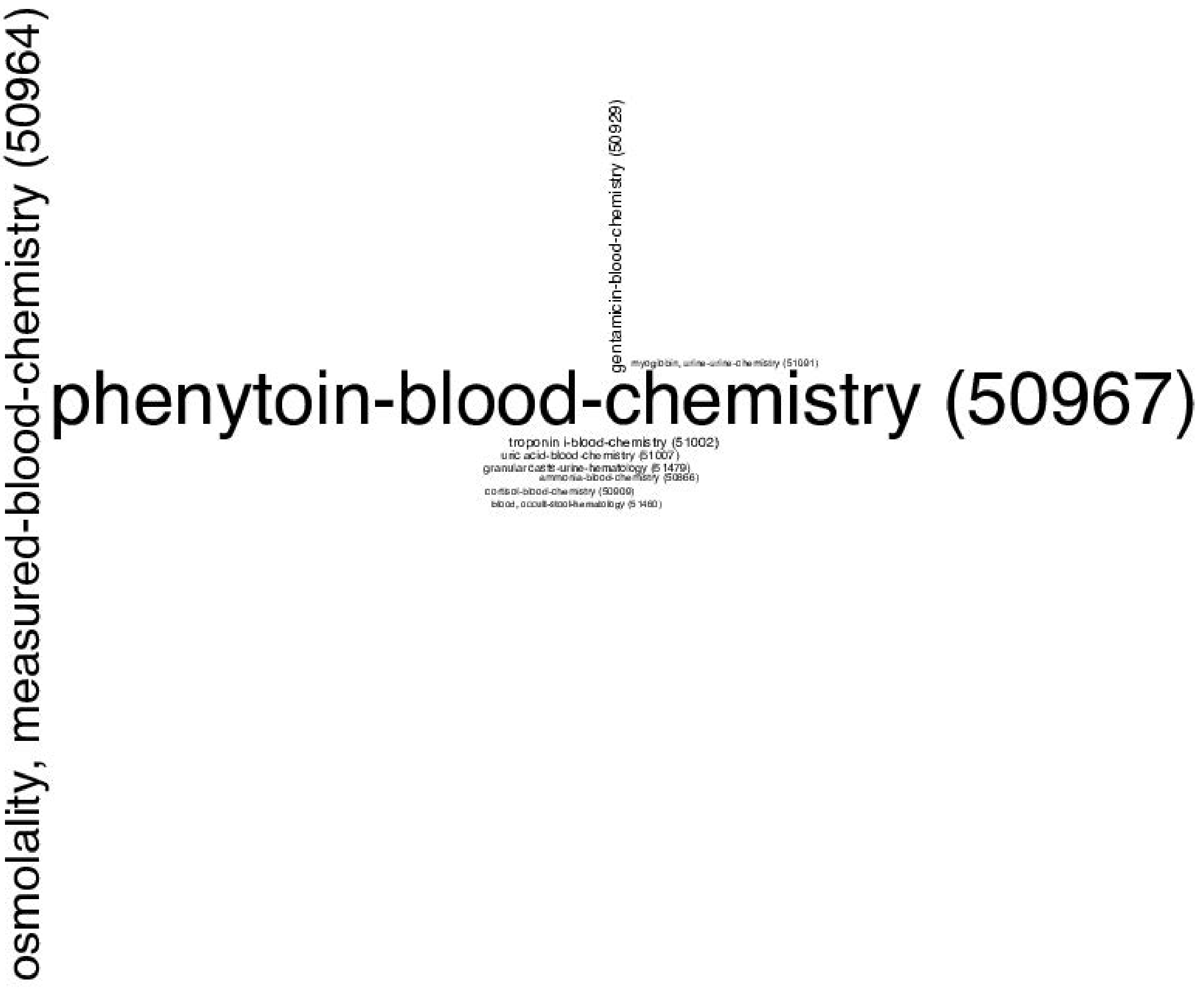
phenytoin-blood-chemistry (50967)
osmolality, measured-blood-chemistry (50964)
gentamicin-blood-chemistry (50929)
troponin i-blood-chemistry (51002)
uric acid-blood-chemistry (51007)

Topic 9

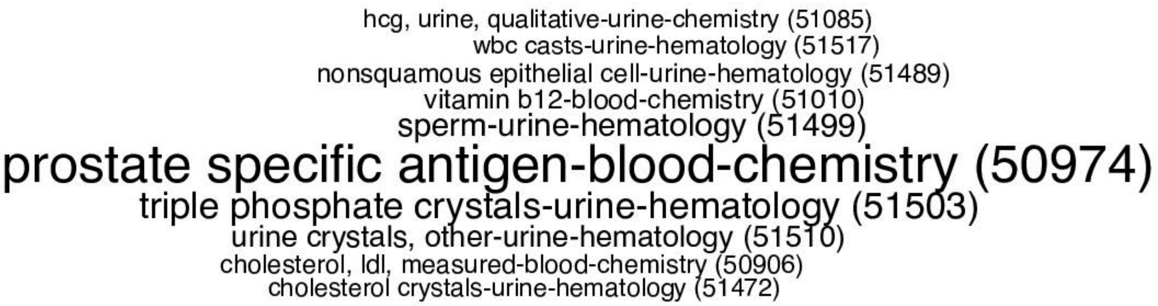
hcg, urine, qualitative-urine-chemistry (51085)
wbc casts-urine-hematology (51517)
nonsquamous epithelial cell-urine-hematology (51489)
vitamin b12-blood-chemistry (51010)
sperm-urine-hematology (51499)
prostate specific antigen-blood-chemistry (50974)
triple phosphate crystals-urine-hematology (51503)
urine crystals, other-urine-hematology (51510)
cholesterol, ldl, measured-blood-chemistry (50906)
cholesterol crystals-urine-hematology (51472)

Topic 10

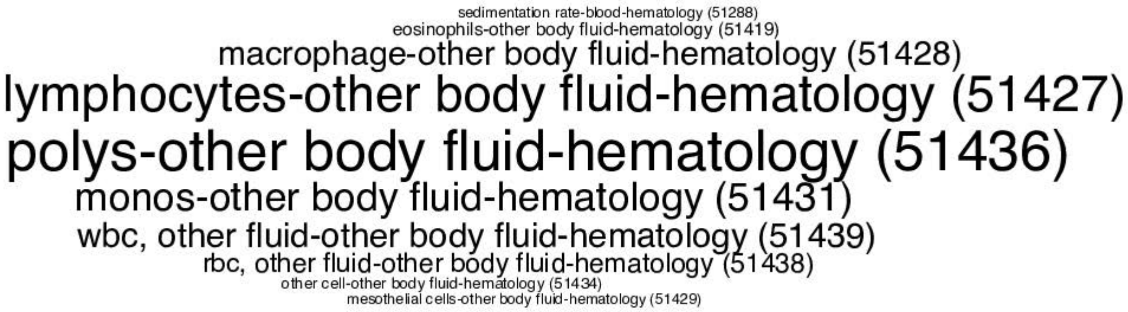
sedimentation rate-blood-hematology (51288)
eosinophils-other body fluid-hematology (51419)
macrophage-other body fluid-hematology (51428)
lymphocytes-other body fluid-hematology (51427)
polys-other body fluid-hematology (51436)
monos-other body fluid-hematology (51431)
wbc, other fluid-other body fluid-hematology (51439)
rbc, other fluid-other body fluid-hematology (51438)
other cell-other body fluid-hematology (51434)
mesothelial cells-other body fluid-hematology (51429)

Topic 11

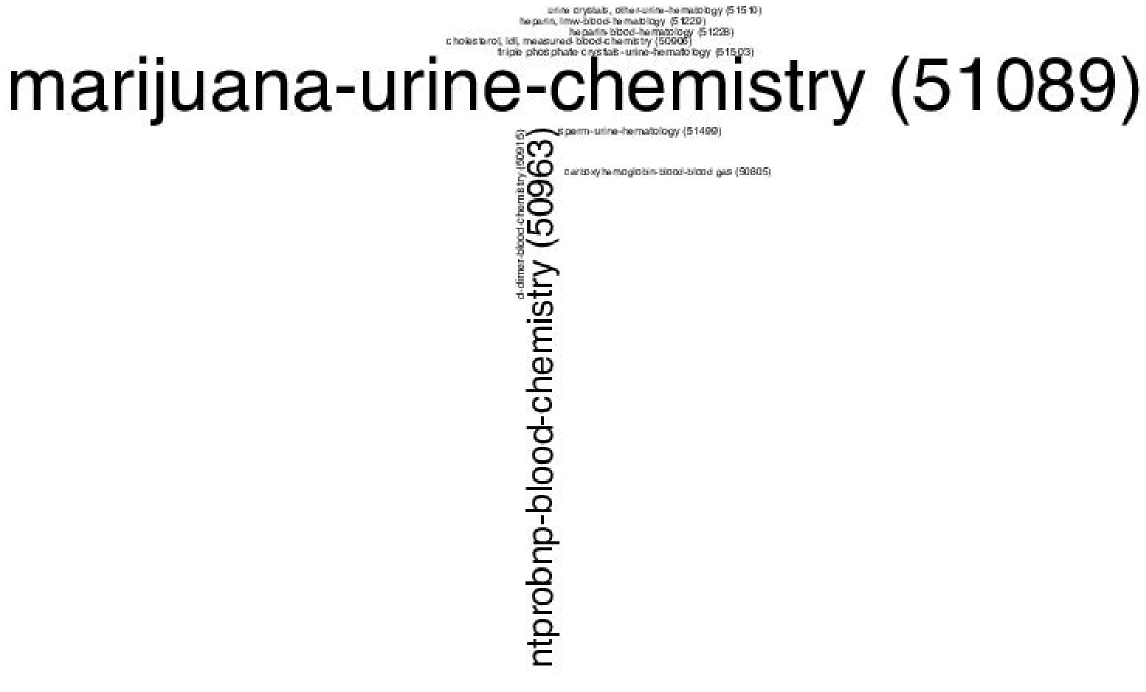
urine crystals, other-urine-hematology (51510)
heparin, lmw-blood-hematology (51229)
heparin-blood-hematology (51228)
cholesterol, ldl, measured-blood-chemistry (50906)
triple phosphate crystals-urine-hematology (51503)
marijuana-urine-chemistry (51089)
sperm-urine-hematology (51499)
carboxyhemoglobin-blood-blood gas (50805)
d-dimer-blood-chemistry (50915)
ntprobnp-blood-chemistry (50963)

Topic 12

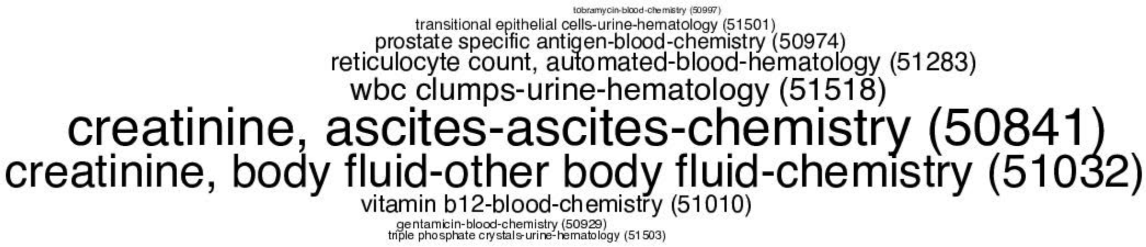
tobramycin-blood-chemistry (50997)
transitional epithelial cells-urine-hematology (51501)
prostate specific antigen-blood-chemistry (50974)
reticulocyte count, automated-blood-hematology (51283)
wbc clumps-urine-hematology (51518)
creatinine, ascites-ascites-chemistry (50841)
creatinine, body fluid-other body fluid-chemistry (51032)
vitamin b12-blood-chemistry (51010)
gentamicin-blood-chemistry (50929)
triple phosphate crystals-urine-hematology (51503)

Topic 13

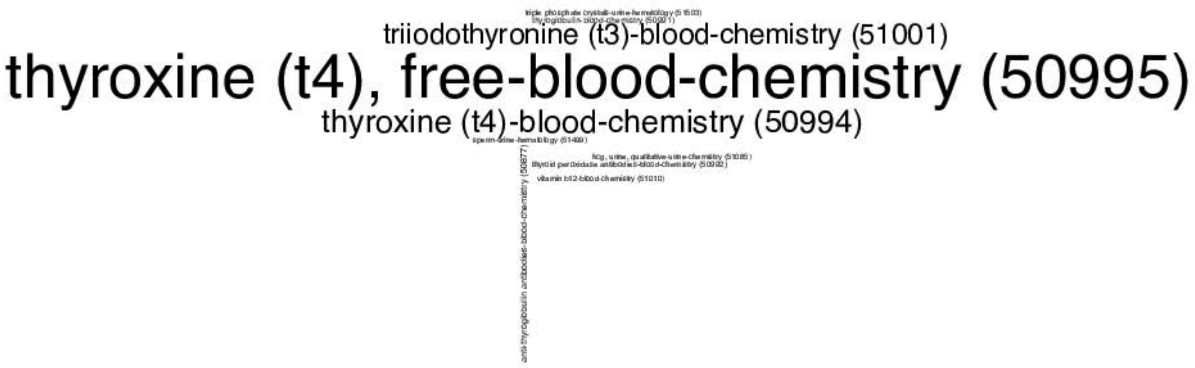
triiodothyronine (t3)-blood-chemistry (51001)
thyroxine (t4), free-blood-chemistry (50995)
thyroxine (t4)-blood-chemistry (50994)
thyroid peroxidase antibodies-blood-chemistry (50992)
vitamin b12-blood-chemistry (51010)
anti-thyroglobulin antibodies-blood-chemistry (50877)

Topic 14

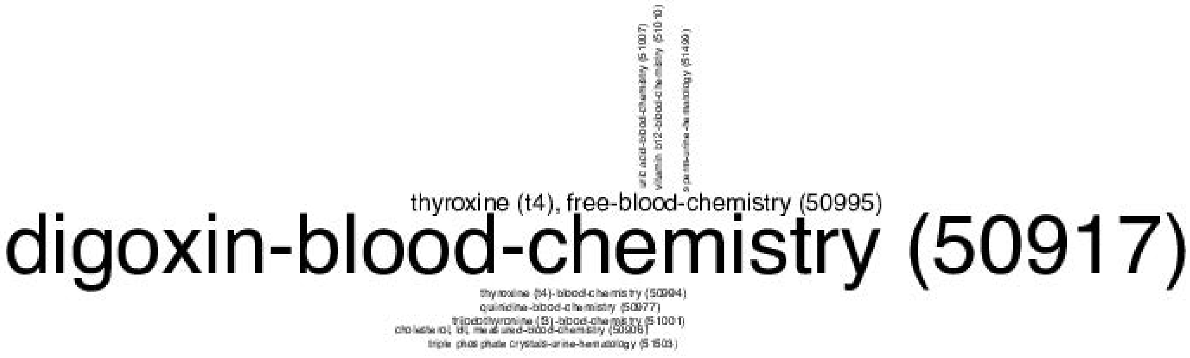
uric acid-blood-chemistry (51007)
vitamin b12-blood-chemistry (51010)
thyroxine (t4), free-blood-chemistry (50995)
digoxin-blood-chemistry (50917)
thyroxine (t4)-blood-chemistry (50994)
quinidine-blood-chemistry (50977)
triiodothyronine (t3)-blood-chemistry (51001)
cholesterol, ldl, measured-blood-chemistry (50906)
triple phosphate crystals-urine-hematology (51503)

Topic 15

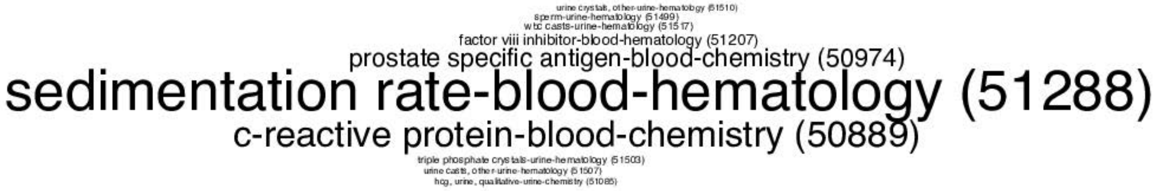
urine crystals, other-urine-hematology (51510)
sperm-urine-hematology (51499)
wbc casts-urine-hematology (51517)
factor viii inhibitor-blood-hematology (51207)
prostate specific antigen-blood-chemistry (50974)
sedimentation rate-blood-hematology (51288)
c-reactive protein-blood-chemistry (50889)
triple phosphate crystals-urine-hematology (51503)
urine casts, other-urine-hematology (51507)
hcg, urine, qualitative-urine-chemistry (51085)

Topic 16

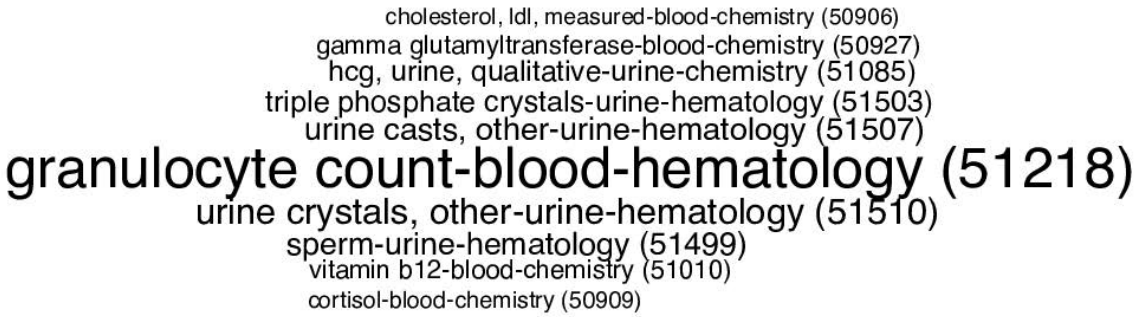
cholesterol, ldl, measured-blood-chemistry (50906)
gamma glutamyltransferase-blood-chemistry (50927)
hcg, urine, qualitative-urine-chemistry (51085)
triple phosphate crystals-urine-hematology (51503)
urine casts, other-urine-hematology (51507)
granulocyte count-blood-hematology (51218)
urine crystals, other-urine-hematology (51510)
sperm-urine-hematology (51499)
vitamin b12-blood-chemistry (51010)
cortisol-blood-chemistry (50909)

Topic 17

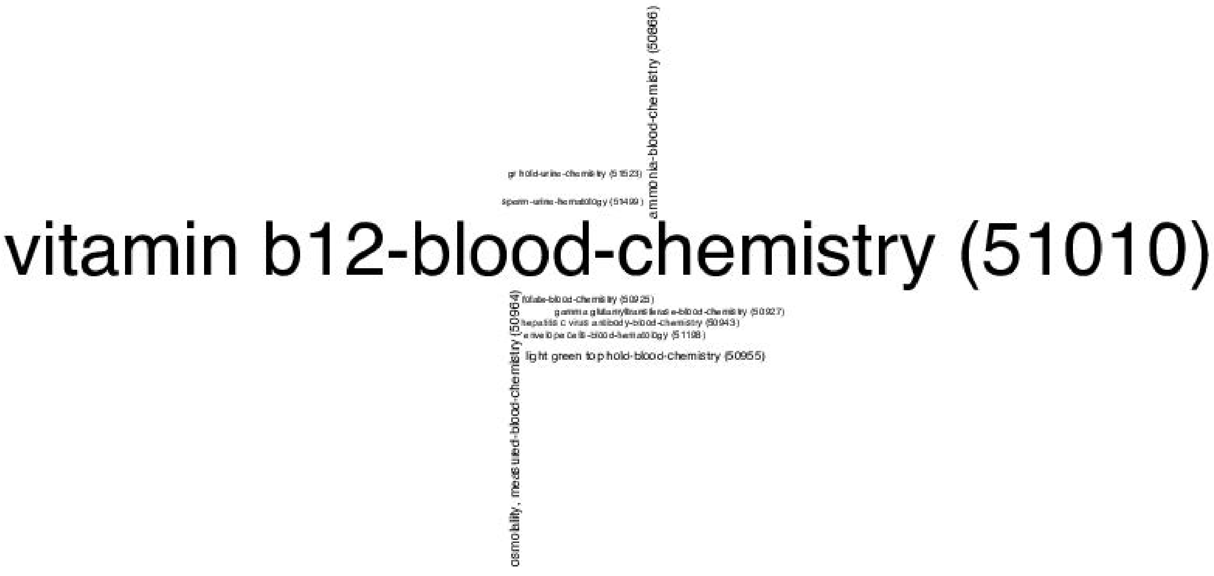
ammonia-blood-chemistry (50866)
vitamin b12-blood-chemistry (51010)
hepatitis c virus antibody-blood-chemistry (50943)
light green top hold-blood-chemistry (50955)
osmolality, measured-blood-chemistry (50964)

Topic 18

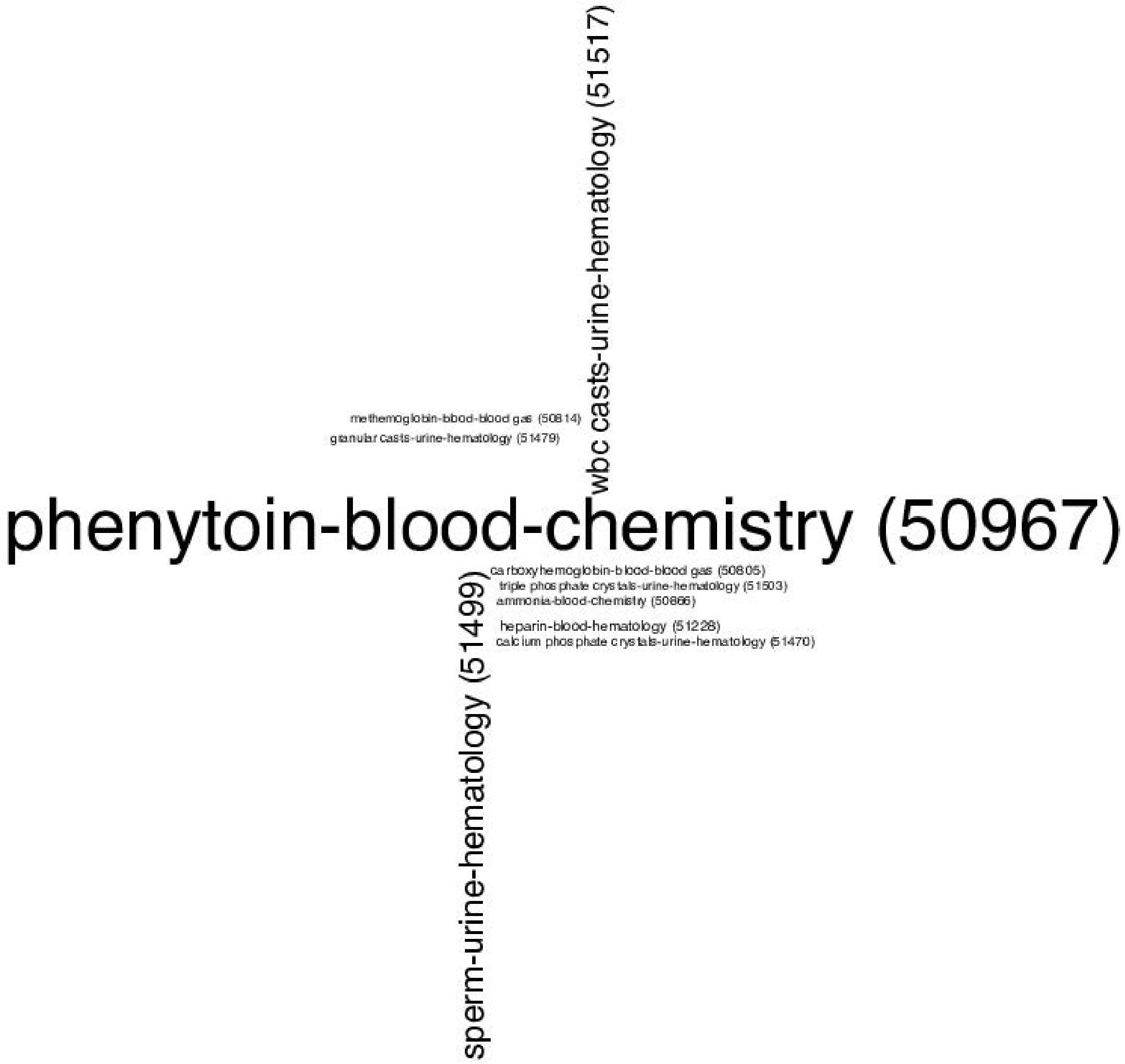
phenytoin-blood-chemistry (50967)
wbc casts-urine-hematology (51517)
sperm-urine-hematology (51499)
methemoglobin-blood-blood gas (50814)
granular casts-urine-hematology (51479)
carboxyhemoglobin-blood-blood gas (50805)
triple phosphate crystals-urine-hematology (51503)
ammonia-blood-chemistry (50866)
heparin-blood-hematology (51228)
calcium phosphate crystals-urine-hematology (51470)

Topic 19

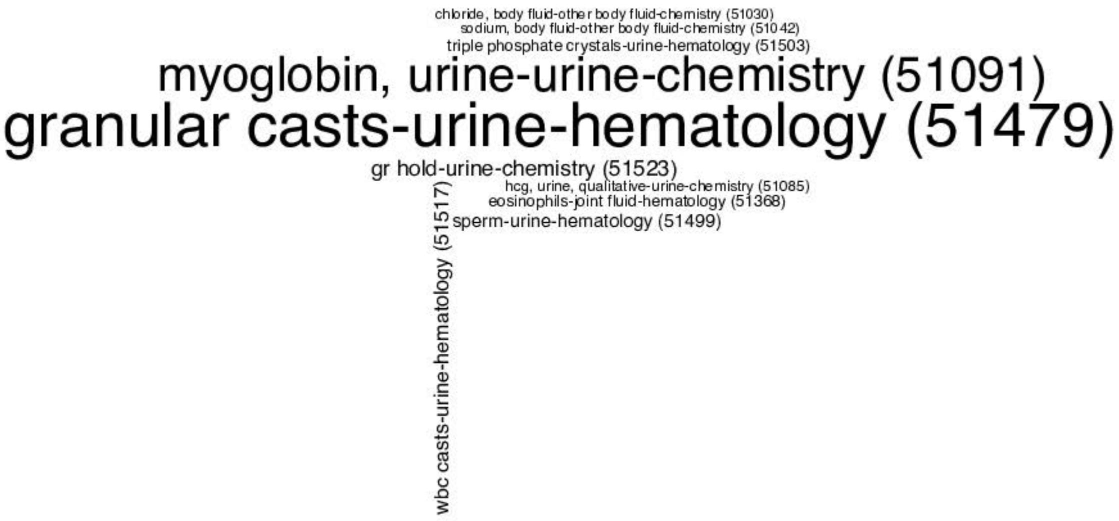
chloride, body fluid-other body fluid-chemistry (51030)
sodium, body fluid-other body fluid-chemistry (51042)
triple phosphate crystals-urine-hematology (51503)
myoglobin, urine-urine-chemistry (51091)
granular casts-urine-hematology (51479)
gr hold-urine-chemistry (51523)
hcg, urine, qualitative-urine-chemistry (51085)
eosinophils-joint fluid-hematology (51368)
sperm-urine-hematology (51499)
wbc casts-urine-hematology (51517)

Topic 20

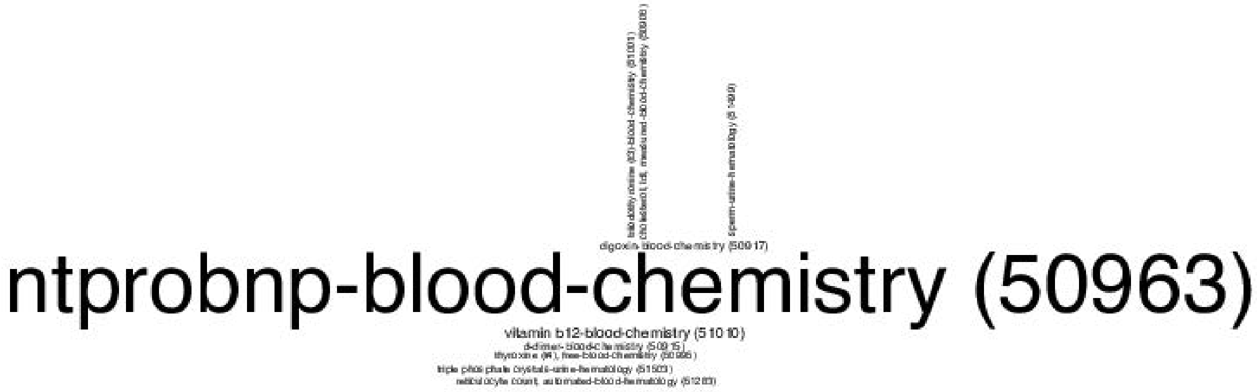

ntprobnp-blood-chemistry (50963)
digoxin-blood-chemistry (50917)
vitamin b12-blood-chemistry (51010)
d-dimer-blood-chemistry (50915)
thyroxine (t4), free-blood-chemistry (50995)
triple phosphate crystals-urine-hematology (51503)
reticulocyte count, automated-blood-hematology (51283)

Topic 21

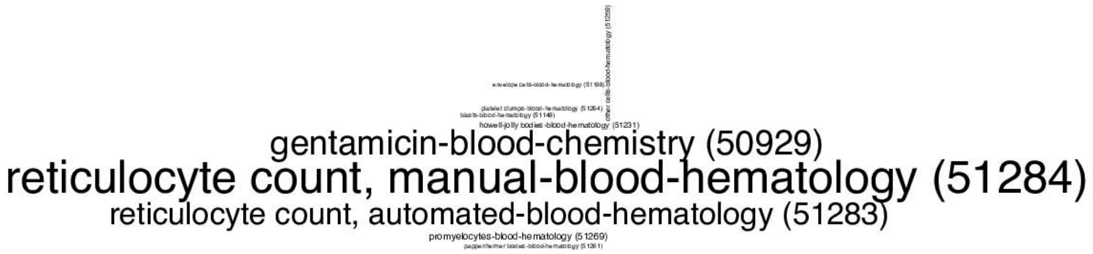
other cells-blood-hematology (51259)
envelope cells-blood-hematology (51198)
platelet clumps-blood-hematology (51264)
blasts-blood-hematology (51148)
howell-jolly bodies-blood-hematology (51231)
gentamicin-blood-chemistry (50929)
reticulocyte count, manual-blood-hematology (51284)
reticulocyte count, automated-blood-hematology (51283)
promyelocytes-blood-hematology (51269)
pappenheimer bodies-blood-hematology (51261)

Topic 22

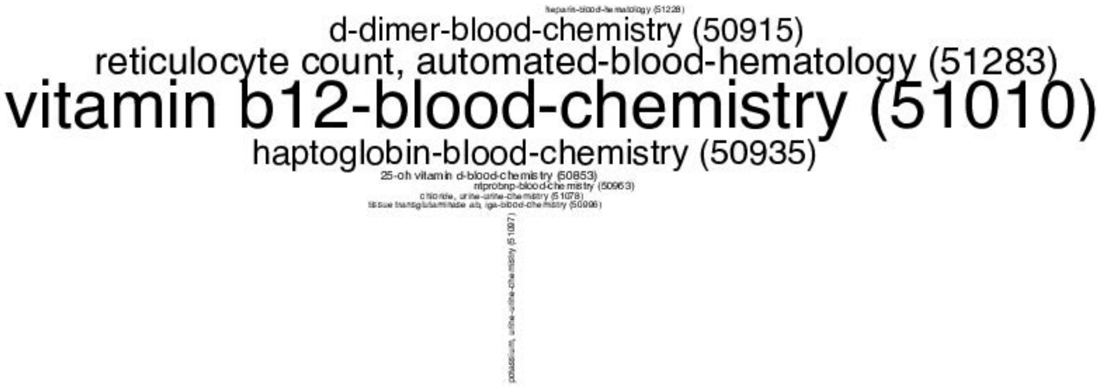
heparin-blood-hematology (51228)
d-dimer-blood-chemistry (50915)
reticulocyte count, automated-blood-hematology (51283)
vitamin b12-blood-chemistry (51010)
haptoglobin-blood-chemistry (50935)
25-oh vitamin d-blood-chemistry (50853)
ntprobnp-blood-chemistry (50963)
chloride, urine-urine-chemistry (51078)
tissue transglutaminase ab, iga-blood-chemistry (50996)
potassium, urine-urine-chemistry (51097)

Topic 23

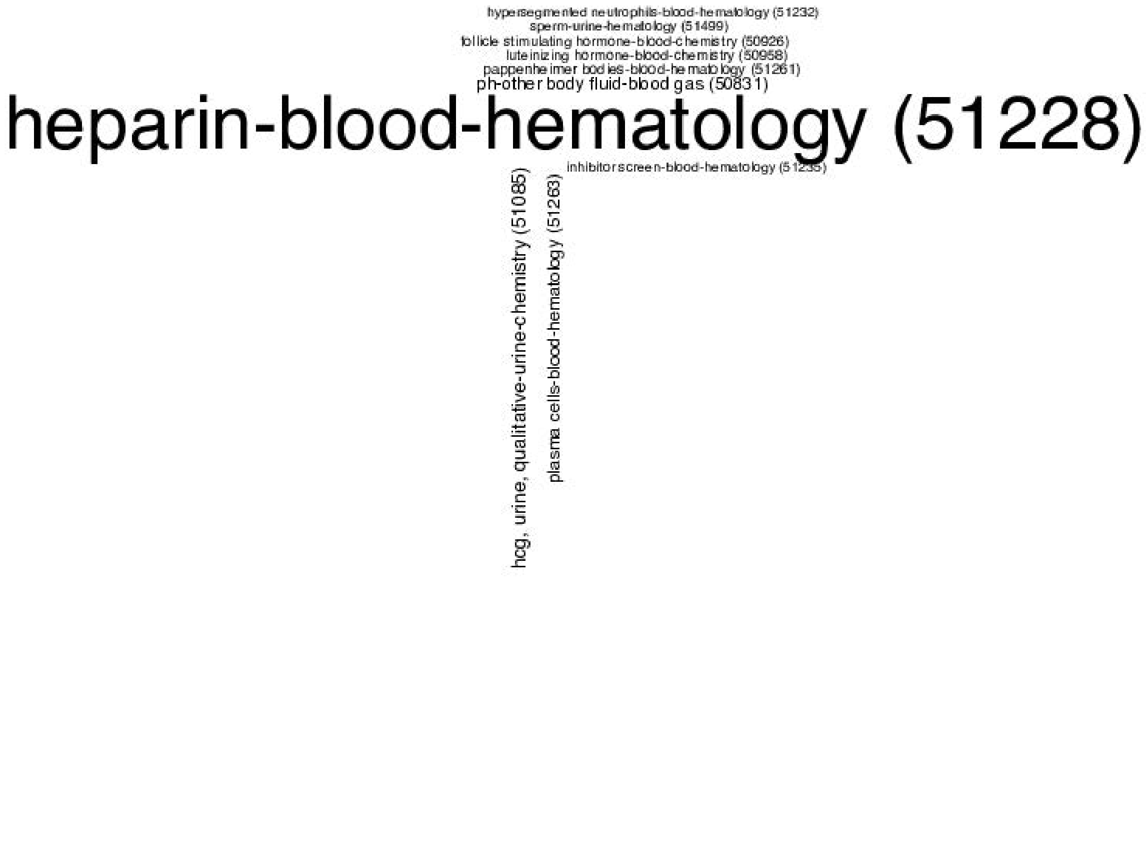
hypersegmented neutrophils-blood-hematology (51232)
sperm-urine-hematology (51499)
follicle stimulating hormone-blood-chemistry (50926)
luteinizing hormone-blood-chemistry (50958)
pappenheimer bodies-blood-hematology (51261)
ph-other body fluid-blood gas (50831)
heparin-blood-hematology (51228)
inhibitor screen-blood-hematology (51235)
hcg, urine, qualitative-urine-chemistry (51085)
plasma cells-blood-hematology (51263)

Topic 24

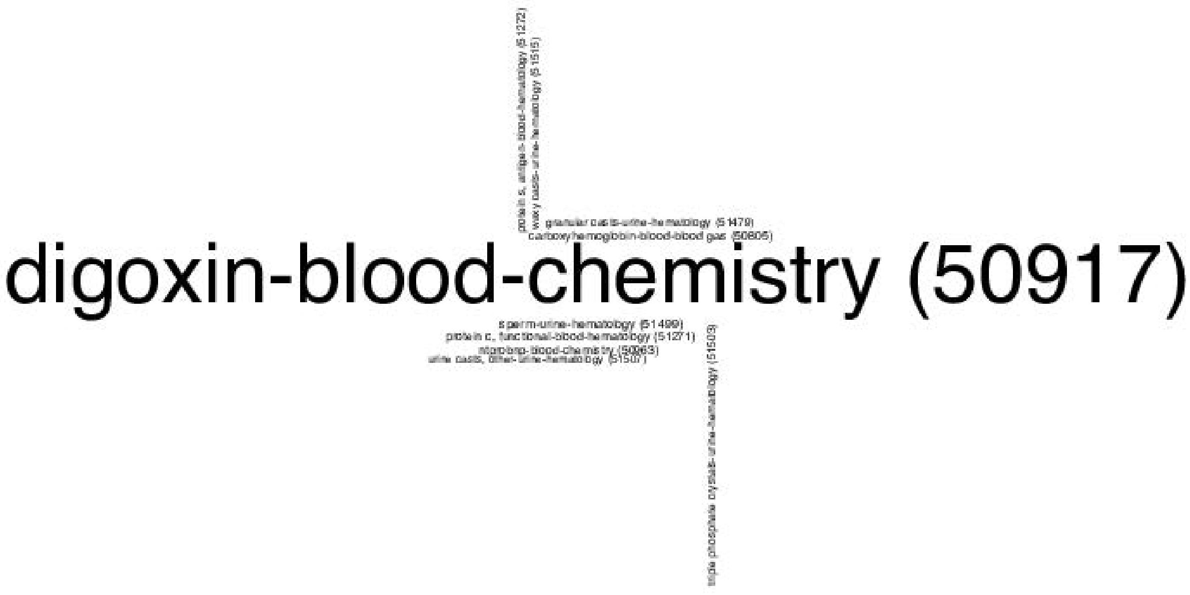
digoxin-blood-chemistry (50917)
protein s, antigen-blood-hematology (51272)
waxy casts-urine-hematology (51515)
granular casts-urine-hematology (51479)
carboxyhemoglobin-blood-blood gas (50805)
sperm-urine-hematology (51499)
protein c, functional-blood-hematology (51271)
urine casts, other-urine-hematology (51507)
triple phosphate crystals-urine-hematology (51503)

Topic 25

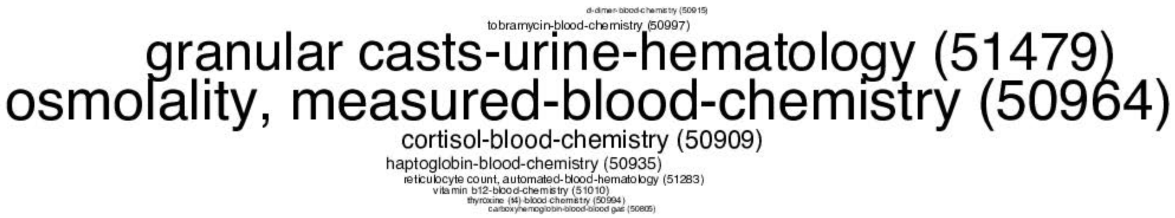
d-dimer-blood-chemistry (50915)
tobramycin-blood-chemistry (50997)
granular casts-urine-hematology (51479)
osmolality, measured-blood-chemistry (50964)
cortisol-blood-chemistry (50909)
haptoglobin-blood-chemistry (50935)
reticulocyte count, automated-blood-hematology (51283)
vitamin b12-blood-chemistry (51010)
thyroxine (t4)-blood chemistry (50994)
carboxyhemoglobin-blood-blood gas (50805)

Topic 26

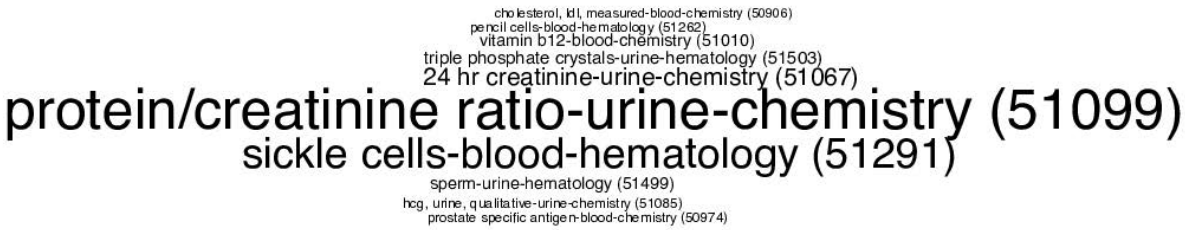
cholesterol, ldl, measured-blood-chemistry (50906)
pencil cells-blood-hematology (51262)
vitamin b12-blood-chemistry (51010)
triple phosphate crystals-urine-hematology (51503)
24 hr creatinine-urine-chemistry (51067)
protein/creatinine ratio-urine-chemistry (51099)
sickle cells-blood-hematology (51291)
sperm-urine-hematology (51499)
hcg, urine, qualitative-urine-chemistry (51085)
prostate specific antigen-blood-chemistry (50974)

Topic 27

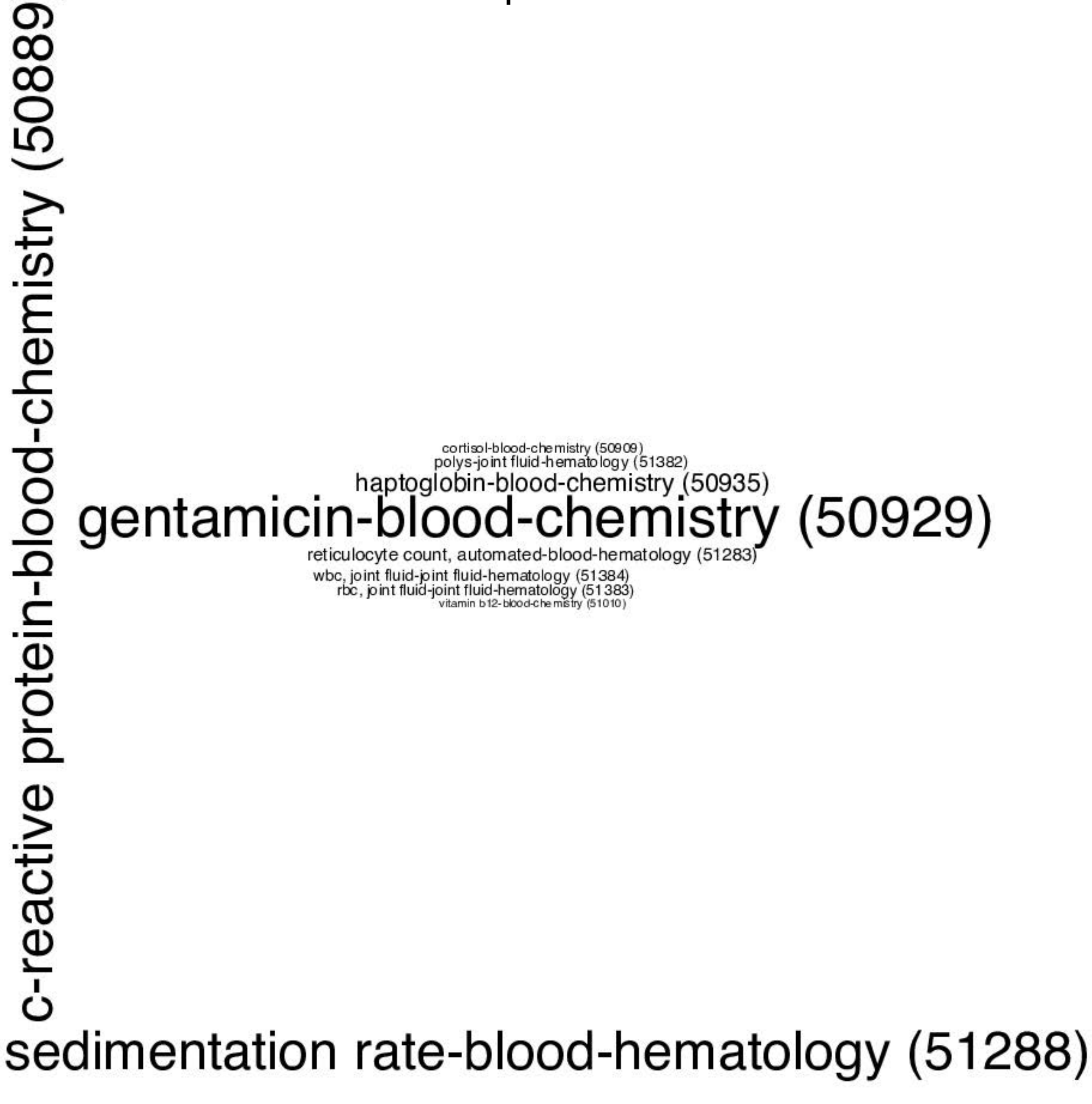
c-reactive protein-blood-chemistry (50889)
cortisol-blood-chemistry (50909)
polys-joint fluid-hematology (51382)
haptoglobin-blood-chemistry (50935)
gentamicin-blood-chemistry (50929)
reticulocyte count, automated-blood-hematology (51283)
wbc, joint fluid-joint fluid-hematology (51384)
rbc, joint fluid-joint fluid-hematology (51383)
vitamin b12-blood-chemistry (51010)
sedimentation rate-blood-hematology (51288)

Topic 28

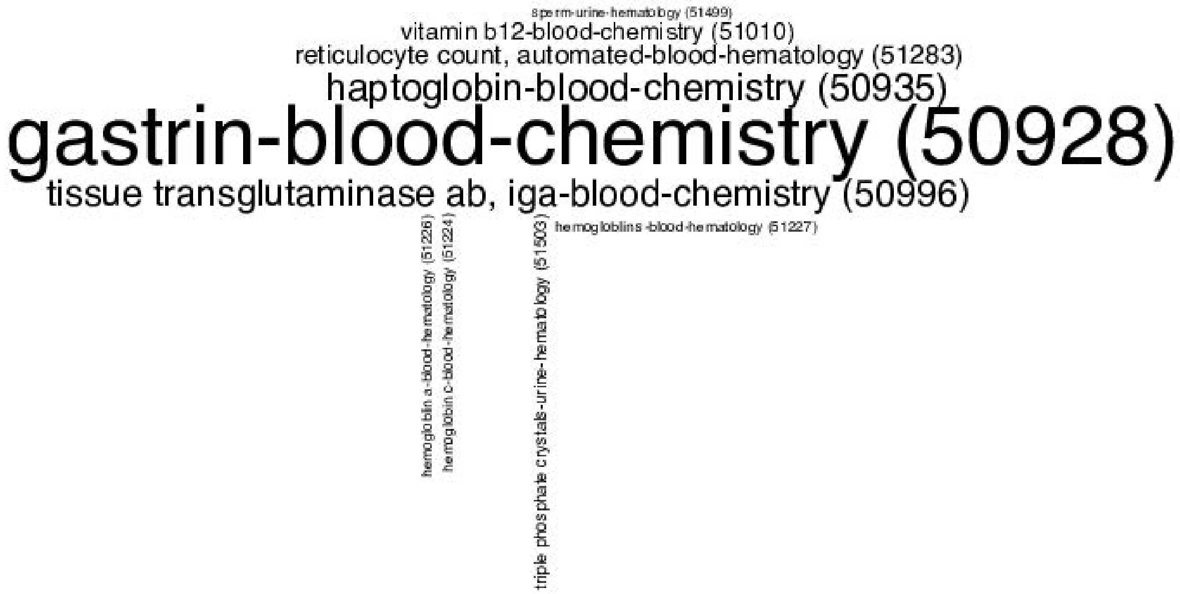

sperm-urine-hematology (51499)
vitamin b12-blood-chemistry (51010)
reticulocyte count, automated-blood-hematology (51283)
haptoglobin-blood-chemistry (50935)
gastrin-blood-chemistry (50928)
tissue transglutaminase ab, iga-blood-chemistry (50996)
hemoglobins-blood-hematology (51227)
hemoglobin a-blood-hematology (51226)
hemoglobin c-blood-hematology (51224)
triple phosphate crystals-urine-hematology (51503)

Topic 29

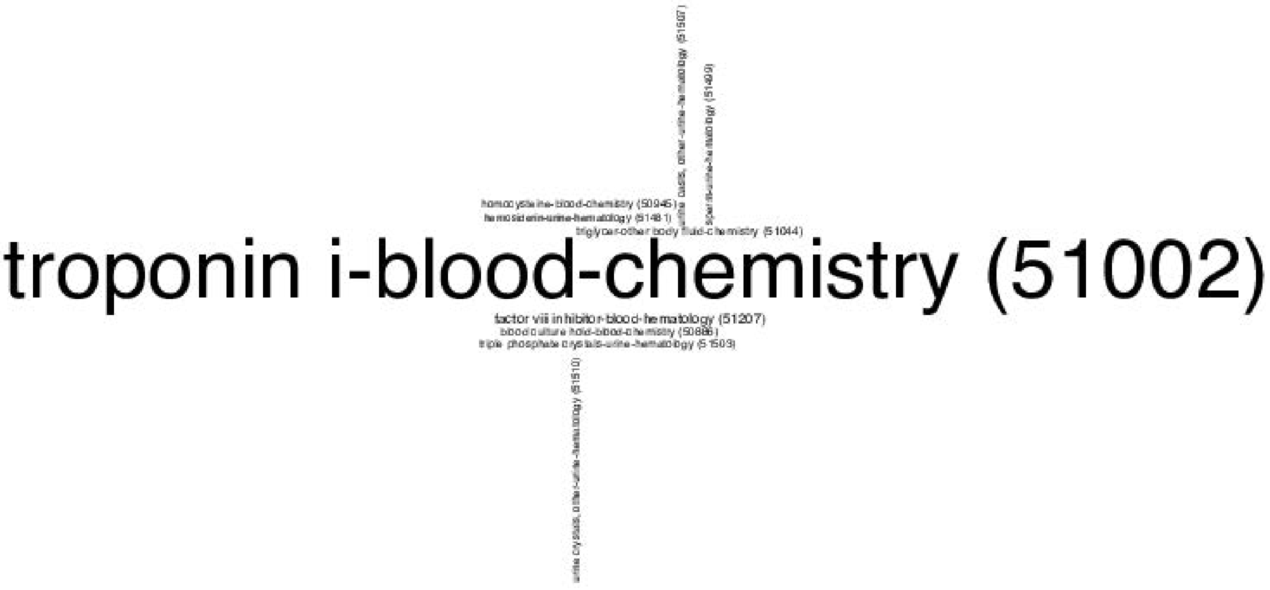
troponin i-blood-chemistry (51002)
homocysteine-blood-chemistry (50945)
hemosiderin-urine-hematology (51481)
triglycer-other body fluid-chemistry (51044)
urine casts, other-urine-hematology (51507)
sperm-urine-hematology (51499)
factor viii inhibitor-blood-hematology (51207)
blood culture hold-blood-chemistry (50886)
triple phosphate crystals-urine-hematology (51503)
urine crystals, other-urine-hematology (51510)

Topic 30

prostate specific antigen-blood-chemistry (50974)
cortisol-blood-chemistry (50909)
h/o smear-blood-hematology (51219)
tissue transglutaminase ab, iga-blood-chemistry (50996)

parathyroid hormone-blood-chemistry (50965)

vitamin b12-blood-chemistry (51010)
haptoglobin-blood-chemistry (50935)
sperm-urine-hematology (51499)
uric acid-blood-chemistry (51007)
eosinophil count-blood-hematology (51199)

Topic 31

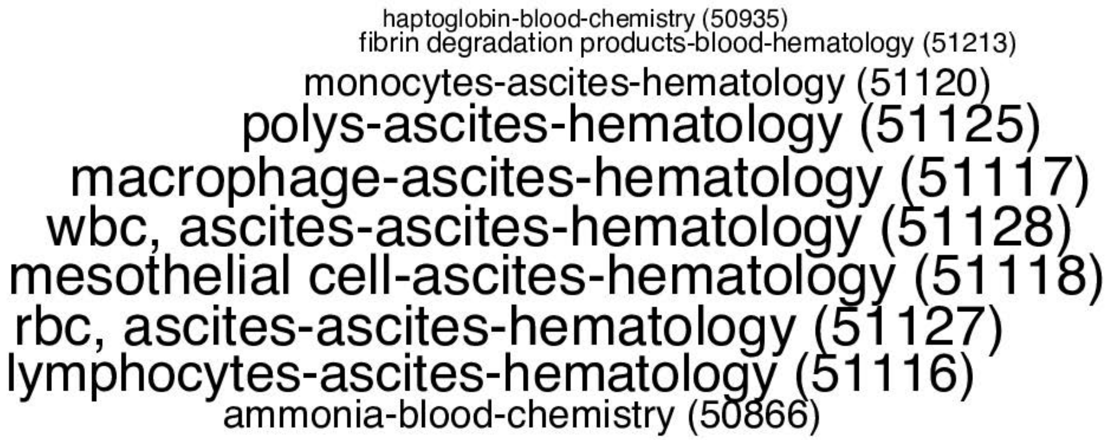
haptoglobin-blood-chemistry (50935)
fibrin degradation products-blood-hematology (51213)
monocytes-ascites-hematology (51120)
polys-ascites-hematology (51125)
macrophage-ascites-hematology (51117)
wbc, ascites-ascites-hematology (51128)
mesothelial cell-ascites-hematology (51118)
rbc, ascites-ascites-hematology (51127)
lymphocytes-ascites-hematology (51116)
ammonia-blood-chemistry (50866)

Topic 32

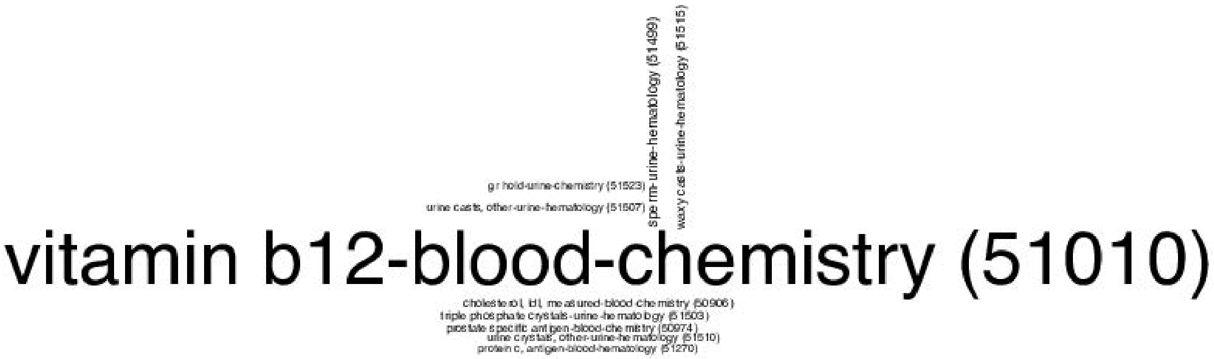
sperm-urine-hematology (51499)
waxy casts-urine-hematology (51515)
gr hold-urine-chemistry (51523)
urine casts, other-urine-hematology (51507)
vitamin b12-blood-chemistry (51010)
cholesterol, ldl, measured-blood chemistry (50906)
triple phosphate crystals-urine-hematology (51503)
prostate specific antigen-blood-chemistry (50974)
urine crystals, other-urine-hematology (51510)
protein c, antigen-blood-hematology (51270)

Topic 33

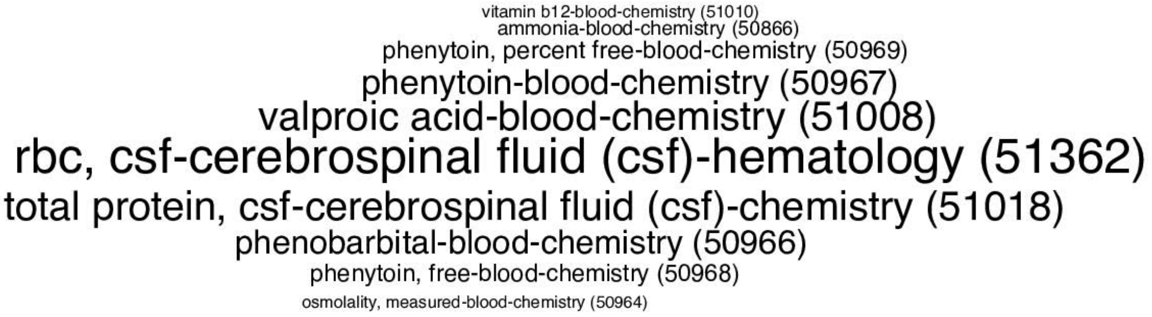
vitamin b12-blood-chemistry (51010)
ammonia-blood-chemistry (50866)
phenytoin, percent free-blood-chemistry (50969)
phenytoin-blood-chemistry (50967)
valproic acid-blood-chemistry (51008)
rbc, csf-cerebrospinal fluid (csf)-hematology (51362)
total protein, csf-cerebrospinal fluid (csf)-chemistry (51018)
phenobarbital-blood-chemistry (50966)
phenytoin, free-blood-chemistry (50968)
osmolality, measured-blood-chemistry (50964)

Topic 34

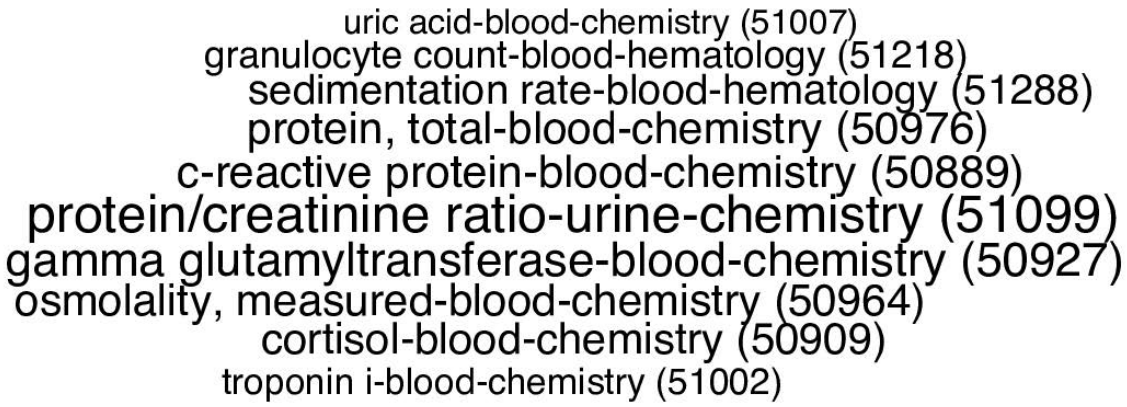
uric acid-blood-chemistry (51007)
granulocyte count-blood-hematology (51218)
sedimentation rate-blood-hematology (51288)
protein, total-blood-chemistry (50976)
c-reactive protein-blood-chemistry (50889)
protein/creatinine ratio-urine-chemistry (51099)
gamma glutamyltransferase-blood-chemistry (50927)
osmolality, measured-blood-chemistry (50964)
cortisol-blood-chemistry (50909)
troponin i-blood-chemistry (51002)

Topic 35

other-pleural-hematology (51453)
eosinophils-pleural-hematology (51444)
macrophages-pleural-hematology (51447)
polys-pleural-hematology (51455)
protein, total-blood-chemistry (50976)
wbc, pleural-pleural-hematology (51458)
rbc, pleural-pleural-hematology (51457)
lymphocytes-pleural-hematology (51446)
mesothelial cells-pleural-hematology (51448)
monos-pleural-hematology (51450)

Topic 36

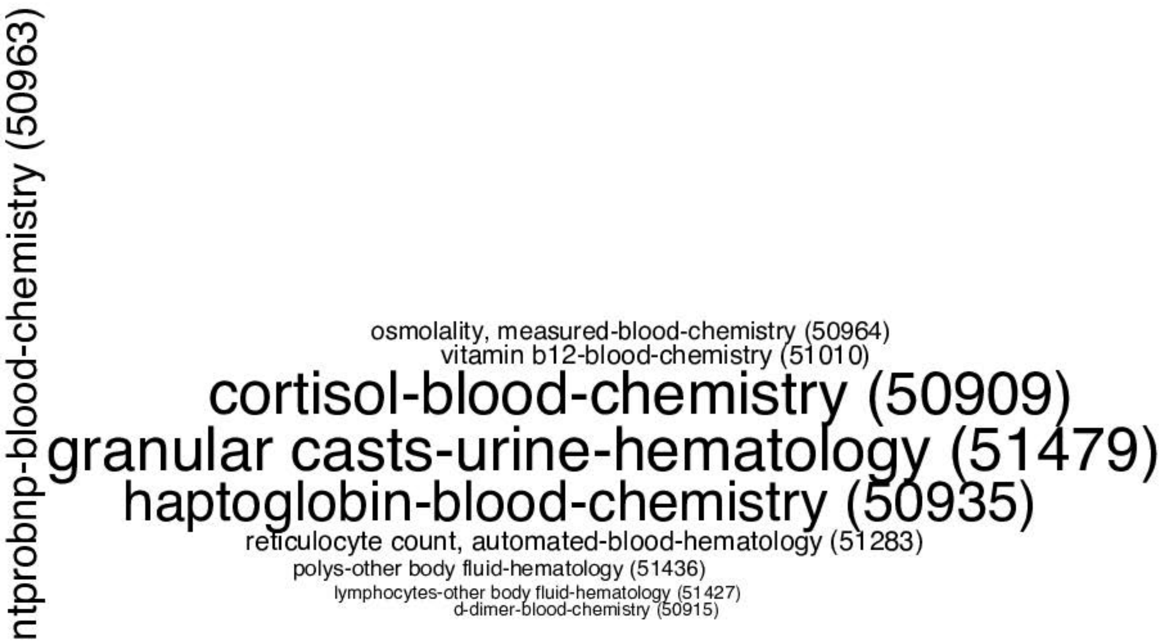
ntprobnp-blood-chemistry (50963)
osmolality, measured-blood-chemistry (50964)
vitamin b12-blood-chemistry (51010)
cortisol-blood-chemistry (50909)
granular casts-urine-hematology (51479)
haptoglobin-blood-chemistry (50935)
reticulocyte count, automated-blood-hematology (51283)
polys-other body fluid-hematology (51436)
lymphocytes-other body fluid-hematology (51427)
d-dimer-blood-chemistry (50915)

Topic 37

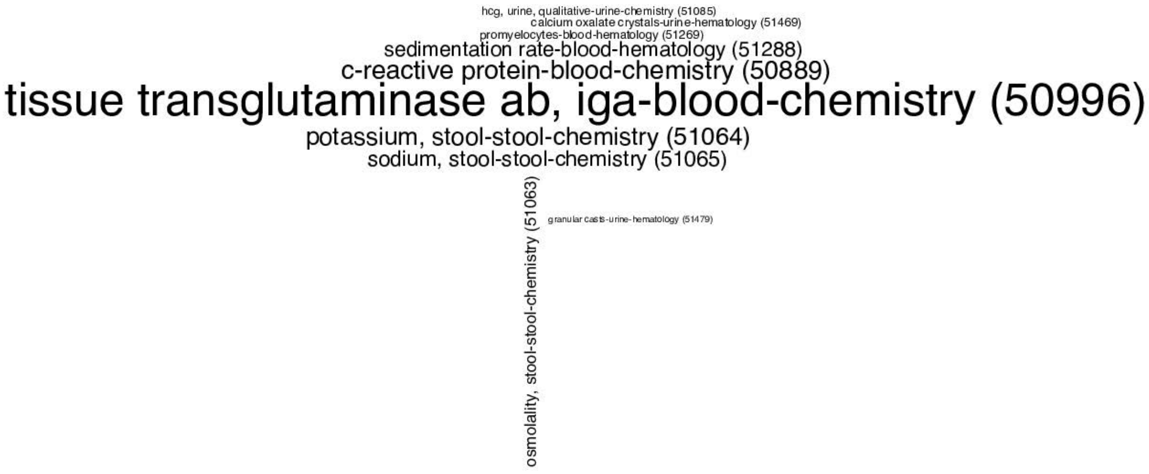
hcg, urine, qualitative-urine-chemistry (51085)
calcium oxalate crystals-urine-hematology (51469)
promyelocytes-blood-hematology (51269)
sedimentation rate-blood-hematology (51288)
c-reactive protein-blood-chemistry (50889)
tissue transglutaminase ab, iga-blood-chemistry (50996)
potassium, stool-stool-chemistry (51064)
sodium, stool-stool-chemistry (51065)
osmolality, stool-stool-chemistry (51063)
granular casts-urine-hematology (51479)

Topic 38

cholesterol, ldl, measured-blood-chemistry (50906)
urine casts, other-urine-hematology (51507)
vitamin b12-blood-chemistry (51010)
sperm-urine-hematology (51499)
prostate specific antigen-blood-chemistry (50974)
gr hold-urine-chemistry (51523)
triple phosphate crystals-urine-hematology (51503)
wbc casts-urine-hematology (51517)
cortisol-blood-chemistry (50909)
pencil cells-blood-hematology (51262)

Topic 39

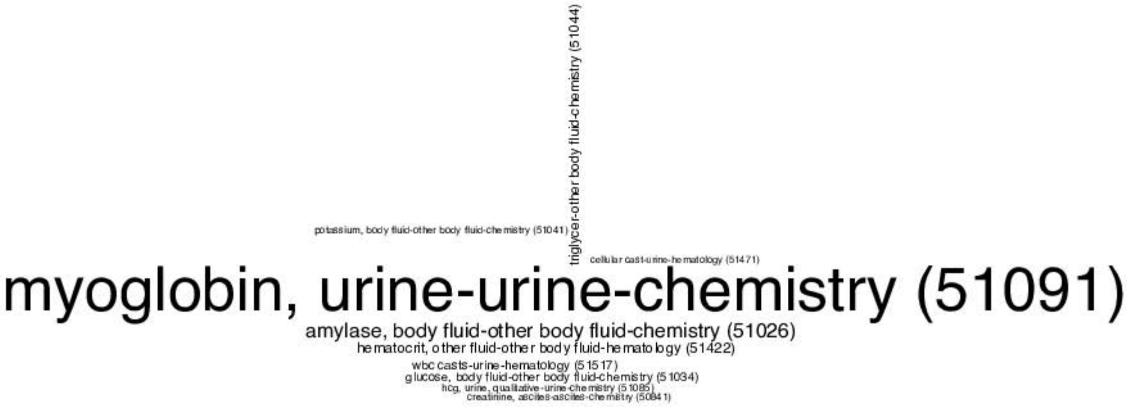
triglycer-other body fluid-chemistry (51044)
potassium, body fluid-other body fluid-chemistry (51041)
cellular cast-urine-hematology (51471)
myoglobin, urine-urine-chemistry (51091)
amylase, body fluid-other body fluid-chemistry (51026)
hematocrit, other fluid-other body fluid-hematology (51422)
wbc casts-urine-hematology (51517)
glucose, body fluid-other body fluid-chemistry (51034)
hcg, urine, qualitative-urine-chemistry (51085)
creatinine, ascites-ascites-chemistry (50841)

Topic 40

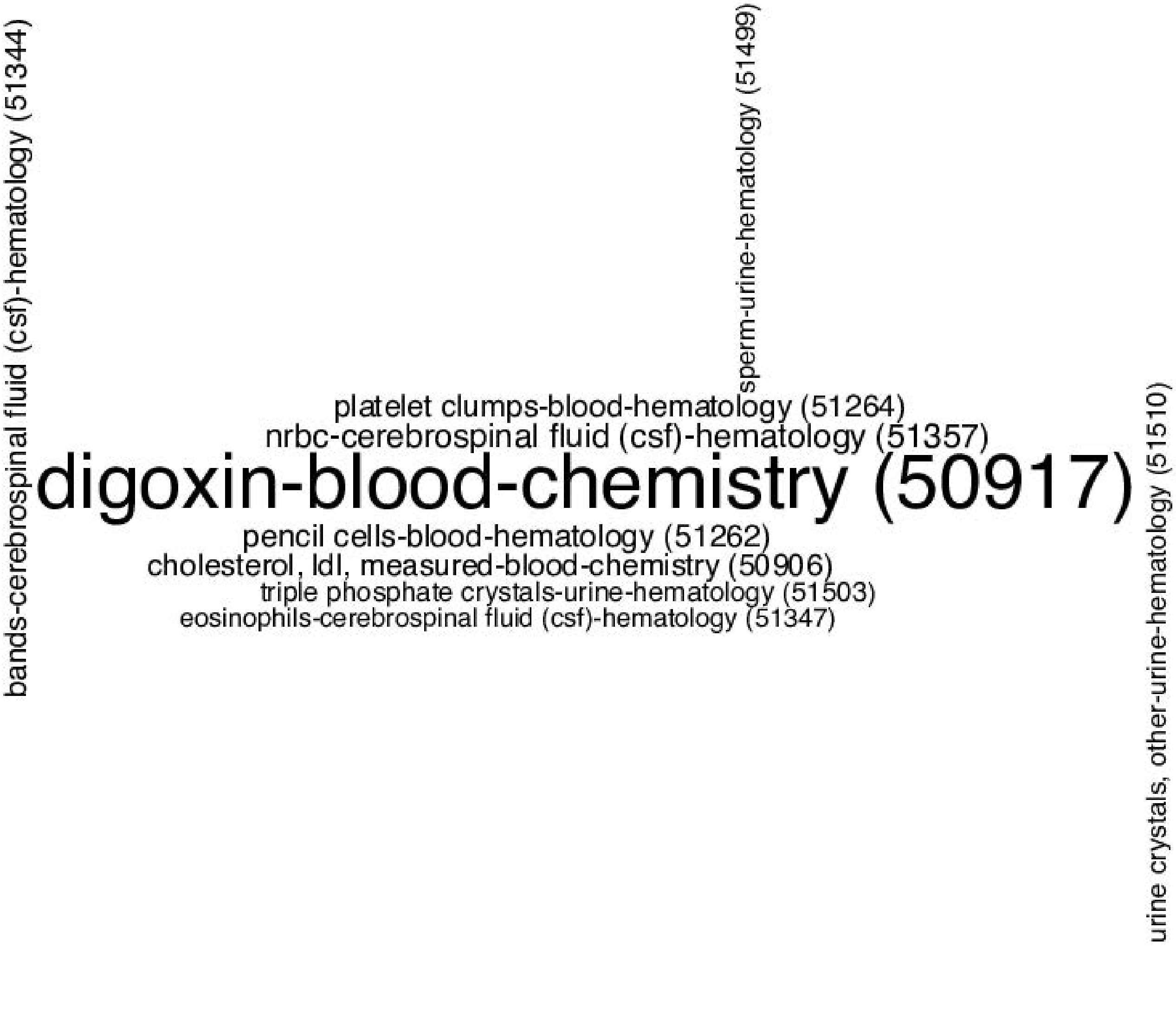
bands-cerebrospinal fluid (csf)-hematology (51344)
sperm-urine-hematology (51499)
platelet clumps-blood-hematology (51264)
nrbc-cerebrospinal fluid (csf)-hematology (51357)
digoxin-blood-chemistry (50917)
pencil cells-blood-hematology (51262)
cholesterol, ldl, measured-blood-chemistry (50906)
triple phosphate crystals-urine-hematology (51503)
eosinophils-cerebrospinal fluid (csf)-hematology (51347)
urine crystals, other-urine-hematology (51510)

Topic 41

cholesterol, ldl, measured-blood-chemistry (50906)
urine crystals, other-urine-hematology (51510)
urine casts, other-urine-hematology (51507)
sperm-urine-hematology (51499)
triple phosphate crystals-urine-hematology (51503)
vitamin b12-blood-chemistry (51010)
prostate specific antigen-blood-chemistry (50974)
hcg, urine, qualitative-urine-chemistry (51085)
cellular cast-urine-hematology (51471)
gray top hold (plasma)-blood-chemistry (50932)

Topic 42

cd3 absolute count-blood-hematology (51175)
haptoglobin-blood-chemistry (50935)
absolute cd8 count-blood-hematology (51132)
cd4 cells, percent-blood-hematology (51180)
wbc count-blood-hematology (51300)
lymphocytes, percent-blood-hematology (51245)
absolute cd4 count-blood-hematology (51131)
cd4/cd8 ratio-blood-hematology (51181)
absolute cd3 count-blood-hematology (51130)
cd8 cells, percent-blood-hematology (51194)

Topic 43

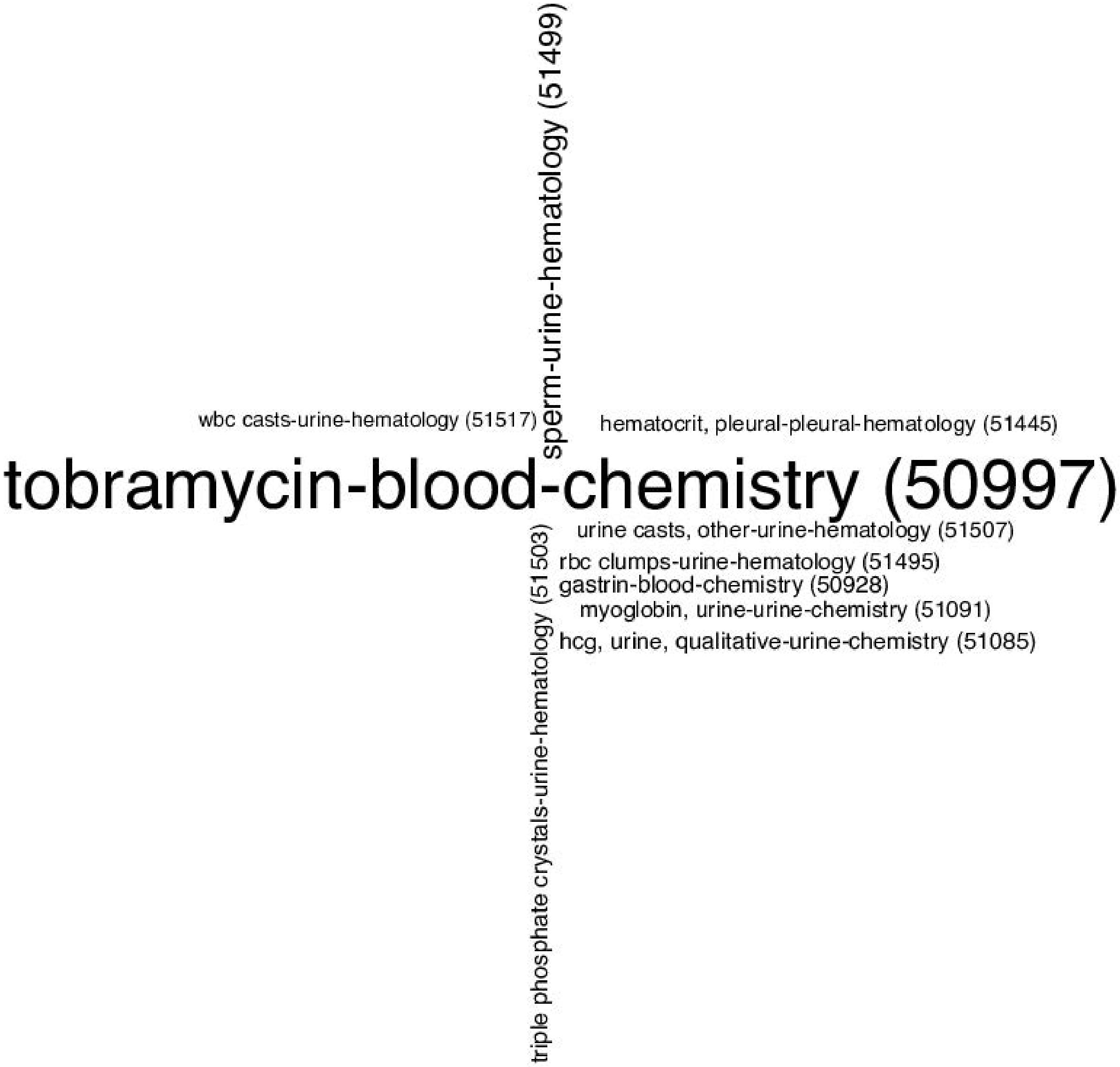

sperm-urine-hematology (51499)
wbc casts-urine-hematology (51517)
hematocrit, pleural-pleural-hematology (51445)
tobramycin-blood-chemistry (50997)
urine casts, other-urine-hematology (51507)
rbc clumps-urine-hematology (51495)
gastrin-blood-chemistry (50928)
myoglobin, urine-urine-chemistry (51091)
hcg, urine, qualitative-urine-chemistry (51085)
triple phosphate crystals-urine-hematology (51503)

Topic 44

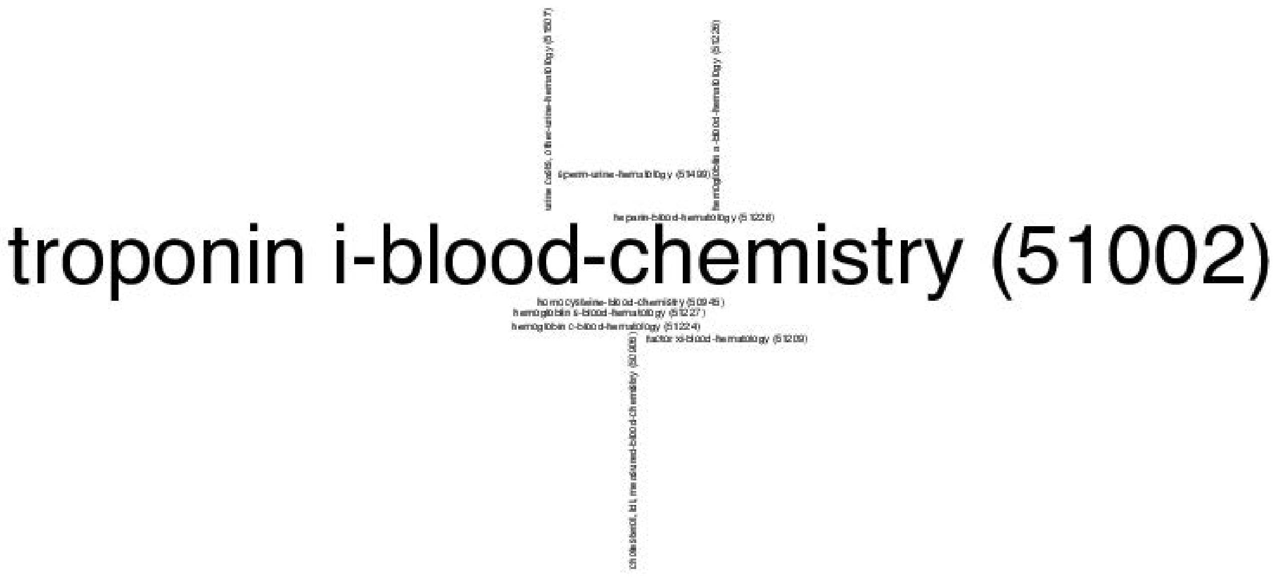
sperm-urine-hematology (51499)
heparin-blood-hematology (51226)
troponin i-blood-chemistry (51002)
homocysteine-blood-chemistry (50945)
hemoglobin s-blood-hematology (51227)
hemoglobin c-blood-hematology (51224)
factor xi-blood-hematology (51209)

Topic 45

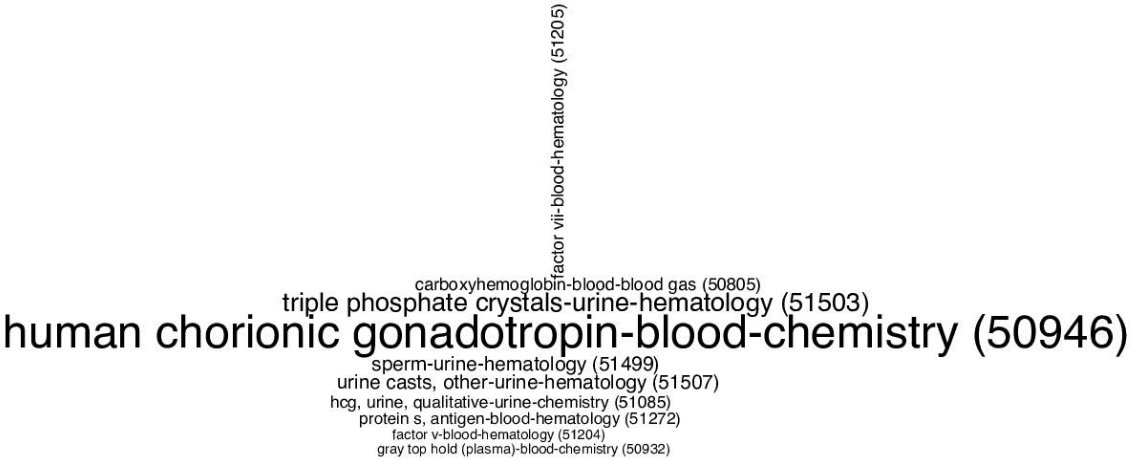
factor vii-blood-hematology (51205)
carboxyhemoglobin-blood-blood gas (50805)
triple phosphate crystals-urine-hematology (51503)
human chorionic gonadotropin-blood-chemistry (50946)
sperm-urine-hematology (51499)
urine casts, other-urine-hematology (51507)
hcg, urine, qualitative-urine-chemistry (51085)
protein s, antigen-blood-hematology (51272)
factor v-blood-hematology (51204)
gray top hold (plasma)-blood-chemistry (50932)

Topic 46

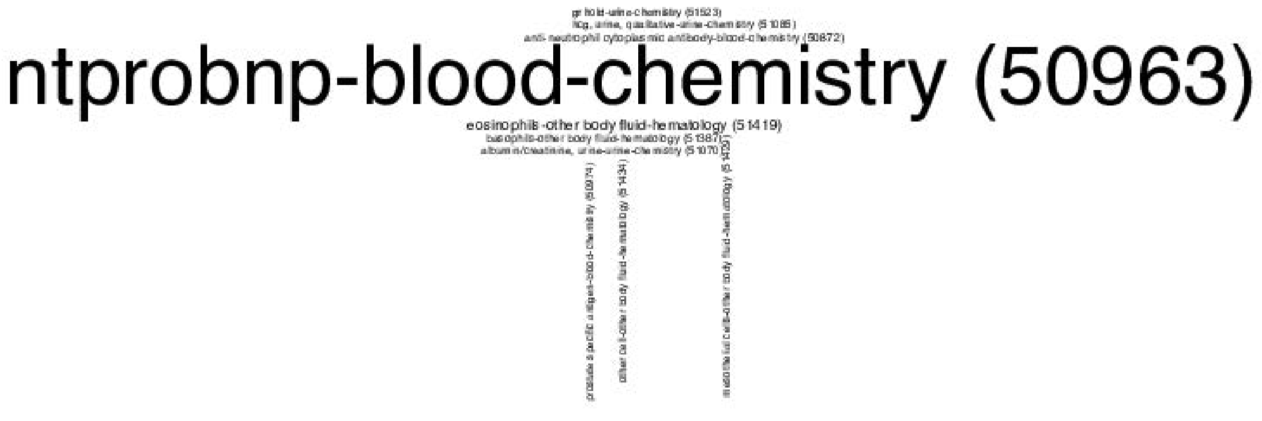
gr hold-urine-chemistry (51523)
hcg, urine, qualitative-urine-chemistry (51085)
anti-neutrophil cytoplasmic antibody-blood-chemistry (50872)
ntprobnp-blood-chemistry (50963)
eosinophils-other body fluid-hematology (51419)
basophils-other body fluid-hematology (51387)
albumin/creatinine, urine-urine-chemistry (51070)
prostate specific antigen-blood-chemistry (50974)
other cell-other body fluid-hematology (51434)

Topic 47

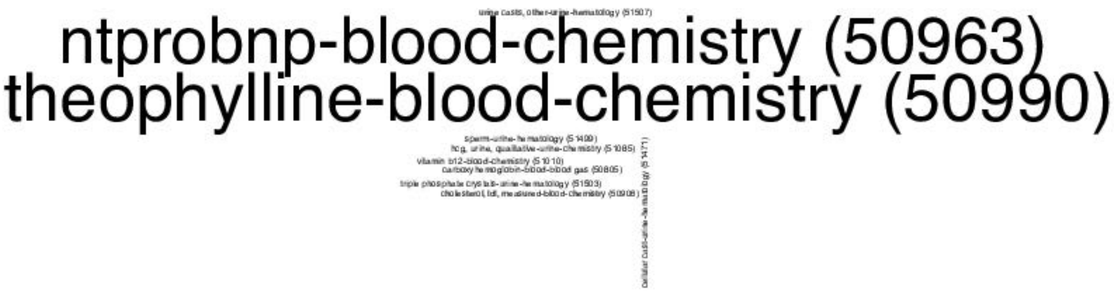

urine casts, other-urine-hematology (51507)
ntprobnp-blood-chemistry (50963)
theophylline-blood-chemistry (50990)
sperm-urine-hematology (51499)
hcg, urine, qualitative-urine-chemistry (51085)
vitamin b12-blood-chemistry (51010)
carboxyhemoglobin-blood-blood gas (50805)
triple phosphate crystals-urine-hematology (51503)
cholesterol, ldl, measured-blood-chemistry (50908)
cellular casts-urine-hematology (51471)

Topic 48

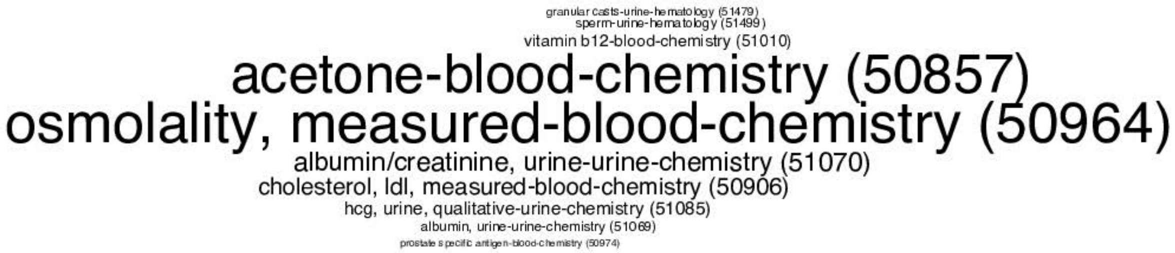
granular casts-urine-hematology (51479)
sperm-urine-hematology (51499)
vitamin b12-blood-chemistry (51010)
acetone-blood-chemistry (50857)
osmolality, measured-blood-chemistry (50964)
albumin/creatinine, urine-urine-chemistry (51070)
cholesterol, ldl, measured-blood-chemistry (50906)
hcg, urine, qualitative-urine-chemistry (51085)
albumin, urine-urine-chemistry (51069)
prostate specific antigen-blood-chemistry (50974)

Topic 49

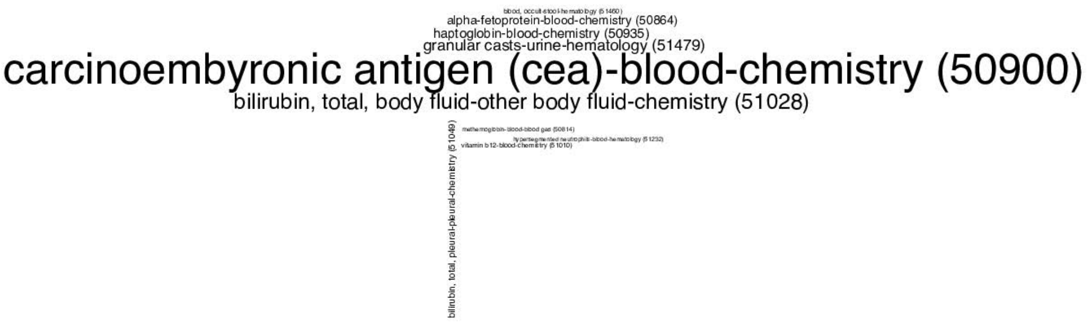
blood, occult-stool-hematology (51460)
alpha-fetoprotein-blood-chemistry (50864)
haptoglobin-blood-chemistry (50935)
granular casts-urine-hematology (51479)
carcinoembyronic antigen (cea)-blood-chemistry (50900)
bilirubin, total, body fluid-other body fluid-chemistry (51028)
bilirubin, total, pleural-pleural-chemistry (51049)
methemoglobin-blood-blood gas (50814)
hypersegmented neutrophils-blood-hematology (51232)
vitamin b12-blood-chemistry (51010)

Topic 50

reticulocyte count, automated-blood-hematology (51283)
haptoglobin-blood-chemistry (50935)
fibrin degradation products-blood-hematology (51213)
protein, total-blood-chemistry (50976)
granulocyte count-blood-hematology (51218)
promyelocytes-blood-hematology (51269)
blasts-blood-hematology (51148)
other cells-blood-hematology (51259)
granular casts-urine-hematology (51479)
uric acid-blood-chemistry (51007)

Topic 51

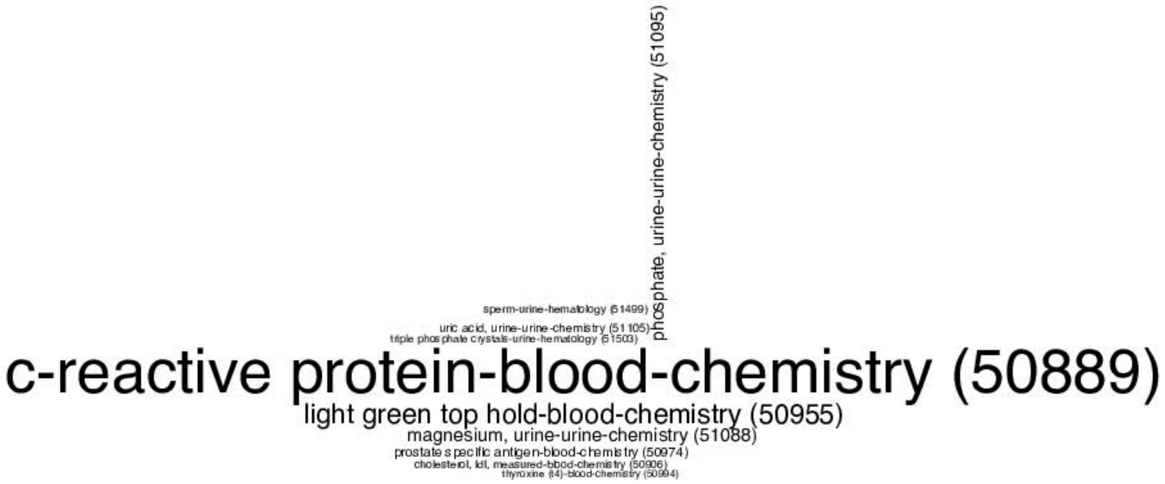
phosphate, urine-urine-chemistry (51095)
sperm-urine-hematology (51499)
uric acid, urine-urine-chemistry (51105)
triple phosphate crystals-urine-hematology (51503)
c-reactive protein-blood-chemistry (50889)
light green top hold-blood-chemistry (50955)
magnesium, urine-urine-chemistry (51088)
prostate specific antigen-blood-chemistry (50974)
cholesterol, ldl, measured-blood-chemistry (50906)
thyroxine (t4)-blood-chemistry (50994)

Topic 52

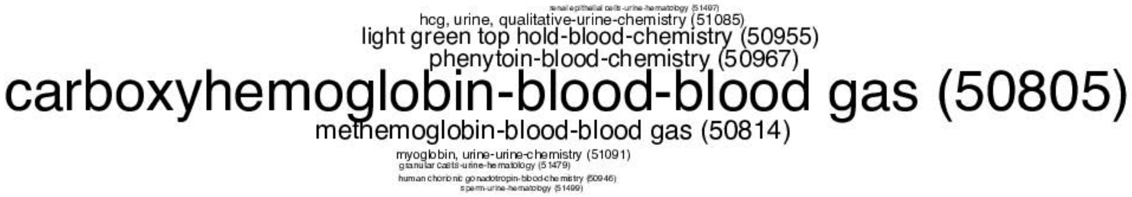

renal epithelial cells-urine-hematology (51497)
hcg, urine, qualitative-urine-chemistry (51085)
light green top hold-blood-chemistry (50955)
phenytoin-blood-chemistry (50967)
carboxyhemoglobin-blood-blood gas (50805)
methemoglobin-blood-blood gas (50814)
myoglobin, urine-urine-chemistry (51091)
granular casts-urine-hematology (51479)
human chorionic gonadotropin-blood-chemistry (50946)
sperm-urine-hematology (51499)

Topic 53

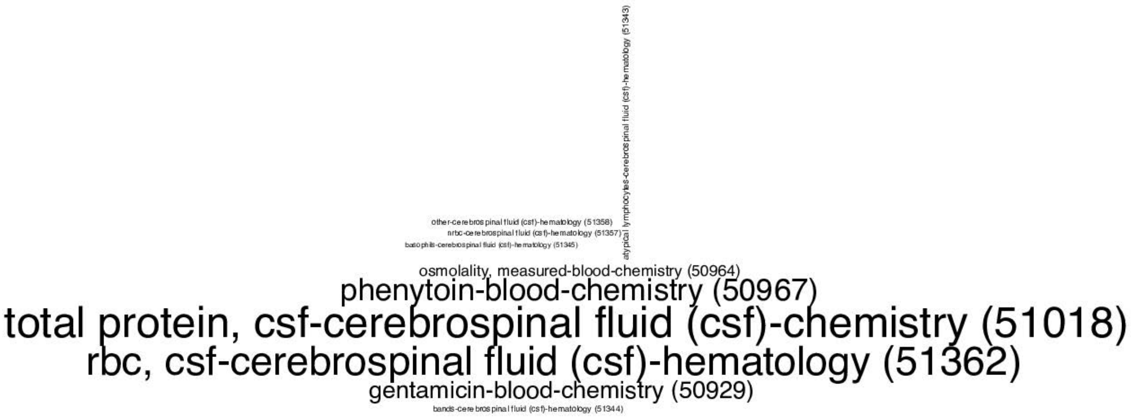
atypical lymphocytes-cerebrospinal fluid (csf)-hematology (51343)
other-cerebrospinal fluid (csf)-hematology (51358)
nrbc-cerebrospinal fluid (csf)-hematology (51357)
basophils-cerebrospinal fluid (csf)-hematology (51345)
osmolality, measured-blood-chemistry (50964)
phenytoin-blood-chemistry (50967)
total protein, csf-cerebrospinal fluid (csf)-chemistry (51018)
rbc, csf-cerebrospinal fluid (csf)-hematology (51362)
gentamicin-blood-chemistry (50929)
bands-cerebrospinal fluid (csf)-hematology (51344)

Topic 54

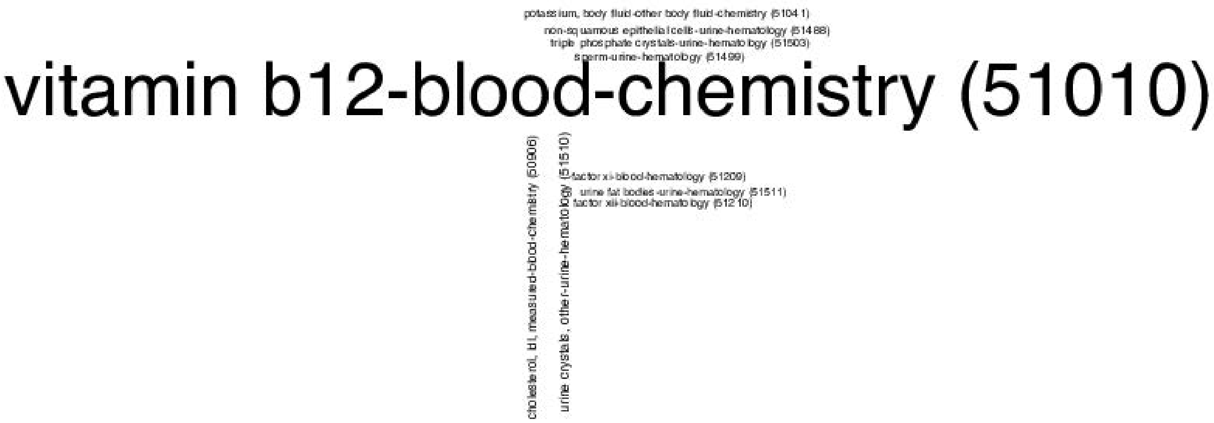
potassium, body fluid-other body fluid-chemistry (51041)
non-squamous epithelial cells-urine-hematology (51488)
triple phosphate crystals-urine-hematology (51503)
sperm-urine-hematology (51499)
vitamin b12-blood-chemistry (51010)
cholesterol, ldl, measured-blood-chemistry (50906)
urine crystals, other-urine-hematology (51510)
factor xi-blood-hematology (51209)
urine fat bodies-urine-hematology (51511)
factor xii-blood-hematology (51210)

Topic 55

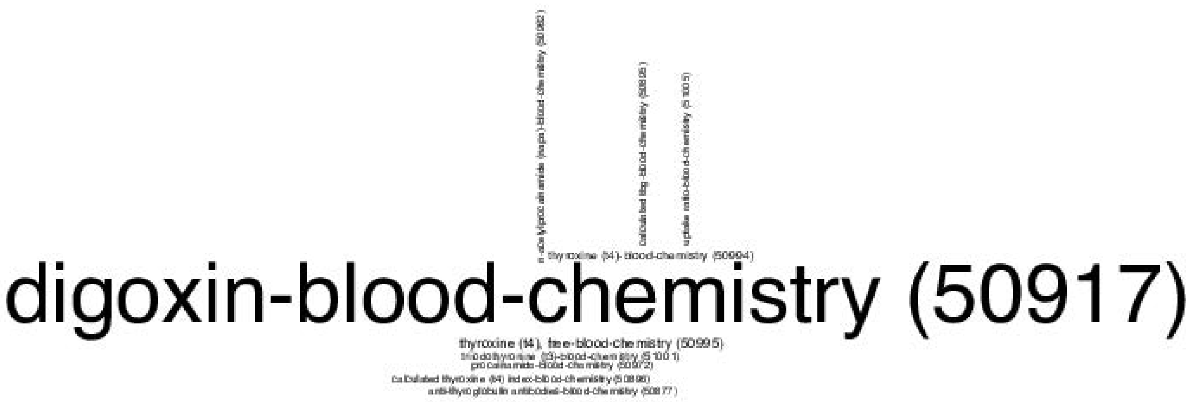
digoxin-blood-chemistry (50917)
thyroxine (t4)-blood-chemistry (50994)
thyroxine (t4), free-blood-chemistry (50995)
procainamide-blood-chemistry (50972)

Topic 56

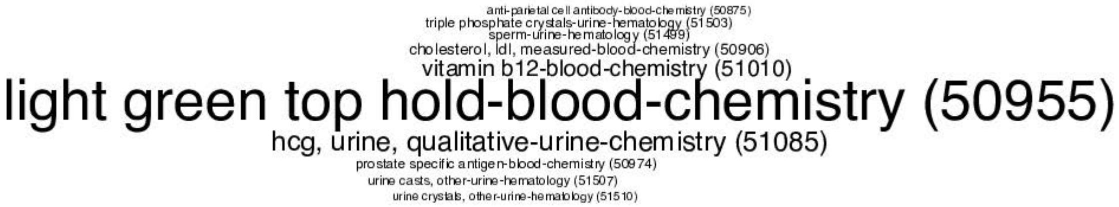

anti-parietal cell antibody-blood-chemistry (50875)
triple phosphate crystals-urine-hematology (51503)
sperm-urine-hematology (51499)
cholesterol, ldl, measured-blood-chemistry (50906)
vitamin b12-blood-chemistry (51010)
light green top hold-blood-chemistry (50955)
hcg, urine, qualitative-urine-chemistry (51085)
prostate specific antigen-blood-chemistry (50974)
urine casts, other-urine-hematology (51507)
urine crystals, other-urine-hematology (51510)

Topic 57

prostate specific antigen-blood-chemistry (50974)
vitamin b12-blood-chemistry (51010)
cholesterol, ldl, measured-blood-chemistry (50906)
absolute hemoglobin-blood-chemistry (50855)
triiodothyronine (t3)-blood-chemistry (51001)
absolute a1c-blood-chemistry (50854)
[a1c]-blood-chemistry (51538)
eosinophil count-blood-hematology (51199)
sperm-urine-hematology (51499)
triple phosphate crystals-urine-hematology (51503)

Topic 58

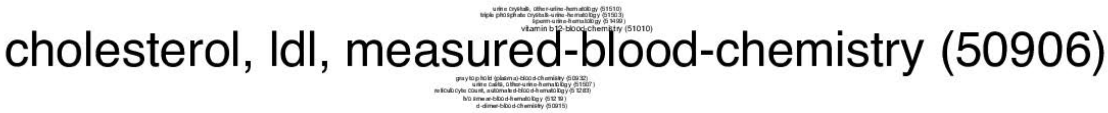
urine crystals, other-urine-hematology (51510)
triple phosphate crystals-urine-hematology (51503)
sperm-urine-hematology (51499)
vitamin b12-blood-chemistry (51010)
cholesterol, ldl, measured-blood-chemistry (50906)
gray top hold (plasma)-blood-chemistry (50932)
urine casts, other-urine-hematology (51507)
reticulocyte count, automated-blood-hematology (51283)
d-dimer-blood-chemistry (50915)

Topic 59

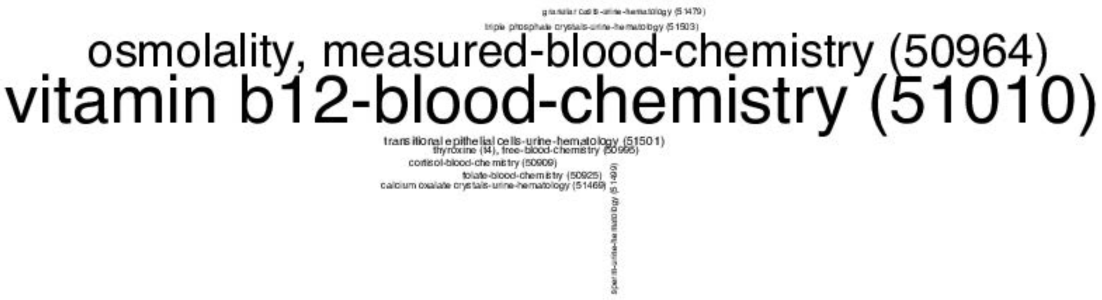
granular casts-urine-hematology (51479)
triple phosphate crystals-urine-hematology (51503)
osmolality, measured-blood-chemistry (50964)
vitamin b12-blood-chemistry (51010)
transitional epithelial cells-urine-hematology (51501)
thyroxine (t4), free-blood-chemistry (50995)
cortisol-blood-chemistry (50909)
folate-blood-chemistry (50925)
calcium oxalate crystals-urine-hematology (51469)

Topic 60

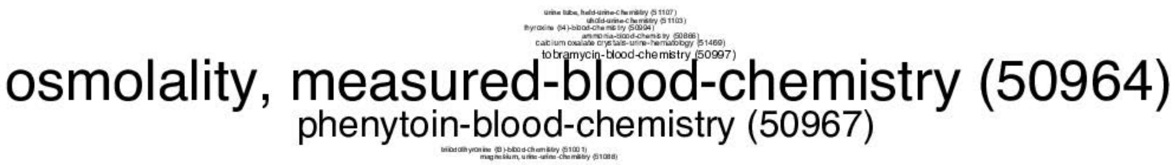
thyroxine (t4)-blood-chemistry (50994)
ammonia-blood-chemistry (50866)
calcium oxalate crystals-urine-hematology (51469)
tobramycin-blood-chemistry (50997)
osmolality, measured-blood-chemistry (50964)
phenytoin-blood-chemistry (50967)

Topic 61

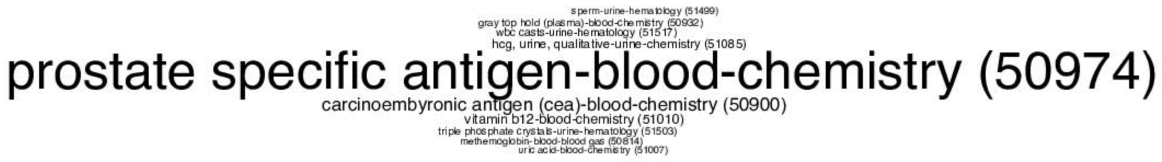
sperm-urine-hematology (51499)
gray top hold (plasma)-blood-chemistry (50932)
wbc casts-urine-hematology (51517)
hcg, urine, qualitative-urine-chemistry (51085)
prostate specific antigen-blood-chemistry (50974)
carcinoembyronic antigen (cea)-blood-chemistry (50900)
vitamin b12-blood-chemistry (51010)
triple phosphate crystals-urine-hematology (51503)
methemoglobin-blood-blood gas (50814)
uric acid-blood-chemistry (51007)

Topic 62

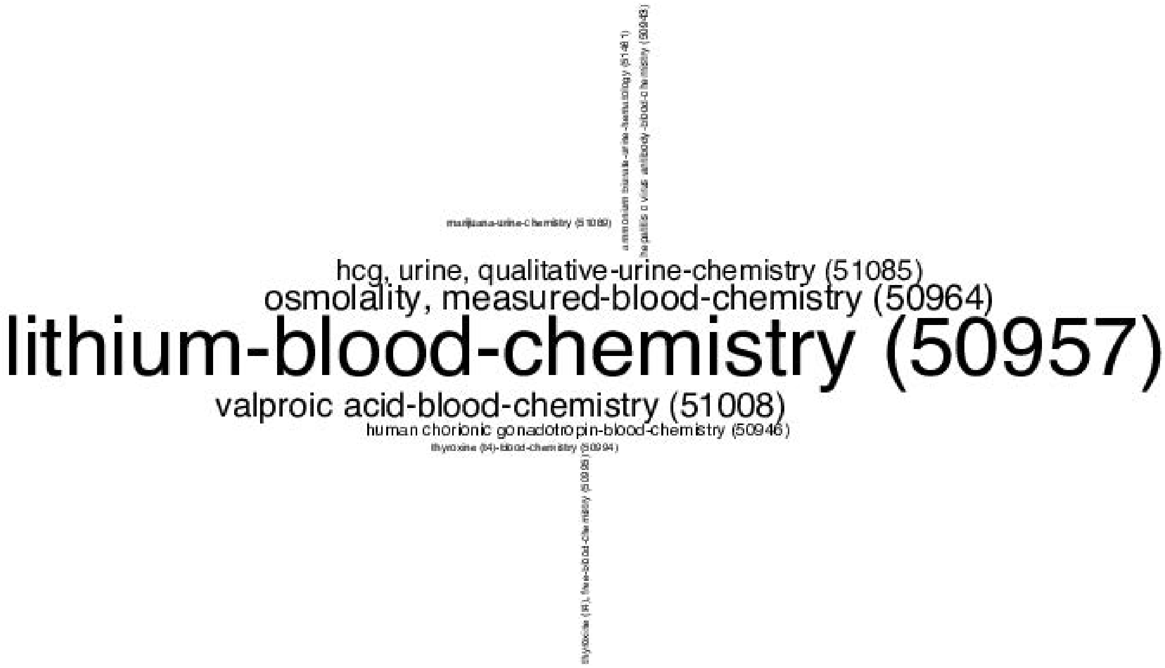
hcg, urine, qualitative-urine-chemistry (51085)
osmolality, measured-blood-chemistry (50964)
lithium-blood-chemistry (50957)
valproic acid-blood-chemistry (51008)
human chorionic gonadotropin-blood-chemistry (50946)

Topic 63

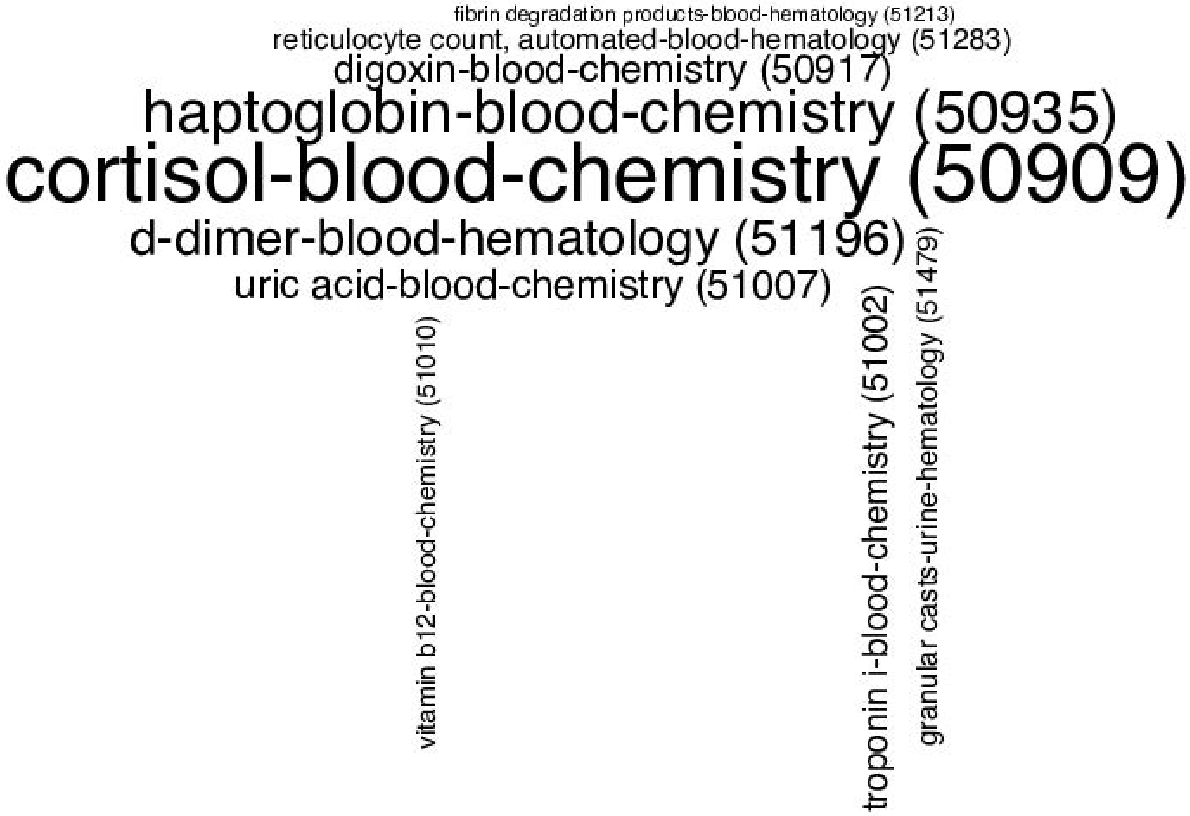
fibrin degradation products-blood-hematology (51213)
reticulocyte count, automated-blood-hematology (51283)
digoxin-blood-chemistry (50917)
haptoglobin-blood-chemistry (50935)
cortisol-blood-chemistry (50909)
d-dimer-blood-hematology (51196)
uric acid-blood-chemistry (51007)
vitamin b12-blood-chemistry (51010)
troponin i-blood-chemistry (51002)
granular casts-urine-hematology (51479)

Topic 64

lupus anticoagulant-blood-hematology (51243)
protein s, functional-blood-hematology (51273)
anticardiolipin antibody igm-blood-hematology (51139)
protein c, functional-blood-hematology (51271)
sedimentation rate-blood-hematology (51288)
osmolality, measured-blood-chemistry (50964)
homocysteine-blood-chemistry (50945)
anticardiolipin antibody igg-blood-hematology (51138)
antithrombin-blood-hematology (51140)
c-reactive protein-blood-chemistry (50889)

Topic 65

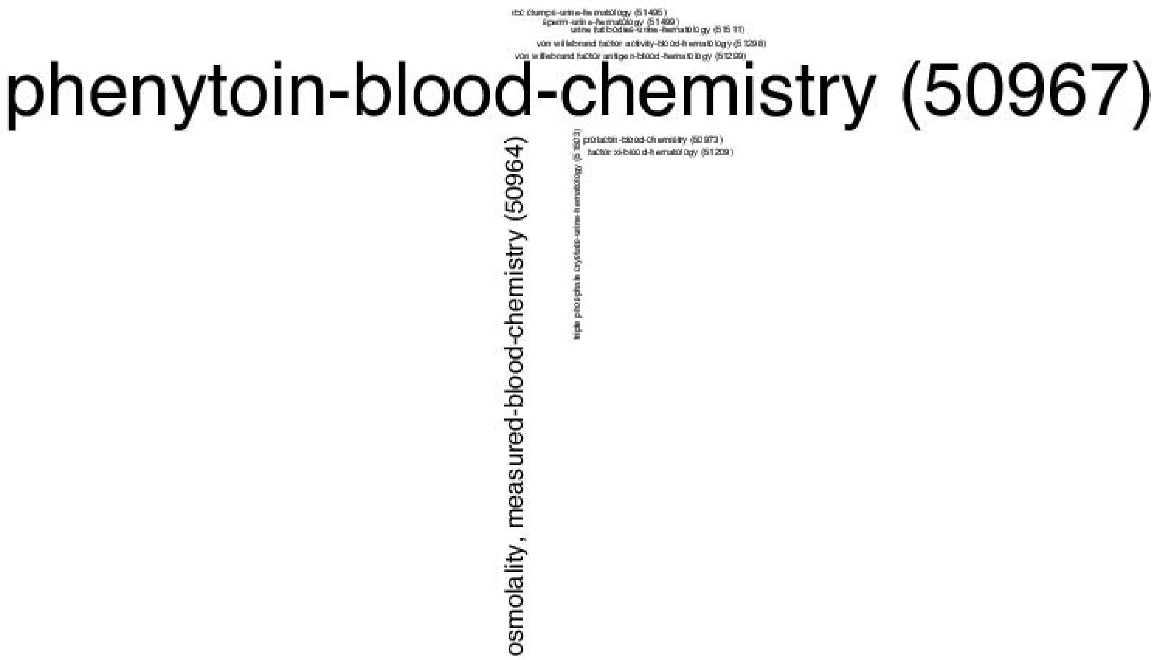

rbc clumps-urine-hematology (51495)
sperm-urine-hematology (51499)
urine fat bodies-urine-hematology (51511)
von willebrand factor activity-blood-hematology (51298)
von willebrand factor antigen-blood-hematology (51299)
phenytoin-blood-chemistry (50967)
prolactin-blood-chemistry (50973)
factor xi-blood-hematology (51209)
triple phosphate crystals-urine-hematology (51503)
osmolality, measured-blood-chemistry (50964)

Topic 66

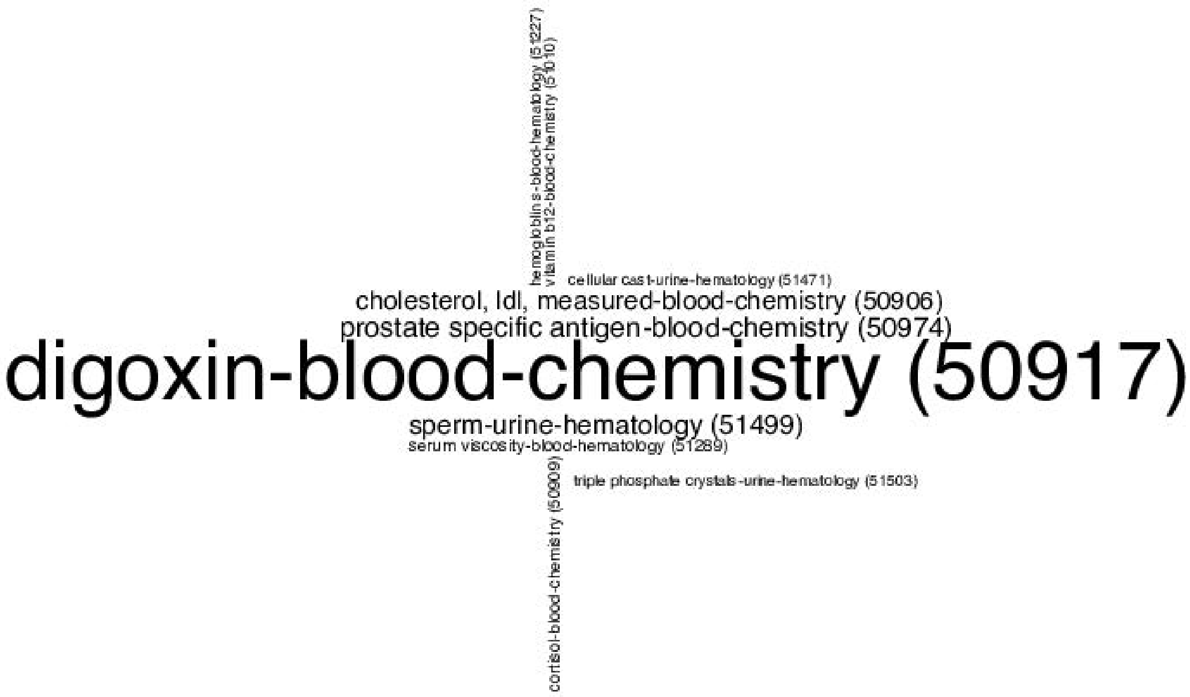
hemoglobins-blood-hematology (51227)
vitamin b12-blood-chemistry (51010)
cellular cast-urine-hematology (51471)
cholesterol, ldl, measured-blood-chemistry (50906)
prostate specific antigen-blood-chemistry (50974)
digoxin-blood-chemistry (50917)
sperm-urine-hematology (51499)
serum viscosity-blood-hematology (51289)
triple phosphate crystals-urine-hematology (51503)
cortisol-blood-chemistry (50909)

Topic 67

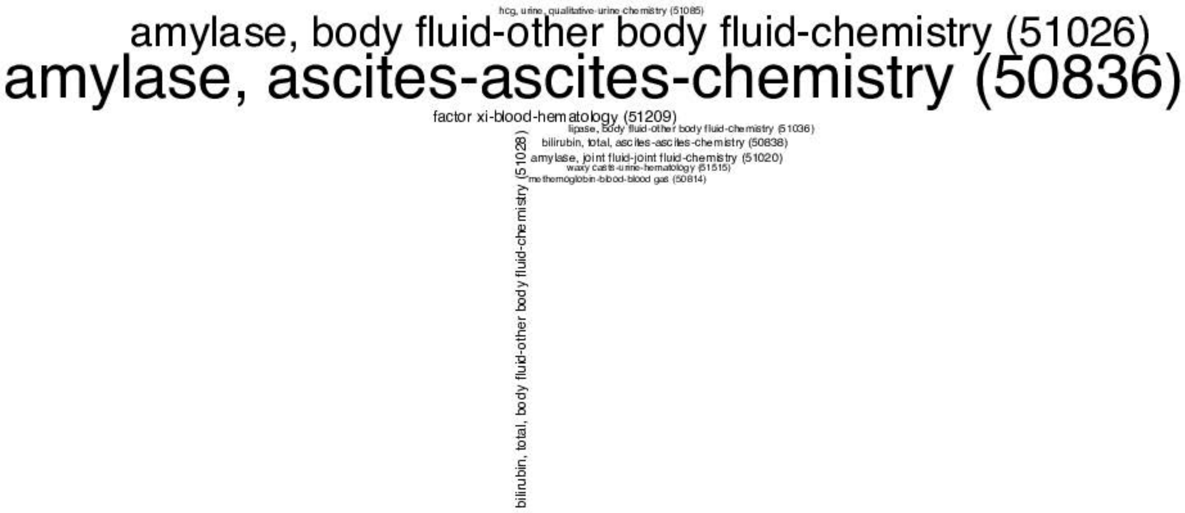
hcg, urine, qualitative-urine-chemistry (51085)
amylase, body fluid-other body fluid-chemistry (51026)
amylase, ascites-ascites-chemistry (50836)
factor xi-blood-hematology (51209)
lipase, body fluid-other body fluid-chemistry (51036)
bilirubin, total, ascites-ascites-chemistry (50838)
amylase, joint fluid-joint fluid-chemistry (51020)
waxy casts-urine-hematology (51515)
methemoglobin-blood-blood gas (50814)
bilirubin, total, body fluid-other body fluid-chemistry (51028)

Topic 68

urine crystals, other-urine-hematology (51510)
quantitative g6pd-blood-hematology (51276)
lambda-other body fluid-hematology (51426)
immunophenotyping-other body fluid-hematology (51424)
cd20-other body fluid-hematology (51400)
triple phosphate crystals-urine-hematology (51503)
porphobilinogen screen-urine-chemistry (51096)
immunoglobulin a-blood-chemistry (50949)
cd45-other body fluid-hematology (51411)
kappa-other body fluid-hematology (51425)

Topic 69

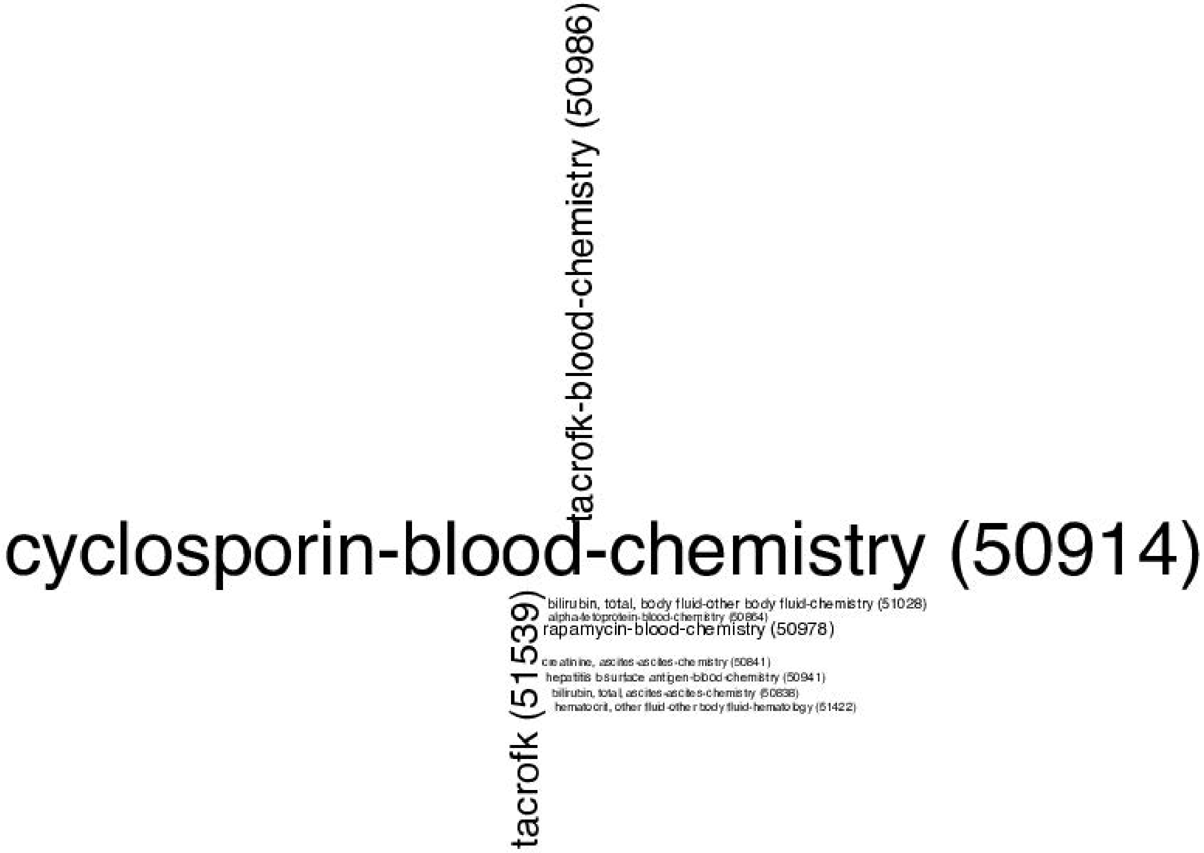

cyclosporin-blood-chemistry (50914)
tacrofk-blood-chemistry (50986)
tacrofk (51539)
bilirubin, total, body fluid-other body fluid-chemistry (51028)
alpha-fetoprotein-blood-chemistry (50864)
rapamycin-blood-chemistry (50978)
creatinine, ascites-ascites-chemistry (50841)
hepatitis b surface antigen-blood-chemistry (50941)
bilirubin, total, ascites-ascites-chemistry (50838)
hematocrit, other fluid-other body fluid-hematology (51422)

Topic 70

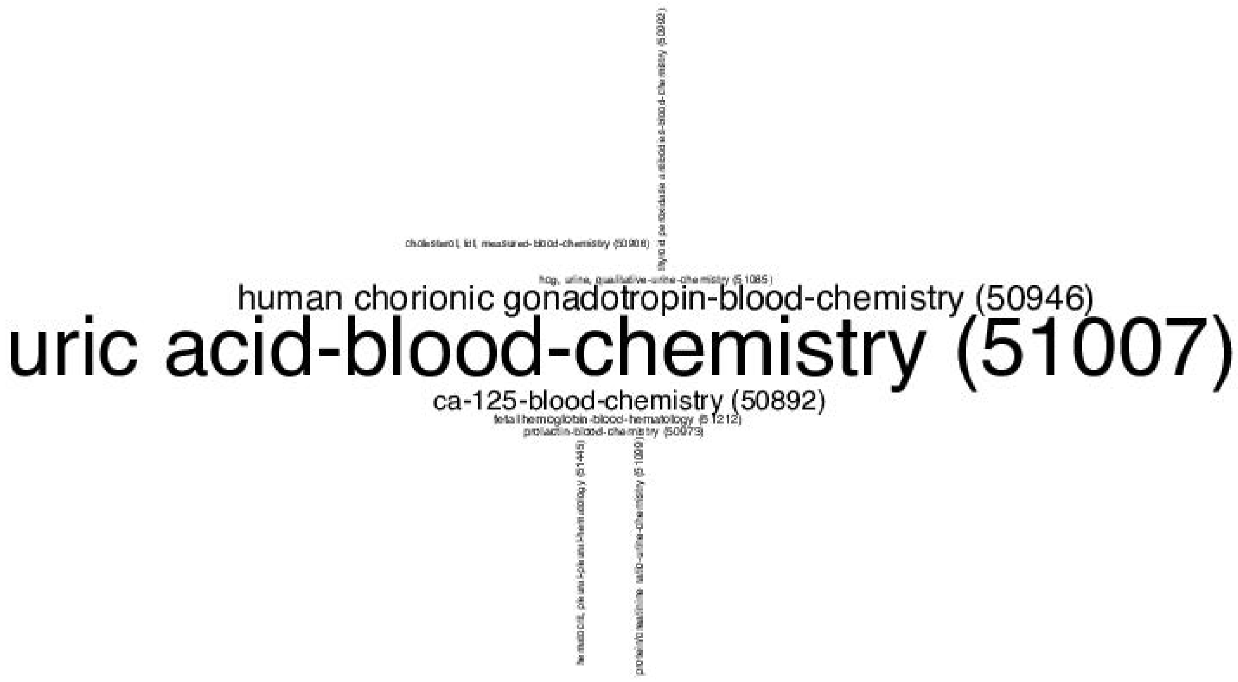

cholesterol, ldl, measured-blood-chemistry (50906)
hcg, urine, qualitative-urine-chemistry (51085)
human chorionic gonadotropin-blood-chemistry (50946)
uric acid-blood-chemistry (51007)
ca-125-blood-chemistry (50892)
fetal hemoglobin-blood-hematology (51212)
prolactin-blood-chemistry (50973)

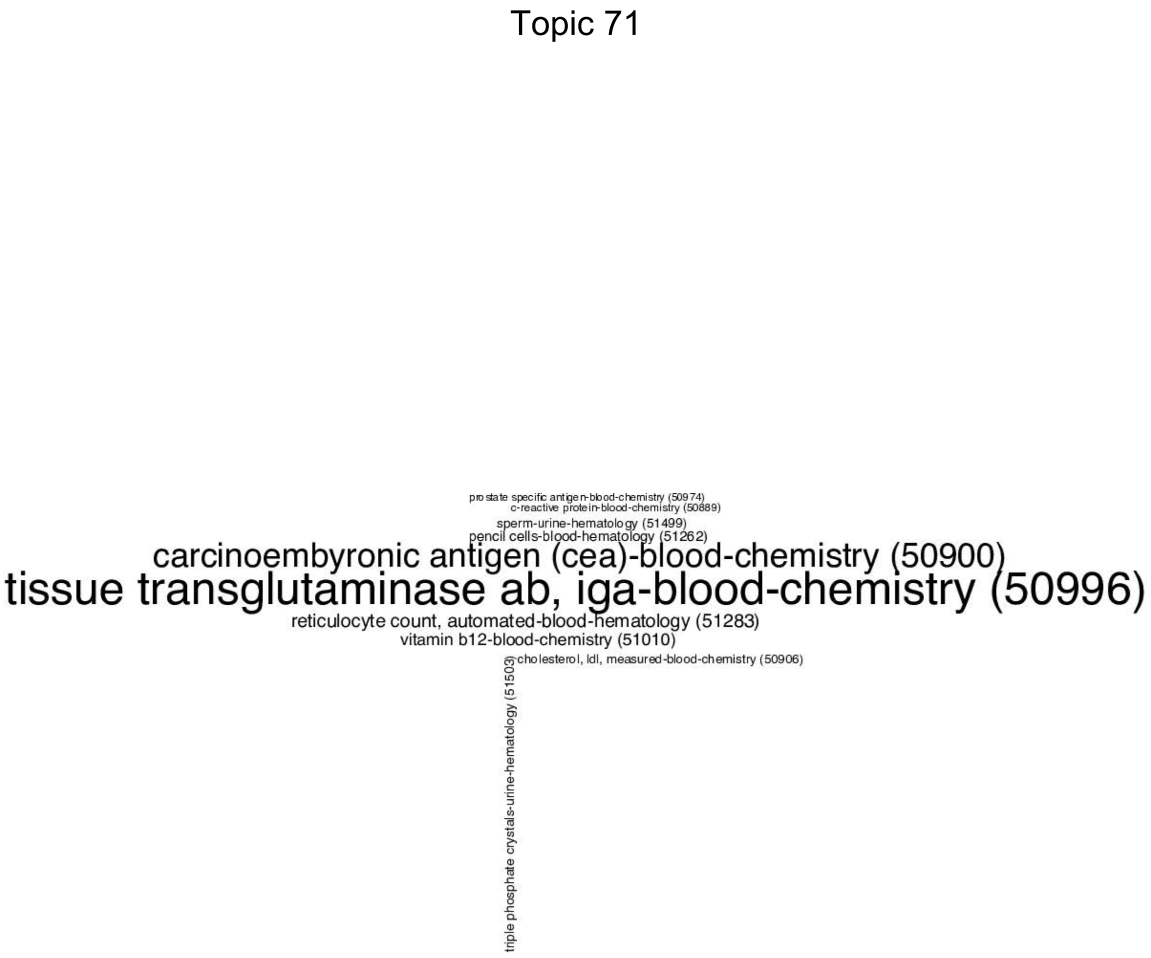
Topic 71
prostate specific antigen-blood-chemistry (50974)
c-reactive protein-blood-chemistry (50889)
sperm-urine-hematology (51499)
pencil cells-blood-hematology (51262)
carcinoembyronic antigen (cea)-blood-chemistry (50900)
tissue transglutaminase ab, iga-blood-chemistry (50996)
reticulocyte count, automated-blood-hematology (51283)
vitamin b12-blood-chemistry (51010)
cholesterol, ldl, measured-blood-chemistry (50906)
triple phosphate crystals-urine-hematology (51503)

Topic 72

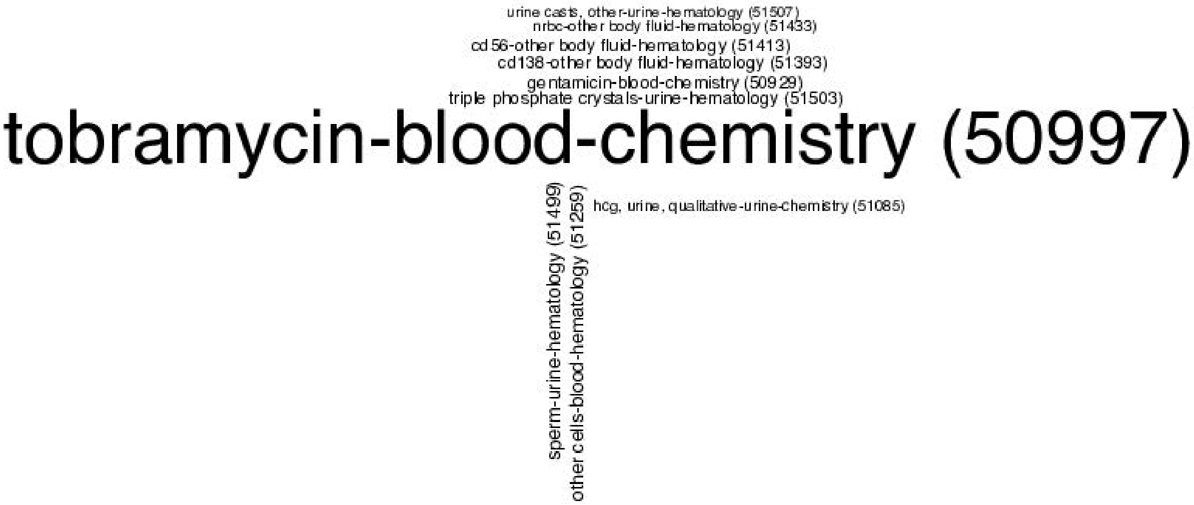

urine casts, other-urine-hematology (51507)
nrbc-other body fluid-hematology (51433)
cd56-other body fluid-hematology (51413)
cd138-other body fluid-hematology (51393)
gentamicin-blood-chemistry (50929)
triple phosphate crystals-urine-hematology (51503)
tobramycin-blood-chemistry (50997)
sperm-urine-hematology (51499)
other cells-blood-hematology (51259)
hcg, urine, qualitative-urine-chemistry (51085)

Topic 73

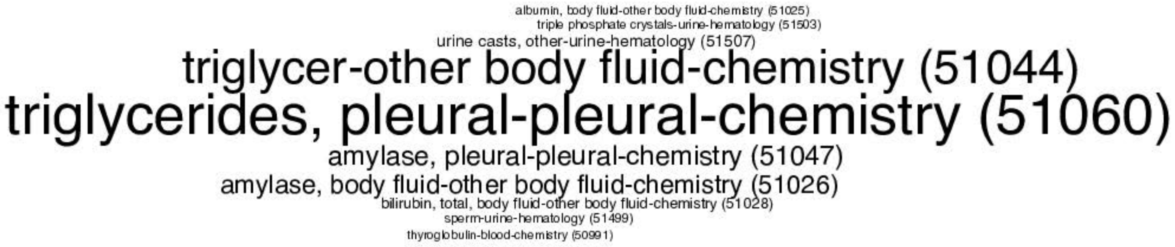
albumin, body fluid-other body fluid-chemistry (51025)
triple phosphate crystals-urine-hematology (51503)
urine casts, other-urine-hematology (51507)
triglycer-other body fluid-chemistry (51044)
triglycerides, pleural-pleural-chemistry (51060)
amylase, pleural-pleural-chemistry (51047)
amylase, body fluid-other body fluid-chemistry (51026)
bilirubin, total, body fluid-other body fluid-chemistry (51028)
sperm-urine-hematology (51499)
thyroglobulin-blood-chemistry (50991)

Topic 74

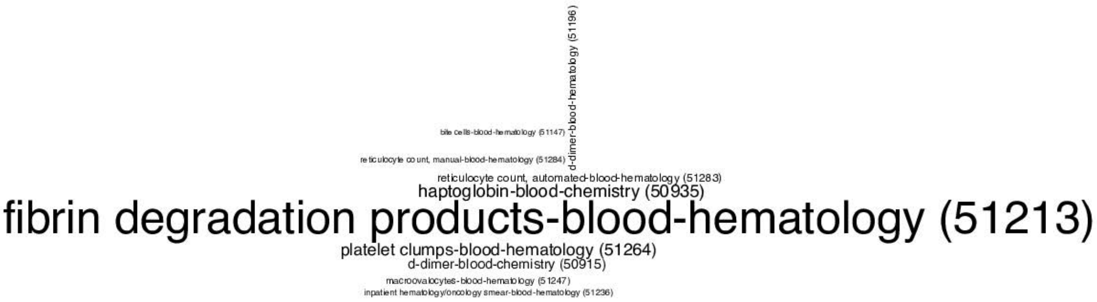
d-dimer-blood-hematology (51196)
bite cells-blood-hematology (51147)
reticulocyte count, manual-blood-hematology (51284)
reticulocyte count, automated-blood-hematology (51283)
haptoglobin-blood-chemistry (50935)
fibrin degradation products-blood-hematology (51213)
platelet clumps-blood-hematology (51264)
d-dimer-blood-chemistry (50915)
macroovalocytes-blood-hematology (51247)
inpatient hematology/oncology smear-blood-hematology (51236)

Topic 75

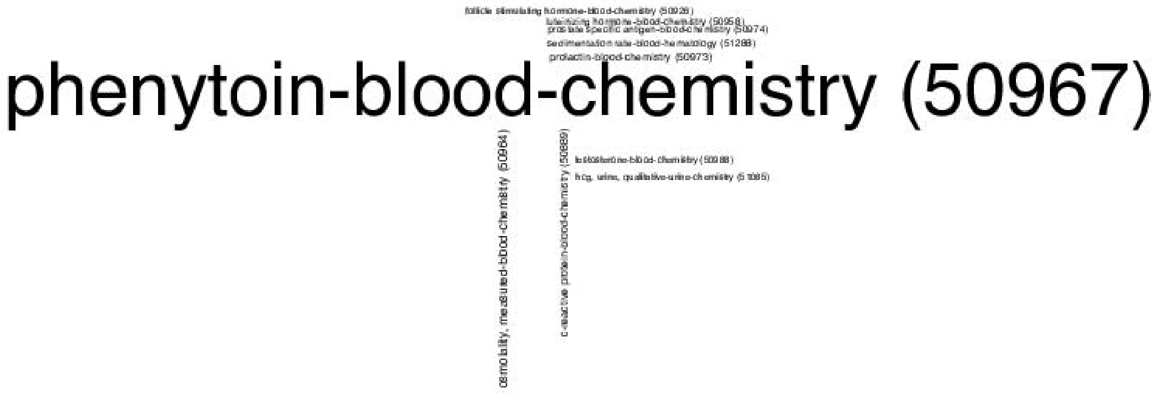
phenytoin-blood-chemistry (50967)
follicle stimulating hormone-blood-chemistry (50926)
luteinizing hormone-blood-chemistry (50958)
prostate specific antigen-blood-chemistry (50974)
sedimentation rate-blood-hematology (51288)
prolactin-blood-chemistry (50973)
osmolality, measured-blood-chemistry (50964)
c-reactive protein-blood-chemistry (50889)
hcg, urine, qualitative-urine-chemistry (51085)

Figure 3: Word clouds of the 75 disease topics learned from the MIMIC-III dataset. The size of the word is proportional to the topic probabilities and colors indicate different data categories. The 75 non-lab topics were plotted first followed by the 75 lab topics.

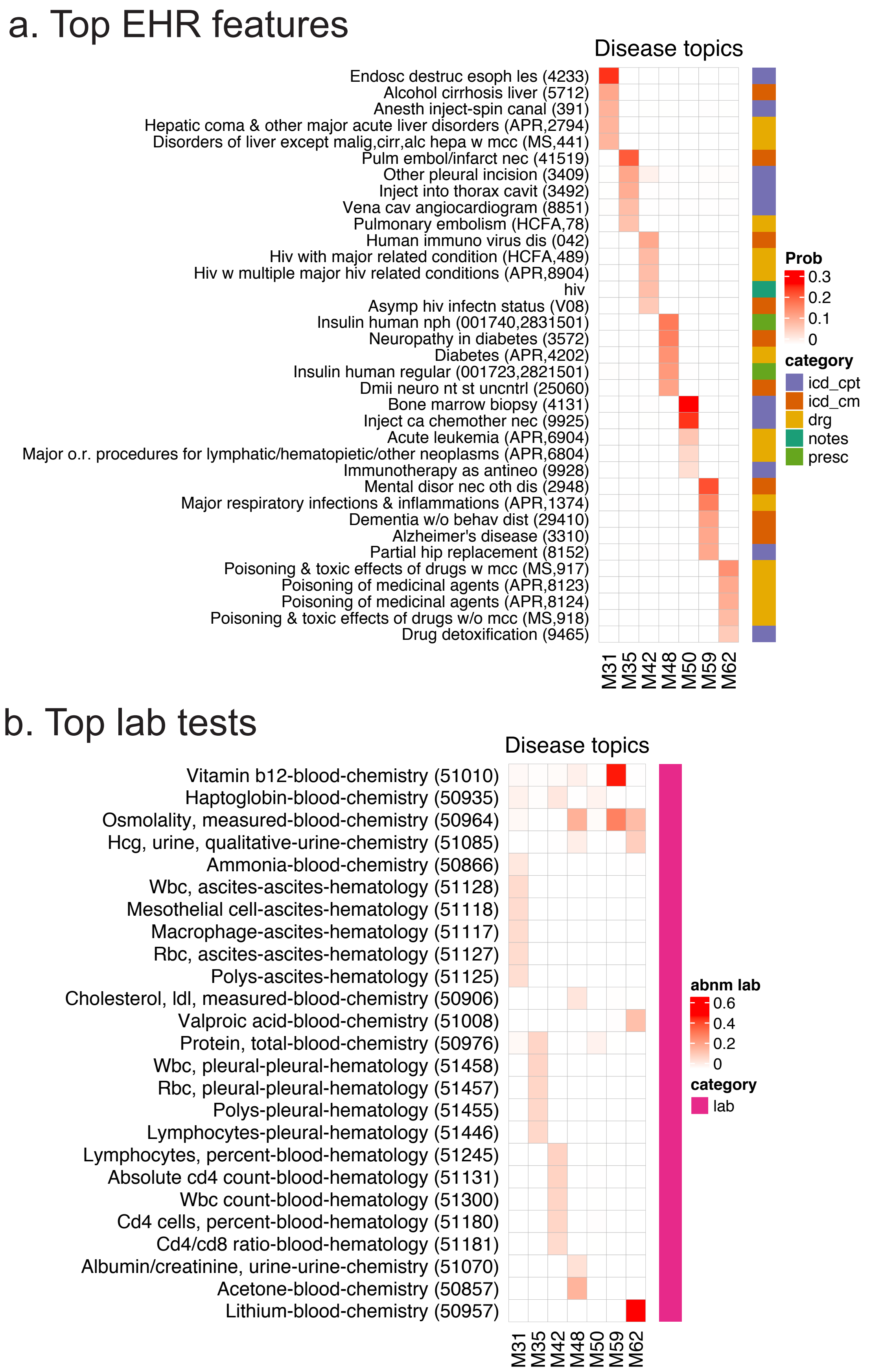

Figure 4: Top five EHR features and lab tests from the MIMIC-III dataset for select topics from a 75-topic MixEHR model.

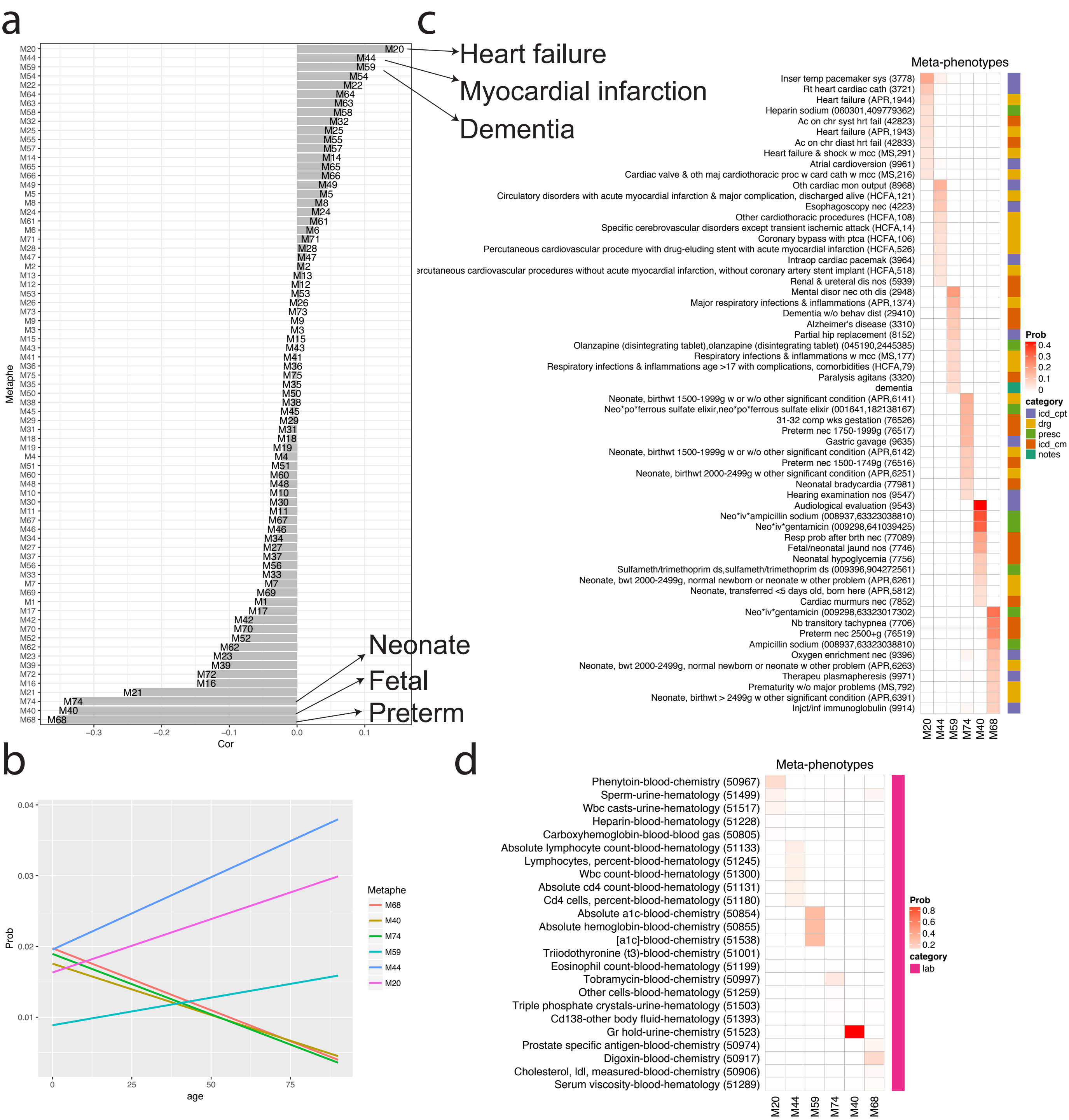

Figure 5: Age correlated topics from MIMIC-III dataset. **a**. Correlation between age and each meta-phenotype. **b**. Specific patient's topic scores correlate with age. The plot display the probabilities of the patient mixtures as a function of age for top 3 most positively age-correlated topics (M20, M44, M59) and 3 most negatively age-correlated topics (M74, M40, M68). **c**. Top 10 clinical features of the 3 most and least age-correlated topics; **d**. Top 5 lab results of the same age-correlated topics.

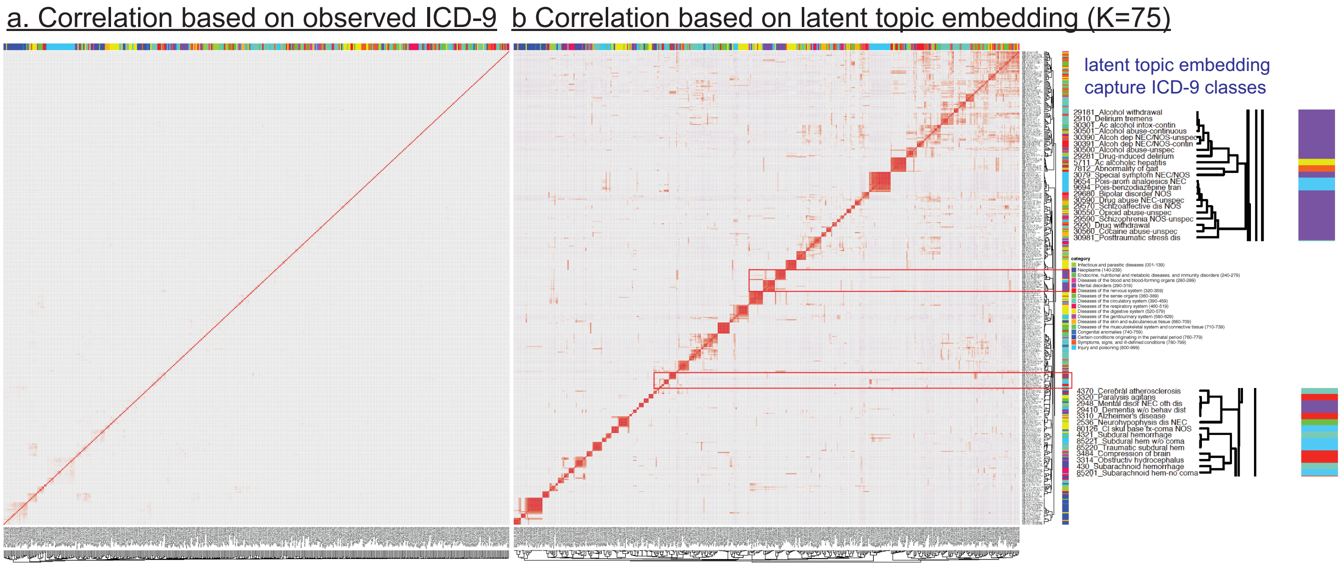

Figure 6: MixEHR reveals disease modules not explicit in the raw EHR data using the MIMIC-III dataset. **a**. Correlation of ICD-9 codes over observed data. **b**. Correlation of ICD-9 codes over latent embeddings learned from MixEHR. The color bars on the top and right of the dendrogram indicate the ICD-9 categories.

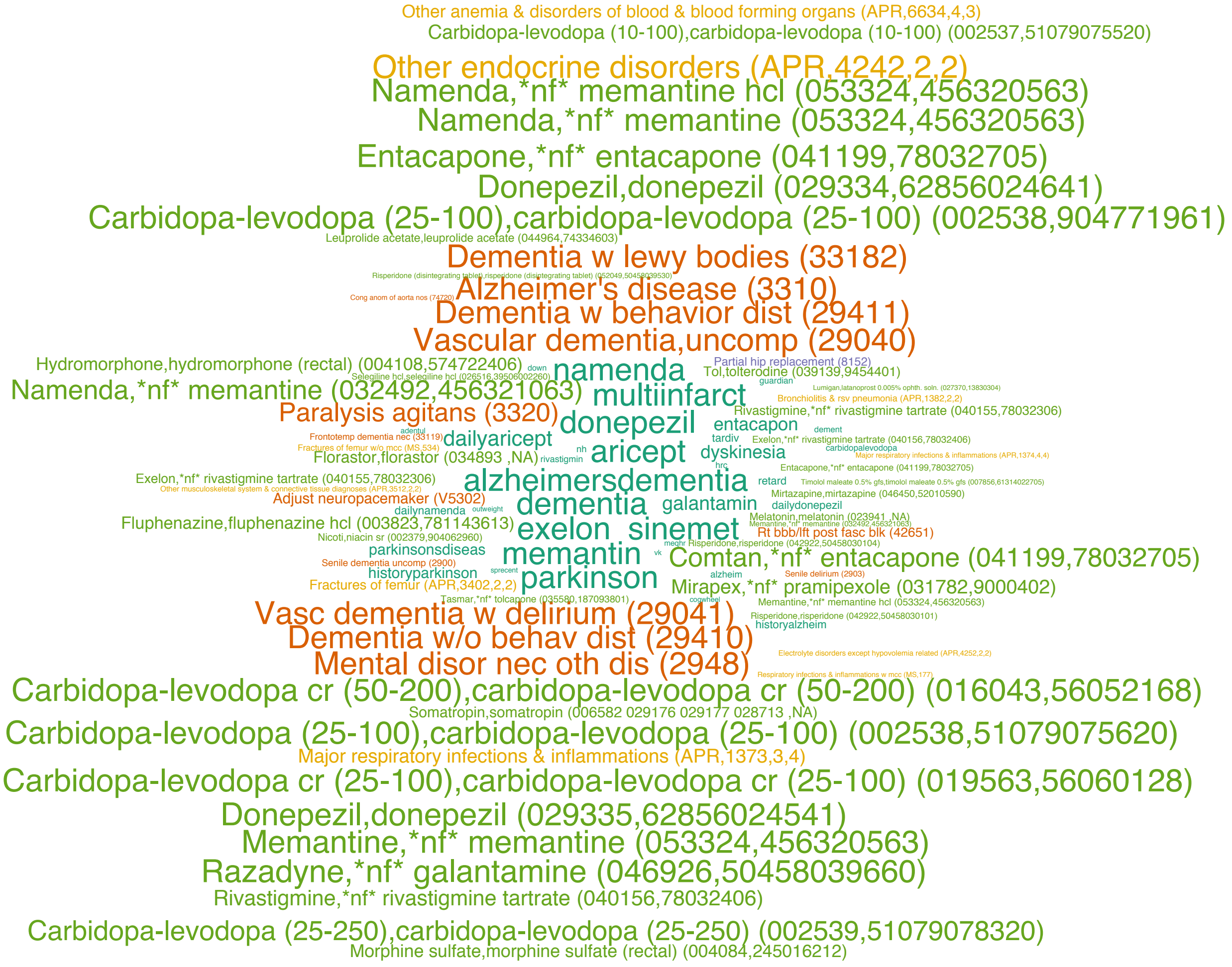

Figure 7: Word cloud of top 100 phenotypes correlated with Alzheimer's disease from the MIMIC-III dataset.

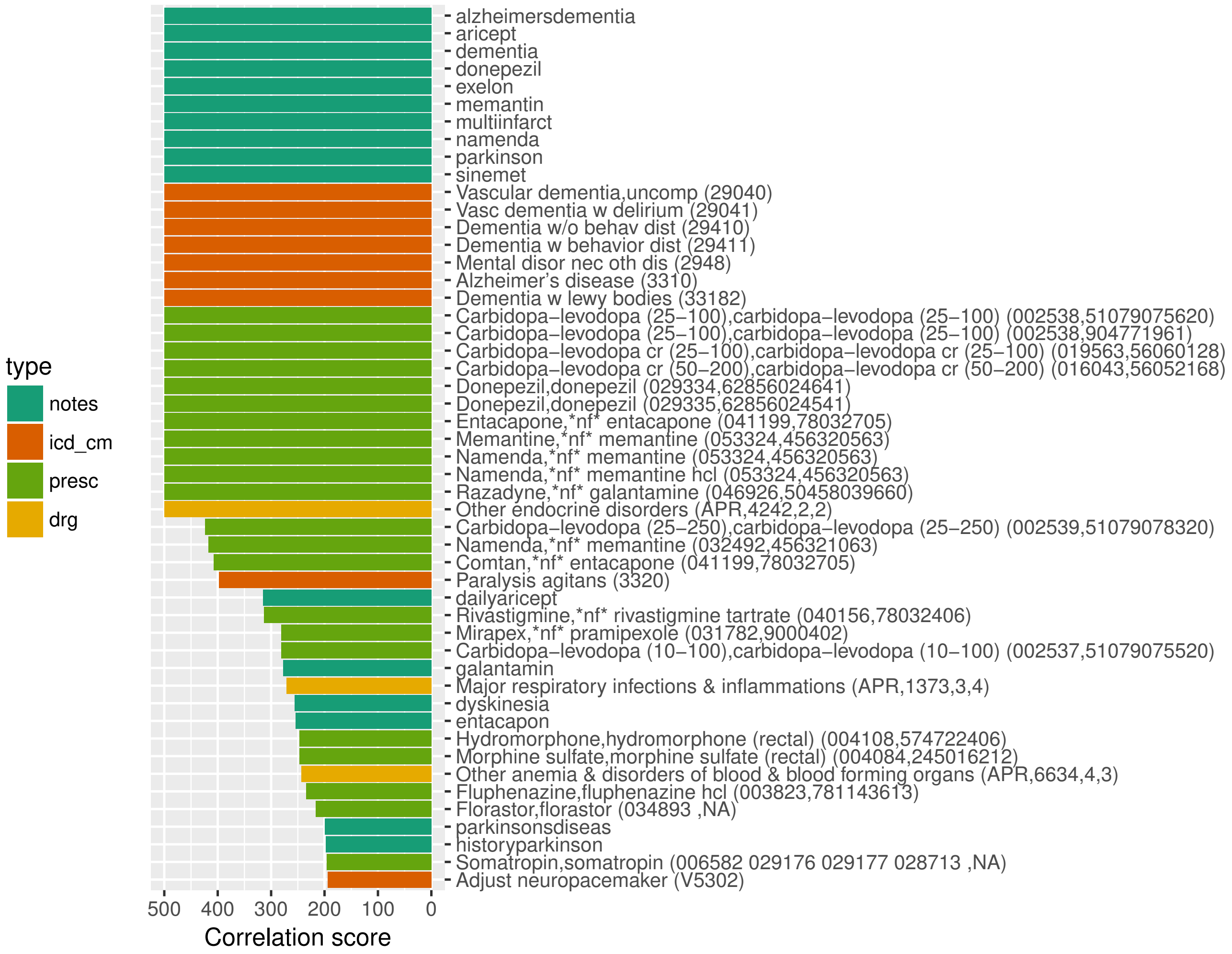

Figure 8: Top 50 phenotypes correlated with Alzheimer's disease from the MIMIC-III dataset.

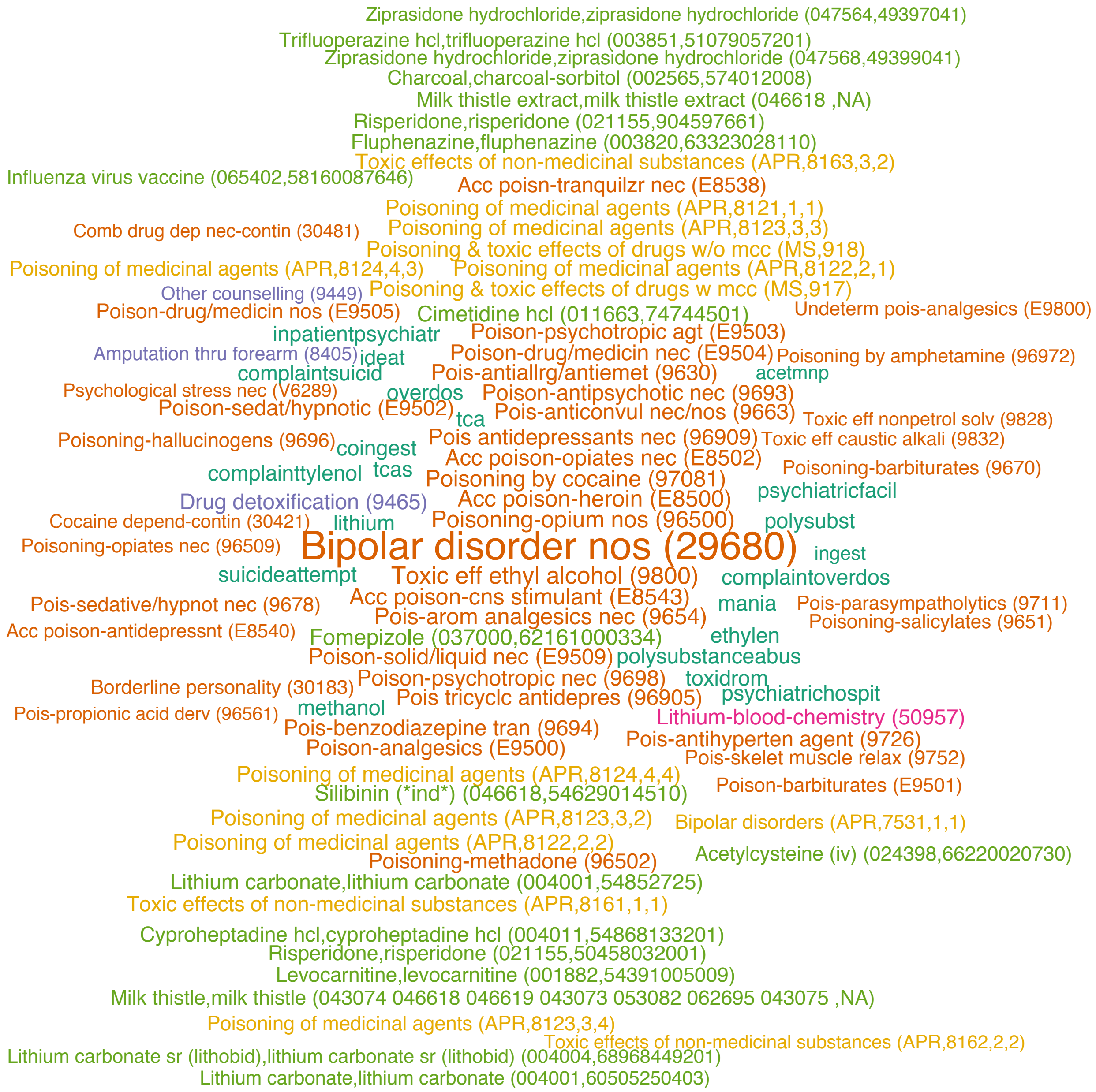

Figure 9: Word cloud of top 100 phenotypes correlated Bipolar Disorder from the MIMIC-III dataset.

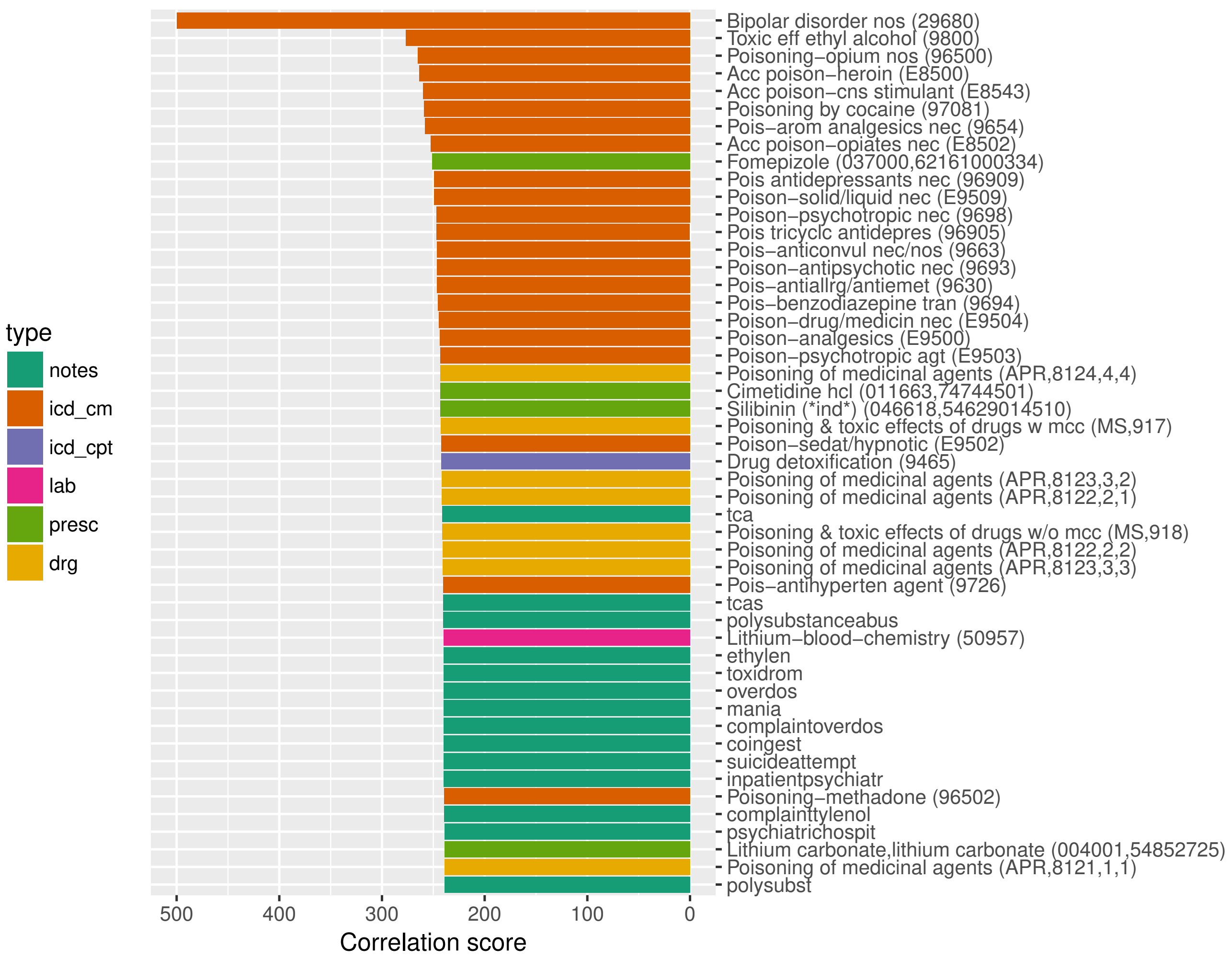

Figure 10: Top 50 phenotypes correlated with Bipolar Disorder from the MIMIC-III dataset.

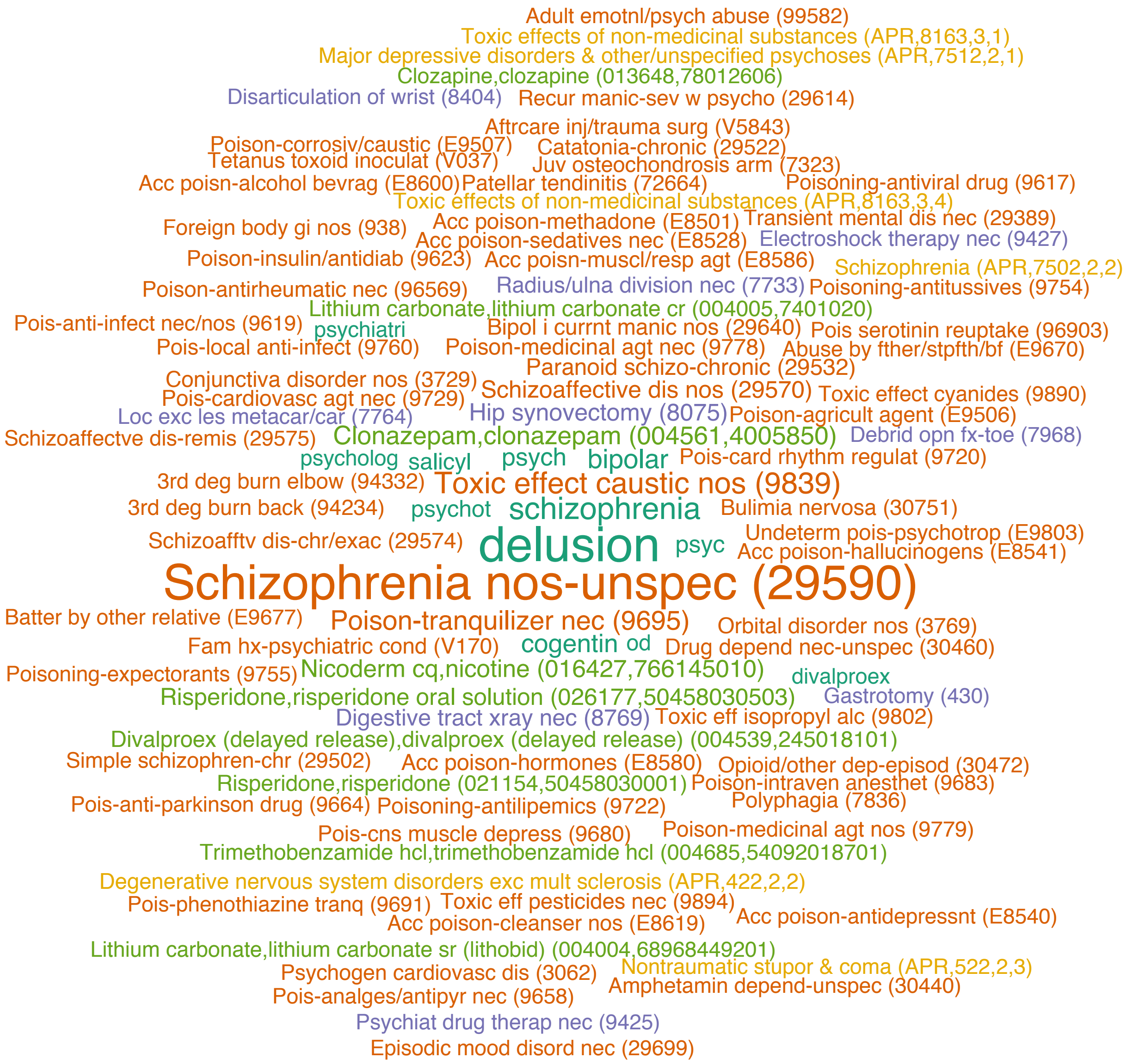

Figure 11: Word cloud of top 100 phenotypes correlated with Schizophrenia from the MIMIC-III dataset.

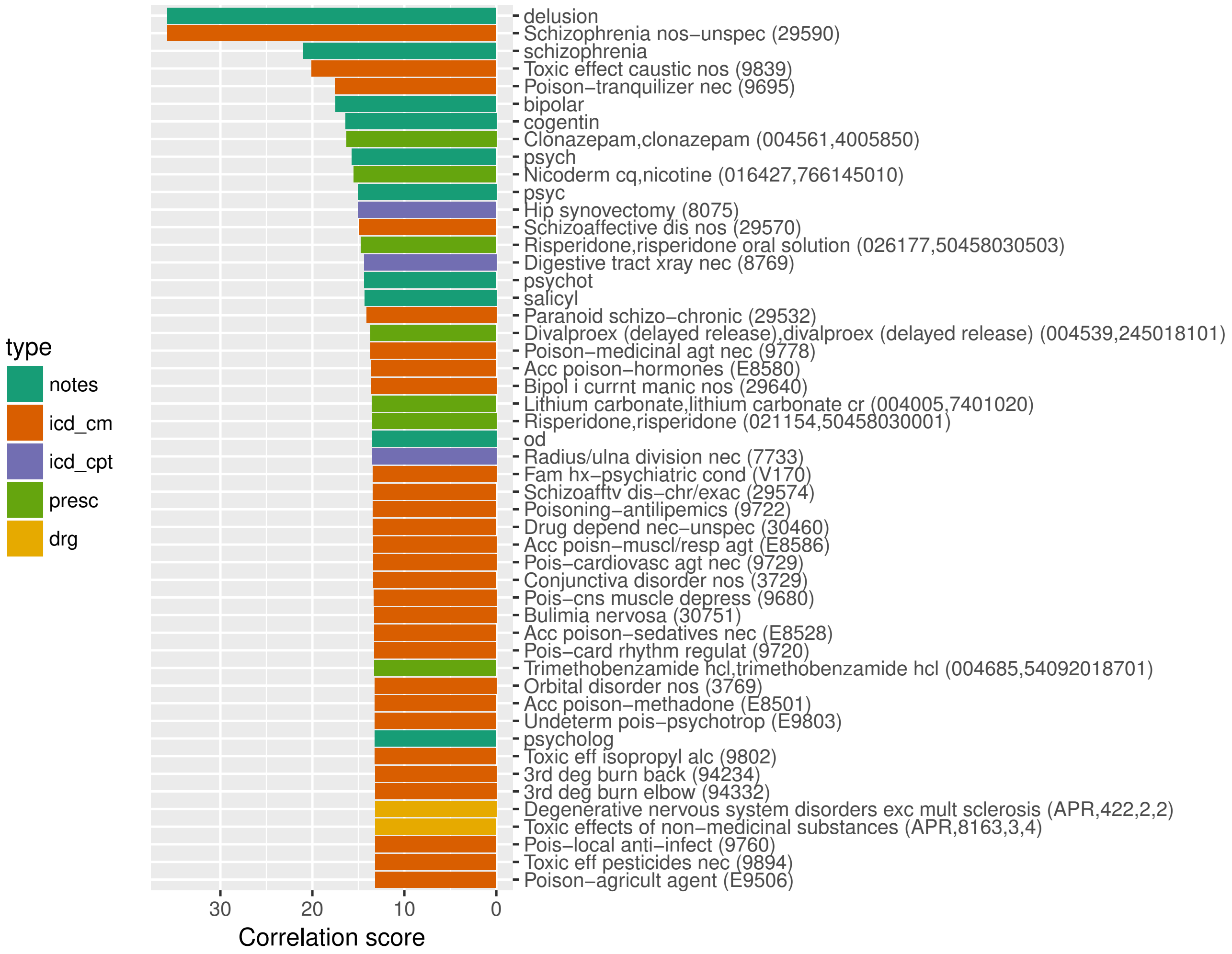

Figure 12: Top 50 phenotypes correlated with Schizophrenia from the MIMIC-III dataset.

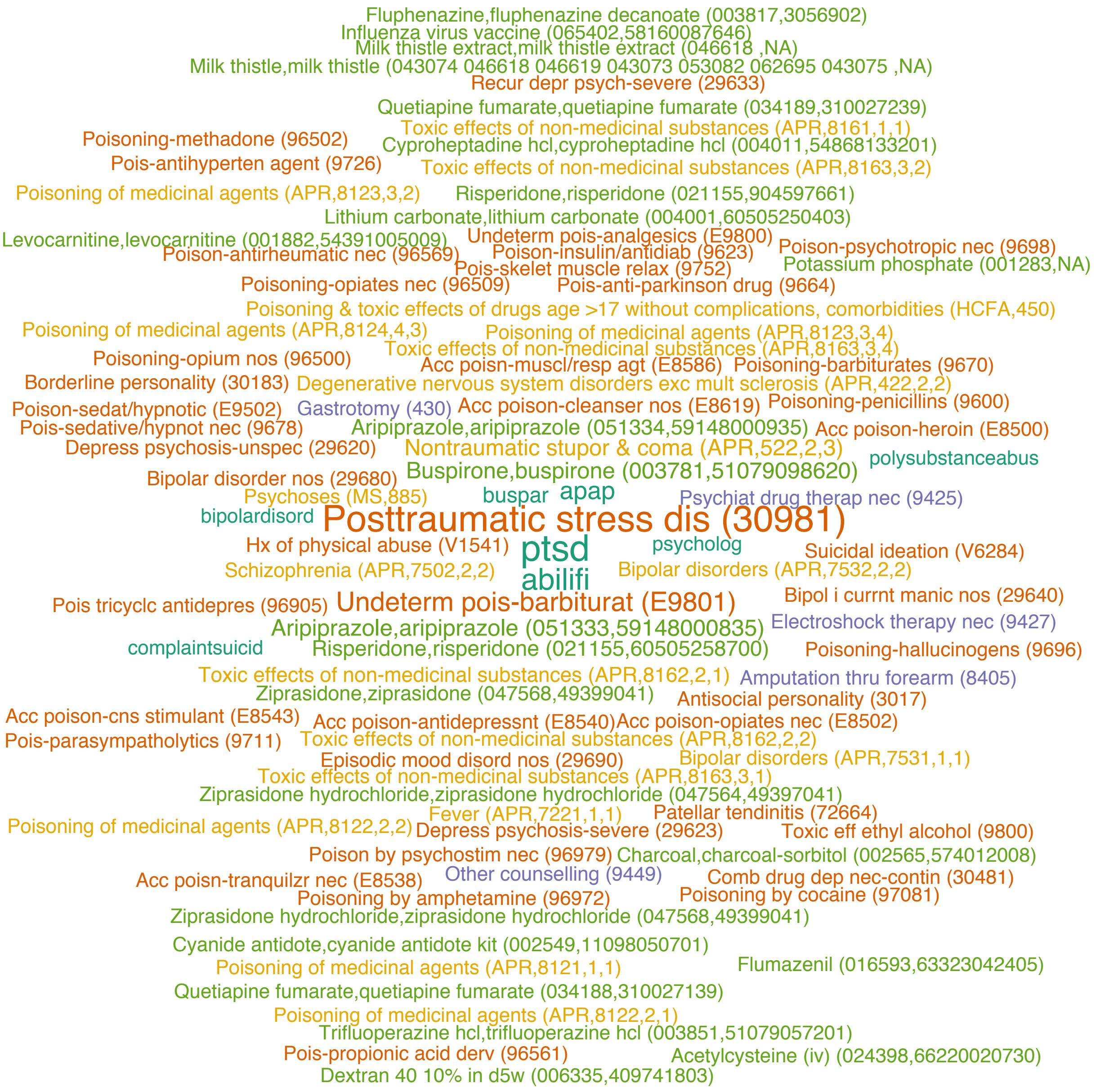

Figure 13: Top 100 phenotypes correlated with PTSD from the MIMIC-III dataset.

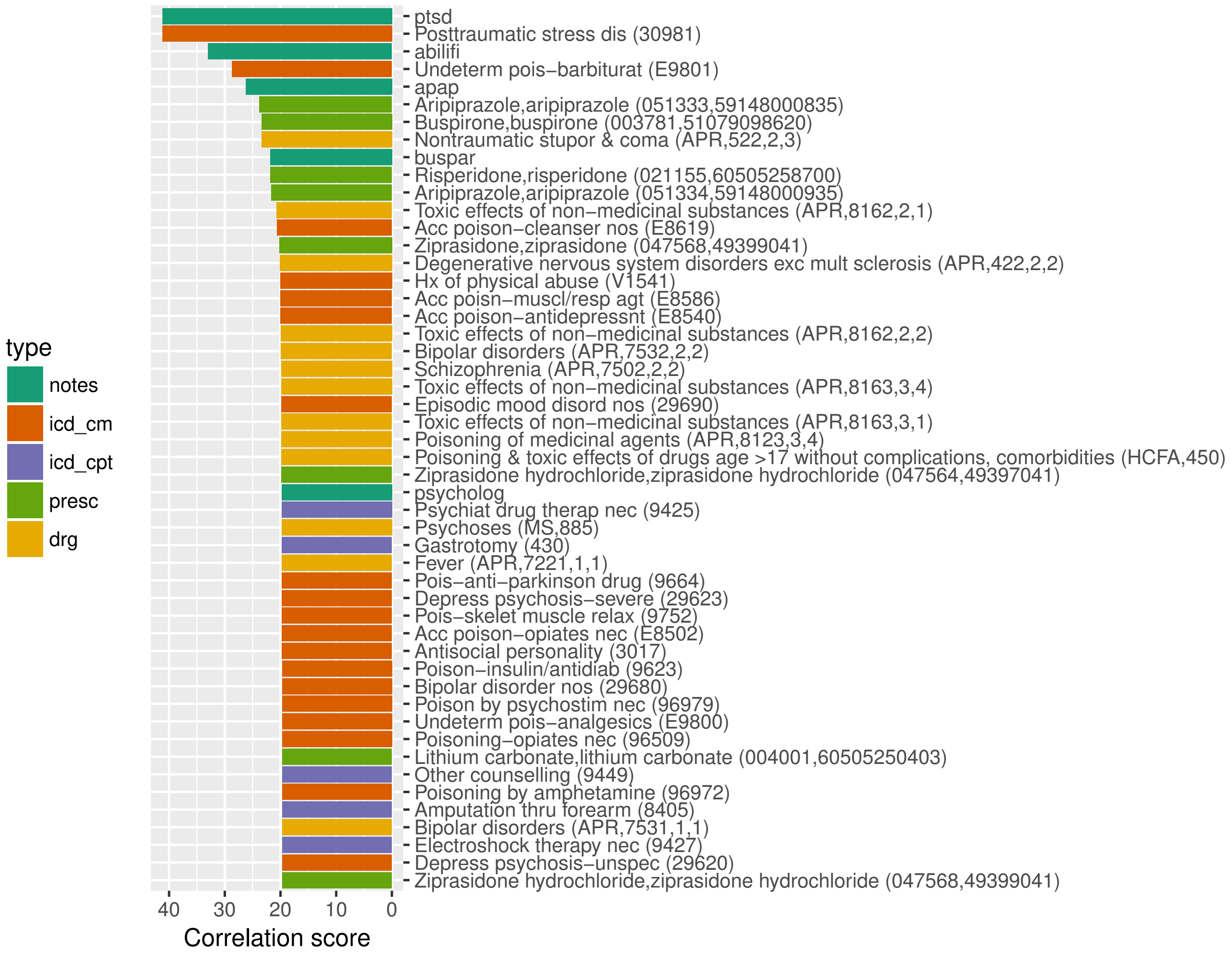

Figure 14: Top 50 phenotypes correlated with with PTSD from the MIMIC-III dataset.

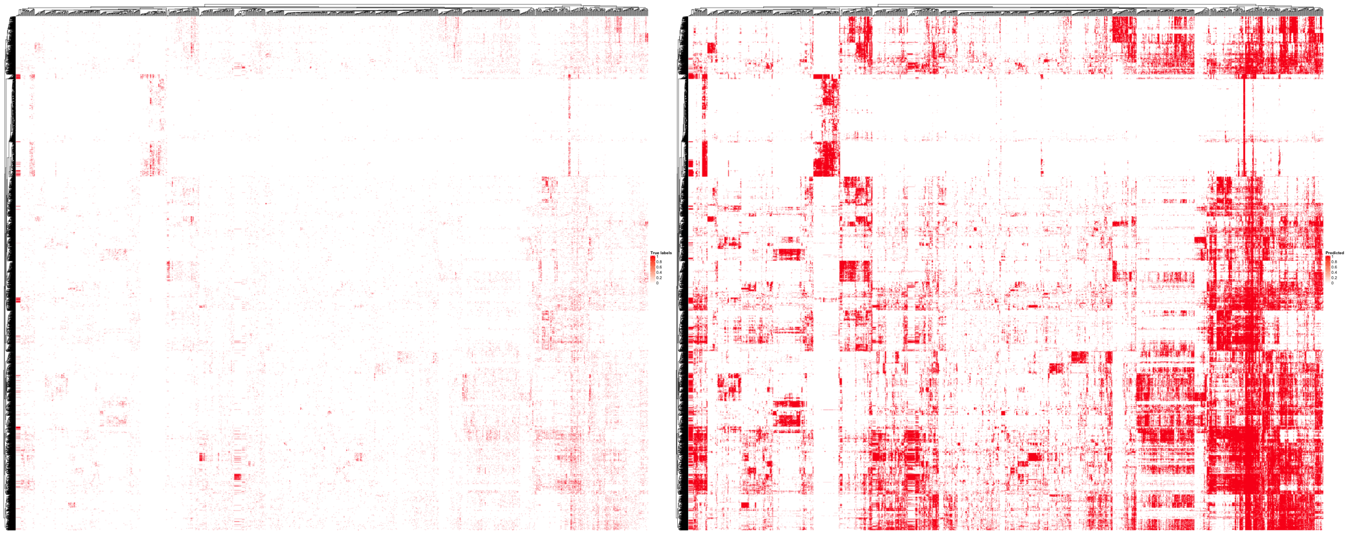

Figure 15: Observed (left) and predicted (right) EHR code from the MIMIC-III dataset

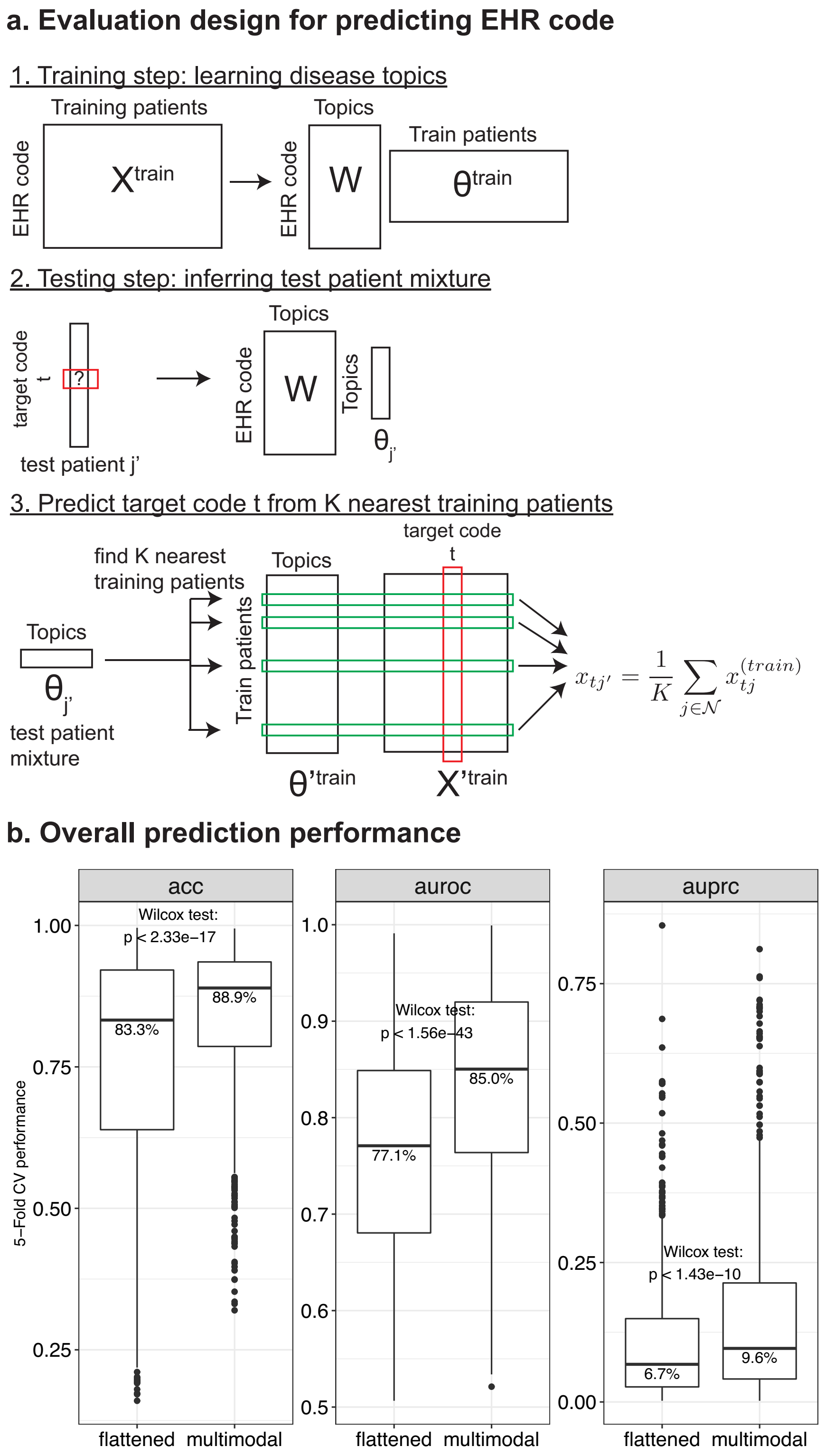

Figure 16: Predicting EHR codes from the MIMIC-III dataset. **a**. Evaluation design for predicting EHR code. **b**. Overall prediction performance. We evaluated the prediction accuracy comparing our proposed multimodal method (i.e., MixEHR) with the baseline unimodal method LDA in terms of accuracy (acc), area under the ROC curve (auroc), and area under of the precision-recall curve (auprc) for 947 codes. The median performance values are indicated below the median line of each boxplots, and the Wilcox test p-values comparing the distributions of the performance values between MixEHR and the baseline are also indicated on the plots.

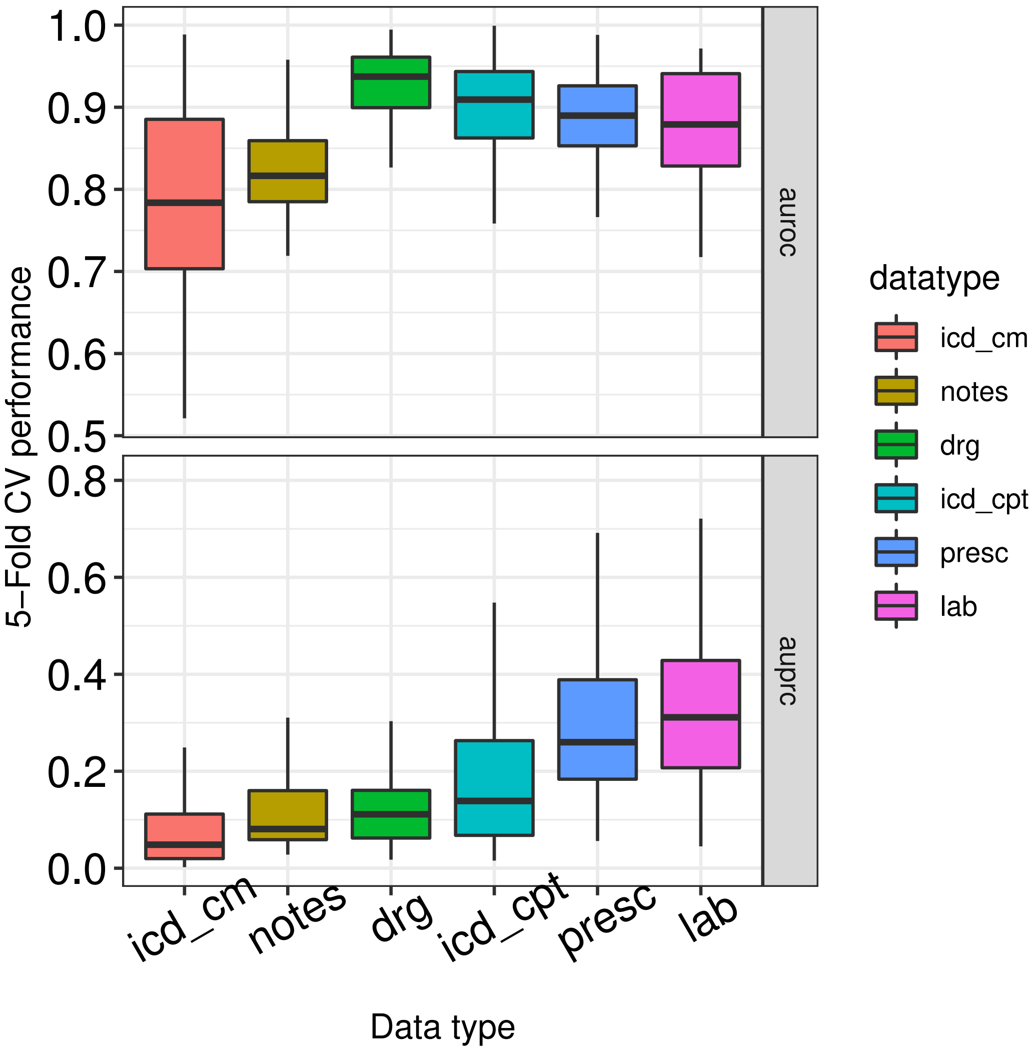

Figure 17: Prediction separated by different data types from the MIMIC-III dataset.

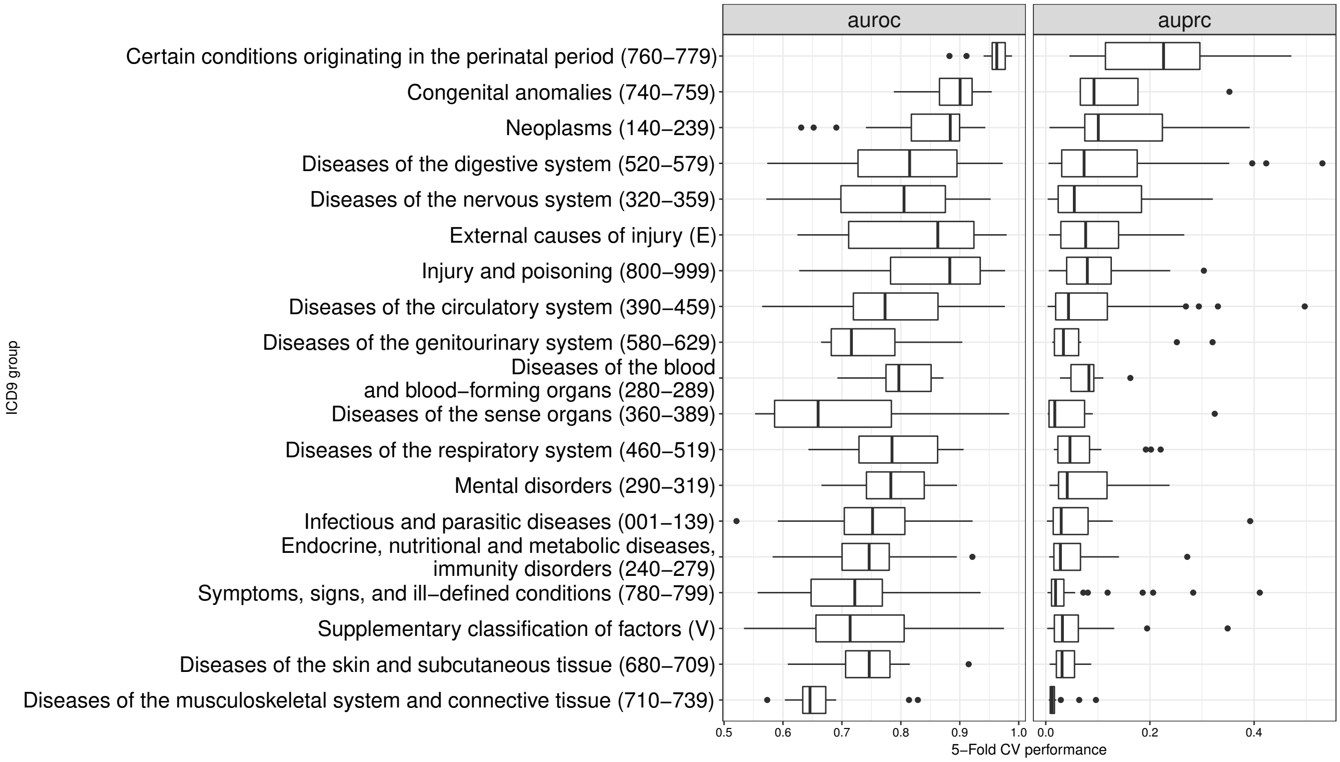

Figure 18: ICD-9 prediction accuracy separated by different ICD-9 groups using the MIMIC-III dataset.

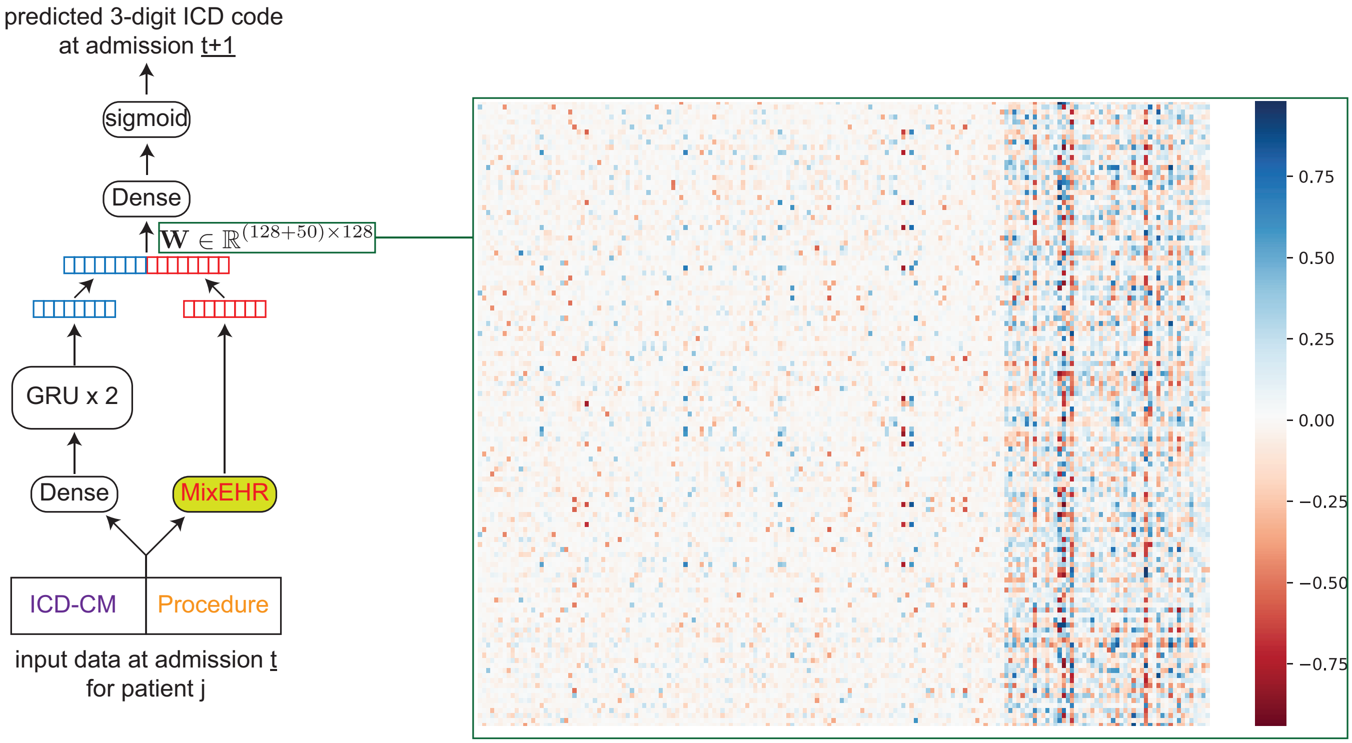

Figure 19: Hinton plot displaying the RNN network weights learned from the Quebec CHD dataset. The connection between the last 128-dense layer and the the previous 178-layer, which concatenates 50-hidden dense layer embedding and the MixEHR 50-topic mixture embedding, are displayed in the heatmap.The connection weights were automatically learned by the neural network. Therefore, the Hinton plot reflects the importance of each embedding features to the RNN in making the prediction.

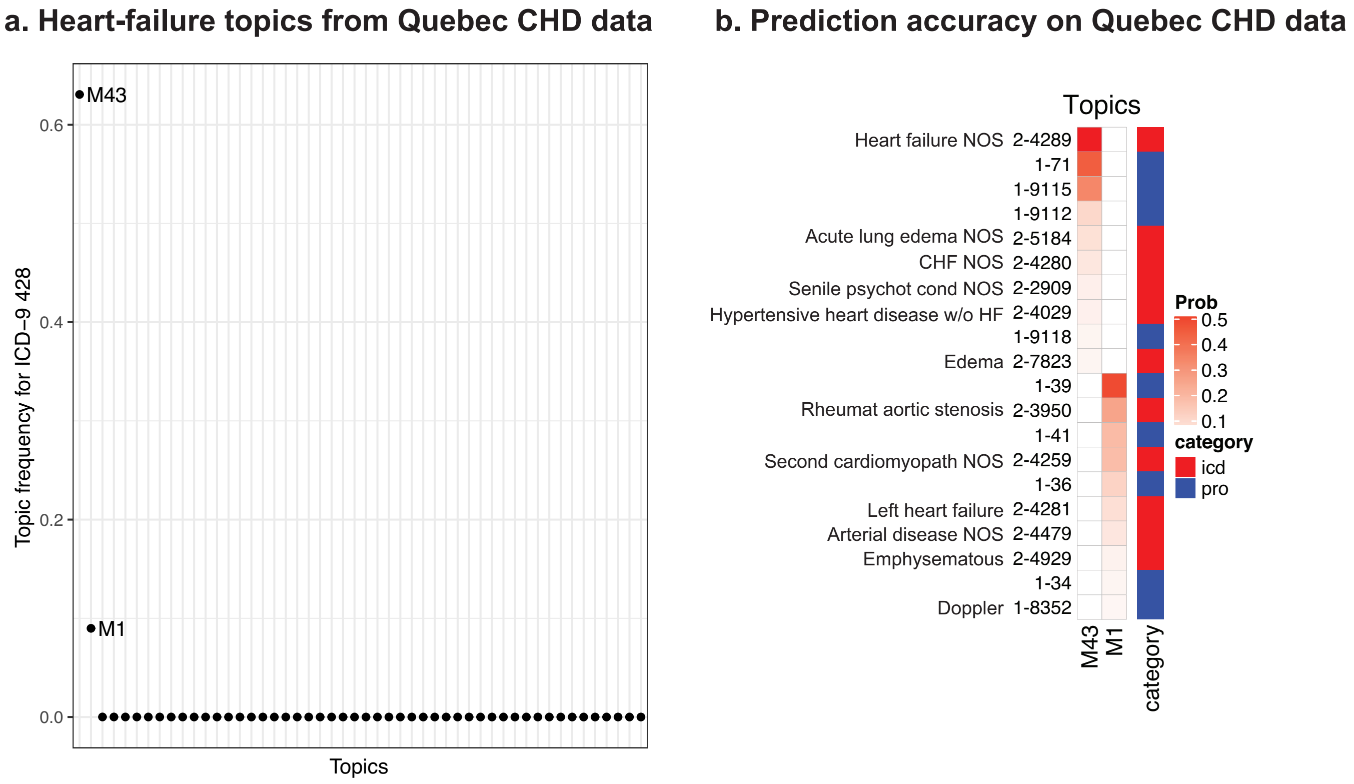

Figure 20: Learned MixEHR topics related to heart failure using the Quebec CHD dataset. **a**. Topics are sorted by their averaged frequency for the EHR code categories 428 (heart failure). **b**. Heatmap for the two latent topics out of the 50 topics that have inferred frequency for ICD-9 code 428 (Heart failure) greater than 0.01. ICD-9 codes start with “2-” prefix and intervention codes start with “1-” prefix. The meaning of the intervention codes were discussed in the main text.

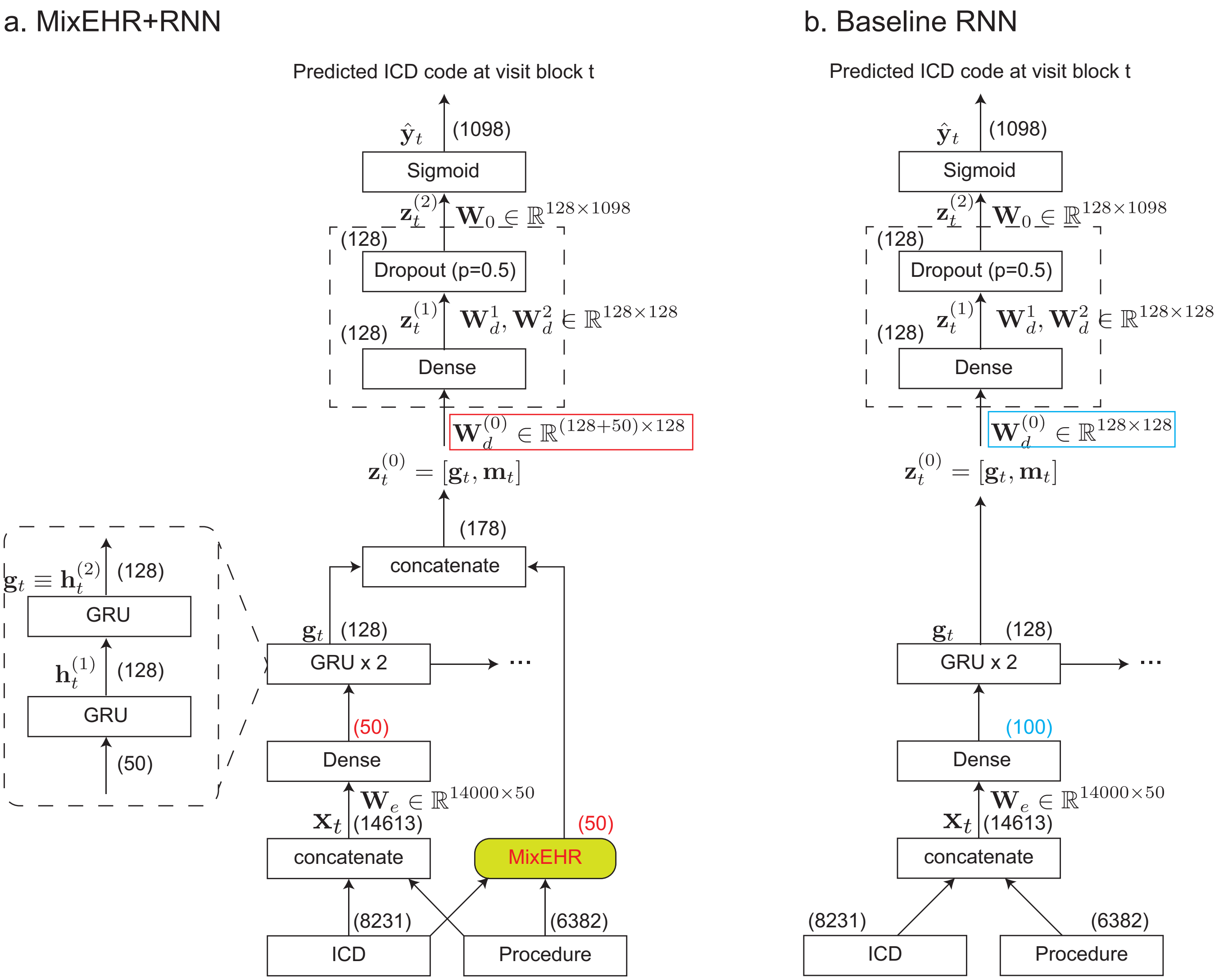

Figure 21: Architectures of the proposed RNN framework on predicting diagnostic code using the 28-year Quebec CHD Dataset. Dimension of each layer is indicated in the parentheses. Some of the connection weights are also indicated on the beside the arrow. **a**. The proposed MixEHR+RNN framework. The key component in the proposed model is the concatenation of the GRU output activities $\mathbf{g}_t$ and the MixEHR inferred topic mixture $\mathbf{m}_t$ at visit block t. The differences from the baseline RNN are highlighted in red. **b**. Baseline RNN framework. This is similar to the framework in Doctor AI [7]. The differences from the MixEHR+RNN are highlighted in cyan.

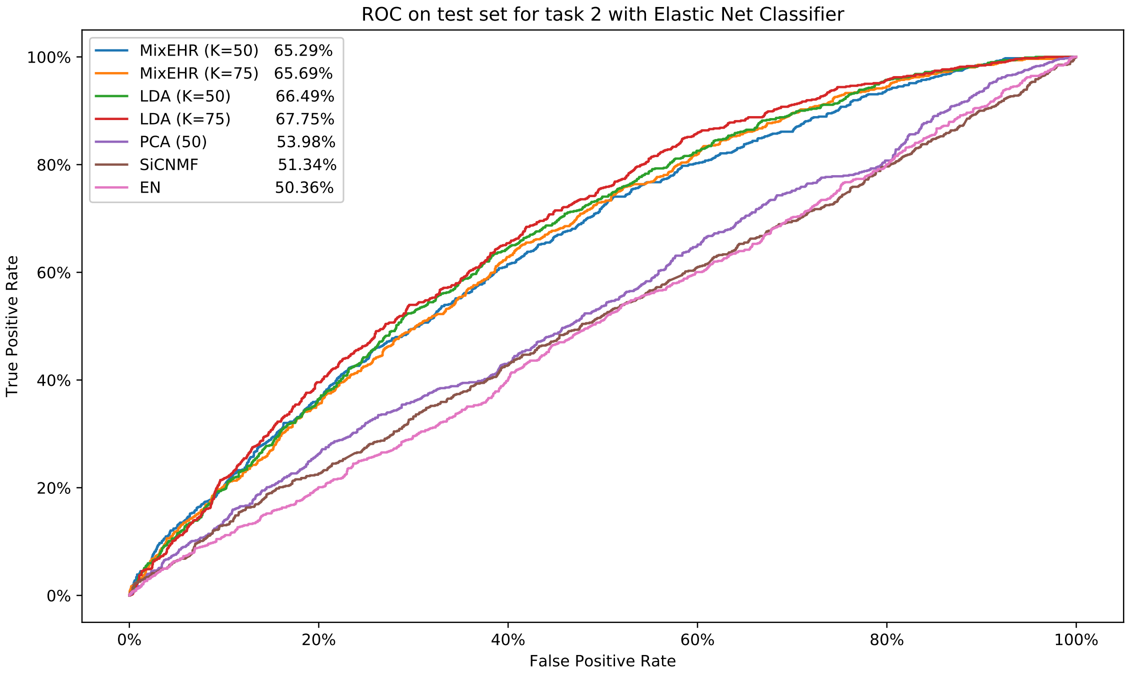

Figure 22: Mortality prediction in the MIMIC-III data. Each unsupervised embedding method was trained on the patients with only one admission in the MIMIC-III data. The trained model was then applied to embed the second last admission from patients with at least two admissions that are within 6 months apart. An elastic-net (EN) classifier was trained to predict the mortality outcome in the last admission. This was performed in a 5-fold cross-validation setting. ROC curves were generated and the area under of the ROC (AUROC) were displayed in the legend for each embedding method. ”EN” represents the performance using the raw EHR features.

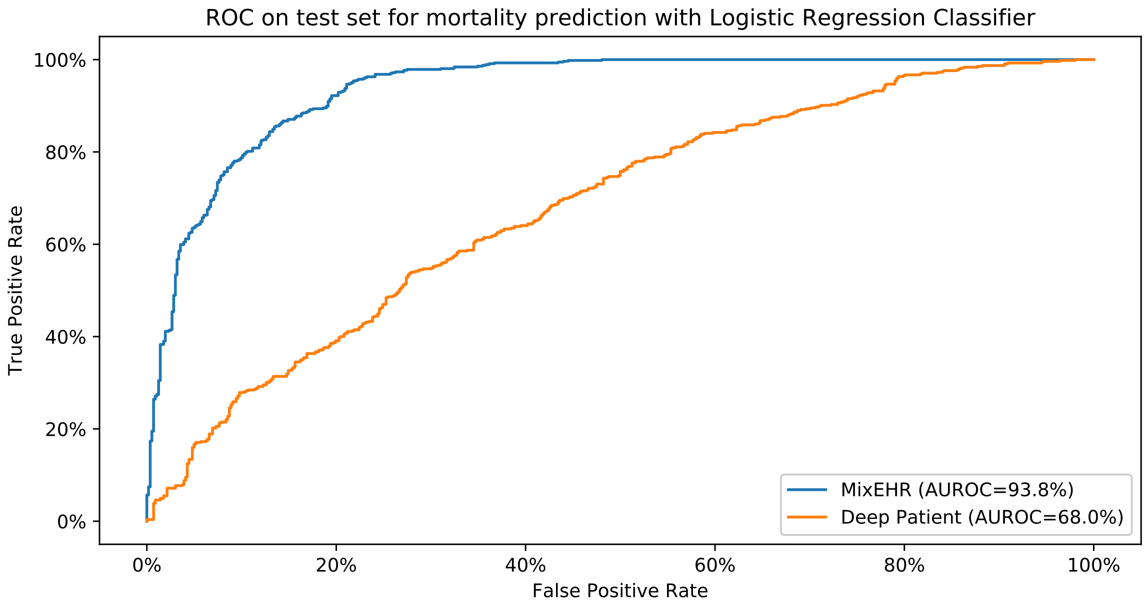

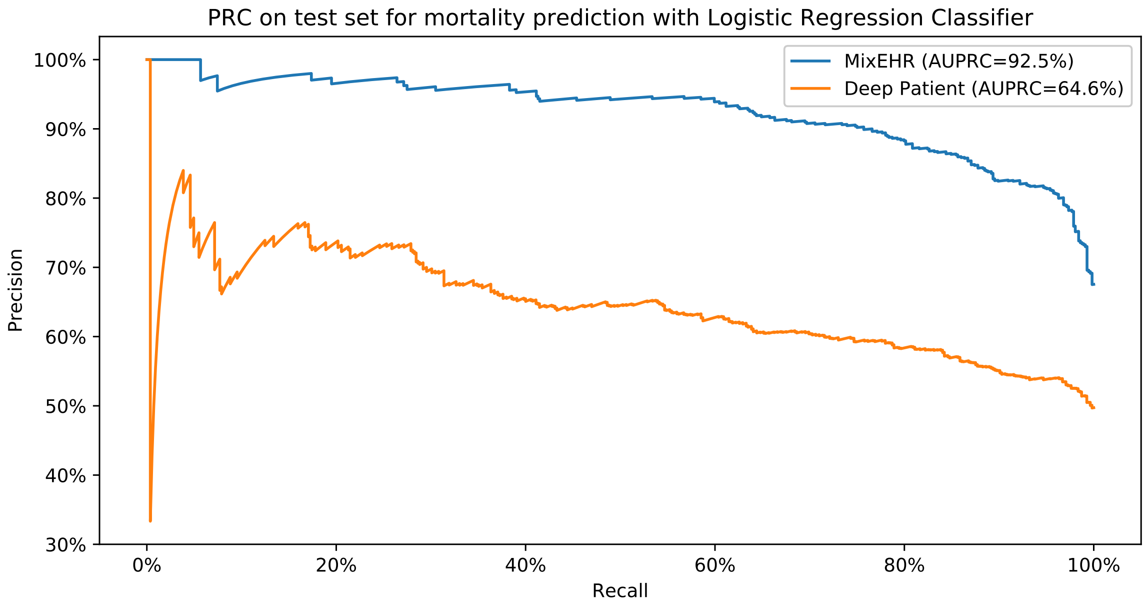

Figure 23: Mortality prediction comparison between MixEHR and deep patient (DP) embeddings using the MIMIC-III dataset. We evaluated the performance of predicting mortality within the same admission, which is a much easier task than predicting future mortality presented in the main text. We randomly split the admissions into 80% for training and 20% for testing. We then trained MixEHR model on the training set and a logistic regression (LR) classifier that used the input topic mixture to predict mortality label in that admission. We also trained DP on the same training set and a logistic regression (LR) classifier that used the input DP embedding to predict mortality label in that admission. We then applied the trained MixEHR+LR and DP+LR on the testing set. The performance was evaluated based on ROC and precision-recall curves.

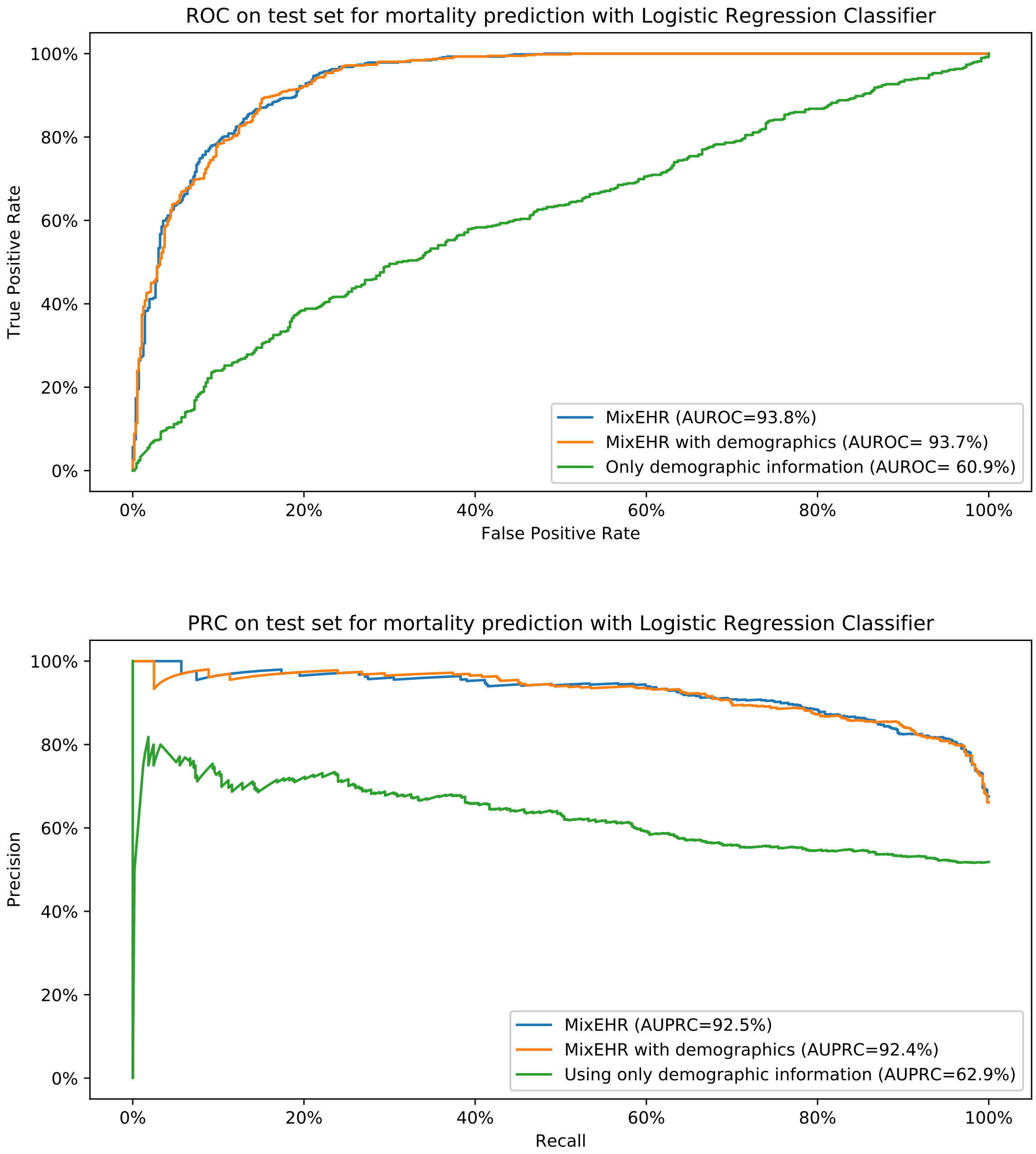

Figure 24: Mortality prediction using demographic information in the MIMIC-III dataset. We evaluated the performance of predicting mortality within the same admission, which is a much easier task than the one presented in the main text. We split the admissions into 80% for training and 20% for testing. We then trained MixEHR model on the training set and a logistic regression (LR) classifier that used the input topic mixture and/or demographic information including sex, age, and religion of the admitted patient and predict mortality label in that admission. We then applied the trained MixEHR and LR on the testing set. The performance was evaluated based on ROC and precision-recall curves.

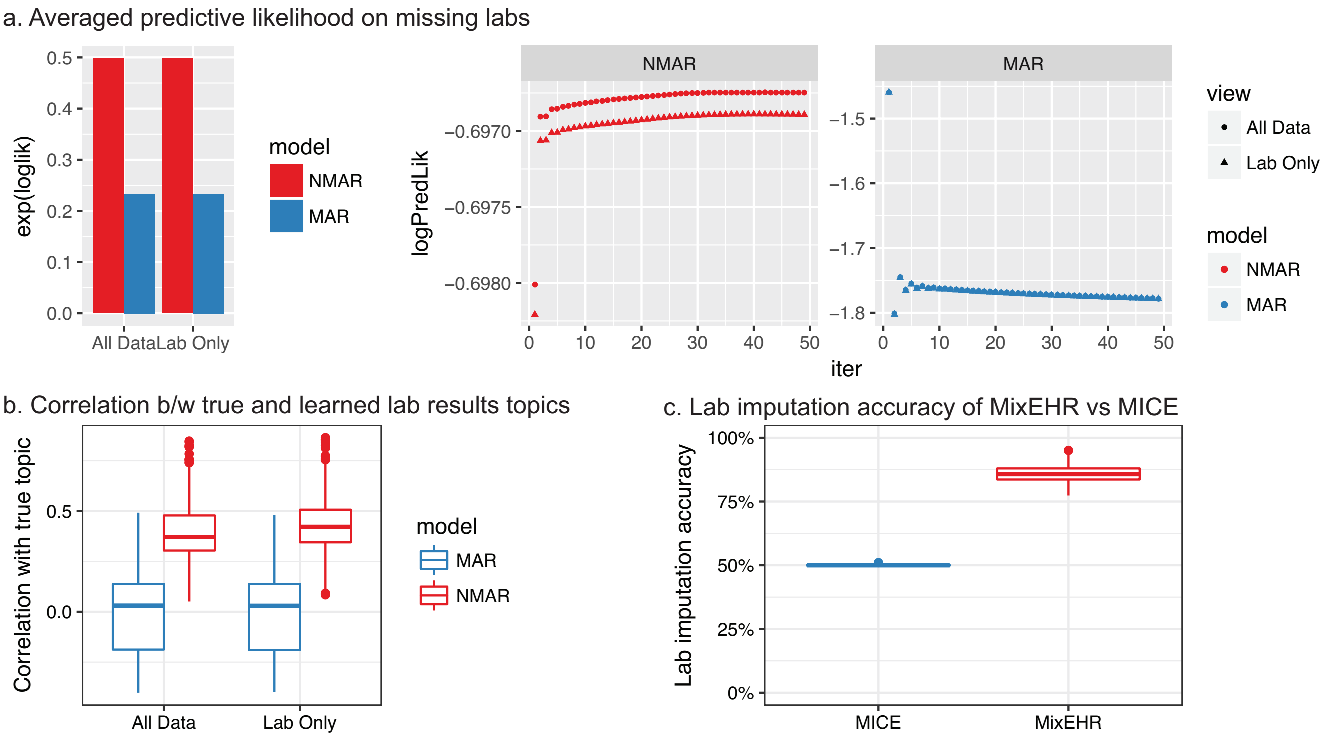

Figure 25: **a**. Predictive log likelihood of missing lab test results using four different MixEHR models using the simulated data from MIMIC-III. We simulated the EHR data using the $K = 75$ model trained on the MIMIC-III dataset. The predictive log likelihood was calculated over the missing lab test results only on the 5-fold CV held-out patients. Four model variables were evaluated: (1) MixEHR_nmar modeling NMAR using lab view only (labview); (2) MixEHR_nmar using all EHR (mixview); (3) & (4) MixEHR_mar assuming the lab results are missing at random (MAR) using lab (labview) and all EHR (mixview), respectively. **b**. Correlation between true and learned lab result topics. **c** Lab imputation accuracy of MixEHR versus MICE.

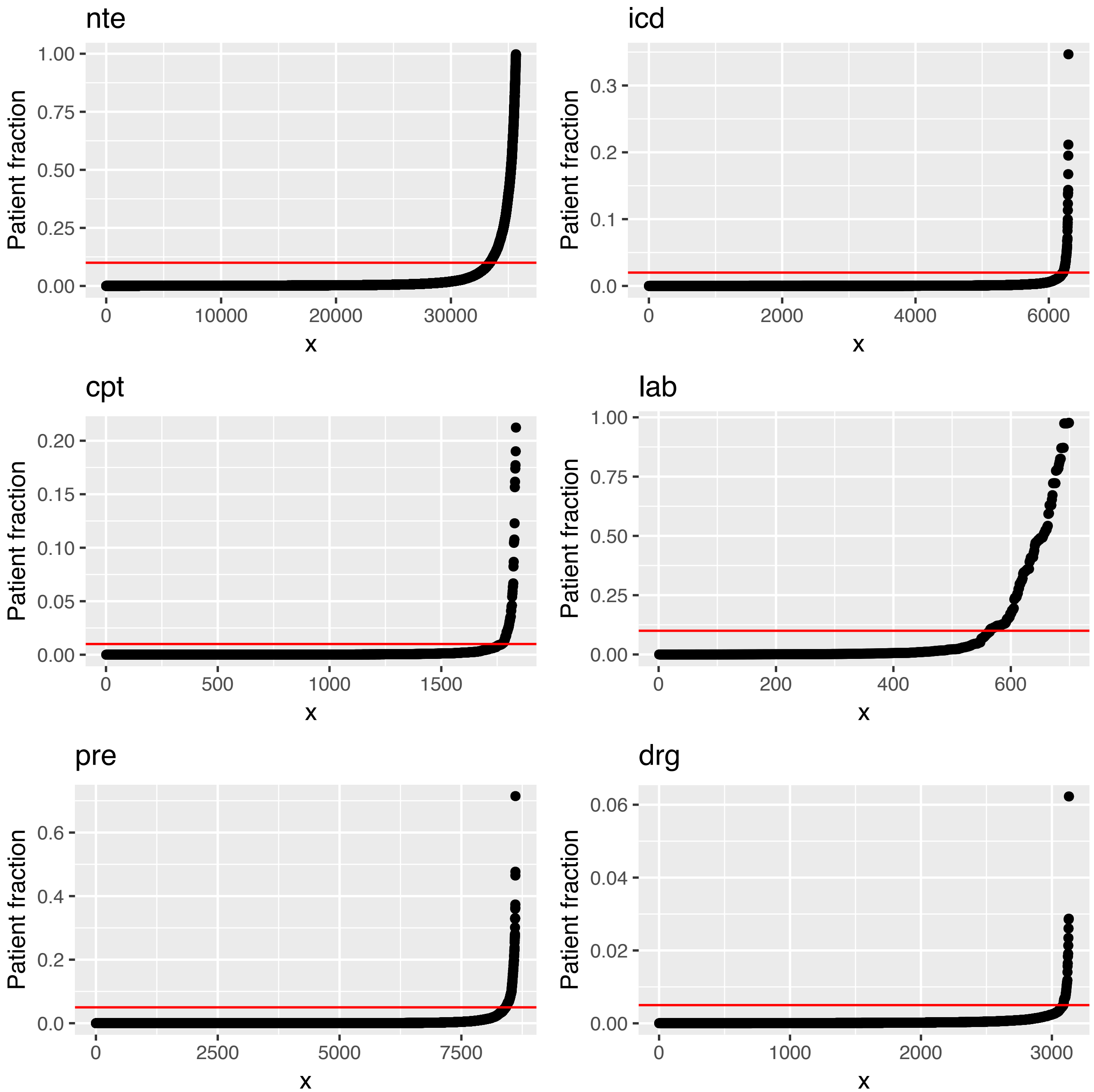

Figure 26: Frequency of EHR code in the MIMIC-III dataset. The frequency of each EHR code was plot in an increasing order. This allows us to identify the inflection points that we used to filter out non-informative commonly occurring EHR code.

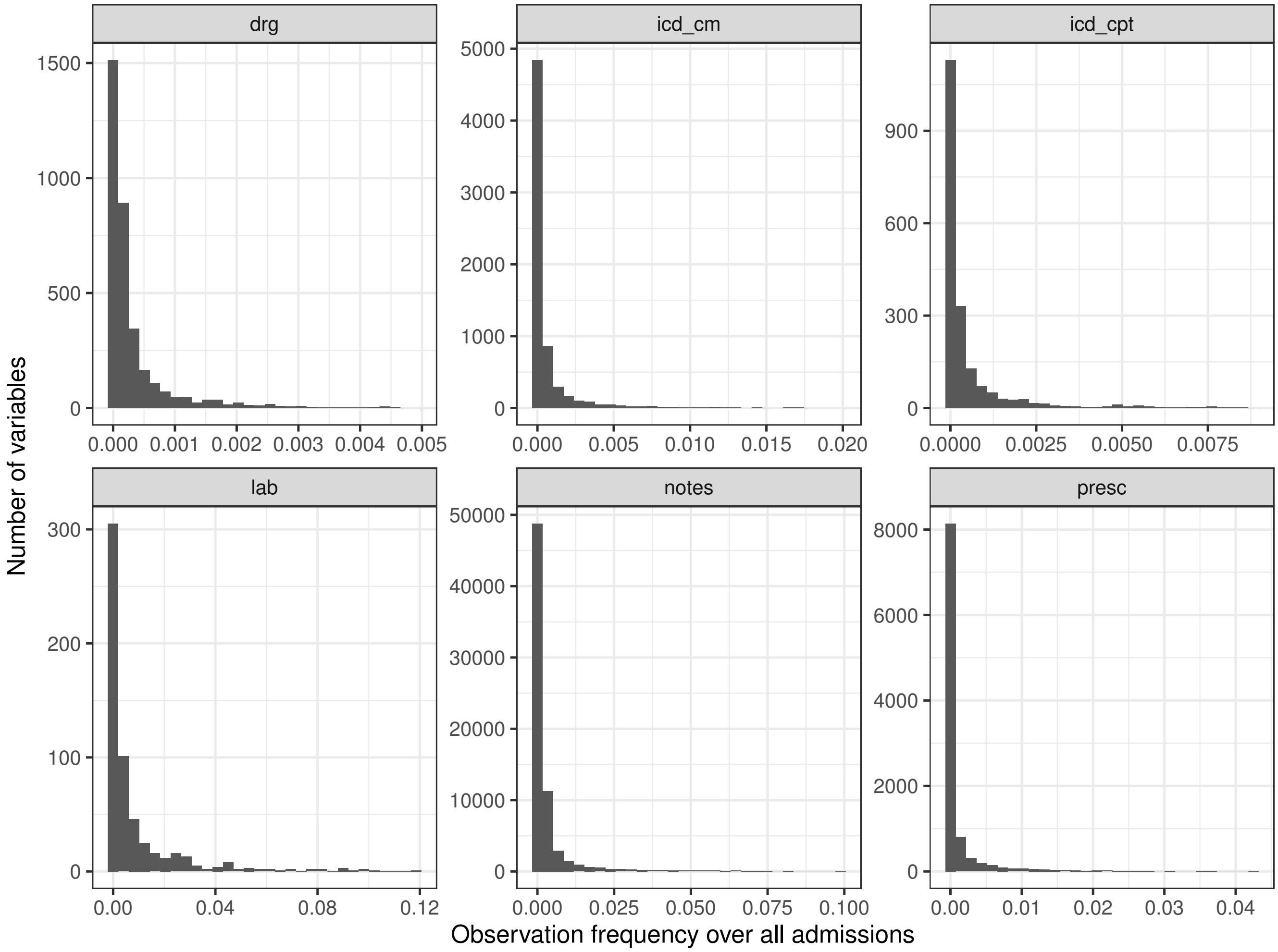

Figure 27: Frequency of EHR code in the processed MIMIC-III dataset. The histogram displays the frequency of observed EHR code over all of the admissions.

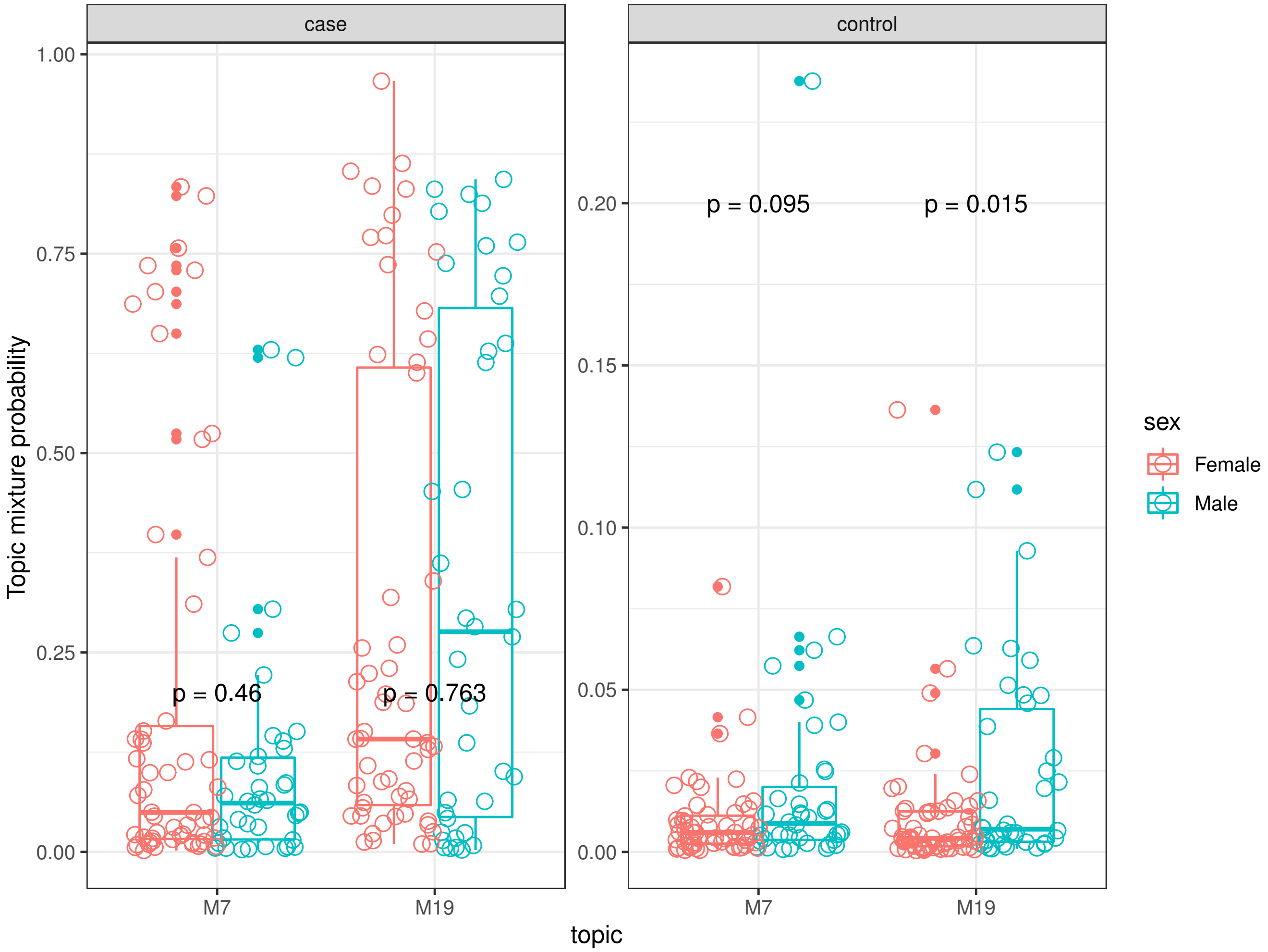

Figure 28: Sex differences for the M19 and M1 BD-related topics from Mayo Clinic dataset. For each topic M7 or M19, we calculated the difference of topic mixture probability between the sex groups for the cases or controls. Boxplots were displayed separately for cases and controls. Each individual patient was shown as bubble superimposed on the boxplot. The displayed p-values were calculated based on Wilcoxon two-sided rank-sum test.

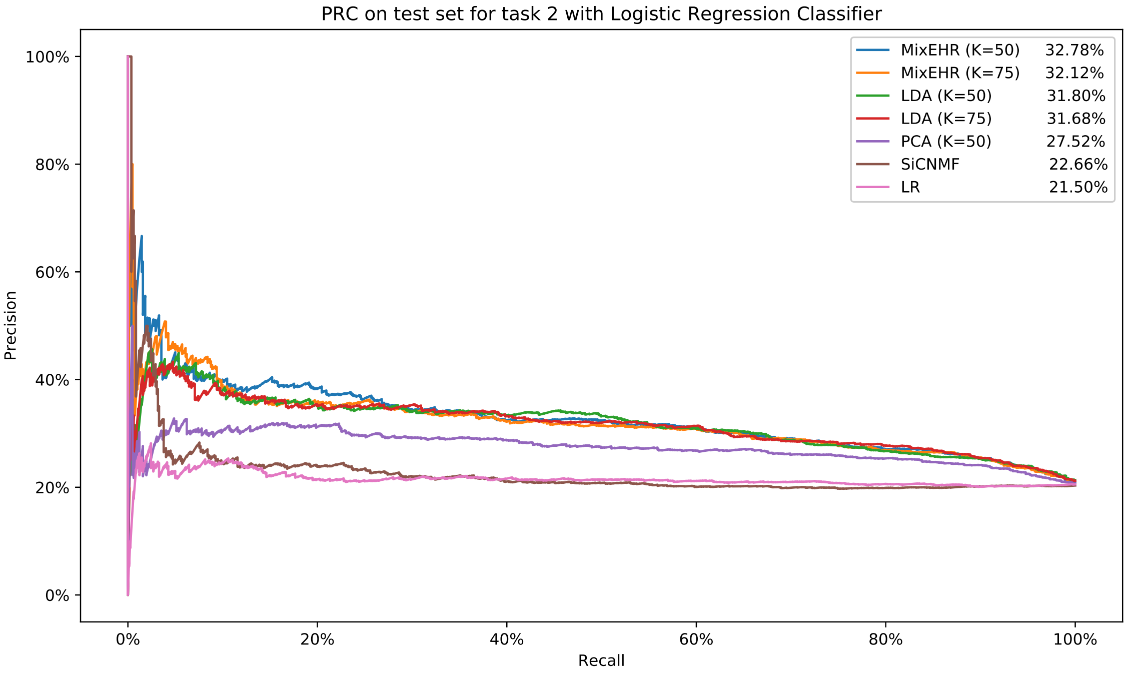

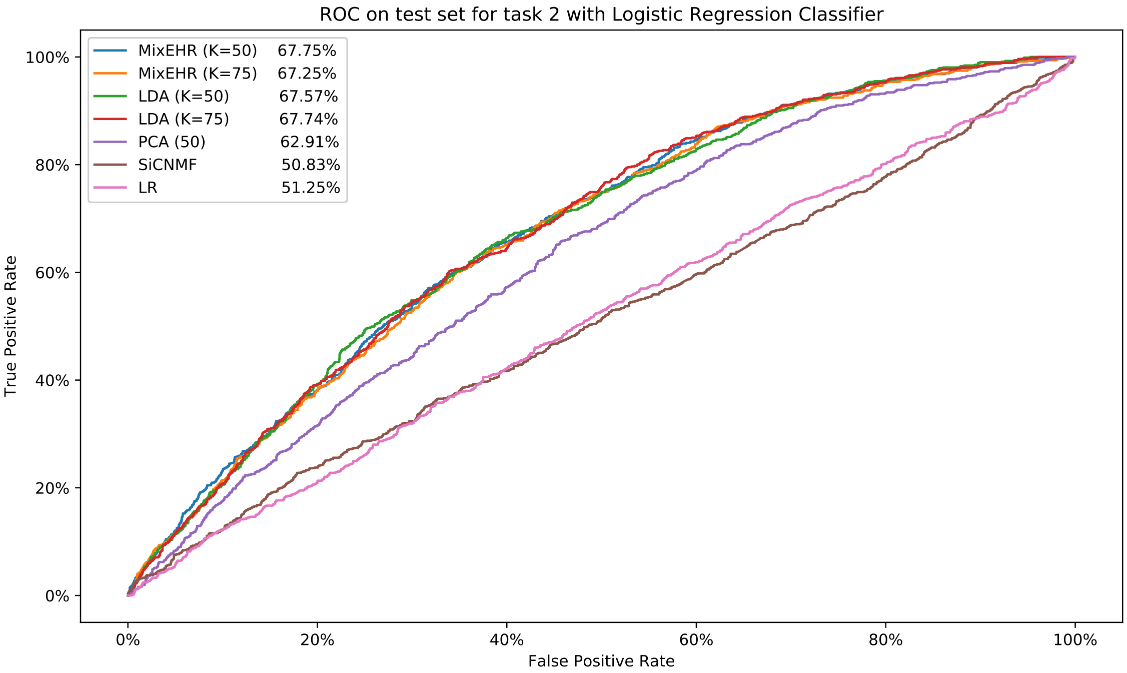

Figure 29: Mortality prediction using logistic regression with L2-norm evaluated on the MIMIC-III dataset. The evaluation procedure is the same as for the elastic net classifier described in **Supplementary Figure** 22 and the main text.

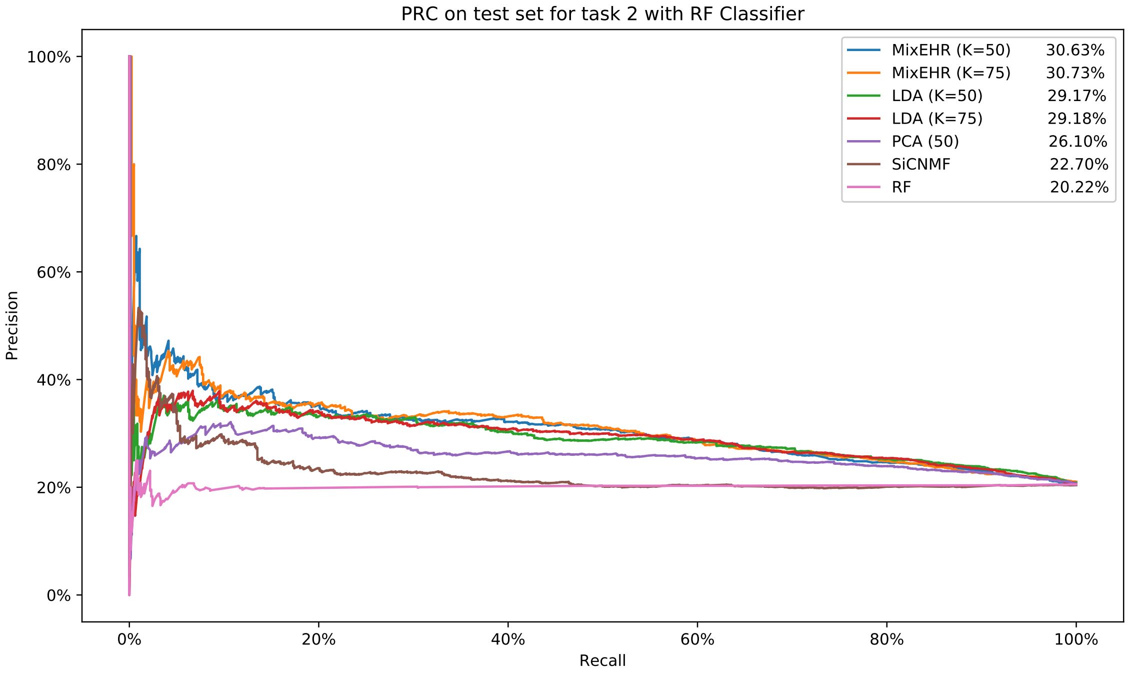

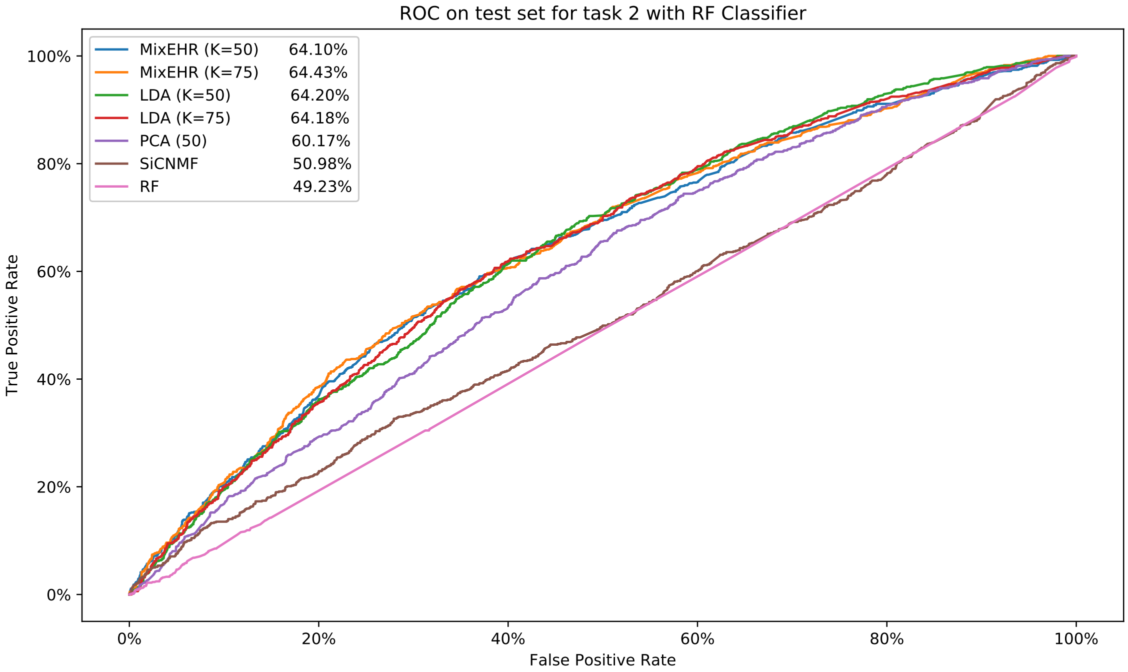

Figure 30: Mortality prediction using Random Forest evaluated on the MIMIC-III dataset. The evaluation procedure is the same as for the elastic net classifier described in **Supplementary Figure** 22 and the main text.

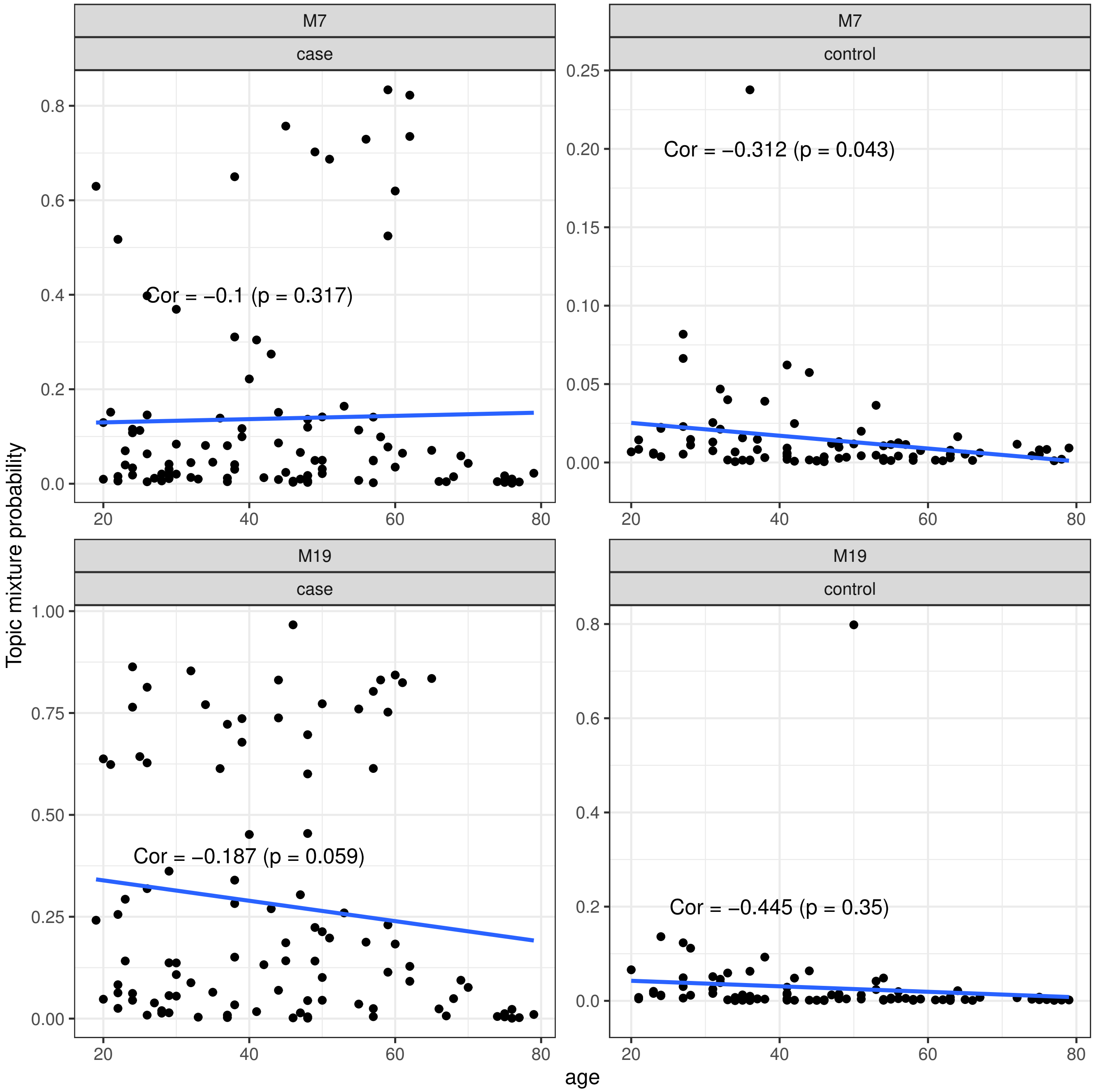

Figure 31: Spearman correlation between topic mixture and ages using the Mayo Clinic dataset. P-values were calculated based on correlation test function `cor.test` in R.

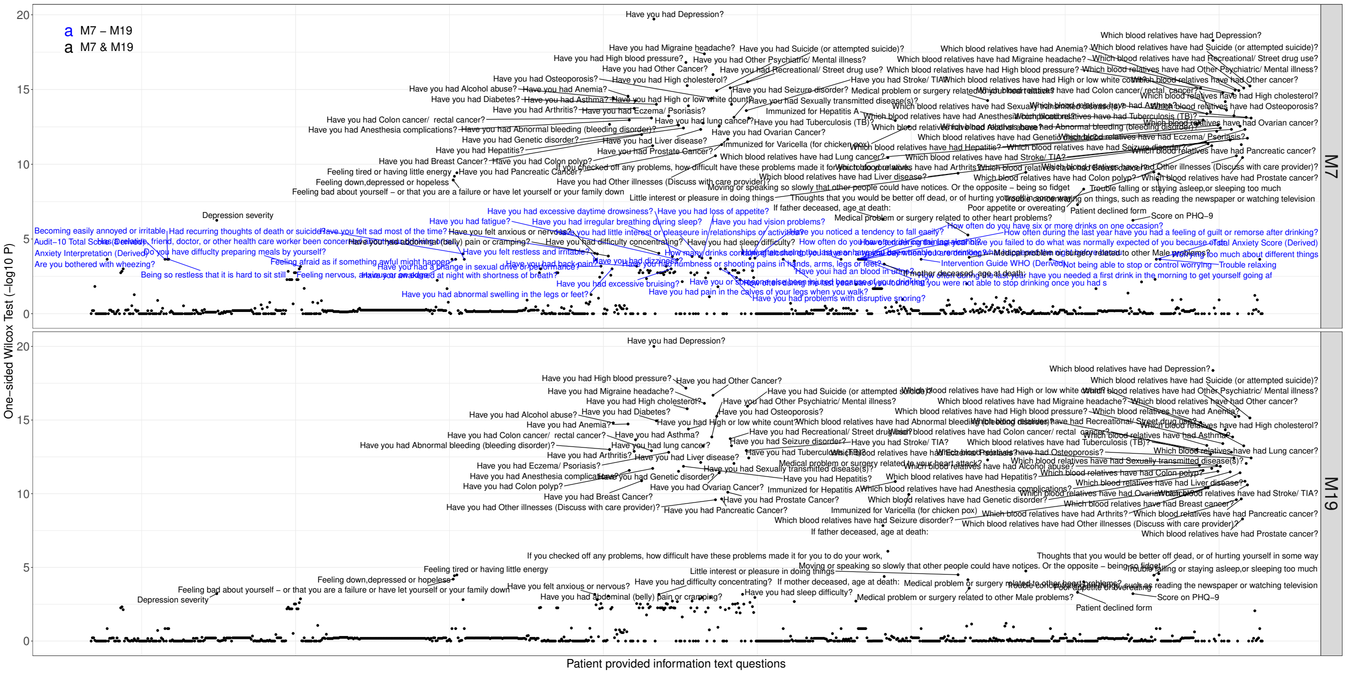

Figure 32: Phenome-wide association studies of the 955 patient provided information (PPI) questions on the two bipolar disorder mixture topics (M7 and M19) based on the Mayo Clinic dataset. For each PPI question, we tested the difference between patient groups with and without that code in terms of M19 and M7. The labelled PPI questions are the ones with Wilcoxon one-sided test p-values < 0.001. The red, blue, and black color indicate the codes that are significant in only M19, in only M7, and in both M19 and M7, respectively. No significant PPI question is detected for only the M19 topic but not M7 whereas many questions are significantly only for M7 but not M19.

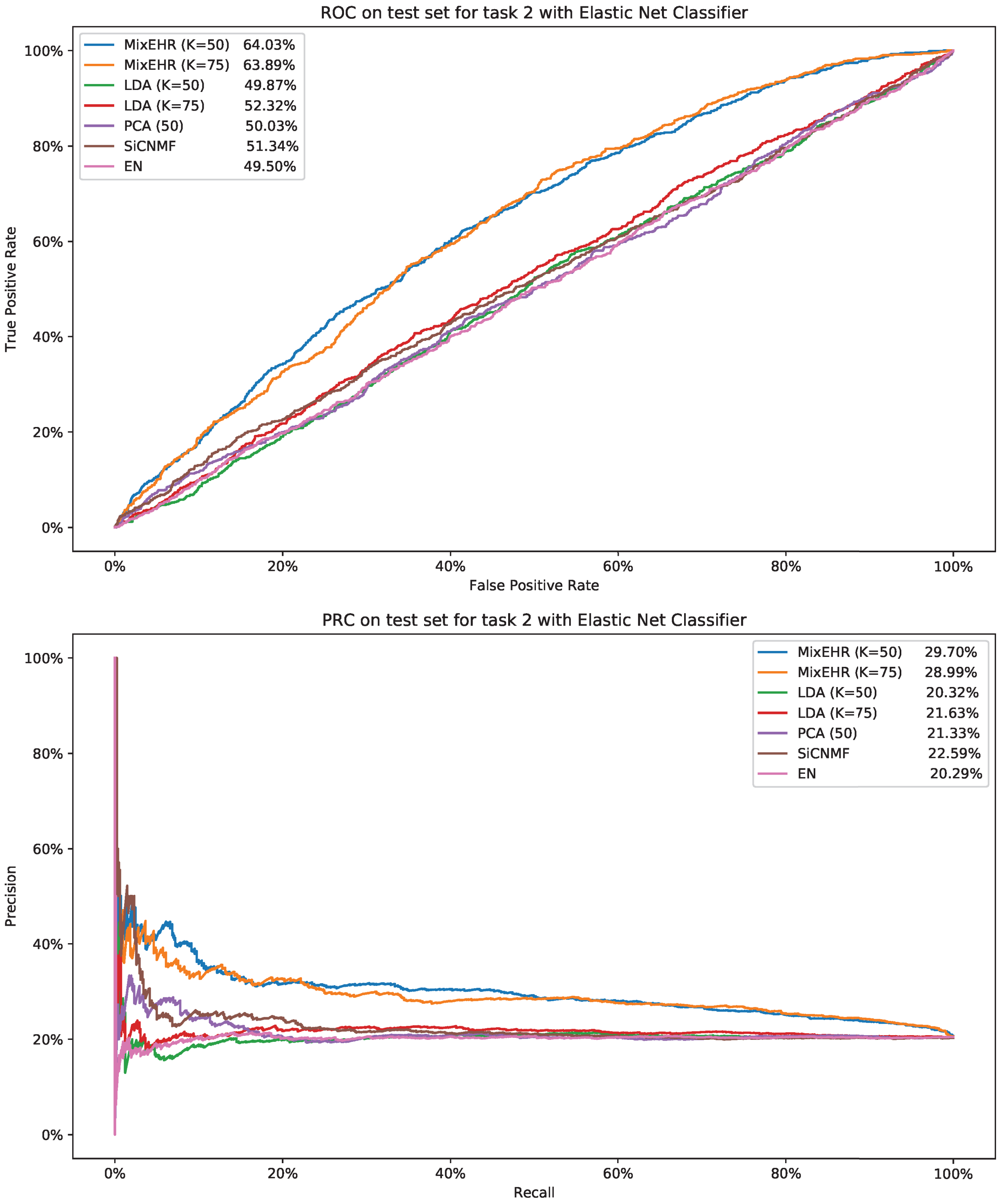

Figure 33: Mortality prediction in the MIMIC-III data. Each unsupervised embedding method was trained on the patients with only one admission in the MIMIC-III data. The trained model was then applied to embed the first admission from patients that lasted shorter than 2 days. An elastic-net (EN) classifier was trained to predict the mortality outcome in the last admission. This was performed in a 5-fold cross-validation setting. ROC curves were generated and the area under of the ROC (AUROC) were displayed in the legend for each embedding method. ”EN” represents the performance using the raw EHR features.

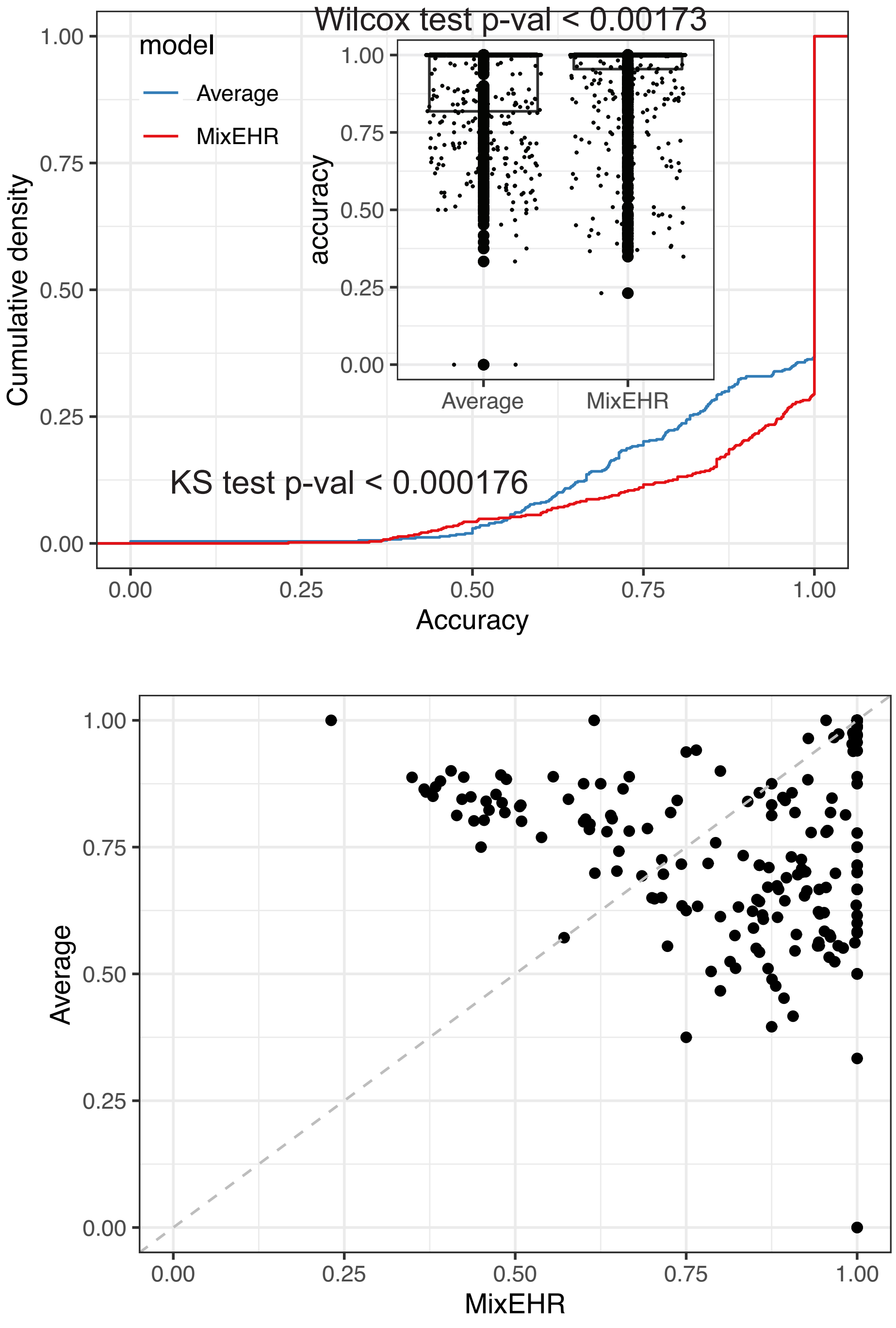

Figure 34: Lab results imputation using MIMIC-III dataset. We compared the imputation accuracy between MixEHR and using the average lab values over the training admissions. We generated the cumulative density function (CDF) of accuracy as well as the boxplot distributions (inset) for each method. In both cases, MixEHR significantly outperformed CF-RBM based on KS-test ($p<0.000176$) and Wilcoxon Signed Rank one-sided test ($p<0.00173$). **b** Average versus MixEHR imputation accuracy scatterplot in terms of imputation accuracy.

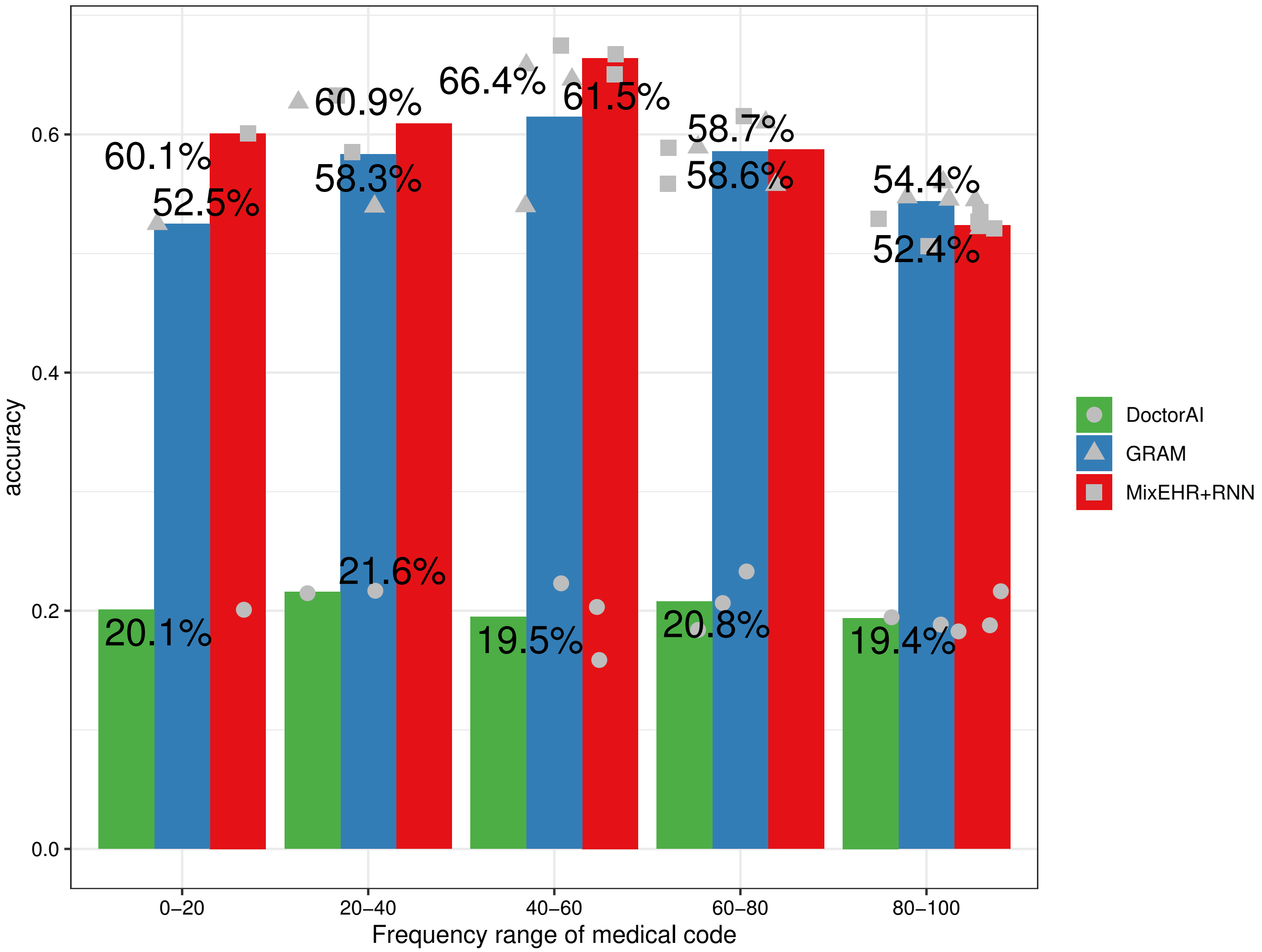

Figure 35: Medical code prediction accuracy. We binned the CCS codes based on percentile of their frequencies in the training data in non-decreasing order. Therefore, 0-20 are the most rare diagnoses and 80-100 are the most common ones. For each medical code, the accuracy was calculated by true positives among the top 20 predicted codes divided by the total number of positives. The dots are individual accuracies for each code predicted by each method.

Supplementary Tables

Data type	Total†
Total training patients (single admission)	**38,981**
Total test patients (multiple admissions)	7,541
Words in clinical notes	33,388
ICD-9 diagnoses codes	6,215
ICD-9 procedural codes	1,770
Lab test* (mostly LOINC codes)	564
Prescription (drug generic code)	8,409
Diagnosis-related group (DRG) codes	3,086
Total unique clinical features	**53,432**
Total measured features in training set	12,833,185
Total measured features in test set	3,198,163

Table 1: Mimic-III EHR data summary. *Lab test data contain both record and result flags of the tests.

Topic ID	Short Name	Long Name
M1	Allergies	Hypersensitivity (allergic) reactions
M2	Bowel procedures	Procedures of the bowel
M3	End stage kidney disease	End stage kidney disease
M4	Subarachnoid hematoma	Subarachnoid hematoma
M5	Acute myocardial infarction	Acute myocardial infarction
M6	Respiratory and digestive cancers	Malignancies of the respiratory and digestive systems
M7	Skin infections	Infections of the skin
M8	Intracranial hypertension	Intracranial hypertension
M9	unclear	unclear
M10	Pericardial diseases	Diseases of the pericardium
M11	Heart failure	Heart failure and sequelae
M12	Renal infections	Infections of the kidneys and urinary tract
M13	Hypothyroidism	Hypothyroidism
M14	Cardiac conduction defects	Diseases of the cardiac conduction system
M15	Spinal disorders/procedures	Disorders and procedures of the spine
M16	unclear	unclear
M17	Alcoholism	Alcohol abuse and sequelae
M18	Cardiac arrest	Cardiac arrest
M19	Musculoskeletal trauma	Musculoskeletal trauma
M20	Heart failure	Heart failure
M21	Premature birth	Complications of premature birth
M22	unclear	unclear

M23	Septicemia	Septicemia
M24	Vascular disease	Vascular disease
M25	Infectious disease	Various infectious diseases
M26	Hypertensive diseases	Hypertensive diseases
M27	Infectious disease	Various infectious diseases
M28	Gastritis	Peptic ulcer and gastritis
M29	unclear	unclear
M30	End stage kidney disease	End stage kidney disease
M31	Cirrhosis	Cirrhosis and sequelae
M32	Cardiac diseases	Cardiac diseases and procedures
M33	Seizure syndrome	Seizure syndrome
M34	unclear	unclear
M35	Pulmonary embolism	Pulmonary embolism
M36	Ventilator use	Respiratory disease resulting in use of ventilator
M37	Gastrointestinal diseases	Major gastrointestinal and peritoneal infections
M38	Pressure ulcer	Pressure ulcers and complications
M39	unclear	unclear
M40	Interstitial lung disease	Interstitial lung disease
M41	unclear	unclear
M42	HIV	HIV and sequelae
M43	Repiratory procedures	Major respiratory and chest procedures
M44	Cardiovascular	Various cardiovascular diseases
M45	Intracranial vasculopathy	Intracranial vasculopathy
M46	Pneumonia	Pneumonia
M47	Obstructive pulmonary disease	Obstructive pulmonary disease
M48	Diabetes	Diabetes and sequelae
M49	Biliary diseases	Disorders of the biliary tract
M50	Leukemia	Leukemia and lymphoma
M51	Trauma	Traumatic injuries
M52	Trauma	Traumatic injuries
M53	Intracranial hypertension	Intracranial hypertension
M54	Coronary/valvular diseases	Coronary artery and valvular diseases
M55	Arrythmia	Cardiac arrythmia and conduction disorders
M56	unclear	unclear
M57	Valvular disease	Cardiac valvular diseases
M58	Vascular procedures	Vascular procedures
M59	Dementia	Dementia and sequelae
M60	Tracheostomy	Tracheostomy and its complications
M61	Digestive system diseases	Procedures of the digestive system
M62	Psychiatric diseases	Various psychiatric diseases
M63	unclear	unclear

M64	Embolic stroke	Embolic stroke
M65	Intracranial hemmorhage	Post-traumatic intracranial hemmorhage
M66	Coronary artery disease	Coronary artery disease and treatments
M67	Digestive system procedures	Procedures of the digestive system
M68	Premature birth	Premature birth and complications
M69	Organ transplant	Organ transplant
M70	Uterine and adnexal procedures	Procedures of the uterus and adnexa
M71	Diseases of the colon	Diseases of the colon
M72	unclear	unclear
M73	Upper GI procedures	Procedures of the upper digestive tract
M74	Neonate	Neonatal care
M75	Meningeal disease	Diseases of the meninges

Table 2: Annotated latent topic based on the top EHR codes. "?" indicates that the topics are not well defined.

Notation	Variable
K	Number of disease topics
D	Number of patients
$W^{(t)}$	Number of distinct EHR features from data type t
V_l	Number of distinct values of lab l
$M_j^{(t)}$	Number of features from data type t measured on patient j
L	Number of lab tests
$\phi_k^{(t)}$	Frequency of EHR features of data type t for disease topic k, $W^{(t)} \times 1$
η_{lk}	Frequency of the lab values for lab l under topic k, $V_l \times 1$
ψ_{lk}	Frequency of observing lab l under topic k
θ_j	Disease mixture memberships of patient j, $1 \times K$
α_k	Hyperparameter of kth component of the Dirichlet topic mixture θ_j
β_{wt}	Hyperparameter of data type t feature wth component for $\phi_k^{(t)}$
ζ_{lv}	Hyperparameter of the vth lab value component of η_{lk} for lab l
a_l, b_l	Hyperparameters for scale and shape of the lab l frequency ψ_{lk}
$z_{ij}^{(t)}$	Latent topic of feature i from data type t of patient j
h_{lj}	Latent topic of lab l of patient j
$x_{ij}^{(t)}$	Observed feature i from data type t on patient j, $x_{ij}^{(t)} \in \{1, \ldots, W^{(t)}\}$
y_{lj}	Value of lab l measured on patient j, $y_{lj} \in \{1, \ldots, V_l\}$
y_{ljv}	Frequency of observing lab l with value v on patient j, $y_{ljv} \in [0, m_{lj}]$
r_{lj}	Observation indicator for lab l on patient j, $r_{lj} \in \{0, 1\}$

Table 3: MixEHR model notations

Table 4: Prediction performance of the 976 target EHR codes. See TableS3_impmimic_perf.csv table

Data type	Total†
ICD-9 codes	2,224
Procedure codes (CPT code)	2,200
Patient provided information (question text)	955
Lab test (lab test code)	1,742
Prescription (medication code)	610
Total EHR codes	7,731
Total observed clinical features	**108,390**

Table 5: Mayo Clinic Dataset.

age	Controls		Cases	
	F	M	F	M
19-32	15	5	16	9
32-44	13	12	10	9
44-57	15	8	16	6
57-79	10	11	10	11

Table 6: Sex-by-age distribution in cases and controls by age percentage.

	Grouped by Day	Grouped by Month
Number of visits	13,437,510	6,851,225
Total number of subjects	84498	84498
Total number of ICD-9 code	8231	8231
Total number of intervention code	6382	6382
Average number of visits per subject	159.028	81.082
Average number of diagnoses	1.276	2.502
Average number of interventions	1.549	3.038
Maximum number of visits per subject	3532	333
Maximum number of diagnoses	38	284
Maximum number of interventions	39	284

Table 7: Summary of Quebec CHD outpatients dataset. Since visits are one time events, the data were summarized by day and by month. In our experiment, we adopted the monthly data as the input because of the scalability issue for the daily data.

Supplementary References

1. Bishop, C. Pattern recognition and machine learning. *library.wisc.edu* (2006).

2. Teh, Y. W., Newman, D. & Welling, M. A collapsed variational Bayesian inference algorithm for latent Dirichlet allocation. *Advances in neural ...* (2006).

3. Asuncion, A., Welling, M., Smyth, P. & Teh, Y. W. On smoothing and inference for topic models. In *Proceedings of the Twenty-Fifth Conference on Uncertainty in Artificial Intelligence*, UAI '09, 27–34 (AUAI Press, Arlington, Virginia, United States, 2009). URL `http://dl.acm.org/citation.cfm?id=1795114.1795118`.

4. Minka, T. Estimating a Dirichlet distribution. *Technical Report* (2000).

5. Hoffman, M. D., Blei, D. M., Wang, C. & Paisley, J. W. Stochastic variational inference. *Journal of Machine Learning Research* (2013).

6. Foulds, J., Boyles, L., Dubois, C., Smyth, P. & Welling, M. Stochastic Collapsed Variational Bayesian Inference for Latent Dirichlet Allocation. *arXiv.org* (2013). `1305.2452v1`.

7. Choi, E., Bahadori, M. T., Schuetz, A., Stewart, W. F. & Sun, J. Doctor AI: Predicting Clinical Events via Recurrent Neural Networks. *JMLR Workshop Conf. Proc.* **56**, 301–318 (2016).

8. Chung, J., Gulcehre, C., Cho, K. & Bengio, Y. Empirical evaluation of gated recurrent neural networks on sequence modeling. *arXiv preprint arXiv:1412.3555* (2014).

9. Cho, K., Van Merriënboer, B., Bahdanau, D. & Bengio, Y. On the properties of neural machine translation: Encoder-decoder approaches. *arXiv preprint arXiv:1409.1259* (2014).

10. Glorot, X. & Bengio, Y. Understanding the difficulty of training deep feedforward neural networks. In *Proceedings of the thirteenth international conference on artificial intelligence and statistics*, 249–256 (2010).

11. Zeiler, M. D. Adadelta: an adaptive learning rate method. *arXiv preprint arXiv:1212.5701* (2012).

12. Kingma, D. P. & Ba, J. Adam: A method for stochastic optimization. *arXiv preprint arXiv:1412.6980* (2014).

13. White, I. R., Royston, P. & Wood, A. M. Multiple imputation using chained equations: Issues and guidance for practice. *Statistics in Medicine* **30**, 377–399 (2010).

14. Wells, B. J., Nowacki, A. S., Chagin, K. & Kattan, M. W. Strategies for Handling Missing Data in Electronic Health Record Derived Data. *eGEMs (Generating Evidence & Methods to improve patient outcomes)* **1**, 7–9 (2017).

15. van Buuren, S. & Groothuis-Oudshoorn, K. mice: Multivariate Imputation by Chained Equations inR. *Journal of Statistical Software* **45** (2011).

16. Salakhutdinov, R., Mnih, A. & Hinton, G. Restricted Boltzmann machines for collaborative filtering. In *Proceedings of the 24th international conference on Machine learning*, 791–798 (ACM Press, New York, New York, USA, 2007).

17. Mnih, A. & Salakhutdinov, R. R. Probabilistic matrix factorization. In *Advances in neural information processing systems*, 1257–1264 (2008).

18. Hernandez-lobato, J. M., Houlsby, N. & Ghahramani, Z. Probabilistic Matrix Factorization with Non-random Missing Data. *ICML* (2014).

19. Marlin, B. M. & Zemel, R. S. Collaborative prediction and ranking with non-random missing data. In *the third ACM conference*, 5–12 (ACM Press, New York, New York, USA, 2009).

20. Fraser, G. & Yan, R. Collaborative Filtering and the Missing at Random Assumption. *Epidemiology* **18**, 1–9 (2016).

21. Blei, D. M., Ng, A. Y. & Jordan, M. I. Latent dirichlet allocation. *J. Mach. Learn. Res.* **3**, 993–1022 (2003).

22. Mcauliffe, J. D. & Blei, D. M. Supervised topic models. In Platt, J. C., Koller, D., Singer, Y. & Roweis, S. T. (eds.) *Advances in Neural Information Processing Systems 20*, 121–128 (Curran Associates, Inc., 2008).

23. Blei, D. M. Probabilistic topic models. *Commun. ACM* **55**, 77–84 (2012).

24. Griffiths, T. L. & Steyvers, M. Finding scientific topics. *Proceedings of the National Academy of Sciences of the United States of America* **101 Suppl 1**, 5228–5235 (2004).

25. Teh, Y. W., Newman, D. & Welling, M. A collapsed variational bayesian inference algorithm for latent dirichlet allocation. In Schölkopf, B., Platt, J. C. & Hoffman, T. (eds.) *Advances in Neural Information Processing Systems 19*, 1353–1360 (MIT Press, 2007).

26. Asuncion, A., Welling, M., Smyth, P. & Teh, Y. W. On smoothing and inference for topic models. In *Proceedings of the Twenty-Fifth Conference on Uncertainty in Artificial Intelligence*, UAI ’09, 27–34 (AUAI Press, Arlington, Virginia, United States, 2009).

27. Wang, Y. *et al.* Unsupervised machine learning for the discovery of latent disease clusters and patient subgroups using electronic health records. *JOURNAL OF BIOMEDICAL INFORMATICS* **102**, 103364 (2020).

28. Little, R. J. A. & Rubin, D. B. *Statistical Analysis with Missing Data* (John Wiley & Sons, 2014).

29. Halpern, Y., Horng, S. & Sontag, D. Clinical Tagging with Joint Probabilistic Models. *arXiv. org* (2016).

30. Joshi, S., Gunasekar, S., Sontag, D. & Ghosh, J. Identifiable Phenotyping using Constrained Non-Negative Matrix Factorization. *arXiv.org* (2016). 1608.00704v3.

31. Pivovarov, R. *et al.* Learning probabilistic phenotypes from heterogeneous EHR data. *J. Biomed. Inform.* **58**, 156–165 (2015).

32. Gunasekar, S. *et al.* Phenotyping using structured collective matrix factorization of multi–source EHR data. *arXiv* (2016). 1609.04466.

33. Flaherty, P., Giaever, G., Kumm, J., Jordan, M. I. & Arkin, A. P. A latent variable model for chemogenomic profiling. *Bioinformatics* **21**, 3286–3293 (2005).

34. Zhao, J. *et al.* Detecting time-evolving phenotypic topics via tensor factorization on electronic health records Cardiovascular disease case study. *JOURNAL OF BIOMEDICAL INFORMATICS* **98**, 103270 (2019).

35. Wang, L., Tong, L., Davis, D., Arnold, T. & Esposito, T. The application of unsupervised deep learning in predictive models using electronic health records. *BMC Medical Research Methodology* **20**, 1–9 (2020).

36. Johnson, A. E. W. *et al.* Machine learning and decision support in critical care. *Proc. IEEE Inst. Electr. Electron. Eng.* **104**, 444–466 (2016).

37. Pathak, J., Kho, A. N. & Denny, J. C. Electronic health records-driven phenotyping: challenges, recent advances, and perspectives. *J. Am. Med. Inform. Assoc.* **20**, e206–11 (2013).

38. Hripcsak, G. & Albers, D. J. Next-generation phenotyping of electronic health records. *J. Am. Med. Inform. Assoc.* **20**, 117–121 (2013).

39. Razavian, N. & Sontag, D. Temporal Convolutional Neural Networks for Diagnosis from Lab Tests. *arXiv. org* (2015).

40. Cheng, Y., Wang, F., Zhang, P. & Hu, J. Risk prediction with electronic health records: A deep learning approach. *2016 SIAM International Conference* 432–440 (2016).

41. Lipton, Z. C., Kale, D. C., Elkan, C. & Wetzel, R. Learning to Diagnose with LSTM Recurrent Neural Networks. *arXiv. org* (2015).

42. Nguyen, P., Tran, T., Wickramasinghe, N. & Venkatesh, S. Deepr: A Convolutional Net for Medical Records. *arXiv. org* 22–30 (2016).

43. Miotto, R., Li, L., Kidd, B. A. & Dudley, J. T. Deep Patient: An Unsupervised Representation to Predict the Future of Patients from the Electronic Health Records. *Sci. Rep.* **6**, 1–10 (2016).

44. Suresh, H., Szolovits, P. & Ghassemi, M. The Use of Autoencoders for Discovering Patient Phenotypes. *arXiv. org* (2017).

45. Rajkomar, A. *et al.* Scalable and accurate deep learning with electronic health records. *npj Digital Medicine* **1**, 18 (2018).

46. Choi, E., Bahadori, M. T., Song, L., Stewart, W. F. & Sun, J. GRAM: Graph-based attention model for healthcare representation learning. In *Proceedings of the ACM SIGKDD International Conference on Knowledge Discovery and Data Mining*, 787–795. Sutter Health, Sacramento, United States (ACM Press, New York, New York, USA, 2017).

47. Rudin, C. Stop explaining black box machine learning models for high stakes decisions and use interpretable models instead. *Nature Machine Intelligence* 1–10 (2019).
