# Supplementary material for: Inferring multimodal latent topics from electronic health records
Source: Nat Commun. 2020 May 21;11:2536. doi: 10.1038/s41467-020-16378-3 (PMC7242436; doi:10.1038/s41467-020-16378-3)
Supplement: Supplementary file 4 — Description of Additional Supplementary Files [file 41467_2020_16378_MOESM4_ESM.pdf]

Description of Additional Supplementary Files

File Name: Supplementary Data 1
Description: Saves the 75-topic distribution over 52,868 binary phenotypes over 5 categories. Row names has format x:y, where x is the name of the category and y is the name of the phenotype term

File Name: Supplementary Data 2
Description: Saves the 75-topic distribution over 564 lab tests for the NMAR distribution

File Name: Supplementary Data 3
Description: Saves the 75-topic distribution of lab results for the 564 lab tests. Here stateId = 0 indicate normal and stateId = 1 indicate abnormal

File Name: Supplementary Data 4
Description: Contains columns as admission ID, lab ID, lab name, MixEHR predicted lab results, CF-RBM predicted lab results, and true lab results.

File Name: Supplementary Data 5
Description: Contains Mayo Clinic Bipolar Disorder patient mixture: 20-topic mixture for 187 patient and case and control labels to generate the heatmap in Figure 4d of the main text.

File Name: Supplementary Data 6
Description: Contains MixEHR-Topics learned from Quebec CHD dataset. These are topics learned from visit blocks for 64,000 patients (i.e., 80% of the cohort) over 28 years from the Quebec CHD Database. The columns are the 50-topic probabilities. The row names are the intervention code (starting with prefix “1-“) and ICD-9 code (start with prefix “2-“).

File Name: Supplementary Data 7 & 8
Description: These files show AUROC and AUPRC for EMB100 (baseline RNN using 100-dimension embedding dense layer) and EMB50+MXR50 (proposed MixEHR+RNN using 50-dimensional embedding dense layer and 50-topic mixture from MixEHR).

File Name: Supplementary Software 1
Description: Encloses the source code for MixEHR. Please see README file for its usage. MixEHR is also on our GitHub repository (https://github.com/li-lab-mcgill/mixehr) with detailed tutorials.
