# Supplementary figures and images for: Inferring multimodal latent topics from electronic health records
Source: Nat Commun. 2020 May 21;11:2536. doi: 10.1038/s41467-020-16378-3 (PMC7242436; doi:10.1038/s41467-020-16378-3)

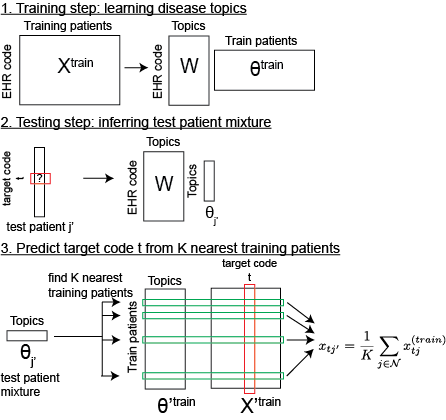

Supplement: Supplementary file 13 — Supplementary Software 1 [file 41467_2020_16378_MOESM13_ESM.zip › Supplementary_software/mixehr/images/ehr_code_prediction.png]

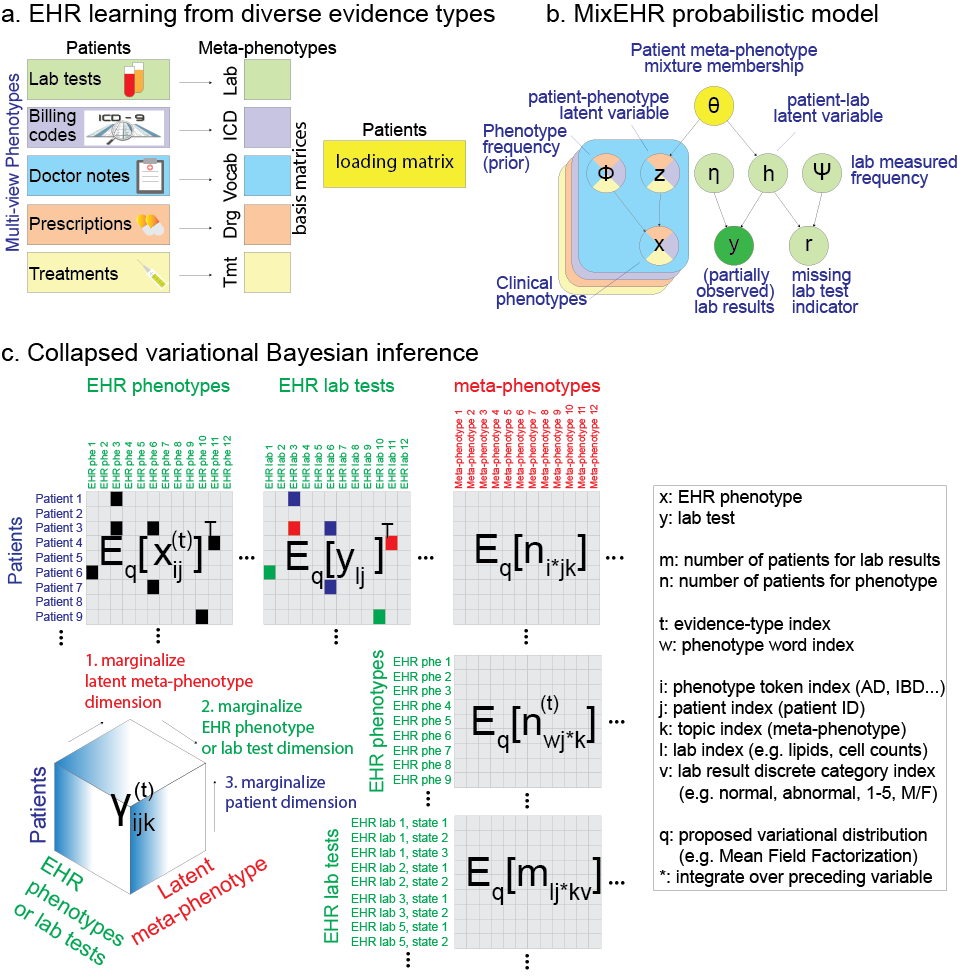

Supplement: Supplementary file 13 — Supplementary Software 1 [file 41467_2020_16378_MOESM13_ESM.zip › Supplementary_software/mixehr/images/mixehr_overview.png]

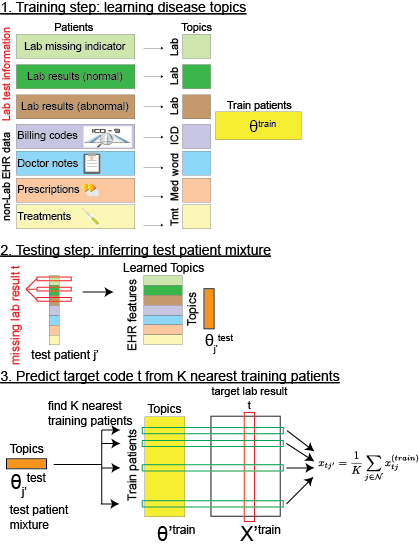

Supplement: Supplementary file 13 — Supplementary Software 1 [file 41467_2020_16378_MOESM13_ESM.zip › Supplementary_software/mixehr/images/lab_imputation.png]

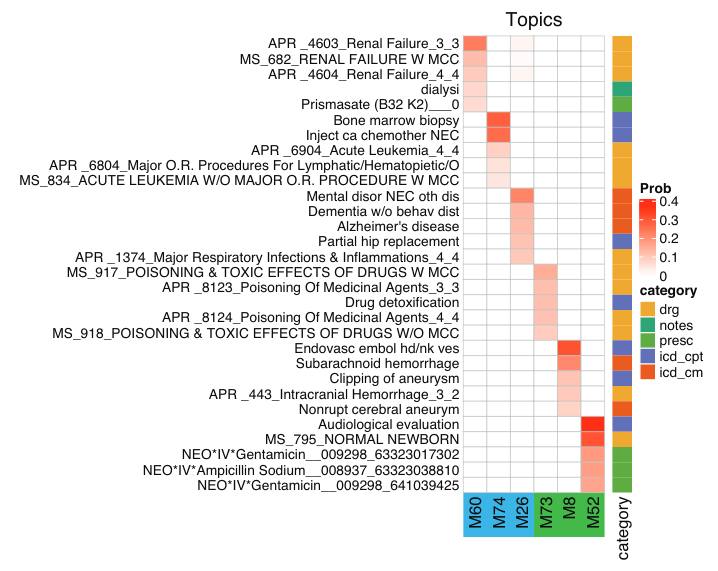

Supplement: Supplementary file 13 — Supplementary Software 1 [file 41467_2020_16378_MOESM13_ESM.zip › Supplementary_software/mixehr/images/heatmap.png]

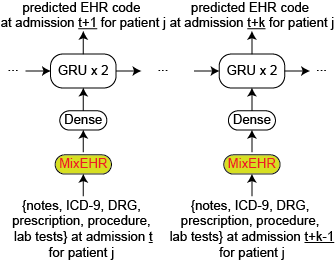

Supplement: Supplementary file 13 — Supplementary Software 1 [file 41467_2020_16378_MOESM13_ESM.zip › Supplementary_software/mixehr/images/code_prediction.png]
